# Genetic evidence for the causal relations between metabolic syndrome and psychiatric disorders: a Mendelian randomization study

**DOI:** 10.1038/s41398-024-02759-5

**Published:** 2024-01-20

**Authors:** Xue Gao, Yi Qin, Shu Jiao, Junhui Hao, Jian Zhao, Jiale Wang, Yanchao Wen, Tong Wang

**Affiliations:** 1 Department of Health Statistics, School of Public Health, Shanxi Medical University, 56 Xinjiannanlu Street, Taiyuan, Shanxi 030001 China; 2 MOE Key Laboratory of Coal Environmental Pathogenicity and Prevention, Shanxi Medical University, Taiyuan, China; 3 School of Public Health and Emergency Management, Southern University of Science and Technology, 1088 Xueyuan Avenue, Shenzhen, Guangdong 518055 China; 4 MRC Integrative Epidemiology Unit, University of Bristol, Bristol, UK

**Keywords:** Psychiatric disorders, Pathogenesis

## Abstract

Emerging evidence reveals associations between metabolic syndrome (MetS) and psychiatric disorders (PDs), although causality remains uncertain. Consequently, we conducted Mendelian randomization (MR) to systematically evaluate the causality between MetS and PDs. Linkage disequilibrium score regression estimated the heritability of PDs and their genetic correlations with MetS. In primary analyses, the main model employed inverse variance weighting method, with sensitivity analyses using various MR models to ensure robustness. Replication MR analyses, involving cohorts distinct from those in the primary analyses, were performed to validate the generalizability of the findings. Multivariable MR analyses were carried out to account for genetically predicted body mass index (BMI). As a result, genetic correlations of MetS with attention-deficit/hyperactivity disorder(ADHD), anorexia nervosa(ANO), major depressive disorder(MDD), and schizophrenia were identified. Causal effects of MetS on ADHD (OR: 1.59 [95% CI:1.45–1.74]), ANO (OR: 1.42 [95% CI:1.25–1.61]), MDD(OR: 1.23 [95% CI: 1.13–1.33]), and the effects of ADHD (OR: 1.03 [95% CI: 1.02–1.04]) and ANO (OR: 1.01 [95% CI: 1.01–1.02]) on MetS were observed in primary analyses. Results from sensitivity analyses and replication analyses were generally consistent with the primary analyses, confirming the robustness and generalizability of the findings. Associations between MetS and ADHD, as well as ANO persisted after adjusting for BMI, whereas the statistical significance of the association between MetS and MDD was no longer observable. These results contribute to a deeper understanding of the mechanisms underlying PDs, suggesting potential modifiable targets for public prevention and clinical intervention in specific PDs related to metabolic pathways.

## Introduction

Metabolic syndrome (MetS) is a persistent global public health challenge characterized by a combination of phenotypic traits, including elevated diastolic or systolic blood pressure, increased fasting glucose and triglycerides (TG), augmented waist circumference, and reduced high-density lipoprotein cholesterol (HDL-C). This cluster of traits triggers more severe risks for adverse clinical outcomes compared to any single risk component [1, 2]. MetS significantly elevates the likelihood of various physical disorders and complications. For example, individuals with MetS face a fivefold increase in the risk of developing type 2 diabetes and are twice as likely to develop cardiovascular disease compared to their healthy counterparts [3–5].

Furthermore, substantial evidence underscores the association between MetS and an increased prevalence of psychiatric disorders (PDs). On average, the prevalence of MetS is 58% higher in individuals with PDs compared to the general population, and those with severe mental disorders also face an elevated risk of developing MetS in comparison to the general population [6]. Multiple studies have provided evidence of the interconnections and shared pathways between depression and MetS [7]. Moreover, a systematic review has identified a bidirectional relationship between MetS and the occurrence as well as severity of bipolar disorder (BIP) [8].

The increased prevalence and substantial comorbidity of MetS and PDs have instigated a systematic investigation into the causal mechanisms, which aims to prioritize or refine disease progression and therapeutic strategies. However, the understanding of pathogenic mechanisms remains incomplete, primarily due to the challenges associated with the gold standard for assessing causality, randomized clinical trials (RCTs), which can be arduous to conduct from both ethical and practical perspectives. In addition, the presence of confounding factors, such as external life environment, the use of antidepressant medications, social discrimination related to obesity, and unhealthy lifestyles, poses difficulties in accurately estimating the true causal effects of MetS on PDs in observational studies [9].

As an alternative to RCTs, Mendelian randomization (MR) has emerged as a predominant method for gaining insights into the causes of diseases. MR employs genetic variants as instrumental variables (IVs) to evaluate the causal effect of exposure on the outcome, leveraging the fact that genetic variants are determined before exposure and outcome and that parental alleles are randomly assigned to offspring, therefore mitigates potential interference from confounding factors and reverse causality [10]. The burgeoning treasure trove of genotype-phenotype associations derived from genome-wide association studies (GWASs) facilitates the MR causal inference across a broad range of phenotypes and related outcomes.

In this study, we utilized the MR framework to assess the causal relationship between MetS and 10 PDs, namely attention-deficit/hyperactivity disorder (ADHD), alcohol dependence (ALC), anorexia nervosa (ANO), anxiety (ANX), autism spectrum disorder (ASD), BIP, major depressive disorder (MDD), schizophrenia (SCZ), post-traumatic stress disorder (PTSD), and Tourette’s syndrome (TS).

## Subjects and methods

### Exposure data

**Fig. 1:**
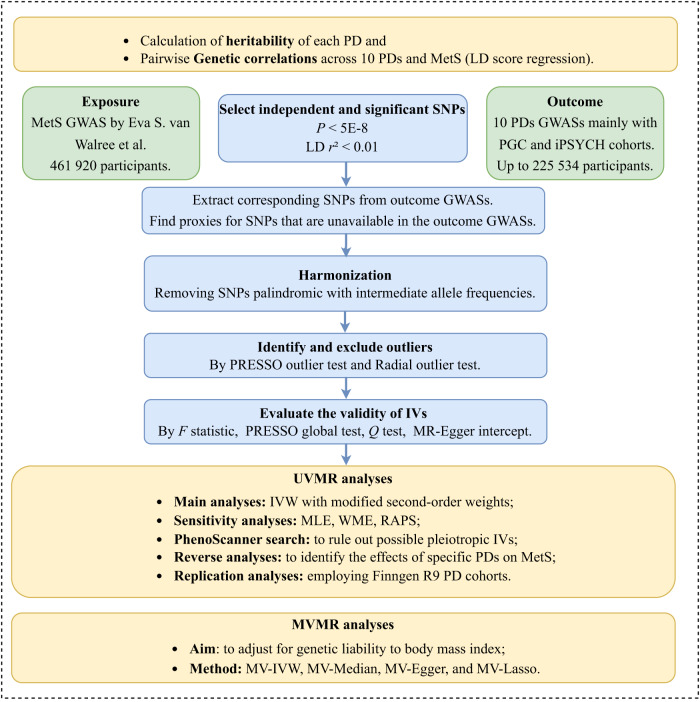
Flowchart illustrating data collection, processing, and analysis procedures. Abbreviations: PDs psychiatric disorders, LD linkage disequilibrium, MetS metabolic syndrome, GWAS genome-wide association study, IVs instrumental variables, SNPs single nucleotide polymorphisms, PRESSO pleiotropy residual sum and outlier, UVMR univariable Mendelian randomization analyses, IVW inverse variance weighting, MLE maximum likelihood estimator, WME weighted median estimator, RAPS robust adjusted profile score, MVMR multivariable MR analyses.

A schematic framework of this study is depicted in Fig. 1. The MetS GWAS was conducted using the genetic single MetS factor, which is derived from the genomic structural equation modeling [11]. The MetS factor captured and summarized genetic correlations and shared genetic variance among five metabolic components. Details on the GWAS cohorts for these components are outlined in Supplementary Table 1.

The MetS factor GWAS exhibited an effective sample size of 461,920 and an observed scale single nucleotide polymorphism (SNP)-based heritability (${h}_{{SNP}}^{2}$) of 0.14, surpassing the largest MetS GWAS to date (with a sample size and ${h}_{{SNP}}^{2}$ of 291,107 and 0.09, respectively) [12]. Consequently, the MetS factor GWAS was utilized to IVs in the primary analysis.

### Outcome data

Our principles for selecting outcome data aimed to maximize sample size while avoiding sample overlap. Since the MetS GWAS included participants from the UK Biobank (UKB), we excluded the UKB cohort from outcome datasets whenever possible. To mitigate potential bias from population stratification, we confined the individuals in the outcome GWAS to European ancestry, aligning with the MetS sample. Following these criteria, GWAS summary statistics of 10 PDs were collected from the largest publicly available datasets (Table 1).

**Table 1:** Selected GWAS summary statistics of PDs included in this study.

	N cases/N controls	N variants	Study cohort(s)	PubMed ID	First Author (Year)
ADHD	38,691	6,774,224	iPSYCH+deCODE+PGC data	36702997	Ditte Demontis (2023)
	186,843				
ALC	8485	9,142,831	PGC	30482948	Raymond K Walters (2018)
	20,272				
ANO	16,992	8,219,102	ANGI + PGC-ED+WTCCC-3 + UKB	31308545	Hunna J Watson (2019)
	55,525				
ANX	4584	8,029,716	iPSYCH	31116379	Sandra M Meier (2019)
	19,225				
ASD	18,382	9,112,386	iPSYCH+PGC	30804558	Jakob Grove (2019)
	27,969				
BIP	20,352	13,413,244	PGC	31043756	Eli A Stahl (2019)
	31,358				
MDD	45,396	9,874,289	PGC	29700475	Naomi R Wray (2018)
	97,250				
SCZ	53,386	7,659,767	PGC	35396580	Vassily Trubetskoy (2022)
	77,258				
PTSD	2424	13,207,411	PGC	28439101	L E Duncan (2018)
	7113				
TS	4819	8,265,318	PGC	30818990	Dongmei Yu (2019)
	9488				

*PDs* psychiatric disorders, *ADHD* attention-deficit/hyperactivity disorder, *ALC* alcohol dependence; *ANO* anorexia nervosa, *ANX* anxiety, *ASD* autism spectrum disorder, *BIP* bipolar disorder, *MDD* major depressive disorder, *SCZ* schizophrenia, *PTSD* post-traumatic stress disorder, *TS* Tourette’s syndrome.

Among them, six PDs (ALC, BIP, MDD, SCZ, PTSD, and TS) had GWAS derived from the Psychiatric Genomics Consortium (PGC) cohort, while the GWAS of ANX was sourced from the Danish Lundbeck Foundation Initiative for Integrative Psychiatric Research (iPSYCH) cohort [13–19]. Summary statistics for ASD were obtained from a meta-analysis of PGC and iPSYCH, and summary statistics for ADHD were extracted from PGC, iPSYCH, and the Icelandic deCODE cohort [20, 21]. For ANO, we utilized the largest GWAS meta-analysis involving four cohorts: the Eating Disorders Working Group of the PGC (PGC-ED) Freeze, the Anorexia Nervosa Genetics Initiative (ANGI), the Wellcome Trust Case Control Consortium-3 (WTCCC3), and the UKB. The total sample size of these cohorts was 72,517, including 3840 (5.2%) participants from the UKB [22].

All mentioned cohorts, except for PTSD, comprised individuals of of European ancestry, while PTSD participants were European-Americans defined as having ≥90% European ancestry. Disease diagnoses for each PD are detailed in Supplementary Note 1. As all analyses were based on publicly available summary data, ethical approval from an institutional review board and informed consent from the subjects were obtained in the original studies.

### Calculation of heritability and genetic correlation

Linkage disequilibrium score regression (LDSC) is a powerful tool for evaluating ${h}_{{SNP}}^{2}$ of traits and genetic correlations (*r*_*g*_) between different traits based on the full GWAS summary data [23]. Here LDSC was applied to estimate the observed scale ${h}_{{SNP}}^{2}$ of each PD trait, and to quantify the pairwise genetic correlations of the 10 PD traits and their correlation with MetS. Population-matched LD scores were calculated using with the European-ancestry samples in the 1000 Genomes Project as the reference panel [24]. To address multiple hypothesis testing, a Bonferroni adjusted *P*-value of 0.0009 (0.05/55, where 55 = 11 × 10/2) was applied as the significance threshold for LDSC analyses.

### Selection of IVs

The validity of IVs for MR causal inference relies on three core assumptions (Supplementary Note 2). To ensure the authenticity and accuracy of the conclusions regarding the causal link between MetS and PD risk, the following steps were taken to select optimal IVs. SNPs with a minor allele frequency < 1% and INFO score < 0.9 (where available) in the MetS summary datasets were removed for variant quality control. Subsequently, SNPs from MetS GWAS under the genome-wide significant level (*P* < 5 × 10^−8^) were selected and clumped with an LD *r*^2^ of 0.01 and a window size of 1MB, ensuring strength and independence among the IVs [25]. The summary statistics of the selected SNPs were then extracted from the PD GWASs. Unavailable SNPs in the outcome GWAS were substituted by genetic variants with a minimum LD *r*^2^ = 0.6 and base pair window of 500 000 in European populations, or excluded if no suitable proxies were identified. In addition, a harmonization process was carried out to align alleles to the human genome reference sequence (build 38) and ensure that the effect of SNPs on exposure and outcome corresponds to the same allele, while palindromic SNPs that could not be inferred were excluded.

To minimize the risk of horizontal pleiotropy, where a variant influences PD outcomes through traits other than MetS, the MR pleiotropy residual sum and outlier (PRESSO) test and the MR-Radial outlier test were performed to detect evidence of heterogeneity, which might be caused by pleiotropy to a large extent [26, 27]. The remaining SNPs constituted the final set for each MR analysis, as detailed in Supplementary Tables 2–11.

### Evaluation of IVs

The validity of IVs was assessed through various dimensions. *F* statistics were computed to gauge the strength of the IVs, with an *F* statistic greater than 10 generally considered sufficiently strong to mitigate weak instrument bias [28]. The PRESSO global test and Cochran’s *Q* test were employed to examine overall heterogeneity across the IVs [26, 27]. MR-Egger regression intercept was estimated to detect directional pleiotropy among the IVs [29]. A statistically significant non-zero intercept indicated unbalanced pleiotropy, suggesting that the pleiotropic effects of the invalid instruments could not counteract each other, potentially biasing the causal estimators. In addition, the proportion of phenotypic variance (PVE) explained by the IVs and post-hoc statistical power were estimated to ascertain whether the MR models possessed adequate capability to detect the causal effects of exposures on outcomes. Detailed estimation methodologies for assessing indicators, including *F* statistics, PVE, and statistical power, are available in Supplementary Note 3.

### Univariable MR (UVMR) analyses

The main MR method utilized was the inverse variance weighting (IVW) model with modified second-order weights, complemented by sensitivity analyses employing maximum likelihood estimator (MLE), weighted median estimator (WME), and robust adjusted profile score (RAPS) [27, 30–32]. The profile of each MR analytical model is provided in Supplementary Note 4. In addition, using the PhenoScanner GWAS database, instrument SNPs previously associated with potential known confounding factors were identified, and causality was reassessed after excluding these potential pleiotropic IVs [33].

Furthermore, the effects of PDs on MetS were explored when the causal effects of MetS on PDs were significant. The same process for selecting instruments for MetS was followed, with a less stringent significance level (*P* < 5 × 10^−5^) chosen as no SNPs were associated with multiple PDs at the genome-wide significance level. The final IV sets are detailed in Supplementary Tables 12–14.

In addition, a replication study was conducted using the same analytical strategy as described for the primary MR analysis. The MetS GWAS data were obtained from van Walree ES’s study, and PDs GWASs data were sourced from the FinnGen R9 database, to investigate the association between MetS and PDs [11, 34]. Detailed information, including the exact number of cases and controls, the online depository of the PD GWASs summary statistics can be found in Supplementary Table 15. We established a multiple testing significance threshold defined as *P* < 0.05/10 PDs for the above UVMR analyses.

### Multivariable MR (MVMR) analyses

For the observed causality between MetS and specific PDs, we conducted additional MVMR analyses to assess the impact of MetS on these PDs while controlling for body mass index (BMI), which is acknowledged as a confounding factor in the MetS-PD relationship [35]. Summary statistics for BMI were derived from a meta-analysis of UKB and the Genetic Investigation of ANthropometric Traits (GIANT) consortium, with an average sample size of 681,275 [36]. We identified the union set of SNPs significantly associated with either risk factor (MetS or BMI) using a threshold of *P* < 5 × 10^−8^. Independent SNPs with LD *r*^2^ < 0.01 were then extracted as candidate IVs. The first-stage conditional *F* statistics were calculated to evaluate the instrument strength of MetS, conditional on BMI. Estimators were obtained using the IVW, Median, Egger, and Lasso models.

### Online tools

LDSC was employed to calculate ${h}_{{SNP}}^{2}$ and *r*_*g*_ (https://github.com/bulik/ldsc), the LDlink platform (https://analysistools.cancer.gov/LDlink/?tab=ldproxy) and the power calculation tool (https://sb452.shinyapps.io/power/) were utilized to identify SNP proxies and estimate statistical power respectively. Various analyses, including LD clumping of SNPs, data harmonization, outlier detection, and MR causal inference, were conducted using R version 4.2.2 (R Foundation for Statistical Computing, Vienna, Austria) packages including MendelianRandomization, MR-PRESSO, RadialMR, TwoSampleMR, and MR-RAPS.

## Results

### Heritability and genetic correlation

All the PDs had ${h}_{{SNP}}^{2}$ higher than 0.05, except for ANX, the highest ${h}_{{SNP}}^{2}$ at 0.40 (standard error [SE] = 0.05) for TS and the lowest at 0.010 (SE = 0.02) for ANX. Significant genetic correlations were observed between MetS and four PDs, including ADHD (*r*_*g*_ = 0.33, SE = 0.02, *P* = 5.60 × 10^−5^), ANO (*r*_*g*_ = 0.35, SE = 0.03; *P* = 4.38 × 10^−39^), MDD (*r*_*g*_ = 0.15, SE = 0.03; *P* = 1.16 × 10^−7^), and SCZ (*r*_*g*_ = −0.10, SE = 0.02; *P* = 9.72 × 10^−8^), indicating a shared genetic architecture between MetS and these PDs (Table 2). Furthermore, the PDs showed low to moderate genetic correlations, and the pairwise correlation across 10 PD factors are displayed in Supplementary Table 16 and Supplementary Fig. 1.

**Table 2:** Heritability estimates for PD traits and genetic correlation with MetS.

	${h}_{{SNP}}^{2}$	${\rm{SE}}({h}_{{SNP}}^{2})$	*r*_*g*_	SE(*r*_*g*_)	*P*
ADHD	0.10	0.00	0.33	0.02	5.60 × 10^−50^
ALC	0.07	0.02	0.18	0.06	0.004
ANO	0.18	0.01	0.35	0.03	4.38 × 10^−39^
ANX	0.01	0.02	0.77	2.38	0.745
ASD	0.20	0.02	0.05	0.03	0.086
BIP	0.36	0.02	−0.04	0.02	0.068
MDD	0.07	0.00	0.15	0.03	1.16 × 10^−7^
SCZ	0.37	0.01	−0.10	0.02	9.72 × 10^−8^
PTSD	0.14	0.06	0.27	0.09	0.001
TS	0.40	0.05	0.00	0.04	0.900

${h}_{{SNP}}^{2}$ heritability, *SE* standard error, *r*_*g*_ genetic correlation, *PD* psychiatric disorder, *MetS* metabolic syndrome, *ADHD* attention-deficit/hyperactivity disorder, *ALC* alcohol dependence, *ANO* anorexia nervosa, *ANX* anxiety, *ASD* autism spectrum disorder, *BIP* bipolar disorder, *MDD* major depressive disorder, *SCZ* schizophrenia, *PTSD* post-traumatic stress disorder, *TS* Tourette’s syndrome.

### Evaluation of IVs

Following genetic quality control, a total of 46–235 SNPs were selected for the primary MR analyses, and 14–236 SNPs for the replication MR analyses. All screened instruments exhibited *F* statistic exceeding 10, indicating a reduced susceptibility to weak instrument bias. The *P* values obtained from the PRESSO global test, Cochran’s *Q* statistic test, and MR-Egger intercept test of deviation from the null were consistently greater than 0.05, suggesting limited evidence of heterogeneity or directional pleiotropy among the SNP effects. Moreover, the majority of MR models demonstrated sufficient statistical power (>80%) to detect a significant causal effect (Supplementary Table 17).

### MR analyses

Primary analyses with the IVW model revealed that genetically predicted MetS exhibited causal effects on increased risks of three PDs: ADHD (OR = 1.59, 95% CI: 1.45–1.74, *P* = 7.09 × 10^−23^), ANO (OR = 1.42, 95% CI: 1.25–1.61, *P* = 1.90 × 10^−7^), and MDD (OR = 1.23, 95% CI: 1.13–1.33, *P* = 8.06 × 10^−7^), respectively. Notably, significant genetic correlations were also identified in these three pairs of relationships. The estimated causal effect sizes via MLE, WME, and RAPS models were broadly consistent with the IVW model (Supplementary Table 18).

**Fig. 2:**
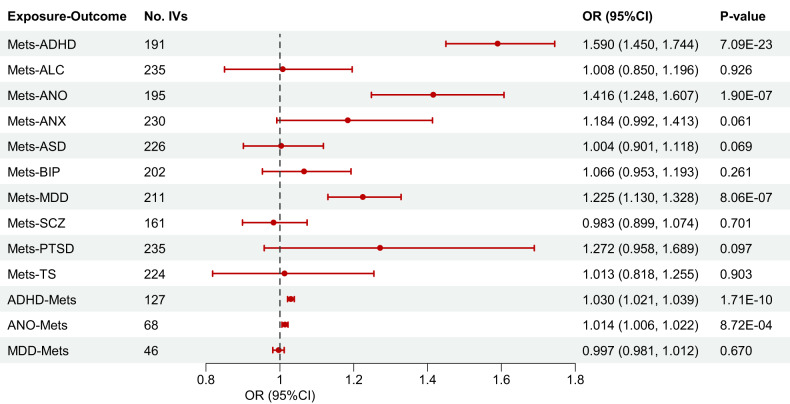
Causal relationships between MetS and PDs using UVMR. Abbreviations: MetS metabolic syndrome, PDs psychiatric disorders, UVMR univariable MR, ADHD attention-deficit/hyperactivity disorder, ALC alcohol dependence, ANO anorexia nervosa, ANX anxiety, ASD autism spectrum disorder, BIP bipolar disorder, MDD major depressive disorder, SCZ schizophrenia, PTSD post-traumatic stress disorder, TS Tourette’s syndrome, No. IVs number of instrumental variables, OR odds ratio, CI confidence interval.

In the PhenoScanner database, 12 unique IVs for MetS significantly associated with PD-relevant traits, and five unique IVs for PDs significantly associated with MetS-relevant traits were identified (Supplementary Tables 2–14). However, removing these SNPs did not alter the pattern of results (Supplementary Table 18). Subsequent exploration of the causal effects of ADHD, ANO, and MDD on MetS indicated significant causality from ADHD (OR = 1.03, 95% CI: 1.02–1.04, *P* = 1.71 × 10^−10^) and ANO (OR = 1.01, 95% CI: 1.01–1.02, *P* = 8.72 × 10^−4^) to MetS. Conversely, there was no evidence supporting a causal effect of MDD on MetS (Fig. 2).

Causal associations were observed between MetS and ANO and MDD in the replication analyses, with consistent results across all sensitivity analyses. However, the causal effect of MetS on ADHD, as well as the causal effects of ANO and MDD on MetS showed compatibility with the null. This might be attributed to the smaller case numbers in the replication analyses for ADHD and ANO GWAS cohorts (with 2340 and 1897, respectively), compared to larger case numbers in the primary analyses (with 38,691 and 16,992, respectively)(Supplementary Table 18).

In the MVMR analyses, utilizing an average of approximately 1000 instruments for both exposures, the mean first-stage conditional *F* statistics all exceeds 10, indicating minimal evidence of potential bias due to weak instruments. The associations between MetS and ADHD, as well as ANO, remained statistically significant even after adjusting for genetic liability to BMI. However, the previously observed association between MetS and MDD was no longer statistically evident (Supplementary Table 18).

## Discussion

Despite the absence of definitive conclusions, prior studies have uncovered extensive insights into the connections between MetS and PDs. Untangling whether these associations are causal remains challenging due to confounding factors and the potential for reverse causality. To address this, we utilized a two-sample MR framework to investigate the role of MetS in 10 major PDs, the results substantiate an adverse effect of MetS on three specific PD outcomes. Notably, within the MR framework, we assessed the impact of a specific cluster of genetic variants associated with MetS on PDs. It is conceivable that additional genetic components or non-genetic factors, including postnatal environmental and social influences, operating through alternative biological mechanisms, contribute to variations in MetS. Consequently, these factors may give rise to diverse causal effects on PDs, a complexity not captured by the MR analysis [37].

In this study, we identified a significant genetic correlation and bidirectional causal relationship between MetS and ADHD, aligning with some previous studies. A diagnostic instrument-based investigation revealed that overweight children with higher BMI exhibited an elevated risk of developing ADHD symptoms [38]. Meanwhile, ADHD emerged as a risk factor for MetS components [39, 40]. The study by Zohar Landau and Orit Pinhas-Hamiel not only confirmed the link between ADHD and obesity, diabetes, and hypertension, but also proposed various underlying mechanisms [41]. Previous research suggested a connection between ADHD, obesity and the dopamine system [42], positing that dopaminergic alterations in the prefrontal cortex of ADHD patients with the attention deficit disorder subtype might heighten their obesity risk [43]. Further studies indicated that changes in the hypocretin/orexin system contribute to impaired alertness and abnormal feeding behaviors in ADHD patients, and this mechanism corroborated in animal models [44].

The observed significant genetic correlation and bidirectional causality between MetS and ANO in this study also align with several prior investigations. A meta-analysis revealed elevated levels of total cholesterol, HDL, LDL, TG, and apolipoprotein B in acute ANO patients compared to controls, possibly due to increased exogenous lipid absorption resulting from intestinal ecology imbalance [45]. Research on adolescents suggested ANO induced damage to liver function as a potential cause for hypercholesterolemia and reduced clearance rates of many steroid hormones [46]. Moreover, hyperactivity of the hypothalamic-pituitary-adrenal (HPA) axis during fasting in ANO patients can lead to increaseD plasma cortisol levels [47]. Changes in these factors may alter metabolic levels and heighten the risk of MetS. Various potential biological mechanisms linking MetS to ANO include alterations in the gut microbiome, central and peripheral immune dysregulation, and endocrine disorders [9].

Our findings also supported a significant genetic correlation and causal relationship between MetS on MDD, consistent with certain observational studies and MR analyses. However, our results provide more robust evidence of causality due to a larger sample size [48–50]. One possible biological mechanism explaining the impact of MetS on MDD involves the HPA axis. HPA axis activation can elevate cortisol levels, and metabolic abnormalities may induce HPA axis overactivity and peripheral changes in cortisol metabolism, a common neuroendocrine abnormality in MDD [51–53]. Another possible pathway connecting the MetS and depression is inflammation. High-fat diets and obesity triggering an inflammatory response that may lead to depressive symptoms (such as insufficient sleep, lack of pleasure, and anorexia) [54]. Key inflammatory cytokines such as C-reactive protein, tumor necrosis factor-α, interferon-γ, and interleukin (IL)-6 and IL-8 also correspond with symptoms of major depressive symptoms [55]. Controlling for BMI diminishes the significance of the association between Metabolic Syndrome (MetS) and Major Depressive Disorder (MDD). This suggests a stronger biological and genetic link between BMI-related components of MetS (such as WC) and MDD when compared to the other components within MetS.

These findings contribute novel perspectives to the formulation of public health intervention policies. In conjunction with the results, employing relatively achievable and cost-effective practices such as modifying dietary habits, promoting physical activities, and monitoring blood pressure and related indicators in a timely manner emerges as a practical approach. This approach aims to enhance metabolic levels and consequently diminish the risk of PDs, including ADHD, ANO, and MDD. Such strategies are worth exploring in the context of public health interventions.

The implications of these findings extend to clinical practices, offering insights for developing innovative treatment strategies for PDs. The bidirectional causal relationships between MetS and ADHD or ANO provide tangible evidence for considering metabolic factors in the diagnostic criteria for PDs. In addition, the results prompt an exploration of the effectiveness and safety of anti-metabolic disruption therapies and pharmacological interventions for the treatment and management of these PDs. Furthermore, these findings open new avenues for the drug target MR studies, aiming at identifying the effects of MetS-related protein targets on specific PDs and subsequently evaluating the clinical validity of pharmaceutical ingredients corresponding to potential causal proteins to alleviate PD symptoms.

Our analyses possess several strengths. Firstly, we comprehensively covered a wide array of PDs and utilized a substantial sample size in the majority of our analyses. Secondly, the application of two-sample MR analyses was pivotal in mitigating issues related to reverse causality and confounding factors, typically challenging to address in conventional observational studies. In addition, by employing GWAS summary data with the maximum available sample size and ensuring the independence of PD cohorts from MetS data, we bolstered statistical power and minimized potential biases arising from sample overlap. Thirdly, our study employed a rigorous analytical framework, incorporating *F* statistics to assess instrument strength, extensive diagnostics to filter out the pleiotropic instruments, and sensitivity analyses to verify result robustness in estimating causal relationships between MetS and PDs.

It is noteworthy that MR models based on the summary data often assume a linear relationship between exposures and outcomes. However, the causal nature between MetS and PDs may be more intricate than we estimated in this study. Consequently, the evidence for causality merits nuanced consideration and confidence, emphasizing the need for further exploration of non-linear relationships when individual-level genetic data is accessible [56]. Furthermore, in the primary analyses, certain PDs, including ANX, PTSD, and TS, along with ADHD in the replication analyses, did not yield significant results, possibly due to insufficient statistical power in these MR analyses [57]. Lastly, the varying risk of some PDs in MetS across different populations (such as age and gender) remains an area of interest. However, the absence of stratified GWAS results based on age or gender hindered the exploration of causality in different sub-populations, underscoring the necessity for further investigation when relevant data become available.

In conclusion, to delve into the intricate relationships of MetS with psychiatric-related traits, we utilized the MR framework that leverages genetic variants as proxy instruments of MetS to delineate its genetic causal associations with various kinds of PDs. The results reveal significant effects of MetS on ADHD, ANO, and MDD. While the precise mechanism by which MetS influences PDs remains undetermined despite the recognized inferential causality in this study, our findings serve as initial insights for subsequent functional experiments and pathophysiological exploration, and also lay the fundamental basis for implications in potential interventions and therapeutic targets of PDs.

### Supplementary information

Supplementary materials

## Data Availability

In this study, we exclusively utilized publicly accessible GWAS summary data. The sources and corresponding download pathways for these data have been elucidated in the article, and there is no need for additional application or acquisition of other data.
