# Supplementary material for: Genetic evidence for the causal relations between metabolic syndrome and psychiatric disorders: a Mendelian randomization study
Source: Transl Psychiatry. 2024 Jan 20;14:46. doi: 10.1038/s41398-024-02759-5 (PMC10799927; doi:10.1038/s41398-024-02759-5)
Supplement: Supplementary file 1 — Supplementary materials [file 41398_2024_2759_MOESM1_ESM.pdf]

Supplementary appendix

Supplement to: Xue Gao *et al*. Genetic evidence for the causal relations between metabolic syndrome and psychiatric disorders: a Mendelian randomization study.

Index

Supplementary Notes

Supplementary Note 1. Additional information on psychiatric disorders.

Supplementary Note 2. Core assumptions of Mendelian randomization (MR).

Supplementary Note 3. F statistic, PVE, and Power estimation.

Supplementary Note 4. Profile of the MR analytical models.

References

Supplementary Figure 1. Heatmap of cross-trait genetic correlation between 10 psychiatric disorders.

Supplementary Tables

Supplementary Table 1. Cohorts included in the MetS components GWAS.

Supplementary Table 2. Genetic instruments for the causal relationship of Mets and ADHD.

Supplementary Table 3. Genetic instruments for the causal relationship of Mets and ALC.

Supplementary Table 4. Genetic instruments for the causal relationship of Mets and ANO.

Supplementary Table 5. Genetic instruments for the causal relationship of Mets and ANX.

Supplementary Table 6. Genetic instruments for the causal relationship of Mets and ASD.

Supplementary Table 7. Genetic instruments for the causal relationship of Mets and BIP.

Supplementary Table 8. Genetic instruments for the causal relationship of Mets and MDD.

Supplementary Table 9. Genetic instruments for the causal relationship of Mets and SCZ.

Supplementary Table 10. Genetic instruments for the causal relationship of Mets and

PTSD.

Supplementary Table 11. Genetic instruments for the causal relationship of Mets and TS.

Supplementary Table 12. Genetic instruments for the causal relationship of ADHD and Mets .

Supplementary Table 13. Genetic instruments for the causal relationship of ANO and Mets .

Supplementary Table 14. Genetic instruments for the causal relationship of MDD and Mets.

Supplementary Table 15. GWAS summary statistics of the replication study.

Supplementary Table 16. Genome-wide genetic correlations across the 10 PDs using LDSC.

Supplementary Table 17. Evaluation of instrumental variables.

Supplementary Table 18. MR results.

Supplementary Note 1. Additional information on psychiatric disorders.

Attention deficit/hyperactivity disorder (ADHD)

The ADHD GWAS summary statistics by Ditte Demontis et al. (2023) are based on the Danish Lundbeck Foundation Initiative for Integrative Psychiatric Research (iPSYCH), deCODE, and Psychiatric Genomics Consortium (PGC) cohorts. All cohorts were case-control studies, and cases met the criteria for ADHD as defined in The International Statistical Classification of Diseases and Related Health Problems-10th Revision (ICD-10) diagnosis code or medication prescription. The combined sample size included 38 691 cases and 186 843 controls. All participants were of European ancestry.

Alcohol dependence (ALC)

The ALC GWAS summary statistics Raymond K. Walters et al. (2018) are based on 14 case-control studies and nine family-based studies. Cases met the criteria for ALC as defined in the Diagnostic and Statistical Manual of Mental Disorders -Fourth Edition (DSM-IV) or DSM-IIIR in one instance. Individuals with no history of drinking alcohol and those meeting criteria for DSM-IV alcohol abuse were excluded as controls where possible. The summary statistics of the case-control GWAS analyzing 28 757 individuals of European uncorrelated individuals (8 485 cases and 20 272 controls) were used in the current study.

Anorexia Nervosa (ANO)

The ANO GWAS summary statistics by Watson et al. (2019) are based on four cohorts of European ancestry. All cohorts were case-control studies, and cases met the criteria for ANO as defined in the DSM-III-R or DSM-IV, or ICD-8, ICD-9, or ICD-10 (apart from the UK Biobank cohort, in which case/control status was defined through self-reported diagnosis history). The combined sample size included 16 992 cases and 55 525 controls.

Anxiety (ANX)

The ANX GWAS summary statistics by Sandra M. Meier et al. (2019) are based on a cohort of European ancestry. All study participants were enrolled in iPSYCH, a study

designed to unravel risk factors of severe mental disorders, including individuals with schizophrenia, autism, ADHD, ANO, and affective disorders, as well as population-based controls. Individuals with comorbid autism were excluded. Although patients with autism experience anxiety, their anxiety is often reflecting their core autistic symptomatology and lacks the social component central to many anxiety and stress-related diagnoses. The participants were from a case-control study, and cases met the criteria for ANX as defined in the ICD-10 (F40.0-F41.9; F43.0-F43.9). The sample size included 4 584 cases and 19 225 controls.

Autism spectrum disorder (ASD)

The GWAS summary statistics on ASD by Grove et al. (2019) are based on six cohorts of European ancestry. Five PGC-ASD cohorts were family-based, and cases were defined according to research-based assessment criteria and clinical consensus diagnosis. The case-control iPSYCH-ASD cohort recruited cases through the Danish Psychiatric Central Research Registrar, and a psychiatrist assessed diagnosis following ICD-10 criteria. The combined sample size consisted of 18 381 cases and 27 969 controls.

Bipolar disorder (BIP)

The BIP GWAS summary statistics by Stahl et al. (2019) are based on an analysis of 32 cohorts from Europe, North America, and Australia. All participants were of European ancestry. A clinical diagnosis of lifetime BIP was established for all cases using structured assessment or checklists by a clinician, or through a review of medical history. All diagnoses followed DSM-IV, ICD-9, or ICD-10 criteria. The combined sample size consisted of 20 352 cases and 31 358 controls.

Major depressive disorder (MDD)

The MDD GWAS summary statistics by Wray et al. (2018) result from a case-control meta-analysis of seven cohorts with individuals of European ancestry. In six cohorts, case status was determined by a range of methods (self-reported medical history or symptoms, interviews, and national treatment registers), while in the remaining cohort, a review of medical history or structured assessment by a clinician following DSM-V, ICD-9, or ICD-10 criteria was applied. The total sample size of all cohorts was 135

458 cases and 344 901 controls. Due to data use restrictions and the exclusion criteria, the summary statistics used in the current study are based on the results excluding the 23andMe cohort (59 851 cases and 113 154 controls) and the UKB cohort (30 211 cases and 134 497controls).

Schizophrenia (SCZ)

The SCZ GWAS summary statistics presented by The Schizophrenia Working Group of the Psychiatric Genomics Consortium (2022) are based on an analysis of 91 cohorts. The GWAS recruit samples of European ancestry (~80% of the sample) and East Asian ancestry (~20%). Cases met clinical DSM-IV or ICD-10 criteria for either schizophrenia or schizoaffective disorder. At the time of analysis, European-only summary statistics include 53 386 cases and 77 258 controls.

Post-traumatic stress disorder (PTSD)

The PTSD GWAS summary statistics by L E Duncan et al. (2018) are based on 10 cohorts. Contributing studies provided individual-level genotype data or summary statistics consistent with their institutional review board-approved protocols. All participants were of European ancestry, and cases met the criteria for PTSD as defined in the self-report scale and DSM-IV. In many cohorts included in the consortium, all the controls were trauma-exposed (for example, Nurses Health Study II PTSD sub-study). Thus, in the combined analysis, the vast majority of controls were trauma-exposed. The summary statistics used in the current study are based on this case-control meta-analysis, the combined sample size included 2 424 cases and 7 113 controls.

Tourette's syndrome (TS)

The TS GWAS summary statistics by Yu et al. (2019) are based on an analysis of four cohorts of individuals of European descent. Three of the cohorts consisted of case-control studies, while the latter was a family-based cohort. Cases met the criteria for TS as defined by DSM-IV-Text Revision (DSM-IV-TR) or DSM-V, and were recruited by specialty clinics or through correspondence and clinical assessment online. The combined sample size consisted of 4 819 cases and 9 488 controls.

Supplementary Note 2. Core assumptions of Mendelian randomization (MR).

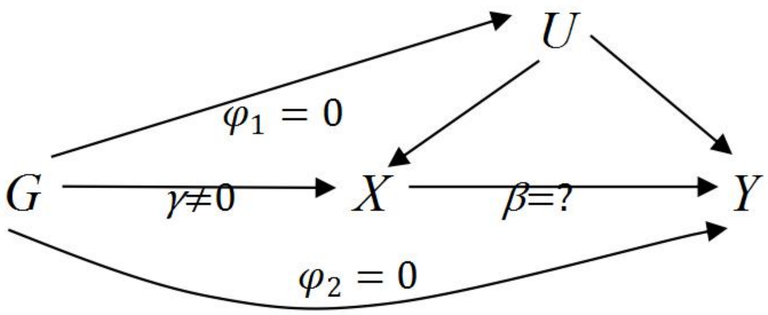

To acquire the causal effects (*β*) of exposure (*X*) on outcome(*Y*), the first assumption of MR is that the genetic variant (*G*) is robustly associated with *X* (relevance assumption, $\gamma \neq 0$), the second and third assumptions of MR are that there is no association between *G* and potential confounding factors (*U*) of the relationship between *X* and *Y* (independence assumption, $\varphi_1 = 0$), and that there is no direct association between the *G* and *Y* when conditioning on the exposure and the confounding factors(exclusion assumption, $\varphi_2 = 0$). Violation of the two assumptions indicates that the SNP affects the outcome outside of its effect on exposure, which can be referred to as horizontal pleiotropy.

Supplementary Note 3. F statistic, PVE, and Power estimation.

F statistic for each instrument was estimated with the formula[1]:

$$F = \beta_1^2 \,/\, var(\beta_1)$$

where β_1 is the effect size of SNP on exposure, Var(β_1) is the square of standard error of the β_1.

Proportion of phenotypic Variance Explained (PVE) for each instrument was estimated with the formula[2]:

$$PVE = \beta_1^2 \,/\, \left(var(\beta_1) * N_1 + \beta_1^2\right)$$

where N_1 corresponds to the sample size of the exposure GWAS.

The statistical power to detect a true causal effect of size β_2 with a significance level of 0.05 is[3]:

$$\Phi\left(\beta_2 \rho \sqrt{\left(N_2 \mathbb{P}(Y=1)\mathbb{P}(Y=0)\right)} - 1.96\right)$$

Here we assumed the true causal odds ratio (OR) of exposure and outcome β_2 was 1.2, and computed the post-hoc statistical power to detect an OR of 1.2 per 1-SD increase in the MetS factor, with type I error rate of 0.05. N_2 corresponds to the sample size of the outcome GWAS, and ρ^2 is the square of the correlation between the exposure and the instrument, which can be substituted by R^2: the coefficient of determination in the GWAS of the exposure. Here R^2 was calculated with sum of each instrument's PVE.

Supplementary Note 4. Profile of the MR analytical models.

The inverse variance weighting (IVW) with modified weights performs best when there are no invalid IVs, and can remove the regression dilution bias derived from weak instruments[4]. Maximum likelihood estimator (MLE) provides reliable estimates of the causal effect when IVs are subject to measurement bias or potential pleiotropy[5]. Weighted median estimator (WME) is consistent even when up to 50% of the information comes from invalid IVs[6]. Robust adjusted profile score (RAPS) leverages robustly adjusted profile scores to correct for the potential bias caused by pleiotropy[7]. The above approaches each entail different assumptions and together offer a range of sensitivity analyses that can be performed to help strengthen the evidence for causal inference[8].

References

1. Bowden J, Del Greco MF, Minelli C, Zhao Q, Lawlor DA, Sheehan NA et al. Improving the accuracy of two-sample summary-data Mendelian randomization: moving beyond the NOME assumption. *Int J Epidemiol* 2019;48(3):728-742. doi:10.1093/ije/dyy258

2. Zeng P, Zhou X. Causal Association Between Birth Weight and Adult Diseases: Evidence From a Mendelian Randomization Analysis. *Front Genet* 2019;10:618. doi:10.3389/fgene.2019.00618

3. Burgess S. Sample size and power calculations in Mendelian randomization with a single instrumental variable and a binary outcome. *Int J Epidemiol* 2014;43(3):922-929. doi:10.1093/ije/dyu005

4. Bowden J, Spiller W, Del Greco MF, Sheehan N, Thompson J, Minelli C et al. Improving the visualization, interpretation and analysis of two-sample summary data Mendelian randomization via the Radial plot and Radial regression. *Int J Epidemiol* 2018;47(6):2100. doi:10.1093/ije/dyy265

5. Lee T, Shi D. A comparison of full information maximum likelihood and multiple imputation in structural equation modeling with missing data. *Psychol Methods* 2021;26(4):466-485. doi:10.1037/met0000381

6. Bowden J, Davey Smith G, Haycock PC, Burgess S. Consistent Estimation in Mendelian Randomization with Some Invalid Instruments Using a Weighted Median Estimator. *Genet Epidemiol* 2016;40(4):304-314. doi:10.1002/gepi.21965.

7. Zhao QY, Wang JS, Hemani G, Bowden J, Small DS. Statistical inference in two-sample summary-data mendelian randomization using robust adjusted profile score. *Annals of Statistics* 2020;48(3):1742-1769. doi:10.1214/19-aos1866.

8. Burgess S, Bowden J, Fall T, Ingelsson E, Thompson SG. Sensitivity Analyses for Robust Causal Inference from Mendelian Randomization Analyses with Multiple Genetic Variants. *Epidemiology* 2017;28(1):30-42. doi:10.1097/ede.0000000000000559.

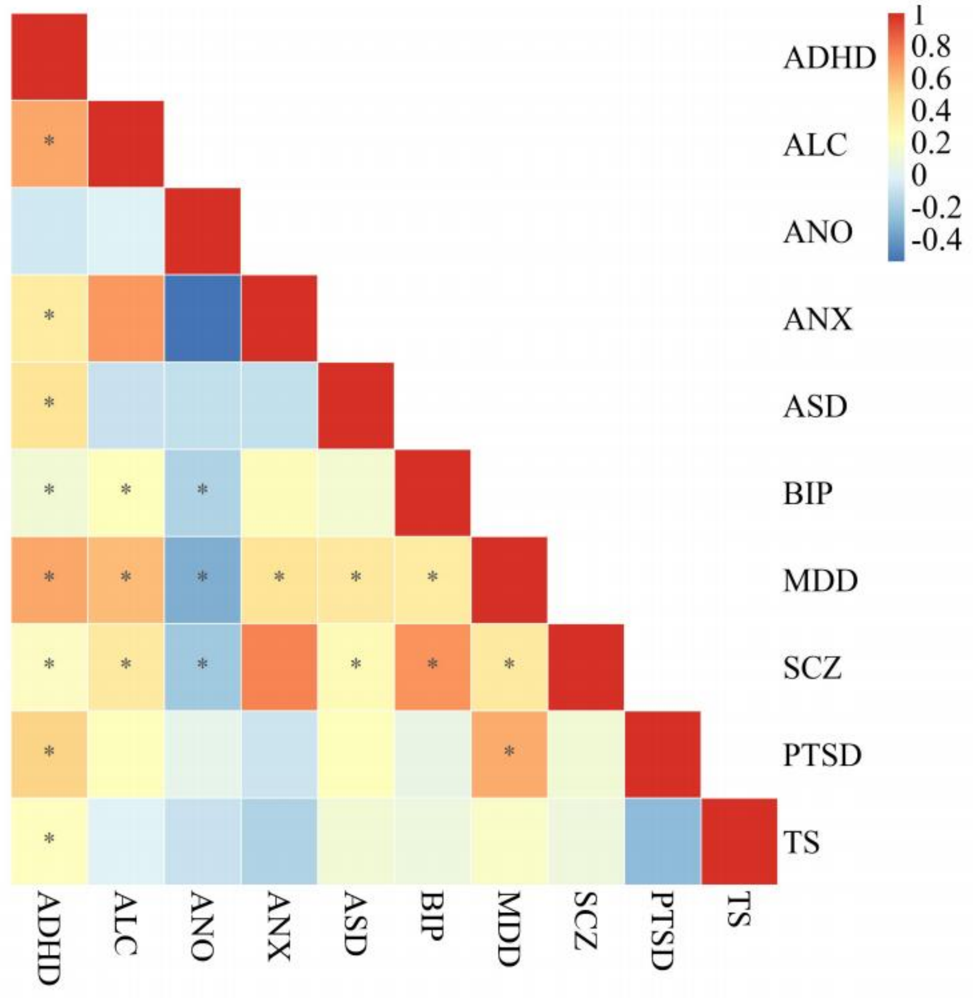

Supplementary Figure 1. Heatmap of cross-trait genetic correlation between 10 psychiatric disorders. Genetic correlations that are significant after Bonferroni correction ($P < 0.05/55$, where $55 = 11 \times 10/2$) are marked with an asterisk.

Supplementary Tables

Supplementary Table 1.Cohorts included in the MetS components GWAS.

Phenotype	Abbreviation	Study cohort(s)	No. of Participants	No. of Variants	Pubmed ID	Author(year)
Fasting glucose	FG	MAGIC	46 186	2 461 105	20081858	Dupuis et al. (2010)
High density lipoprotein	HDL-C	Global Lipids Genetics Consortium	99 000	2 692 429	20686565	Teslovich et al. (2010)
Systolic blood pressure	SBP	UK Biobank	361 402	10 534 620	31427789	Watanabe et al. (2019)
Triglycerides	TG	Global Lipids Genetics Consortium	96 598	2 692 560	20686565	Teslovich et al. (2010)
Waist circumference	WC	UK Biobank	385 932	10 599 054	31427789	Watanabe et al. (2019)

Supplementary Table 2. Genetic instruments for the causal relationship of Mets and ADHD.

SNP	CHR	Position	EA	OA	EAF	Beta for exposure	SE for exposure	P value for exposure	Beta for outcome	SE for outcome	P value for outcome	F statistics	PVE	Association with potential confounding factors
rs10116353	9	111950088	G	T	0.307	0.013	0.002	9.39E-09	0.021	0.010	0.035	32.964	7.14E-05	NA
rs10160660	11	76474537	T	C	0.248	0.013	0.002	4.83E-08	0.022	0.011	0.058	29.786	6.45E-05	NA
rs102275	11	61557803	T	C	0.363	-0.013	0.002	6.28E-11	-0.003	0.010	0.744	42.731	9.25E-05	NA
rs1032524	1	201790826	T	C	0.484	-0.015	0.002	6.01E-12	-0.013	0.009	0.174	47.325	1.02E-04	NA
rs10423928	19	46182304	T	A	0.214	0.020	0.003	8.49E-13	0.005	0.012	0.641	51.166	1.11E-04	NA
rs1046080	6	31595882	C	A	0.233	-0.015	0.003	1.75E-09	-0.012	0.010	0.254	36.231	7.84E-05	NA
rs10734924	12	123018894	T	G	0.259	-0.015	0.003	2.19E-09	0.004	0.011	0.707	35.801	7.75E-05	NA
rs10779835	1	230299949	T	C	0.391	0.015	0.002	2.45E-14	0.019	0.010	0.043	58.131	1.26E-04	NA
rs10808546	8	126495818	C	T	0.431	0.016	0.002	4.56E-19	0.004	0.009	0.689	79.612	1.72E-04	NA
rs10838620	11	46818098	G	A	0.214	-0.015	0.002	1.25E-09	-0.018	0.011	0.101	36.883	7.98E-05	NA
rs10872224	6	98435125	G	T	0.409	-0.014	0.002	9.76E-11	-0.005	0.010	0.591	41.868	9.06E-05	Educational attainment; Intelligence
rs10887579	10	88097210	A	C	0.354	-0.013	0.002	4.09E-09	0.000	0.010	0.956	34.579	7.49E-05	NA
rs10930502	2	172890588	A	G	0.304	0.013	0.002	1.18E-08	-0.006	0.010	0.575	32.519	7.04E-05	NA
rs10938397	4	45182527	A	G	0.420	-0.023	0.002	8.14E-26	-0.024	0.010	0.009	110.367	2.39E-04	NA
rs11079849	17	47090785	C	T	0.290	0.015	0.002	6.90E-11	0.013	0.010	0.187	42.547	9.21E-05	NA
rs11165643	1	96924097	C	T	0.417	-0.013	0.002	6.90E-10	-0.013	0.009	0.171	38.049	8.24E-05	NA
rs11187845	10	96060198	C	A	0.096	0.020	0.004	8.08E-09	0.033	0.017	0.054	33.256	7.20E-05	NA
rs1138429	16	56942921	A	T	0.101	-0.028	0.003	1.42E-18	-0.008	0.015	0.612	77.365	1.67E-04	NA
rs1159974	6	126090277	T	C	0.499	-0.013	0.002	3.30E-10	-0.008	0.009	0.373	39.489	8.55E-05	NA
rs11611246	12	939480	G	T	0.183	-0.016	0.003	1.14E-08	0.009	0.012	0.459	32.583	7.05E-05	NA
rs11631408	15	51746236	A	G	0.463	0.014	0.002	2.44E-11	0.025	0.009	0.008	44.582	9.65E-05	NA
rs11636565	15	36391846	C	A	0.488	-0.014	0.002	6.84E-12	-0.010	0.009	0.274	47.072	1.02E-04	NA
rs11660335	18	22154235	T	C	0.161	0.018	0.003	1.07E-09	-0.005	0.012	0.679	37.189	8.05E-05	NA
rs11676272	2	25141538	A	G	0.464	-0.020	0.002	1.05E-17	-0.022	0.009	0.017	73.421	1.59E-04	NA
rs11747810	5	153100273	T	C	0.495	0.013	0.002	1.83E-09	0.019	0.009	0.043	36.148	7.83E-05	NA
rs11866815	16	387867	C	T	0.242	0.016	0.002	2.32E-10	0.027	0.012	0.018	40.181	8.70E-05	NA
rs11880870	19	18830704	A	G	0.499	0.013	0.002	2.98E-09	0.021	0.010	0.031	35.198	7.62E-05	NA
rs11981190	7	71659538	G	A	0.341	0.013	0.002	5.84E-09	0.018	0.010	0.077	33.887	7.34E-05	NA
rs1200349	15	41821752	T	C	0.482	-0.013	0.002	1.36E-09	-0.015	0.009	0.098	36.724	7.95E-05	NA
rs12201987	6	143182396	C	T	0.205	0.016	0.003	1.87E-09	0.011	0.012	0.363	36.103	7.82E-05	NA
rs12446554	16	19935073	G	T	0.130	0.020	0.003	3.79E-10	-0.009	0.014	0.537	39.220	8.49E-05	NA
rs12520822	5	105699867	C	T	0.408	0.012	0.002	1.84E-08	0.005	0.009	0.618	31.658	6.85E-05	NA
rs12608504	19	18389135	A	G	0.359	0.016	0.002	2.27E-13	-0.011	0.011	0.311	53.757	1.16E-04	NA
rs12624640	20	32952125	G	A	0.430	-0.012	0.002	7.17E-09	-0.006	0.009	0.546	33.489	7.25E-05	NA
rs12967135	18	57849023	G	A	0.240	-0.046	0.003	3.09E-66	-0.009	0.011	0.405	295.539	6.39E-04	Worrier or anxious feelings
rs13024601	2	100902032	C	T	0.346	0.014	0.002	1.39E-09	0.007	0.010	0.481	36.680	7.94E-05	NA
rs13107325	4	103188709	C	T	0.080	-0.023	0.004	1.94E-09	-0.005	0.024	0.844	36.033	7.80E-05	Intelligence
rs13206549	6	162978032	T	G	0.134	0.019	0.003	1.54E-09	0.020	0.014	0.141	36.486	7.90E-05	NA
rs13244268	7	72911843	T	C	0.114	0.024	0.003	9.17E-18	0.023	0.014	0.108	73.684	1.59E-04	NA
rs13250456	8	72408720	C	T	0.055	-0.025	0.005	4.67E-08	-0.050	0.019	0.009	29.850	6.46E-05	NA
rs13275593	8	68257300	G	A	0.285	-0.015	0.002	2.14E-10	-0.012	0.010	0.244	40.332	8.73E-05	NA
rs1330304	9	16714629	C	G	0.267	0.013	0.002	2.32E-08	0.002	0.011	0.863	31.202	6.75E-05	NA
rs13416004	2	69664976	C	T	0.415	0.012	0.002	3.16E-08	-0.004	0.009	0.639	30.605	6.63E-05	NA
rs13416667	2	56608267	C	A	0.274	-0.015	0.002	2.32E-10	0.010	0.013	0.450	40.179	8.70E-05	NA
rs13435860	4	136975545	A	T	0.368	-0.013	0.002	1.94E-09	-0.004	0.010	0.692	36.037	7.80E-05	NA
rs1353580	15	47738732	T	A	0.374	-0.012	0.002	1.70E-08	0.004	0.010	0.692	31.810	6.89E-05	NA
rs1362910	8	30856464	A	G	0.386	0.014	0.002	9.70E-11	0.019	0.010	0.042	41.882	9.07E-05	NA
rs1363695	5	130378027	C	T	0.228	0.015	0.003	6.49E-09	-0.008	0.011	0.449	33.681	7.29E-05	NA
rs1412239	9	28425515	C	G	0.304	-0.022	0.002	2.39E-21	-0.007	0.010	0.492	89.993	1.95E-04	NA

rs1421085	16	53800954	T	C	0.432	-0.062	0.002	5.07E-152	-0.020	0.009	0.032	689.747	1.49E-03	NA
rs1452075	3	62481063	C	T	0.284	-0.014	0.002	9.96E-09	-0.008	0.010	0.417	32.850	7.11E-05	NA
rs1456116	3	123084379	G	A	0.417	-0.013	0.002	1.99E-09	0.010	0.009	0.285	35.987	7.79E-05	NA
rs1460940	1	72814617	G	A	0.182	-0.020	0.003	2.89E-13	-0.032	0.012	0.008	53.284	1.15E-04	NA
rs1507153	6	79484386	C	A	0.364	-0.013	0.002	3.86E-09	-0.024	0.010	0.012	34.694	7.51E-05	NA
rs1513475	3	35724256	T	C	0.343	0.015	0.002	8.46E-11	0.001	0.010	0.938	42.149	9.12E-05	NA
rs1534696	7	26397239	C	A	0.439	0.014	0.002	1.24E-11	0.000	0.010	0.996	45.905	9.94E-05	NA
rs17024393	1	110154688	T	C	0.033	-0.048	0.006	1.96E-15	-0.036	0.026	0.169	63.106	1.37E-04	NA
rs17115145	14	30122409	C	T	0.406	-0.012	0.002	1.29E-08	-0.020	0.009	0.033	32.350	7.00E-05	NA
rs1730858	1	107619244	T	C	0.328	0.015	0.002	1.04E-10	0.006	0.010	0.549	41.736	9.03E-05	NA
rs17391694	1	78623626	C	T	0.096	-0.026	0.004	2.39E-12	-0.023	0.014	0.089	49.132	1.06E-04	NA
rs17513613	19	30286822	T	C	0.289	-0.017	0.002	1.94E-13	0.001	0.010	0.948	54.063	1.17E-04	NA
rs17618704	17	46221010	T	G	0.068	0.025	0.004	1.52E-09	0.039	0.018	0.028	36.510	7.90E-05	NA
rs17639546	3	61251635	G	A	0.148	0.018	0.003	3.65E-09	0.011	0.012	0.361	34.803	7.53E-05	NA
rs17694506	2	171632225	C	T	0.388	0.014	0.002	3.33E-11	-0.008	0.010	0.424	43.971	9.52E-05	NA
rs17724992	19	18454825	A	G	0.245	0.014	0.002	8.13E-09	0.026	0.011	0.015	33.243	7.20E-05	NA
rs17783751	16	89513984	C	A	0.176	-0.015	0.003	4.79E-08	0.009	0.012	0.442	29.800	6.45E-05	NA
rs17821316	15	58703069	A	C	0.488	-0.012	0.002	9.97E-10	-0.010	0.010	0.335	37.331	8.08E-05	NA
rs1784460	11	118938371	T	A	0.360	-0.015	0.002	8.15E-12	0.006	0.010	0.541	46.729	1.01E-04	NA
rs1876359	16	4930100	C	T	0.377	-0.013	0.002	5.40E-09	-0.007	0.010	0.492	34.038	7.37E-05	NA
rs192812	3	161491831	C	T	0.454	-0.012	0.002	4.87E-09	-0.014	0.009	0.131	34.241	7.41E-05	NA
rs1945941	11	132620118	G	A	0.376	0.012	0.002	3.63E-08	0.022	0.009	0.017	30.337	6.57E-05	NA
rs2002023	10	76848524	C	T	0.441	-0.012	0.002	8.92E-09	-0.007	0.009	0.431	33.064	7.16E-05	NA
rs2015407	14	103274547	A	G	0.345	0.014	0.002	4.68E-10	0.024	0.010	0.015	38.808	8.40E-05	NA
rs2035831	3	173683791	G	C	0.321	0.013	0.002	1.48E-08	0.005	0.010	0.628	32.085	6.95E-05	NA
rs2060604	8	76650334	T	C	0.414	0.015	0.002	2.80E-12	0.012	0.009	0.220	48.822	1.06E-04	NA
rs2065418	11	30422068	T	G	0.354	0.016	0.002	1.39E-12	-0.002	0.010	0.825	50.196	1.09E-04	NA
rs2066295	6	26168903	A	G	0.219	0.023	0.003	5.73E-18	0.010	0.011	0.342	74.611	1.61E-04	NA
rs2143253	20	41987392	G	A	0.137	0.019	0.003	1.29E-09	0.037	0.015	0.013	36.823	7.97E-05	NA
rs2180454	14	29690513	T	C	0.241	-0.015	0.003	1.90E-09	-0.006	0.011	0.582	36.071	7.81E-05	NA
rs2237717	7	116405387	T	C	0.443	-0.011	0.002	2.63E-08	0.005	0.010	0.601	30.960	6.70E-05	NA
rs2242449	17	7095507	C	T	0.417	-0.013	0.002	2.85E-09	-0.002	0.010	0.861	35.287	7.64E-05	NA
rs227199	1	210265384	G	C	0.316	0.013	0.002	5.06E-09	0.017	0.010	0.079	34.168	7.40E-05	NA
rs2272168	7	99102777	G	C	0.163	0.017	0.003	3.68E-09	-0.011	0.013	0.401	34.785	7.53E-05	NA
rs2275543	9	107651174	T	C	0.099	-0.019	0.003	6.58E-09	-0.026	0.016	0.102	33.655	7.29E-05	NA
rs2298969	4	3186244	A	G	0.490	0.012	0.002	2.79E-08	0.004	0.009	0.705	30.851	6.68E-05	NA
rs2299383	7	103418846	C	T	0.410	-0.014	0.002	2.20E-10	-0.004	0.009	0.688	40.282	8.72E-05	NA
rs2307111	5	75003678	T	C	0.396	0.025	0.002	4.41E-29	0.006	0.010	0.537	125.285	2.71E-04	NA
rs2436726	6	40366427	C	T	0.405	-0.016	0.002	2.24E-12	-0.003	0.010	0.739	49.262	1.07E-04	NA
rs2531995	16	4013467	C	T	0.387	-0.019	0.002	2.80E-17	0.001	0.010	0.954	71.477	1.55E-04	NA
rs2596933	3	9365746	G	A	0.267	0.014	0.002	1.26E-08	-0.012	0.011	0.263	32.396	7.01E-05	NA
rs261336	15	58742418	G	A	0.161	-0.020	0.003	2.07E-14	0.013	0.013	0.298	58.461	1.27E-04	NA
rs2781668	6	131897278	C	T	0.170	-0.015	0.003	3.64E-08	-0.019	0.012	0.136	30.331	6.57E-05	NA
rs2787693	1	49915848	C	A	0.302	0.017	0.002	1.94E-11	0.011	0.010	0.287	45.027	9.75E-05	NA
rs2814944	6	34552797	G	A	0.137	-0.034	0.003	1.03E-26	-0.027	0.013	0.031	114.463	2.48E-04	NA
rs2820232	6	35003603	G	T	0.459	-0.012	0.002	2.95E-08	-0.009	0.009	0.316	30.741	6.65E-05	NA
rs2836753	21	40291187	T	C	0.385	-0.013	0.002	7.29E-10	-0.010	0.010	0.301	37.941	8.21E-05	NA
rs2861688	2	67838474	T	C	0.273	0.016	0.002	8.97E-11	-0.005	0.011	0.671	42.033	9.10E-05	NA
rs2925979	16	81534790	T	C	0.288	0.014	0.002	1.10E-10	0.004	0.010	0.689	41.628	9.01E-05	NA
rs2968856	7	150614934	T	C	0.261	0.016	0.002	1.80E-11	0.020	0.011	0.069	45.179	9.78E-05	NA
rs2990997	1	190119703	T	A	0.353	-0.012	0.002	3.24E-08	0.014	0.010	0.177	30.558	6.62E-05	NA

rs320	8	19819077	T	G	0.287	0.031	0.002	2.30E-43	0.010	0.010	0.321	190.642	4.13E-04	NA
rs323781	5	86752616	A	C	0.034	-0.032	0.006	3.82E-08	0.011	0.029	0.702	30.237	6.55E-05	NA
rs349088	11	84814393	C	A	0.480	0.013	0.002	3.23E-09	0.009	0.009	0.320	35.040	7.59E-05	NA
rs350832	19	4069426	G	A	0.227	-0.014	0.003	1.88E-08	-0.028	0.013	0.034	31.615	6.84E-05	NA
rs3749262	3	131624097	G	A	0.263	-0.019	0.002	1.12E-14	-0.024	0.010	0.023	59.681	1.29E-04	NA
rs3808436	8	116560967	A	G	0.432	0.016	0.002	1.58E-13	-0.001	0.009	0.940	54.468	1.18E-04	NA
rs3814424	5	87968953	C	T	0.172	-0.025	0.003	2.55E-16	0.010	0.013	0.470	67.124	1.45E-04	Intelligence
rs3852012	3	44045981	G	A	0.287	0.013	0.002	1.57E-08	-0.001	0.010	0.948	31.963	6.92E-05	NA
rs3902840	9	129419025	G	A	0.092	-0.021	0.004	1.72E-08	-0.005	0.016	0.767	31.786	6.88E-05	NA
rs4074448	15	63888495	G	A	0.434	0.012	0.002	2.37E-08	-0.001	0.010	0.948	31.161	6.75E-05	NA
rs4283409	2	228977142	A	G	0.387	-0.012	0.002	7.64E-09	-0.014	0.009	0.113	33.365	7.22E-05	NA
rs429343	2	147903382	A	G	0.437	0.012	0.002	1.26E-08	0.009	0.009	0.339	32.394	7.01E-05	NA
rs4377779	6	12117344	T	C	0.334	0.013	0.002	5.27E-09	0.007	0.010	0.464	34.086	7.38E-05	NA
rs4410790	7	17284577	T	C	0.385	-0.012	0.002	5.72E-09	0.004	0.010	0.699	33.927	7.34E-05	NA
rs442177	4	88030261	G	T	0.421	-0.011	0.002	3.89E-08	-0.001	0.009	0.888	30.204	6.54E-05	NA
rs4477562	13	54104968	C	T	0.110	-0.020	0.003	9.80E-09	0.004	0.014	0.781	32.880	7.12E-05	NA
rs4482463	2	205375909	C	A	0.081	0.029	0.004	4.75E-12	-0.004	0.017	0.811	47.787	1.03E-04	NA
rs4660208	1	39722214	A	G	0.195	-0.023	0.003	2.84E-18	-0.006	0.011	0.612	75.995	1.64E-04	NA
rs4665991	2	27766284	G	A	0.264	-0.013	0.002	2.16E-09	0.008	0.012	0.496	35.821	7.75E-05	NA
rs4693325	4	94408529	A	C	0.199	-0.016	0.003	1.74E-09	-0.021	0.012	0.080	36.241	7.85E-05	NA
rs4755720	11	43628749	C	T	0.400	0.016	0.002	4.50E-14	0.004	0.010	0.676	56.935	1.23E-04	NA
rs4777542	15	73082366	C	T	0.300	0.014	0.002	3.14E-09	0.008	0.010	0.421	35.098	7.60E-05	NA
rs4784745	16	57014875	A	G	0.329	-0.019	0.002	4.00E-21	0.002	0.010	0.867	88.973	1.93E-04	NA
rs4790292	17	1824305	C	A	0.144	0.020	0.003	2.66E-10	0.010	0.013	0.445	39.913	8.64E-05	NA
rs479315	11	66064991	G	C	0.170	-0.016	0.003	2.08E-08	0.001	0.012	0.948	31.420	6.80E-05	NA
rs4805758	19	32866424	G	A	0.162	0.020	0.003	5.55E-13	-0.013	0.013	0.303	51.999	1.13E-04	Fed-up feelings
rs4810479	20	44545048	C	T	0.288	0.013	0.002	6.17E-10	0.008	0.011	0.455	38.267	8.28E-05	NA
rs4889782	17	78640510	C	T	0.415	0.013	0.002	1.61E-09	0.003	0.010	0.778	36.391	7.88E-05	NA
rs4890439	18	40732586	G	A	0.375	0.012	0.002	4.60E-08	0.015	0.010	0.118	29.879	6.47E-05	NA
rs4929923	11	8639200	T	C	0.377	-0.015	0.002	4.28E-11	-0.007	0.010	0.498	43.484	9.41E-05	NA
rs4947406	7	50661735	T	G	0.350	0.016	0.002	9.35E-12	0.001	0.010	0.899	46.461	1.01E-04	NA
rs4971632	2	50240091	T	C	0.166	0.017	0.003	1.26E-09	0.016	0.012	0.194	36.870	7.98E-05	NA
rs512005	1	33377679	T	C	0.236	0.014	0.003	4.27E-08	0.015	0.011	0.192	30.024	6.50E-05	NA
rs563296	10	99772404	G	A	0.441	-0.013	0.002	1.23E-08	-0.019	0.010	0.043	32.438	7.02E-05	NA
rs6115015	20	25027692	G	A	0.333	-0.013	0.002	6.30E-09	0.004	0.010	0.716	33.741	7.30E-05	NA
rs619054	11	116660813	G	A	0.232	0.015	0.002	2.70E-11	-0.007	0.011	0.533	44.384	9.61E-05	NA
rs649458	1	23712528	T	A	0.137	0.018	0.003	5.66E-09	0.006	0.013	0.660	33.949	7.35E-05	NA
rs6499240	16	69686912	A	G	0.422	-0.020	0.002	1.52E-18	0.004	0.009	0.702	77.230	1.67E-04	NA
rs6499861	16	56991495	C	G	0.162	-0.018	0.003	8.61E-12	0.007	0.013	0.605	46.623	1.01E-04	NA
rs6545714	2	59307725	G	A	0.382	0.019	0.002	3.15E-18	0.021	0.010	0.027	75.796	1.64E-04	NA
rs6548834	3	82704753	G	A	0.378	-0.013	0.002	1.08E-08	0.010	0.010	0.302	32.694	7.08E-05	NA
rs6550597	3	18738940	G	A	0.298	-0.013	0.002	4.61E-09	-0.013	0.010	0.212	34.346	7.43E-05	NA
rs6575340	14	94023972	G	A	0.377	-0.014	0.002	8.82E-10	-0.015	0.010	0.119	37.571	8.13E-05	NA
rs6577584	1	6715390	T	G	0.372	-0.014	0.002	1.65E-10	0.005	0.010	0.640	40.843	8.84E-05	NA
rs6602151	10	16764483	T	C	0.294	-0.015	0.002	5.89E-10	0.000	0.010	0.997	38.356	8.30E-05	NA
rs6603004	15	84568504	A	G	0.466	-0.013	0.002	1.81E-09	-0.022	0.009	0.017	36.163	7.83E-05	NA
rs6673977	1	7736097	A	G	0.354	-0.012	0.002	1.63E-08	-0.009	0.010	0.337	31.892	6.90E-05	NA
rs6711375	2	161090873	G	A	0.320	-0.014	0.002	1.11E-09	-0.027	0.010	0.008	37.115	8.03E-05	NA
rs6754311	2	136707982	T	C	0.489	0.013	0.002	3.95E-10	0.026	0.015	0.084	39.137	8.47E-05	NA
rs676387	17	40706273	C	A	0.266	-0.013	0.002	3.42E-08	-0.003	0.010	0.762	30.452	6.59E-05	NA
rs687672	11	65649984	T	C	0.189	0.021	0.003	5.92E-14	0.023	0.012	0.051	56.399	1.22E-04	NA

rs6907923	6	51802669	G	A	0.341	-0.015	0.002	1.71E-10	-0.005	0.010	0.596	40.774	8.83E-05	NA
rs7016529	8	19806631	T	C	0.011	-0.102	0.009	1.94E-27	0.025	0.040	0.531	117.778	2.55E-04	NA
rs703965	10	80955067	C	T	0.456	-0.012	0.002	8.25E-09	-0.006	0.010	0.559	33.214	7.19E-05	NA
rs705986	8	64737682	A	G	0.491	0.012	0.002	2.53E-08	-0.009	0.009	0.316	31.039	6.72E-05	NA
rs7075195	10	65050659	A	G	0.435	0.017	0.002	5.31E-16	0.014	0.009	0.142	65.676	1.42E-04	Educational attainment
rs7084454	10	21821274	G	A	0.338	-0.016	0.002	9.04E-13	-0.016	0.010	0.109	51.042	1.10E-04	NA
rs7132908	12	50263148	G	A	0.357	-0.019	0.002	9.07E-15	0.009	0.010	0.352	60.088	1.30E-04	NA
rs7144011	14	79940383	G	T	0.207	-0.023	0.003	1.71E-17	-0.014	0.011	0.208	72.451	1.57E-04	NA
rs7186893	16	24806420	G	T	0.267	0.020	0.002	3.04E-16	0.029	0.011	0.005	66.779	1.45E-04	Mood swings
rs7198357	16	67884619	C	T	0.178	0.017	0.003	4.09E-11	-0.012	0.012	0.322	43.573	9.43E-05	NA
rs7206608	16	82872628	C	G	0.313	-0.013	0.002	1.26E-08	0.001	0.010	0.886	32.386	7.01E-05	NA
rs7241918	18	47160953	G	T	0.163	0.018	0.003	7.52E-13	-0.001	0.012	0.912	51.403	1.11E-04	NA
rs7323827	13	94116675	A	G	0.304	0.013	0.002	4.59E-09	0.000	0.010	0.968	34.358	7.44E-05	NA
rs7488867	12	103699685	C	T	0.281	0.015	0.003	1.57E-09	-0.007	0.011	0.541	36.447	7.89E-05	NA
rs7515635	1	42408070	T	C	0.471	0.012	0.002	4.68E-09	0.010	0.009	0.278	34.317	7.43E-05	NA
rs7537581	1	2725475	C	A	0.476	-0.012	0.002	4.55E-08	0.009	0.010	0.361	29.899	6.47E-05	NA
rs7549987	1	80789011	C	T	0.299	-0.014	0.002	4.02E-09	-0.013	0.010	0.219	34.614	7.49E-05	NA
rs7581217	2	25524944	T	C	0.370	0.013	0.002	5.93E-09	0.023	0.010	0.029	33.857	7.33E-05	NA
rs7638388	3	47914974	C	T	0.088	-0.024	0.004	1.01E-10	-0.017	0.016	0.278	41.794	9.05E-05	NA
rs7642876	3	15883094	A	G	0.404	0.012	0.002	1.35E-08	0.021	0.009	0.025	32.265	6.98E-05	NA
rs7668263	4	143781835	T	C	0.382	-0.012	0.002	4.21E-08	-0.002	0.010	0.869	30.049	6.50E-05	NA
rs779655	5	170495158	G	C	0.272	-0.014	0.002	1.21E-08	-0.019	0.010	0.066	32.464	7.03E-05	NA
rs7824519	8	14222796	T	G	0.324	-0.013	0.002	1.51E-08	0.008	0.010	0.399	32.035	6.93E-05	NA
rs7928842	11	47566352	T	C	0.422	-0.020	0.002	2.30E-20	-0.009	0.009	0.326	85.511	1.85E-04	NA
rs7947951	11	13356030	A	G	0.297	-0.014	0.002	1.59E-10	-0.007	0.010	0.452	40.911	8.86E-05	NA
rs7982447	13	54453811	T	C	0.200	-0.019	0.003	6.75E-13	-0.003	0.011	0.753	51.616	1.12E-04	NA
rs8015400	14	25930988	C	A	0.348	-0.018	0.002	8.13E-15	-0.005	0.010	0.630	60.303	1.31E-04	NA
rs8078079	17	34959346	T	C	0.395	0.015	0.002	2.89E-11	0.018	0.010	0.052	44.247	9.58E-05	NA
rs818524	1	85201228	T	C	0.289	-0.013	0.002	2.84E-08	0.004	0.011	0.709	30.812	6.67E-05	NA
rs8192675	3	170724883	T	C	0.289	-0.013	0.002	4.30E-08	-0.003	0.010	0.802	30.007	6.50E-05	NA
rs938875	15	67952922	A	G	0.408	-0.019	0.002	3.72E-18	-0.026	0.009	0.006	75.463	1.63E-04	NA
rs9556979	13	99241507	T	G	0.324	-0.013	0.002	4.53E-09	0.000	0.010	0.974	34.382	7.44E-05	NA
rs9563569	13	58571207	G	A	0.150	0.018	0.003	2.09E-08	0.028	0.012	0.020	31.408	6.80E-05	NA
rs9630985	2	181607676	A	C	0.349	-0.014	0.002	9.30E-11	-0.024	0.010	0.016	41.963	9.08E-05	NA
rs9686661	5	55861786	C	T	0.186	-0.023	0.003	4.65E-18	-0.010	0.012	0.414	75.025	1.62E-04	NA
rs9816226	3	185834499	A	T	0.188	-0.016	0.003	8.56E-09	0.002	0.012	0.847	33.144	7.17E-05	NA
rs9872156	3	176337348	G	A	0.260	-0.013	0.002	4.04E-08	-0.014	0.010	0.168	30.129	6.52E-05	NA
rs987237	6	50803050	A	G	0.181	-0.030	0.003	7.81E-26	-0.009	0.012	0.463	110.449	2.39E-04	NA
rs9947450	18	58052665	C	T	0.030	0.062	0.007	1.22E-21	0.035	0.023	0.125	91.321	1.98E-04	NA
rs9987289	8	9183358	A	G	0.075	0.022	0.004	1.84E-09	-0.001	0.016	0.945	36.136	7.82E-05	NA

Genomic positions reported in the GWAS of Mets refer to human reference assembly (GRCh37/hg19).

Abbreviations:MetS, Metabolic syndrome; ADHD, Attention-deficit/hyperactivity disorder; SNPs, Single nucleotide polymorphisms; CHR, Chromosome; EA, Effect allele; OA, Other allele; EAF, Effect allele frequency; SE, Standard error; PVE, Proportion of phenotypic variance explained.

SNPs that are unavailable in the outcome GWAS: rs6754311,rs2516740,rs12596491,rs1914888,rs12981256,rs2074881,among them, rs6754311,rs2074881 were substitute by proxy SNPs: rs11693502,rs8100279, respectively.

Palindromic and not-inferrable SNPs that are abandoned as instruments: rs1937433, rs2253310, rs2396625, rs6916318.

5 SNPs detected by PRESSO outlier test, and 48 SNPs were detected by MR-Radial outlier test.

Supplementary Table 3. Genetic instruments for the causal relationship of Mets and ALC.

SNP	CHR	Position	EA	OA	EAF	Beta for exposure	SE for exposure	P value for exposure	Beta for outcome	SE for outcome	P value for outcome	F statistics	PVE	Association with potential confounding factors
rs10116353	9	111950088	G	T	0.307	0.013	0.002	9.39E-09	0.045	0.024	0.062	32.964	7.14E-05	NA
rs10160660	11	76474537	T	C	0.248	0.013	0.002	4.83E-08	0.024	0.028	0.379	29.786	6.45E-05	NA
rs102275	11	61557803	T	C	0.363	-0.013	0.002	6.28E-11	0.006	0.023	0.793	42.731	9.25E-05	NA
rs1032524	1	201790826	T	C	0.484	-0.015	0.002	6.01E-12	0.011	0.022	0.640	47.325	1.02E-04	NA
rs1042034	2	21225281	C	T	0.218	-0.020	0.002	7.07E-21	0.007	0.027	0.793	87.848	1.90E-04	NA
rs10423928	19	46182304	T	A	0.214	0.020	0.003	8.49E-13	-0.008	0.031	0.797	51.166	1.11E-04	NA
rs10734924	12	123018894	T	G	0.259	-0.015	0.003	2.19E-09	-0.004	0.025	0.884	35.801	7.75E-05	NA
rs10768994	11	43936945	T	C	0.430	0.012	0.002	4.52E-08	-0.013	0.022	0.573	29.912	6.48E-05	NA
rs10779835	1	230299949	T	C	0.391	0.015	0.002	2.45E-14	0.002	0.023	0.932	58.131	1.26E-04	NA
rs10783779	12	56491880	T	G	0.403	0.013	0.002	3.84E-09	-0.003	0.024	0.894	34.704	7.51E-05	NA
rs10872224	6	98435125	G	T	0.409	-0.014	0.002	9.76E-11	-0.010	0.023	0.665	41.868	9.06E-05	Educational attainment; Intelligence
rs10887579	10	88097210	A	C	0.354	-0.013	0.002	4.09E-09	0.024	0.025	0.333	34.579	7.49E-05	NA
rs10930502	2	172890588	A	G	0.304	0.013	0.002	1.18E-08	-0.010	0.024	0.685	32.519	7.04E-05	NA
rs10938397	4	45182527	A	G	0.420	-0.023	0.002	8.14E-26	-0.026	0.023	0.261	110.367	2.39E-04	NA
rs11072938	15	81021661	C	T	0.241	0.016	0.002	4.30E-11	0.000	0.025	1.000	43.470	9.41E-05	NA
rs11079849	17	47090785	C	T	0.290	0.015	0.002	6.90E-11	-0.012	0.029	0.679	42.547	9.21E-05	NA
rs11150604	16	31037020	A	T	0.389	0.024	0.002	3.91E-28	-0.024	0.023	0.293	120.954	2.62E-04	NA
rs11165643	1	96924097	C	T	0.417	-0.013	0.002	6.90E-10	-0.019	0.023	0.398	38.049	8.24E-05	NA
rs11187845	10	96060198	C	A	0.096	0.020	0.004	8.08E-09	-0.021	0.038	0.587	33.256	7.20E-05	NA
rs1138429	16	56942921	A	T	0.101	-0.028	0.003	1.42E-18	0.043	0.040	0.283	77.365	1.67E-04	NA
rs1159974	6	126090277	T	C	0.499	-0.013	0.002	3.30E-10	-0.008	0.022	0.714	39.489	8.55E-05	NA
rs11611246	12	939480	G	T	0.183	-0.016	0.003	1.14E-08	0.043	0.028	0.133	32.583	7.05E-05	NA
rs11631408	15	51746236	A	G	0.463	0.014	0.002	2.44E-11	0.006	0.022	0.781	44.582	9.65E-05	NA
rs11636565	15	36391846	C	A	0.488	-0.014	0.002	6.84E-12	-0.016	0.022	0.470	47.072	1.02E-04	NA
rs11660335	18	22154235	T	C	0.161	0.018	0.003	1.07E-09	-0.034	0.028	0.228	37.189	8.05E-05	NA
rs11676272	2	25141538	A	G	0.464	-0.020	0.002	1.05E-17	0.019	0.022	0.398	73.421	1.59E-04	NA
rs11826999	11	116553778	G	A	0.223	-0.023	0.002	6.07E-26	-0.026	0.026	0.318	110.950	2.40E-04	NA
rs11866815	16	387867	C	T	0.242	0.016	0.002	2.32E-10	-0.031	0.026	0.227	40.181	8.70E-05	NA
rs11880870	19	18830704	A	G	0.499	0.013	0.002	2.98E-09	0.034	0.028	0.226	35.198	7.62E-05	NA
rs11981190	7	71659538	G	A	0.341	0.013	0.002	5.84E-09	-0.017	0.023	0.474	33.887	7.34E-05	NA
rs1200349	15	41821752	T	C	0.482	-0.013	0.002	1.36E-09	-0.018	0.022	0.415	36.724	7.95E-05	NA
rs12033257	1	112318484	A	G	0.382	0.014	0.002	8.97E-10	0.034	0.028	0.225	37.538	8.13E-05	NA
rs12201987	6	143182396	C	T	0.205	0.016	0.003	1.87E-09	0.041	0.028	0.146	36.103	7.82E-05	NA
rs12446554	16	19935073	G	T	0.130	0.020	0.003	3.79E-10	-0.028	0.031	0.380	39.220	8.49E-05	NA
rs12513212	4	96149346	A	C	0.387	-0.014	0.002	2.85E-10	-0.005	0.023	0.834	39.778	8.61E-05	NA
rs12520822	5	105699867	C	T	0.408	0.012	0.002	1.84E-08	0.008	0.022	0.702	31.658	6.85E-05	NA
rs12596491	16	88928417	C	T	0.340	0.012	0.002	4.58E-08	-0.049	0.026	0.058	29.888	6.47E-05	NA
rs12608504	19	18389135	A	G	0.359	0.016	0.002	2.27E-13	-0.002	0.023	0.945	53.757	1.16E-04	NA
rs12624640	20	32952125	G	A	0.430	-0.012	0.002	7.17E-09	-0.030	0.023	0.180	33.489	7.25E-05	NA
rs12824567	12	124495203	G	C	0.350	0.013	0.002	1.94E-09	-0.036	0.024	0.127	36.032	7.80E-05	NA
rs12967135	18	57849023	G	A	0.240	-0.046	0.003	3.09E-66	0.025	0.026	0.343	295.539	6.39E-04	Worrier or anxious feelings
rs12981256	19	1865901	G	A	0.475	-0.016	0.002	7.75E-14	0.005	0.027	0.844	55.867	1.21E-04	NA
rs13024601	2	100902032	C	T	0.346	0.014	0.002	1.39E-09	-0.008	0.023	0.735	36.680	7.94E-05	NA
rs13029936	2	208465544	C	T	0.176	0.017	0.003	1.31E-09	-0.051	0.028	0.063	36.795	7.96E-05	NA
rs13107325	4	103188709	C	T	0.080	-0.023	0.004	1.94E-09	0.040	0.049	0.415	36.033	7.80E-05	Intelligence
rs13206549	6	162978032	T	G	0.134	0.019	0.003	1.54E-09	-0.038	0.033	0.258	36.486	7.90E-05	NA
rs13244268	7	72911843	T	C	0.114	0.024	0.003	9.17E-18	-0.028	0.034	0.407	73.684	1.59E-04	NA
rs13250456	8	72408720	C	T	0.055	-0.025	0.005	4.67E-08	-0.001	0.055	0.992	29.850	6.46E-05	NA
rs13275593	8	68257300	G	A	0.285	-0.015	0.002	2.14E-10	-0.022	0.028	0.419	40.332	8.73E-05	NA

rs1330304	9	16714629	C	G	0.267	0.013	0.002	2.32E-08	0.033	0.026	0.214	31.202	6.75E-05	NA
rs13322435	3	156795468	A	G	0.407	0.020	0.002	6.60E-20	-0.009	0.030	0.776	83.432	1.81E-04	NA
rs13416004	2	69664976	C	T	0.415	0.012	0.002	3.16E-08	-0.020	0.022	0.369	30.605	6.63E-05	NA
rs13416667	2	56608267	C	A	0.274	-0.015	0.002	2.32E-10	-0.037	0.033	0.262	40.179	8.70E-05	NA
rs13435860	4	136975545	A	T	0.368	-0.013	0.002	1.94E-09	0.000	0.024	0.993	36.037	7.80E-05	NA
rs1353580	15	47738732	T	A	0.374	-0.012	0.002	1.70E-08	-0.002	0.023	0.921	31.810	6.89E-05	NA
rs1362910	8	30856464	A	G	0.386	0.014	0.002	9.70E-11	0.014	0.022	0.535	41.882	9.07E-05	NA
rs1363695	5	130378027	C	T	0.228	0.015	0.003	6.49E-09	0.003	0.029	0.907	33.681	7.29E-05	NA
rs1412239	9	28425515	C	G	0.304	-0.022	0.002	2.39E-21	0.006	0.024	0.806	89.993	1.95E-04	NA
rs1421085	16	53800954	T	C	0.432	-0.062	0.002	5.07E-152	0.002	0.023	0.938	689.747	1.49E-03	NA
rs1431659	8	73439070	A	G	0.278	0.019	0.002	4.48E-15	0.011	0.025	0.667	61.476	1.33E-04	NA
rs1448613	3	85875265	G	A	0.386	0.014	0.002	2.40E-10	0.009	0.023	0.708	40.110	8.68E-05	NA
rs1452075	3	62481063	C	T	0.284	-0.014	0.002	9.96E-09	-0.030	0.025	0.224	32.850	7.11E-05	NA
rs1456116	3	123084379	G	A	0.417	-0.013	0.002	1.99E-09	0.023	0.022	0.292	35.987	7.79E-05	NA
rs1458156	12	41887940	C	T	0.449	-0.013	0.002	4.45E-09	-0.008	0.022	0.706	34.416	7.45E-05	NA
rs1460940	1	72814617	G	A	0.182	-0.020	0.003	2.89E-13	-0.019	0.028	0.517	53.284	1.15E-04	NA
rs1513475	3	35724256	T	C	0.343	0.015	0.002	8.46E-11	0.025	0.023	0.286	42.149	9.12E-05	NA
rs1534696	7	26397239	C	A	0.439	0.014	0.002	1.24E-11	0.008	0.029	0.792	45.905	9.94E-05	NA
rs1580099	3	94003603	C	A	0.406	0.014	0.002	4.35E-09	-0.015	0.022	0.490	34.459	7.46E-05	NA
rs17024393	1	110154688	T	C	0.033	-0.048	0.006	1.96E-15	-0.061	0.066	0.350	63.106	1.37E-04	NA
rs17115145	14	30122409	C	T	0.406	-0.012	0.002	1.29E-08	-0.012	0.024	0.624	32.350	7.00E-05	NA
rs17162333	1	27236757	C	G	0.132	-0.020	0.003	6.24E-11	-0.011	0.031	0.730	42.744	9.25E-05	NA
rs17207196	7	75101065	C	T	0.444	0.018	0.002	2.68E-16	0.050	0.026	0.051	67.024	1.45E-04	NA
rs1730858	1	107619244	T	C	0.328	0.015	0.002	1.04E-10	-0.037	0.024	0.118	41.736	9.03E-05	NA
rs17391694	1	78623626	C	T	0.096	-0.026	0.004	2.39E-12	-0.003	0.038	0.930	49.132	1.06E-04	NA
rs17513613	19	30286822	T	C	0.289	-0.017	0.002	1.94E-13	0.000	0.024	0.992	54.063	1.17E-04	NA
rs17618704	17	46221010	T	G	0.068	0.025	0.004	1.52E-09	0.032	0.043	0.459	36.510	7.90E-05	NA
rs17639546	3	61251635	G	A	0.148	0.018	0.003	3.65E-09	-0.041	0.031	0.184	34.803	7.53E-05	NA
rs17694506	2	171632225	C	T	0.388	0.014	0.002	3.33E-11	-0.025	0.024	0.294	43.971	9.52E-05	NA
rs17724992	19	18454825	A	G	0.245	0.014	0.002	8.13E-09	0.004	0.029	0.886	33.243	7.20E-05	NA
rs17783751	16	89513984	C	A	0.176	-0.015	0.003	4.79E-08	0.022	0.028	0.420	29.800	6.45E-05	NA
rs17806379	20	51107290	C	T	0.185	0.020	0.003	3.64E-13	-0.001	0.029	0.984	52.829	1.14E-04	NA
rs17821316	15	58703069	A	C	0.488	-0.012	0.002	9.97E-10	0.015	0.037	0.681	37.331	8.08E-05	NA
rs1784460	11	118938371	T	A	0.360	-0.015	0.002	8.15E-12	0.023	0.026	0.366	46.729	1.01E-04	NA
rs1876359	16	4930100	C	T	0.377	-0.013	0.002	5.40E-09	0.004	0.023	0.856	34.038	7.37E-05	NA
rs1886558	13	111982615	A	G	0.389	0.012	0.002	4.14E-08	-0.008	0.026	0.757	30.082	6.51E-05	NA
rs1899689	7	121964349	C	T	0.380	-0.012	0.002	4.27E-08	-0.006	0.023	0.783	30.021	6.50E-05	NA
rs1914888	17	21267541	A	G	0.462	0.013	0.002	1.33E-09	0.038	0.024	0.121	36.762	7.96E-05	NA
rs192812	3	161491831	C	T	0.454	-0.012	0.002	4.87E-09	-0.021	0.025	0.412	34.241	7.41E-05	NA
rs1945941	11	132620118	G	A	0.376	0.012	0.002	3.63E-08	-0.007	0.026	0.774	30.337	6.57E-05	NA
rs2002023	10	76848524	C	T	0.441	-0.012	0.002	8.92E-09	-0.009	0.024	0.708	33.064	7.16E-05	NA
rs2015407	14	103274547	A	G	0.345	0.014	0.002	4.68E-10	0.006	0.024	0.823	38.808	8.40E-05	NA
rs2035831	3	173683791	G	C	0.321	0.013	0.002	1.48E-08	0.007	0.025	0.771	32.085	6.95E-05	NA
rs2060604	8	76650334	T	C	0.414	0.015	0.002	2.80E-12	0.006	0.022	0.785	48.822	1.06E-04	NA
rs2065418	11	30422068	T	G	0.354	0.016	0.002	1.39E-12	-0.009	0.023	0.708	50.196	1.09E-04	NA
rs2066295	6	26168903	A	G	0.219	0.023	0.003	5.73E-18	0.031	0.026	0.226	74.611	1.61E-04	NA
rs2074881	19	1970021	C	T	0.179	0.015	0.003	2.25E-08	0.041	0.040	0.303	31.270	6.77E-05	NA
rs2075226	2	36805676	A	G	0.396	-0.013	0.002	2.48E-08	-0.004	0.023	0.866	31.073	6.73E-05	NA
rs208015	17	46252346	T	C	0.088	0.022	0.004	1.07E-08	0.002	0.043	0.958	32.711	7.08E-05	NA
rs2143253	20	41987392	G	A	0.137	0.019	0.003	1.29E-09	0.037	0.036	0.297	36.823	7.97E-05	NA
rs2180454	14	29690513	T	C	0.241	-0.015	0.003	1.90E-09	0.044	0.027	0.108	36.071	7.81E-05	NA

rs2237717	7	116405387	T	C	0.443	-0.011	0.002	2.63E-08	-0.024	0.022	0.289	30.960	6.70E-05	NA
rs2242449	17	7095507	C	T	0.417	-0.013	0.002	2.85E-09	-0.011	0.029	0.698	35.287	7.64E-05	NA
rs227199	1	210265384	G	C	0.316	0.013	0.002	5.06E-09	0.011	0.023	0.649	34.168	7.40E-05	NA
rs2272168	7	99102777	G	C	0.163	0.017	0.003	3.68E-09	-0.009	0.031	0.778	34.785	7.53E-05	NA
rs2275543	9	107651174	T	C	0.099	-0.019	0.003	6.58E-09	0.006	0.037	0.861	33.655	7.29E-05	NA
rs2298969	4	3186244	A	G	0.490	0.012	0.002	2.79E-08	0.015	0.022	0.509	30.851	6.68E-05	NA
rs2299383	7	103418846	C	T	0.410	-0.014	0.002	2.20E-10	0.012	0.022	0.606	40.282	8.72E-05	NA
rs2307111	5	75003678	T	C	0.396	0.025	0.002	4.41E-29	0.020	0.022	0.373	125.285	2.71E-04	NA
rs2396625	7	113028634	T	A	0.401	0.014	0.002	1.99E-10	0.012	0.023	0.604	40.473	8.76E-05	NA
rs2436726	6	40366427	C	T	0.405	-0.016	0.002	2.24E-12	0.023	0.026	0.379	49.262	1.07E-04	NA
rs2453763	5	92376460	T	A	0.347	-0.012	0.002	3.72E-08	-0.046	0.023	0.046	30.290	6.56E-05	NA
rs2470945	7	104585760	C	A	0.408	-0.012	0.002	2.10E-08	-0.032	0.023	0.150	31.396	6.80E-05	NA
rs2516740	16	2097110	A	C	0.226	0.020	0.003	7.50E-14	-0.027	0.033	0.415	55.934	1.21E-04	NA
rs2526754	3	50125996	G	A	0.494	0.022	0.002	5.98E-22	-0.012	0.022	0.573	92.735	2.01E-04	Educational attainment; Intelligence
rs2531995	16	4013467	C	T	0.387	-0.019	0.002	2.80E-17	0.010	0.024	0.675	71.477	1.55E-04	NA
rs2596933	3	9365746	G	A	0.267	0.014	0.002	1.26E-08	0.033	0.029	0.255	32.396	7.01E-05	NA
rs261336	15	58742418	G	A	0.161	-0.020	0.003	2.07E-14	0.011	0.031	0.721	58.461	1.27E-04	NA
rs2781668	6	131897278	C	T	0.170	-0.015	0.003	3.64E-08	-0.005	0.031	0.874	30.331	6.57E-05	NA
rs2787693	1	49915848	C	A	0.302	0.017	0.002	1.94E-11	0.016	0.023	0.505	45.027	9.75E-05	NA
rs2814944	6	34552797	G	A	0.137	-0.034	0.003	1.03E-26	-0.009	0.030	0.761	114.463	2.48E-04	NA
rs2820232	6	35003603	G	T	0.459	-0.012	0.002	2.95E-08	-0.013	0.022	0.556	30.741	6.65E-05	NA
rs2836753	21	40291187	T	C	0.385	-0.013	0.002	7.29E-10	0.040	0.024	0.096	37.941	8.21E-05	NA
rs2861688	2	67838474	T	C	0.273	0.016	0.002	8.97E-11	0.016	0.025	0.506	42.033	9.10E-05	NA
rs2925979	16	81534790	T	C	0.288	0.014	0.002	1.10E-10	-0.001	0.025	0.982	41.628	9.01E-05	NA
rs2968856	7	150614934	T	C	0.261	0.016	0.002	1.80E-11	0.013	0.031	0.670	45.179	9.78E-05	NA
rs320	8	19819077	T	G	0.287	0.031	0.002	2.30E-43	-0.028	0.026	0.287	190.642	4.13E-04	NA
rs323781	5	86752616	A	C	0.034	-0.032	0.006	3.82E-08	-0.023	0.067	0.727	30.237	6.55E-05	NA
rs349088	11	84814393	C	A	0.480	0.013	0.002	3.23E-09	0.003	0.022	0.903	35.040	7.59E-05	NA
rs350832	19	4069426	G	A	0.227	-0.014	0.003	1.88E-08	0.036	0.030	0.234	31.615	6.84E-05	NA
rs3749262	3	131624097	G	A	0.263	-0.019	0.002	1.12E-14	0.001	0.025	0.957	59.681	1.29E-04	NA
rs3808436	8	116560967	A	G	0.432	0.016	0.002	1.58E-13	-0.010	0.022	0.660	54.468	1.18E-04	NA
rs3810291	19	47569003	G	A	0.337	-0.019	0.002	2.74E-17	-0.034	0.029	0.240	71.521	1.55E-04	NA
rs3814424	5	87968953	C	T	0.172	-0.025	0.003	2.55E-16	-0.037	0.030	0.213	67.124	1.45E-04	Intelligence
rs3814883	16	29994922	C	T	0.460	-0.026	0.002	7.26E-31	0.035	0.028	0.210	133.436	2.89E-04	Nervous feelings
rs3852012	3	44045981	G	A	0.287	0.013	0.002	1.57E-08	-0.039	0.025	0.125	31.963	6.92E-05	NA
rs3864004	3	41240177	G	A	0.432	-0.014	0.002	2.30E-09	0.000	0.022	0.992	35.700	7.73E-05	NA
rs3902840	9	129419025	G	A	0.092	-0.021	0.004	1.72E-08	-0.003	0.039	0.946	31.786	6.88E-05	NA
rs397092	21	46582564	A	G	0.456	-0.013	0.002	1.07E-09	-0.027	0.022	0.224	37.196	8.05E-05	NA
rs40067	5	107439012	G	A	0.180	0.018	0.003	4.05E-11	0.027	0.030	0.360	43.590	9.44E-05	NA
rs4074448	15	63888495	G	A	0.434	0.012	0.002	2.37E-08	0.041	0.026	0.116	31.161	6.75E-05	NA
rs4283409	2	228977142	A	G	0.387	-0.012	0.002	7.64E-09	0.025	0.026	0.341	33.365	7.22E-05	NA
rs429343	2	147903382	A	G	0.437	0.012	0.002	1.26E-08	-0.025	0.023	0.276	32.394	7.01E-05	NA
rs4377779	6	12117344	T	C	0.334	0.013	0.002	5.27E-09	-0.011	0.023	0.634	34.086	7.38E-05	NA
rs4410790	7	17284577	T	C	0.385	-0.012	0.002	5.72E-09	-0.010	0.024	0.665	33.927	7.34E-05	NA
rs442177	4	88030261	G	T	0.421	-0.011	0.002	3.89E-08	-0.020	0.023	0.368	30.204	6.54E-05	NA
rs4477562	13	54104968	C	T	0.110	-0.020	0.003	9.80E-09	-0.018	0.036	0.620	32.880	7.12E-05	NA
rs4482463	2	205375909	C	A	0.081	0.029	0.004	4.75E-12	0.016	0.046	0.722	47.787	1.03E-04	NA
rs4660208	1	39722214	A	G	0.195	-0.023	0.003	2.84E-18	0.025	0.027	0.356	75.995	1.64E-04	NA
rs4665991	2	27766284	G	A	0.264	-0.013	0.002	2.16E-09	-0.001	0.025	0.971	35.821	7.75E-05	NA
rs4671328	2	58935282	T	G	0.430	0.018	0.002	9.13E-17	-0.010	0.025	0.682	69.149	1.50E-04	NA
rs4693325	4	94408529	A	C	0.199	-0.016	0.003	1.74E-09	0.007	0.029	0.800	36.241	7.85E-05	NA

rs4740619	9	15634326	T	C	0.481	0.014	0.002	6.33E-11	0.007	0.022	0.765	42.715	9.25E-05	NA
rs4755720	11	43628749	C	T	0.400	0.016	0.002	4.50E-14	-0.009	0.024	0.713	56.935	1.23E-04	NA
rs4777542	15	73082366	C	T	0.300	0.014	0.002	3.14E-09	0.023	0.029	0.432	35.098	7.60E-05	NA
rs4784745	16	57014875	A	G	0.329	-0.019	0.002	4.00E-21	0.014	0.026	0.602	88.973	1.93E-04	NA
rs4790292	17	1824305	C	A	0.144	0.020	0.003	2.66E-10	-0.005	0.035	0.889	39.913	8.64E-05	NA
rs479315	11	66064991	G	C	0.170	-0.016	0.003	2.08E-08	-0.018	0.028	0.525	31.420	6.80E-05	NA
rs4805758	19	32866424	G	A	0.162	0.020	0.003	5.55E-13	0.027	0.030	0.377	51.999	1.13E-04	Fed-up feelings
rs4810479	20	44545048	C	T	0.288	0.013	0.002	6.17E-10	0.004	0.025	0.863	38.267	8.28E-05	NA
rs4889782	17	78640510	C	T	0.415	0.013	0.002	1.61E-09	0.047	0.024	0.046	36.391	7.88E-05	NA
rs4890439	18	40732586	G	A	0.375	0.012	0.002	4.60E-08	0.038	0.023	0.095	29.879	6.47E-05	NA
rs4929923	11	8639200	T	C	0.377	-0.015	0.002	4.28E-11	0.039	0.023	0.091	43.484	9.41E-05	NA
rs4947406	7	50661735	T	G	0.350	0.016	0.002	9.35E-12	0.023	0.024	0.347	46.461	1.01E-04	NA
rs4971632	2	50240091	T	C	0.166	0.017	0.003	1.26E-09	-0.050	0.030	0.097	36.870	7.98E-05	NA
rs545608	1	177899121	G	C	0.186	-0.038	0.003	1.86E-37	0.037	0.028	0.175	163.594	3.54E-04	NA
rs563296	10	99772404	G	A	0.441	-0.013	0.002	1.23E-08	-0.013	0.022	0.561	32.438	7.02E-05	NA
rs6115015	20	25027692	G	A	0.333	-0.013	0.002	6.30E-09	0.040	0.029	0.162	33.741	7.30E-05	NA
rs619054	11	116660813	G	A	0.232	0.015	0.002	2.70E-11	0.014	0.027	0.600	44.384	9.61E-05	NA
rs645040	3	135926622	G	T	0.229	-0.023	0.002	1.31E-20	0.038	0.027	0.150	86.621	1.87E-04	NA
rs6463489	7	5542513	T	C	0.069	0.024	0.004	1.03E-08	-0.007	0.037	0.843	32.787	7.10E-05	NA
rs649458	1	23712528	T	A	0.137	0.018	0.003	5.66E-09	0.046	0.032	0.152	33.949	7.35E-05	NA
rs6499240	16	69686912	A	G	0.422	-0.020	0.002	1.52E-18	-0.011	0.022	0.625	77.230	1.67E-04	NA
rs6499861	16	56991495	C	G	0.162	-0.018	0.003	8.61E-12	-0.010	0.033	0.756	46.623	1.01E-04	NA
rs6545714	2	59307725	G	A	0.382	0.019	0.002	3.15E-18	0.011	0.023	0.643	75.796	1.64E-04	NA
rs6548834	3	82704753	G	A	0.378	-0.013	0.002	1.08E-08	-0.026	0.023	0.258	32.694	7.08E-05	NA
rs6550597	3	18738940	G	A	0.298	-0.013	0.002	4.61E-09	-0.045	0.025	0.066	34.346	7.43E-05	NA
rs6575340	14	94023972	G	A	0.377	-0.014	0.002	8.82E-10	-0.002	0.023	0.937	37.571	8.13E-05	NA
rs6577584	1	6715390	T	G	0.372	-0.014	0.002	1.65E-10	-0.040	0.023	0.085	40.843	8.84E-05	NA
rs6602151	10	16764483	T	C	0.294	-0.015	0.002	5.89E-10	-0.007	0.026	0.773	38.356	8.30E-05	NA
rs6603004	15	84568504	A	G	0.466	-0.013	0.002	1.81E-09	0.007	0.022	0.757	36.163	7.83E-05	NA
rs6673977	1	7736097	A	G	0.354	-0.012	0.002	1.63E-08	-0.022	0.024	0.363	31.892	6.90E-05	NA
rs6711375	2	161090873	G	A	0.320	-0.014	0.002	1.11E-09	-0.006	0.024	0.811	37.115	8.03E-05	NA
rs6754311	2	136707982	T	C	0.489	0.013	0.002	3.95E-10	0.023	0.030	0.438	39.137	8.47E-05	NA
rs676387	17	40706273	C	A	0.266	-0.013	0.002	3.42E-08	-0.030	0.025	0.226	30.452	6.59E-05	NA
rs6870983	5	87697533	C	T	0.236	0.020	0.003	1.66E-15	-0.025	0.027	0.357	63.434	1.37E-04	NA
rs687672	11	65649984	T	C	0.189	0.021	0.003	5.92E-14	0.051	0.029	0.079	56.399	1.22E-04	NA
rs6907923	6	51802669	G	A	0.341	-0.015	0.002	1.71E-10	-0.020	0.023	0.399	40.774	8.83E-05	NA
rs7016529	8	19806631	T	C	0.011	-0.102	0.009	1.94E-27	0.006	0.102	0.952	117.778	2.55E-04	NA
rs703965	10	80955067	C	T	0.456	-0.012	0.002	8.25E-09	0.007	0.024	0.765	33.214	7.19E-05	NA
rs705986	8	64737682	A	G	0.491	0.012	0.002	2.53E-08	-0.036	0.022	0.101	31.039	6.72E-05	NA
rs7075195	10	65050659	A	G	0.435	0.017	0.002	5.31E-16	-0.007	0.022	0.749	65.676	1.42E-04	Educational attainment
rs7084454	10	21821274	G	A	0.338	-0.016	0.002	9.04E-13	0.017	0.026	0.502	51.042	1.10E-04	NA
rs7132908	12	50263148	G	A	0.357	-0.019	0.002	9.07E-15	0.044	0.026	0.092	60.088	1.30E-04	NA
rs7144011	14	79940383	G	T	0.207	-0.023	0.003	1.71E-17	0.045	0.027	0.093	72.451	1.57E-04	NA
rs7186893	16	24806420	G	T	0.267	0.020	0.002	3.04E-16	0.046	0.025	0.064	66.779	1.45E-04	Mood swings
rs7198357	16	67884619	C	T	0.178	0.017	0.003	4.09E-11	-0.009	0.030	0.753	43.573	9.43E-05	NA
rs7206608	16	82872628	C	G	0.313	-0.013	0.002	1.26E-08	-0.006	0.024	0.793	32.386	7.01E-05	NA
rs7241918	18	47160953	G	T	0.163	0.018	0.003	7.52E-13	-0.009	0.030	0.760	51.403	1.11E-04	NA
rs7323827	13	94116675	A	G	0.304	0.013	0.002	4.59E-09	0.030	0.024	0.212	34.358	7.44E-05	NA
rs741959	1	47676233	A	G	0.402	0.014	0.002	6.19E-11	-0.008	0.023	0.726	42.760	9.26E-05	NA
rs7488867	12	103699685	C	T	0.281	0.015	0.003	1.57E-09	-0.019	0.025	0.447	36.447	7.89E-05	NA
rs7515635	1	42408070	T	C	0.471	0.012	0.002	4.68E-09	-0.006	0.022	0.800	34.317	7.43E-05	NA

rs7537581	1	2725475	C	A	0.476	-0.012	0.002	4.55E-08	0.027	0.026	0.298	29.899	6.47E-05	NA
rs7549987	1	80789011	C	T	0.299	-0.014	0.002	4.02E-09	0.005	0.024	0.852	34.614	7.49E-05	NA
rs7581217	2	25524944	T	C	0.370	0.013	0.002	5.93E-09	0.055	0.029	0.055	33.857	7.33E-05	NA
rs7601028	2	642499	C	G	0.174	-0.046	0.003	1.02E-52	-0.058	0.030	0.050	233.512	5.05E-04	NA
rs7638388	3	47914974	C	T	0.088	-0.024	0.004	1.01E-10	0.020	0.039	0.601	41.794	9.05E-05	NA
rs7668263	4	143781835	T	C	0.382	-0.012	0.002	4.21E-08	0.025	0.023	0.272	30.049	6.50E-05	NA
rs779655	5	170495158	G	C	0.272	-0.014	0.002	1.21E-08	-0.003	0.025	0.909	32.464	7.03E-05	NA
rs7824519	8	14222796	T	G	0.324	-0.013	0.002	1.51E-08	-0.005	0.024	0.827	32.035	6.93E-05	NA
rs7928842	11	47566352	T	C	0.422	-0.020	0.002	2.30E-20	0.035	0.023	0.123	85.511	1.85E-04	NA
rs7947951	11	13356030	A	G	0.297	-0.014	0.002	1.59E-10	0.002	0.024	0.946	40.911	8.86E-05	NA
rs7962636	12	116450006	T	C	0.355	0.013	0.002	1.32E-08	0.012	0.023	0.594	32.308	6.99E-05	NA
rs7982447	13	54453811	T	C	0.200	-0.019	0.003	6.75E-13	0.050	0.027	0.065	51.616	1.12E-04	NA
rs8015400	14	25930988	C	A	0.348	-0.018	0.002	8.13E-15	-0.002	0.024	0.926	60.303	1.31E-04	NA
rs8055138	16	28891465	C	T	0.329	-0.030	0.002	1.86E-35	0.027	0.023	0.236	154.432	3.34E-04	Educational attainment; Intelligence
rs8078079	17	34959346	T	C	0.395	0.015	0.002	2.89E-11	-0.018	0.024	0.430	44.247	9.58E-05	NA
rs808820	12	89744826	A	G	0.116	-0.022	0.004	8.37E-10	0.005	0.034	0.879	37.671	8.15E-05	NA
rs818524	1	85201228	T	C	0.289	-0.013	0.002	2.84E-08	0.006	0.026	0.801	30.812	6.67E-05	NA
rs8192675	3	170724883	T	C	0.289	-0.013	0.002	4.30E-08	0.005	0.024	0.851	30.007	6.50E-05	NA
rs891387	18	21103909	T	C	0.473	0.021	0.002	3.25E-21	-0.022	0.022	0.321	89.383	1.93E-04	Educational attainment
rs906673	3	12898941	C	T	0.389	0.013	0.002	3.67E-09	0.009	0.024	0.710	34.789	7.53E-05	NA
rs9277972	6	33276770	A	T	0.168	-0.016	0.003	1.55E-08	0.033	0.030	0.272	31.995	6.93E-05	NA
rs938875	15	67952922	A	G	0.408	-0.019	0.002	3.72E-18	-0.026	0.023	0.244	75.463	1.63E-04	NA
rs9478499	6	154345289	T	A	0.147	-0.019	0.003	1.35E-10	-0.021	0.030	0.486	41.238	8.93E-05	NA
rs9556979	13	99241507	T	G	0.324	-0.013	0.002	4.53E-09	-0.031	0.031	0.313	34.382	7.44E-05	NA
rs9563569	13	58571207	G	A	0.150	0.018	0.003	2.09E-08	-0.007	0.029	0.819	31.408	6.80E-05	NA
rs9630985	2	181607676	A	C	0.349	-0.014	0.002	9.30E-11	0.026	0.024	0.270	41.963	9.08E-05	NA
rs9686661	5	55861786	C	T	0.186	-0.023	0.003	4.65E-18	0.000	0.029	1.000	75.025	1.62E-04	NA
rs9816226	3	185834499	A	T	0.188	-0.016	0.003	8.56E-09	-0.021	0.029	0.476	33.144	7.17E-05	NA
rs9872156	3	176337348	G	A	0.260	-0.013	0.002	4.04E-08	-0.004	0.025	0.874	30.129	6.52E-05	NA
rs987237	6	50803050	A	G	0.181	-0.030	0.003	7.81E-26	0.003	0.028	0.909	110.449	2.39E-04	NA
rs9947450	18	58052665	C	T	0.030	0.062	0.007	1.22E-21	0.007	0.058	0.909	91.321	1.98E-04	NA
rs998584	6	43757896	C	A	0.496	-0.013	0.002	9.99E-11	-0.037	0.027	0.169	41.823	9.05E-05	NA
rs9987289	8	9183358	A	G	0.075	0.022	0.004	1.84E-09	-0.045	0.039	0.243	36.136	7.82E-05	NA

Genomic positions reported in the GWAS of Mets refer to human reference assembly (GRCh37/hg19).

Abbreviations:MetS, Metabolic syndrome; ALC, Alcohol dependence; SNPs, Single nucleotide polymorphisms; CHR, Chromosome; EA, Effect allele; OA, Other allele; EAF, Effect allele frequency; SE, Standard error; PVE, Proportion of phenotypic variance explained.

Palindromic and not-inferrable SNPs that are abandoned as instruments: rs1937433, rs2253310, rs6916318.

2 SNPs detected by PRESSO outlier test, and 12 SNPs were detected by MR-Radial outlier test.

Supplementary Table 4. Genetic instruments for the causal relationship of Mets and ANO.

SNP	CHR	Position	EA	OA	EAF	Beta for exposure	SE for exposure	P value for exposure	Beta for outcome	SE for outcome	P value for outcome	F statistics	PVE	Association with potential confounding factors
rs10116353	9	111950088	G	T	0.307	0.013	0.002	9.39E-09	0.014	0.015	0.333	32.964	7.14E-05	NA
rs1013402	11	27712381	A	G	0.290	-0.025	0.002	1.34E-23	-0.035	0.014	0.016	100.255	2.17E-04	NA
rs10160660	11	76474537	T	C	0.248	0.013	0.002	4.83E-08	0.017	0.017	0.293	29.786	6.45E-05	NA
rs102275	11	61557803	T	C	0.363	-0.013	0.002	6.28E-11	-0.013	0.014	0.366	42.731	9.25E-05	NA
rs1032524	1	201790826	T	C	0.484	-0.015	0.002	6.01E-12	-0.002	0.013	0.902	47.325	1.02E-04	NA
rs1042034	2	21225281	C	T	0.218	-0.020	0.002	7.07E-21	-0.017	0.016	0.284	87.848	1.90E-04	NA
rs1046080	6	31595882	C	A	0.233	-0.015	0.003	1.75E-09	-0.017	0.015	0.279	36.231	7.84E-05	NA
rs10734924	12	123018894	T	G	0.259	-0.015	0.003	2.19E-09	-0.009	0.015	0.574	35.801	7.75E-05	NA
rs10768994	11	43936945	T	C	0.430	0.012	0.002	4.52E-08	-0.001	0.014	0.938	29.912	6.48E-05	NA
rs10779835	1	230299949	T	C	0.391	0.015	0.002	2.45E-14	-0.008	0.014	0.536	58.131	1.26E-04	NA
rs10808546	8	126495818	C	T	0.431	0.016	0.002	4.56E-19	0.003	0.014	0.836	79.612	1.72E-04	NA
rs10838620	11	46818098	G	A	0.214	-0.015	0.002	1.25E-09	0.015	0.016	0.336	36.883	7.98E-05	NA
rs10887579	10	88097210	A	C	0.354	-0.013	0.002	4.09E-09	0.021	0.015	0.148	34.579	7.49E-05	NA
rs10938397	4	45182527	A	G	0.420	-0.023	0.002	8.14E-26	-0.008	0.014	0.545	110.367	2.39E-04	NA
rs11079849	17	47090785	C	T	0.290	0.015	0.002	6.90E-11	0.000	0.015	0.991	42.547	9.21E-05	NA
rs11187845	10	96060198	C	A	0.096	0.020	0.004	8.08E-09	0.037	0.024	0.120	33.256	7.20E-05	NA
rs1138429	16	56942921	A	T	0.101	-0.028	0.003	1.42E-18	0.042	0.027	0.120	77.365	1.67E-04	NA
rs1159974	6	126090277	T	C	0.499	-0.013	0.002	3.30E-10	-0.010	0.013	0.447	39.489	8.55E-05	NA
rs11611246	12	939480	G	T	0.183	-0.016	0.003	1.14E-08	-0.001	0.018	0.950	32.583	7.05E-05	NA
rs11631408	15	51746236	A	G	0.463	0.014	0.002	2.44E-11	0.021	0.014	0.128	44.582	9.65E-05	NA
rs11636565	15	36391846	C	A	0.488	-0.014	0.002	6.84E-12	-0.016	0.014	0.246	47.072	1.02E-04	NA
rs11660335	18	22154235	T	C	0.161	0.018	0.003	1.07E-09	0.013	0.017	0.441	37.189	8.05E-05	NA
rs11676272	2	25141538	A	G	0.464	-0.020	0.002	1.05E-17	-0.008	0.013	0.572	73.421	1.59E-04	NA
rs11747810	5	153100273	T	C	0.495	0.013	0.002	1.83E-09	-0.007	0.014	0.615	36.148	7.83E-05	NA
rs11826999	11	116553778	G	A	0.223	-0.023	0.002	6.07E-26	-0.008	0.016	0.623	110.950	2.40E-04	NA
rs11866815	16	387867	C	T	0.242	0.016	0.002	2.32E-10	-0.008	0.017	0.636	40.181	8.70E-05	NA
rs11880870	19	18830704	A	G	0.499	0.013	0.002	2.98E-09	-0.016	0.014	0.264	35.198	7.62E-05	NA
rs11981190	7	71659538	G	A	0.341	0.013	0.002	5.84E-09	-0.013	0.014	0.365	33.887	7.34E-05	NA
rs1200349	15	41821752	T	C	0.482	-0.013	0.002	1.36E-09	-0.024	0.013	0.078	36.724	7.95E-05	NA
rs12201987	6	143182396	C	T	0.205	0.016	0.003	1.87E-09	-0.002	0.017	0.902	36.103	7.82E-05	NA
rs12513212	4	96149346	A	C	0.387	-0.014	0.002	2.85E-10	-0.014	0.014	0.311	39.778	8.61E-05	NA
rs12520822	5	105699867	C	T	0.408	0.012	0.002	1.84E-08	0.017	0.014	0.214	31.658	6.85E-05	NA
rs12596491	16	88928417	C	T	0.340	0.012	0.002	4.58E-08	0.007	0.016	0.689	29.888	6.47E-05	NA
rs12608504	19	18389135	A	G	0.359	0.016	0.002	2.27E-13	-0.003	0.014	0.806	53.757	1.16E-04	NA
rs12624640	20	32952125	G	A	0.430	-0.012	0.002	7.17E-09	0.000	0.014	0.978	33.489	7.25E-05	NA
rs12824567	12	124495203	G	C	0.350	0.013	0.002	1.94E-09	-0.019	0.015	0.185	36.032	7.80E-05	NA
rs12967135	18	57849023	G	A	0.240	-0.046	0.003	3.09E-66	0.011	0.016	0.473	295.539	6.39E-04	Worrier or anxious feelings
rs12981256	19	1865901	G	A	0.475	-0.016	0.002	7.75E-14	-0.028	0.017	0.093	55.867	1.21E-04	NA
rs13024601	2	100902032	C	T	0.346	0.014	0.002	1.39E-09	0.022	0.014	0.109	36.680	7.94E-05	NA
rs13029936	2	208465544	C	T	0.176	0.017	0.003	1.31E-09	0.035	0.017	0.038	36.795	7.96E-05	NA
rs13107325	4	103188709	C	T	0.080	-0.023	0.004	1.94E-09	-0.002	0.029	0.938	36.033	7.80E-05	Intelligence
rs13206549	6	162978032	T	G	0.134	0.019	0.003	1.54E-09	0.011	0.020	0.589	36.486	7.90E-05	NA
rs13244268	7	72911843	T	C	0.114	0.024	0.003	9.17E-18	-0.020	0.021	0.341	73.684	1.59E-04	NA
rs13275593	8	68257300	G	A	0.285	-0.015	0.002	2.14E-10	-0.024	0.015	0.112	40.332	8.73E-05	NA
rs1330304	9	16714629	C	G	0.267	0.013	0.002	2.32E-08	-0.003	0.016	0.838	31.202	6.75E-05	NA
rs13322435	3	156795468	A	G	0.407	0.020	0.002	6.60E-20	0.021	0.014	0.134	83.432	1.81E-04	NA
rs13416004	2	69664976	C	T	0.415	0.012	0.002	3.16E-08	0.002	0.014	0.905	30.605	6.63E-05	NA
rs13416667	2	56608267	C	A	0.274	-0.015	0.002	2.32E-10	-0.026	0.017	0.126	40.179	8.70E-05	NA
rs13435860	4	136975545	A	T	0.368	-0.013	0.002	1.94E-09	0.020	0.015	0.165	36.037	7.80E-05	NA

rs1362910	8	30856464	A	G	0.386	0.014	0.002	9.70E-11	0.002	0.014	0.877	41.882	9.07E-05	NA
rs1363695	5	130378027	C	T	0.228	0.015	0.003	6.49E-09	0.010	0.016	0.553	33.681	7.29E-05	NA
rs1412239	9	28425515	C	G	0.304	-0.022	0.002	2.39E-21	-0.002	0.015	0.870	89.993	1.95E-04	NA
rs1431659	8	73439070	A	G	0.278	0.019	0.002	4.48E-15	0.031	0.016	0.051	61.476	1.33E-04	NA
rs1448613	3	85875265	G	A	0.386	0.014	0.002	2.40E-10	-0.008	0.014	0.544	40.110	8.68E-05	NA
rs1456116	3	123084379	G	A	0.417	-0.013	0.002	1.99E-09	0.013	0.014	0.329	35.987	7.79E-05	NA
rs1458156	12	41887940	C	T	0.449	-0.013	0.002	4.45E-09	0.002	0.013	0.873	34.416	7.45E-05	NA
rs1507153	6	79484386	C	A	0.364	-0.013	0.002	3.86E-09	0.009	0.014	0.525	34.694	7.51E-05	NA
rs1534696	7	26397239	C	A	0.439	0.014	0.002	1.24E-11	-0.008	0.014	0.576	45.905	9.94E-05	NA
rs17115145	14	30122409	C	T	0.406	-0.012	0.002	1.29E-08	-0.014	0.014	0.302	32.350	7.00E-05	NA
rs17162333	1	27236757	C	G	0.132	-0.020	0.003	6.24E-11	0.002	0.019	0.907	42.744	9.25E-05	NA
rs17391694	1	78623626	C	T	0.096	-0.026	0.004	2.39E-12	-0.015	0.021	0.467	49.132	1.06E-04	NA
rs17513613	19	30286822	T	C	0.289	-0.017	0.002	1.94E-13	-0.025	0.015	0.083	54.063	1.17E-04	NA
rs17618704	17	46221010	T	G	0.068	0.025	0.004	1.52E-09	-0.024	0.027	0.383	36.510	7.90E-05	NA
rs17639546	3	61251635	G	A	0.148	0.018	0.003	3.65E-09	0.025	0.019	0.195	34.803	7.53E-05	NA
rs17783751	16	89513984	C	A	0.176	-0.015	0.003	4.79E-08	0.019	0.017	0.271	29.800	6.45E-05	NA
rs17806379	20	51107290	C	T	0.185	0.020	0.003	3.64E-13	0.035	0.018	0.047	52.829	1.14E-04	NA
rs17821316	15	58703069	A	C	0.488	-0.012	0.002	9.97E-10	-0.003	0.015	0.827	37.331	8.08E-05	NA
rs1784460	11	118938371	T	A	0.360	-0.015	0.002	8.15E-12	0.022	0.014	0.114	46.729	1.01E-04	NA
rs1876359	16	4930100	C	T	0.377	-0.013	0.002	5.40E-09	-0.001	0.014	0.949	34.038	7.37E-05	NA
rs1886558	13	111982615	A	G	0.389	0.012	0.002	4.14E-08	0.001	0.014	0.929	30.082	6.51E-05	NA
rs1899689	7	121964349	C	T	0.380	-0.012	0.002	4.27E-08	-0.019	0.014	0.176	30.021	6.50E-05	NA
rs1914888	17	21267541	A	G	0.462	0.013	0.002	1.33E-09	0.006	0.015	0.675	36.762	7.96E-05	NA
rs192812	3	161491831	C	T	0.454	-0.012	0.002	4.87E-09	-0.015	0.014	0.265	34.241	7.41E-05	NA
rs1945941	11	132620118	G	A	0.376	0.012	0.002	3.63E-08	0.016	0.014	0.231	30.337	6.57E-05	NA
rs2002023	10	76848524	C	T	0.441	-0.012	0.002	8.92E-09	0.002	0.014	0.879	33.064	7.16E-05	NA
rs2015407	14	103274547	A	G	0.345	0.014	0.002	4.68E-10	0.016	0.014	0.266	38.808	8.40E-05	NA
rs2035831	3	173683791	G	C	0.321	0.013	0.002	1.48E-08	0.012	0.014	0.395	32.085	6.95E-05	NA
rs2060604	8	76650334	T	C	0.414	0.015	0.002	2.80E-12	-0.014	0.014	0.312	48.822	1.06E-04	NA
rs2066295	6	26168903	A	G	0.219	0.023	0.003	5.73E-18	-0.008	0.016	0.628	74.611	1.61E-04	NA
rs2074881	19	1970021	C	T	0.179	0.015	0.003	2.25E-08	-0.014	0.021	0.507	31.270	6.77E-05	NA
rs2075226	2	36805676	A	G	0.396	-0.013	0.002	2.48E-08	-0.031	0.014	0.026	31.073	6.73E-05	NA
rs208015	17	46252346	T	C	0.088	0.022	0.004	1.07E-08	0.045	0.026	0.084	32.711	7.08E-05	NA
rs2143253	20	41987392	G	A	0.137	0.019	0.003	1.29E-09	0.007	0.021	0.755	36.823	7.97E-05	NA
rs2180454	14	29690513	T	C	0.241	-0.015	0.003	1.90E-09	-0.025	0.016	0.106	36.071	7.81E-05	NA
rs227199	1	210265384	G	C	0.316	0.013	0.002	5.06E-09	-0.009	0.015	0.536	34.168	7.40E-05	NA
rs2275543	9	107651174	T	C	0.099	-0.019	0.003	6.58E-09	-0.013	0.022	0.578	33.655	7.29E-05	NA
rs2298969	4	3186244	A	G	0.490	0.012	0.002	2.79E-08	-0.010	0.014	0.463	30.851	6.68E-05	NA
rs2299383	7	103418846	C	T	0.410	-0.014	0.002	2.20E-10	-0.006	0.014	0.638	40.282	8.72E-05	NA
rs2307111	5	75003678	T	C	0.396	0.025	0.002	4.41E-29	0.014	0.014	0.317	125.285	2.71E-04	NA
rs2436726	6	40366427	C	T	0.405	-0.016	0.002	2.24E-12	0.003	0.014	0.840	49.262	1.07E-04	NA
rs2453763	5	92376460	T	A	0.347	-0.012	0.002	3.72E-08	0.018	0.014	0.215	30.290	6.56E-05	NA
rs2470945	7	104585760	C	A	0.408	-0.012	0.002	2.10E-08	0.003	0.014	0.850	31.396	6.80E-05	NA
rs2516740	16	2097110	A	C	0.226	0.020	0.003	7.50E-14	-0.017	0.019	0.384	55.934	1.21E-04	NA
rs2531995	16	4013467	C	T	0.387	-0.019	0.002	2.80E-17	0.004	0.015	0.780	71.477	1.55E-04	NA
rs2596933	3	9365746	G	A	0.267	0.014	0.002	1.26E-08	0.027	0.017	0.103	32.396	7.01E-05	NA
rs261336	15	58742418	G	A	0.161	-0.020	0.003	2.07E-14	-0.021	0.018	0.242	58.461	1.27E-04	NA
rs2781668	6	131897278	C	T	0.170	-0.015	0.003	3.64E-08	-0.015	0.019	0.416	30.331	6.57E-05	NA
rs2787693	1	49915848	C	A	0.302	0.017	0.002	1.94E-11	0.015	0.014	0.310	45.027	9.75E-05	NA
rs2814944	6	34552797	G	A	0.137	-0.034	0.003	1.03E-26	-0.030	0.018	0.096	114.463	2.48E-04	NA
rs2820232	6	35003603	G	T	0.459	-0.012	0.002	2.95E-08	0.005	0.014	0.716	30.741	6.65E-05	NA

rs2836753	21	40291187	T	C	0.385	-0.013	0.002	7.29E-10	0.007	0.014	0.610	37.941	8.21E-05	NA
rs2861688	2	67838474	T	C	0.273	0.016	0.002	8.97E-11	0.008	0.015	0.610	42.033	9.10E-05	NA
rs2925979	16	81534790	T	C	0.288	0.014	0.002	1.10E-10	-0.003	0.015	0.818	41.628	9.01E-05	NA
rs2968856	7	150614934	T	C	0.261	0.016	0.002	1.80E-11	-0.004	0.016	0.789	45.179	9.78E-05	NA
rs320	8	19819077	T	G	0.287	0.031	0.002	2.30E-43	0.024	0.015	0.107	190.642	4.13E-04	NA
rs323781	5	86752616	A	C	0.034	-0.032	0.006	3.82E-08	-0.045	0.043	0.293	30.237	6.55E-05	NA
rs349088	11	84814393	C	A	0.480	0.013	0.002	3.23E-09	-0.012	0.014	0.379	35.040	7.59E-05	NA
rs350832	19	4069426	G	A	0.227	-0.014	0.003	1.88E-08	-0.025	0.019	0.190	31.615	6.84E-05	NA
rs3749262	3	131624097	G	A	0.263	-0.019	0.002	1.12E-14	0.008	0.015	0.590	59.681	1.29E-04	NA
rs3810291	19	47569003	G	A	0.337	-0.019	0.002	2.74E-17	-0.001	0.015	0.957	71.521	1.55E-04	NA
rs3814424	5	87968953	C	T	0.172	-0.025	0.003	2.55E-16	-0.002	0.019	0.896	67.124	1.45E-04	Intelligence
rs3814883	16	29994922	C	T	0.460	-0.026	0.002	7.26E-31	-0.017	0.014	0.220	133.436	2.89E-04	Nervous feelings
rs3852012	3	44045981	G	A	0.287	0.013	0.002	1.57E-08	-0.002	0.015	0.913	31.963	6.92E-05	NA
rs3902840	9	129419025	G	A	0.092	-0.021	0.004	1.72E-08	0.029	0.023	0.209	31.786	6.88E-05	NA
rs40067	5	107439012	G	A	0.180	0.018	0.003	4.05E-11	0.035	0.018	0.050	43.590	9.44E-05	NA
rs4074448	15	63888495	G	A	0.434	0.012	0.002	2.37E-08	0.011	0.014	0.438	31.161	6.75E-05	NA
rs4283409	2	228977142	A	G	0.387	-0.012	0.002	7.64E-09	-0.013	0.014	0.352	33.365	7.22E-05	NA
rs4377779	6	12117344	T	C	0.334	0.013	0.002	5.27E-09	0.029	0.014	0.039	34.086	7.38E-05	NA
rs4410790	7	17284577	T	C	0.385	-0.012	0.002	5.72E-09	-0.008	0.014	0.573	33.927	7.34E-05	NA
rs442177	4	88030261	G	T	0.421	-0.011	0.002	3.89E-08	-0.019	0.014	0.159	30.204	6.54E-05	NA
rs4477562	13	54104968	C	T	0.110	-0.020	0.003	9.80E-09	0.031	0.020	0.116	32.880	7.12E-05	NA
rs4482463	2	205375909	C	A	0.081	0.029	0.004	4.75E-12	0.011	0.026	0.659	47.787	1.03E-04	NA
rs4660208	1	39722214	A	G	0.195	-0.023	0.003	2.84E-18	-0.035	0.016	0.033	75.995	1.64E-04	NA
rs4665991	2	27766284	G	A	0.264	-0.013	0.002	2.16E-09	0.014	0.016	0.356	35.821	7.75E-05	NA
rs4693325	4	94408529	A	C	0.199	-0.016	0.003	1.74E-09	-0.018	0.018	0.312	36.241	7.85E-05	NA
rs4740619	9	15634326	T	C	0.481	0.014	0.002	6.33E-11	0.018	0.014	0.208	42.715	9.25E-05	NA
rs4777542	15	73082366	C	T	0.300	0.014	0.002	3.14E-09	0.001	0.015	0.933	35.098	7.60E-05	NA
rs4784745	16	57014875	A	G	0.329	-0.019	0.002	4.00E-21	-0.016	0.015	0.273	88.973	1.93E-04	NA
rs4790292	17	1824305	C	A	0.144	0.020	0.003	2.66E-10	0.006	0.019	0.768	39.913	8.64E-05	NA
rs479315	11	66064991	G	C	0.170	-0.016	0.003	2.08E-08	-0.021	0.018	0.233	31.420	6.80E-05	NA
rs4805758	19	32866424	G	A	0.162	0.020	0.003	5.55E-13	0.032	0.019	0.092	51.999	1.13E-04	Fed-up feelings
rs4810479	20	44545048	C	T	0.288	0.013	0.002	6.17E-10	-0.007	0.015	0.634	38.267	8.28E-05	NA
rs4889782	17	78640510	C	T	0.415	0.013	0.002	1.61E-09	0.030	0.015	0.046	36.391	7.88E-05	NA
rs4890439	18	40732586	G	A	0.375	0.012	0.002	4.60E-08	-0.001	0.014	0.948	29.879	6.47E-05	NA
rs4929923	11	8639200	T	C	0.377	-0.015	0.002	4.28E-11	-0.017	0.014	0.222	43.484	9.41E-05	NA
rs4947406	7	50661735	T	G	0.350	0.016	0.002	9.35E-12	-0.016	0.015	0.273	46.461	1.01E-04	NA
rs4971632	2	50240091	T	C	0.166	0.017	0.003	1.26E-09	0.000	0.018	0.980	36.870	7.98E-05	NA
rs512005	1	33377679	T	C	0.236	0.014	0.003	4.27E-08	0.020	0.017	0.232	30.024	6.50E-05	NA
rs545608	1	177899121	G	C	0.186	-0.038	0.003	1.86E-37	0.014	0.017	0.396	163.594	3.54E-04	NA
rs6115015	20	25027692	G	A	0.333	-0.013	0.002	6.30E-09	0.006	0.015	0.680	33.741	7.30E-05	NA
rs619054	11	116660813	G	A	0.232	0.015	0.002	2.70E-11	-0.018	0.016	0.256	44.384	9.61E-05	NA
rs645040	3	135926622	G	T	0.229	-0.023	0.002	1.31E-20	-0.022	0.016	0.172	86.621	1.87E-04	NA
rs6463489	7	5542513	T	C	0.069	0.024	0.004	1.03E-08	0.023	0.025	0.359	32.787	7.10E-05	NA
rs649458	1	23712528	T	A	0.137	0.018	0.003	5.66E-09	-0.008	0.020	0.703	33.949	7.35E-05	NA
rs6499240	16	69686912	A	G	0.422	-0.020	0.002	1.52E-18	0.001	0.014	0.928	77.230	1.67E-04	NA
rs6499861	16	56991495	C	G	0.162	-0.018	0.003	8.61E-12	0.007	0.019	0.702	46.623	1.01E-04	NA
rs6545714	2	59307725	G	A	0.382	0.019	0.002	3.15E-18	-0.004	0.014	0.747	75.796	1.64E-04	NA
rs6550597	3	18738940	G	A	0.298	-0.013	0.002	4.61E-09	-0.002	0.015	0.910	34.346	7.43E-05	NA
rs6575340	14	94023972	G	A	0.377	-0.014	0.002	8.82E-10	-0.009	0.014	0.510	37.571	8.13E-05	NA
rs6577584	1	6715390	T	G	0.372	-0.014	0.002	1.65E-10	-0.006	0.014	0.690	40.843	8.84E-05	NA
rs6602151	10	16764483	T	C	0.294	-0.015	0.002	5.89E-10	-0.005	0.015	0.731	38.356	8.30E-05	NA

rs6603004	15	84568504	A	G	0.466	-0.013	0.002	1.81E-09	0.007	0.013	0.600	36.163	7.83E-05	NA
rs6673977	1	7736097	A	G	0.354	-0.012	0.002	1.63E-08	0.001	0.014	0.946	31.892	6.90E-05	NA
rs6711375	2	161090873	G	A	0.320	-0.014	0.002	1.11E-09	-0.018	0.015	0.211	37.115	8.03E-05	NA
rs676387	17	40706273	C	A	0.266	-0.013	0.002	3.42E-08	-0.004	0.015	0.794	30.452	6.59E-05	NA
rs687672	11	65649984	T	C	0.189	0.021	0.003	5.92E-14	0.010	0.017	0.572	56.399	1.22E-04	NA
rs6907923	6	51802669	G	A	0.341	-0.015	0.002	1.71E-10	0.003	0.014	0.806	40.774	8.83E-05	NA
rs705986	8	64737682	A	G	0.491	0.012	0.002	2.53E-08	0.012	0.014	0.372	31.039	6.72E-05	NA
rs7075195	10	65050659	A	G	0.435	0.017	0.002	5.31E-16	0.024	0.014	0.078	65.676	1.42E-04	Educational attainment
rs7084454	10	21821274	G	A	0.338	-0.016	0.002	9.04E-13	-0.007	0.015	0.625	51.042	1.10E-04	NA
rs7161874	15	53163740	C	G	0.214	-0.015	0.003	4.11E-09	0.010	0.015	0.527	34.573	7.48E-05	NA
rs7198357	16	67884619	C	T	0.178	0.017	0.003	4.09E-11	-0.006	0.018	0.747	43.573	9.43E-05	NA
rs7206608	16	82872628	C	G	0.313	-0.013	0.002	1.26E-08	0.022	0.015	0.129	32.386	7.01E-05	NA
rs7241918	18	47160953	G	T	0.163	0.018	0.003	7.52E-13	0.010	0.018	0.586	51.403	1.11E-04	NA
rs7323827	13	94116675	A	G	0.304	0.013	0.002	4.59E-09	0.021	0.014	0.143	34.358	7.44E-05	NA
rs741959	1	47676233	A	G	0.402	0.014	0.002	6.19E-11	-0.012	0.014	0.365	42.760	9.26E-05	NA
rs7488867	12	103699685	C	T	0.281	0.015	0.003	1.57E-09	0.019	0.015	0.205	36.447	7.89E-05	NA
rs7515635	1	42408070	T	C	0.471	0.012	0.002	4.68E-09	0.008	0.014	0.532	34.317	7.43E-05	NA
rs7537581	1	2725475	C	A	0.476	-0.012	0.002	4.55E-08	-0.033	0.017	0.044	29.899	6.47E-05	NA
rs7549987	1	80789011	C	T	0.299	-0.014	0.002	4.02E-09	-0.006	0.016	0.692	34.614	7.49E-05	NA
rs7581217	2	25524944	T	C	0.370	0.013	0.002	5.93E-09	0.000	0.015	0.987	33.857	7.33E-05	NA
rs7601028	2	642499	C	G	0.174	-0.046	0.003	1.02E-52	-0.014	0.018	0.429	233.512	5.05E-04	NA
rs7668263	4	143781835	T	C	0.382	-0.012	0.002	4.21E-08	0.002	0.014	0.877	30.049	6.50E-05	NA
rs779655	5	170495158	G	C	0.272	-0.014	0.002	1.21E-08	0.007	0.015	0.620	32.464	7.03E-05	NA
rs7824519	8	14222796	T	G	0.324	-0.013	0.002	1.51E-08	-0.015	0.015	0.304	32.035	6.93E-05	NA
rs7928842	11	47566352	T	C	0.422	-0.020	0.002	2.30E-20	-0.012	0.014	0.375	85.511	1.85E-04	NA
rs7962636	12	116450006	T	C	0.355	0.013	0.002	1.32E-08	-0.017	0.018	0.332	32.308	6.99E-05	NA
rs7982447	13	54453811	T	C	0.200	-0.019	0.003	6.75E-13	-0.038	0.017	0.024	51.616	1.12E-04	NA
rs8015400	14	25930988	C	A	0.348	-0.018	0.002	8.13E-15	0.002	0.015	0.875	60.303	1.31E-04	NA
rs8055138	16	28891465	C	T	0.329	-0.030	0.002	1.86E-35	-0.023	0.014	0.095	154.432	3.34E-04	Intelligence
rs808820	12	89744826	A	G	0.116	-0.022	0.004	8.37E-10	0.005	0.022	0.829	37.671	8.15E-05	Educational attainment
rs818524	1	85201228	T	C	0.289	-0.013	0.002	2.84E-08	0.002	0.017	0.902	30.812	6.67E-05	NA
rs8192675	3	170724883	T	C	0.289	-0.013	0.002	4.30E-08	-0.027	0.015	0.067	30.007	6.50E-05	NA
rs891387	18	21103909	T	C	0.473	0.021	0.002	3.25E-21	0.026	0.014	0.053	89.383	1.93E-04	Educational attainment
rs9277972	6	33276770	A	T	0.168	-0.016	0.003	1.55E-08	0.000	0.018	0.991	31.995	6.93E-05	NA
rs9478499	6	154345289	T	A	0.147	-0.019	0.003	1.35E-10	0.003	0.018	0.886	41.238	8.93E-05	NA
rs9556979	13	99241507	T	G	0.324	-0.013	0.002	4.53E-09	0.003	0.016	0.839	34.382	7.44E-05	NA
rs9630985	2	181607676	A	C	0.349	-0.014	0.002	9.30E-11	-0.005	0.014	0.747	41.963	9.08E-05	NA
rs9686661	5	55861786	C	T	0.186	-0.023	0.003	4.65E-18	-0.005	0.018	0.759	75.025	1.62E-04	NA
rs9816226	3	185834499	A	T	0.188	-0.016	0.003	8.56E-09	0.026	0.018	0.137	33.144	7.17E-05	NA
rs9872156	3	176337348	G	A	0.260	-0.013	0.002	4.04E-08	-0.030	0.016	0.052	30.129	6.52E-05	NA
rs987237	6	50803050	A	G	0.181	-0.030	0.003	7.81E-26	0.019	0.017	0.277	110.449	2.39E-04	NA
rs9947450	18	58052665	C	T	0.030	0.062	0.007	1.22E-21	-0.001	0.038	0.973	91.321	1.98E-04	NA
rs998584	6	43757896	C	A	0.496	-0.013	0.002	9.99E-11	-0.020	0.014	0.157	41.823	9.05E-05	NA
rs9987289	8	9183358	A	G	0.075	0.022	0.004	1.84E-09	-0.007	0.023	0.750	36.136	7.82E-05	NA

Genomic positions reported in the GWAS of Mets refer to human reference assembly (GRCh37/hg19).

Abbreviations:MetS, Metabolic syndrome; ANO, Anorexia Nervosa; SNPs, Single nucleotide polymorphisms; CHR, Chromosome; EA, Effect allele; OA, Other allele; EAF, Effect allele frequency; SE, Standard error; PVE, Proportion of phenotypic variance explained.

SNPs that are unavailable in the outcome GWAS: rs6754311,rs17207196.

Palindromic and not-inferrable SNPs that are abandoned as instruments: rs1937433, rs2253310, rs6916318.

8 SNPs detected by PRESSO outlier test, and 44 SNPs were detected by MR-Radial outlier test.

Supplementary Table 5. Genetic instruments for the causal relationship of Mets and ANX.

SNP	CHR	Position	EA	OA	EAF	Beta for exposure	SE for exposure	P value for exposure	Beta for outcome	SE for outcome	P value for outcome	F statistics	PVE	Association with potential confounding factors
rs10116353	9	111950088	G	T	0.307	0.013	0.002	9.39E-09	-0.015	0.027	0.581	32.964	7.14E-05	NA
rs1013402	11	27712381	A	G	0.290	-0.025	0.002	1.34E-23	-0.006	0.026	0.817	100.255	2.17E-04	NA
rs102275	11	61557803	T	C	0.363	-0.013	0.002	6.28E-11	-0.019	0.026	0.462	42.731	9.25E-05	NA
rs1032524	1	201790826	T	C	0.484	-0.015	0.002	6.01E-12	-0.050	0.025	0.043	47.325	1.02E-04	NA
rs10423928	19	46182304	T	A	0.214	0.020	0.003	8.49E-13	0.028	0.029	0.336	51.166	1.11E-04	NA
rs10734924	12	123018894	T	G	0.259	-0.015	0.003	2.19E-09	-0.011	0.028	0.692	35.801	7.75E-05	NA
rs10768994	11	43936945	T	C	0.430	0.012	0.002	4.52E-08	0.009	0.025	0.705	29.912	6.48E-05	NA
rs10779835	1	230299949	T	C	0.391	0.015	0.002	2.45E-14	0.021	0.025	0.407	58.131	1.26E-04	NA
rs10783779	12	56491880	T	G	0.403	0.013	0.002	3.84E-09	0.006	0.025	0.808	34.704	7.51E-05	NA
rs10808546	8	126495818	C	T	0.431	0.016	0.002	4.56E-19	0.010	0.025	0.698	79.612	1.72E-04	NA
rs10872224	6	98435125	G	T	0.409	-0.014	0.002	9.76E-11	-0.004	0.025	0.886	41.868	9.06E-05	Educational attainment; Intelligence
rs10887579	10	88097210	A	C	0.354	-0.013	0.002	4.09E-09	0.041	0.026	0.118	34.579	7.49E-05	NA
rs10930502	2	172890588	A	G	0.304	0.013	0.002	1.18E-08	-0.007	0.026	0.789	32.519	7.04E-05	NA
rs10938397	4	45182527	A	G	0.420	-0.023	0.002	8.14E-26	0.012	0.025	0.634	110.367	2.39E-04	NA
rs11072938	15	81021661	C	T	0.241	0.016	0.002	4.30E-11	0.050	0.027	0.067	43.470	9.41E-05	NA
rs11079849	17	47090785	C	T	0.290	0.015	0.002	6.90E-11	-0.041	0.027	0.128	42.547	9.21E-05	NA
rs11150604	16	31037020	A	T	0.389	0.024	0.002	3.91E-28	0.038	0.025	0.125	120.954	2.62E-04	NA
rs11165643	1	96924097	C	T	0.417	-0.013	0.002	6.90E-10	0.028	0.025	0.264	38.049	8.24E-05	NA
rs11187845	10	96060198	C	A	0.096	0.020	0.004	8.08E-09	0.027	0.047	0.568	33.256	7.20E-05	NA
rs1138429	16	56942921	A	T	0.101	-0.028	0.003	1.42E-18	0.011	0.046	0.804	77.365	1.67E-04	NA
rs1159974	6	126090277	T	C	0.499	-0.013	0.002	3.30E-10	0.033	0.025	0.176	39.489	8.55E-05	NA
rs11611246	12	939480	G	T	0.183	-0.016	0.003	1.14E-08	-0.012	0.030	0.692	32.583	7.05E-05	NA
rs11631408	15	51746236	A	G	0.463	0.014	0.002	2.44E-11	-0.008	0.025	0.756	44.582	9.65E-05	NA
rs11636565	15	36391846	C	A	0.488	-0.014	0.002	6.84E-12	-0.024	0.024	0.313	47.072	1.02E-04	NA
rs11660335	18	22154235	T	C	0.161	0.018	0.003	1.07E-09	-0.012	0.030	0.690	37.189	8.05E-05	NA
rs11676272	2	25141538	A	G	0.464	-0.020	0.002	1.05E-17	-0.047	0.024	0.055	73.421	1.59E-04	NA
rs11747810	5	153100273	T	C	0.495	0.013	0.002	1.83E-09	0.011	0.025	0.669	36.148	7.83E-05	NA
rs11766944	7	1888051	G	A	0.214	0.015	0.003	4.17E-08	0.023	0.031	0.451	30.068	6.51E-05	NA
rs11826999	11	116553778	G	A	0.223	-0.023	0.002	6.07E-26	0.041	0.029	0.153	110.950	2.40E-04	NA
rs11866815	16	387867	C	T	0.242	0.016	0.002	2.32E-10	0.020	0.030	0.491	40.181	8.70E-05	NA
rs11880870	19	18830704	A	G	0.499	0.013	0.002	2.98E-09	0.008	0.024	0.742	35.198	7.62E-05	NA
rs11981190	7	71659538	G	A	0.341	0.013	0.002	5.84E-09	-0.026	0.026	0.316	33.887	7.34E-05	NA
rs1200349	15	41821752	T	C	0.482	-0.013	0.002	1.36E-09	-0.030	0.024	0.220	36.724	7.95E-05	NA
rs12033257	1	112318484	A	G	0.382	0.014	0.002	8.97E-10	0.005	0.028	0.852	37.538	8.13E-05	NA
rs12201987	6	143182396	C	T	0.205	0.016	0.003	1.87E-09	-0.004	0.031	0.901	36.103	7.82E-05	NA
rs12446554	16	19935073	G	T	0.130	0.020	0.003	3.79E-10	-0.028	0.037	0.439	39.220	8.49E-05	NA
rs12513212	4	96149346	A	C	0.387	-0.014	0.002	2.85E-10	0.004	0.025	0.878	39.778	8.61E-05	NA
rs12520822	5	105699867	C	T	0.408	0.012	0.002	1.84E-08	-0.016	0.024	0.507	31.658	6.85E-05	NA
rs12596491	16	88928417	C	T	0.340	0.012	0.002	4.58E-08	0.061	0.030	0.045	29.888	6.47E-05	NA
rs12608504	19	18389135	A	G	0.359	0.016	0.002	2.27E-13	0.006	0.026	0.826	53.757	1.16E-04	NA
rs12624640	20	32952125	G	A	0.430	-0.012	0.002	7.17E-09	0.012	0.025	0.621	33.489	7.25E-05	NA
rs12824567	12	124495203	G	C	0.350	0.013	0.002	1.94E-09	-0.014	0.026	0.589	36.032	7.80E-05	NA
rs12967135	18	57849023	G	A	0.240	-0.046	0.003	3.09E-66	0.040	0.029	0.157	295.539	6.39E-04	Worrier or anxious feelings
rs12981256	19	1865901	G	A	0.475	-0.016	0.002	7.75E-14	-0.032	0.027	0.241	55.867	1.21E-04	NA
rs13024601	2	100902032	C	T	0.346	0.014	0.002	1.39E-09	-0.033	0.025	0.195	36.680	7.94E-05	NA
rs13029936	2	208465544	C	T	0.176	0.017	0.003	1.31E-09	-0.052	0.030	0.084	36.795	7.96E-05	NA
rs13107325	4	103188709	C	T	0.080	-0.023	0.004	1.94E-09	0.003	0.058	0.955	36.033	7.80E-05	Intelligence
rs13206549	6	162978032	T	G	0.134	0.019	0.003	1.54E-09	0.004	0.037	0.916	36.486	7.90E-05	NA
rs13244268	7	72911843	T	C	0.114	0.024	0.003	9.17E-18	0.027	0.037	0.469	73.684	1.59E-04	NA

rs13250456	8	72408720	C	T	0.055	-0.025	0.005	4.67E-08	-0.019	0.064	0.771	29.850	6.46E-05		NA
rs13275593	8	68257300	G	A	0.285	-0.015	0.002	2.14E-10	0.018	0.028	0.512	40.332	8.73E-05		NA
rs1330304	9	16714629	C	G	0.267	0.013	0.002	2.32E-08	0.044	0.028	0.118	31.202	6.75E-05		NA
rs13416004	2	69664976	C	T	0.415	0.012	0.002	3.16E-08	0.019	0.025	0.433	30.605	6.63E-05		NA
rs13416667	2	56608267	C	A	0.274	-0.015	0.002	2.32E-10	0.001	0.029	0.987	40.179	8.70E-05		NA
rs13435860	4	136975545	A	T	0.368	-0.013	0.002	1.94E-09	0.044	0.026	0.084	36.037	7.80E-05		NA
rs1353580	15	47738732	T	A	0.374	-0.012	0.002	1.70E-08	-0.003	0.025	0.904	31.810	6.89E-05		NA
rs1362910	8	30856464	A	G	0.386	0.014	0.002	9.70E-11	0.039	0.025	0.111	41.882	9.07E-05		NA
rs1363695	5	130378027	C	T	0.228	0.015	0.003	6.49E-09	0.043	0.029	0.140	33.681	7.29E-05		NA
rs1412239	9	28425515	C	G	0.304	-0.022	0.002	2.39E-21	0.007	0.027	0.786	89.993	1.95E-04		NA
rs1421085	16	53800954	T	C	0.432	-0.062	0.002	5.07E-152	-0.018	0.025	0.474	689.747	1.49E-03		NA
rs1431659	8	73439070	A	G	0.278	0.019	0.002	4.48E-15	0.019	0.028	0.481	61.476	1.33E-04		NA
rs1448613	3	85875265	G	A	0.386	0.014	0.002	2.40E-10	0.016	0.026	0.538	40.110	8.68E-05		NA
rs1452075	3	62481063	C	T	0.284	-0.014	0.002	9.96E-09	-0.016	0.027	0.563	32.850	7.11E-05		NA
rs1456116	3	123084379	G	A	0.417	-0.013	0.002	1.99E-09	0.022	0.024	0.358	35.987	7.79E-05		NA
rs1458156	12	41887940	C	T	0.449	-0.013	0.002	4.45E-09	0.006	0.024	0.794	34.416	7.45E-05		NA
rs1460940	1	72814617	G	A	0.182	-0.020	0.003	2.89E-13	-0.027	0.032	0.404	53.284	1.15E-04		NA
rs1507153	6	79484386	C	A	0.364	-0.013	0.002	3.86E-09	-0.017	0.025	0.498	34.694	7.51E-05		NA
rs1513475	3	35724256	T	C	0.343	0.015	0.002	8.46E-11	0.032	0.026	0.223	42.149	9.12E-05		NA
rs1534696	7	26397239	C	A	0.439	0.014	0.002	1.24E-11	0.037	0.026	0.154	45.905	9.94E-05		NA
rs1580099	3	94003603	C	A	0.406	0.014	0.002	4.35E-09	0.023	0.025	0.356	34.459	7.46E-05		NA
rs17024393	1	110154688	T	C	0.033	-0.048	0.006	1.96E-15	-0.027	0.067	0.682	63.106	1.37E-04		NA
rs17162333	1	27236757	C	G	0.132	-0.020	0.003	6.24E-11	0.038	0.034	0.262	42.744	9.25E-05		NA
rs17207196	7	75101065	C	T	0.444	0.018	0.002	2.68E-16	0.035	0.026	0.176	67.024	1.45E-04		NA
rs1730858	1	107619244	T	C	0.328	0.015	0.002	1.04E-10	0.021	0.026	0.427	41.736	9.03E-05		NA
rs17391694	1	78623626	C	T	0.096	-0.026	0.004	2.39E-12	-0.026	0.035	0.447	49.132	1.06E-04		NA
rs17618704	17	46221010	T	G	0.068	0.025	0.004	1.52E-09	-0.075	0.046	0.105	36.510	7.90E-05		NA
rs17639546	3	61251635	G	A	0.148	0.018	0.003	3.65E-09	-0.043	0.033	0.200	34.803	7.53E-05		NA
rs17694506	2	171632225	C	T	0.388	0.014	0.002	3.33E-11	0.014	0.025	0.587	43.971	9.52E-05		NA
rs17724992	19	18454825	A	G	0.245	0.014	0.002	8.13E-09	0.031	0.029	0.271	33.243	7.20E-05		NA
rs17783751	16	89513984	C	A	0.176	-0.015	0.003	4.79E-08	-0.008	0.031	0.789	29.800	6.45E-05		NA
rs17821316	15	58703069	A	C	0.488	-0.012	0.002	9.97E-10	0.037	0.027	0.175	37.331	8.08E-05		NA
rs1784460	11	118938371	T	A	0.360	-0.015	0.002	8.15E-12	-0.048	0.025	0.054	46.729	1.01E-04		NA
rs1886558	13	111982615	A	G	0.389	0.012	0.002	4.14E-08	0.016	0.026	0.545	30.082	6.51E-05		NA
rs1899689	7	121964349	C	T	0.380	-0.012	0.002	4.27E-08	-0.015	0.025	0.567	30.021	6.50E-05		NA
rs1914888	17	21267541	A	G	0.462	0.013	0.002	1.33E-09	-0.011	0.026	0.667	36.762	7.96E-05		NA
rs192812	3	161491831	C	T	0.454	-0.012	0.002	4.87E-09	-0.009	0.025	0.714	34.241	7.41E-05		NA
rs1945941	11	132620118	G	A	0.376	0.012	0.002	3.63E-08	0.041	0.025	0.100	30.337	6.57E-05		NA
rs2002023	10	76848524	C	T	0.441	-0.012	0.002	8.92E-09	-0.019	0.025	0.437	33.064	7.16E-05		NA
rs2015407	14	103274547	A	G	0.345	0.014	0.002	4.68E-10	0.014	0.026	0.587	38.808	8.40E-05		NA
rs2035831	3	173683791	G	C	0.321	0.013	0.002	1.48E-08	-0.031	0.026	0.222	32.085	6.95E-05		NA
rs2065418	11	30422068	T	G	0.354	0.016	0.002	1.39E-12	0.012	0.025	0.623	50.196	1.09E-04		NA
rs2066295	6	26168903	A	G	0.219	0.023	0.003	5.73E-18	-0.038	0.029	0.186	74.611	1.61E-04		NA
rs2074881	19	1970021	C	T	0.179	0.015	0.003	2.25E-08	0.007	0.035	0.853	31.270	6.77E-05		NA
rs2075226	2	36805676	A	G	0.396	-0.013	0.002	2.48E-08	-0.008	0.026	0.769	31.073	6.73E-05		NA
rs208015	17	46252346	T	C	0.088	0.022	0.004	1.07E-08	0.052	0.049	0.294	32.711	7.08E-05		NA
rs2143253	20	41987392	G	A	0.137	0.019	0.003	1.29E-09	0.067	0.041	0.097	36.823	7.97E-05		NA
rs2180454	14	29690513	T	C	0.241	-0.015	0.003	1.90E-09	-0.023	0.028	0.414	36.071	7.81E-05		NA
rs2237717	7	116405387	T	C	0.443	-0.011	0.002	2.63E-08	0.028	0.025	0.264	30.960	6.70E-05		NA
rs2242449	17	7095507	C	T	0.417	-0.013	0.002	2.85E-09	0.007	0.025	0.779	35.287	7.64E-05		NA
rs227199	1	210265384	G	C	0.316	0.013	0.002	5.06E-09	0.005	0.026	0.845	34.168	7.40E-05		NA

rs2272168	7	99102777	G	C	0.163	0.017	0.003	3.68E-09	0.027	0.036	0.448	34.785	7.53E-05	NA
rs2275543	9	107651174	T	C	0.099	-0.019	0.003	6.58E-09	-0.013	0.042	0.750	33.655	7.29E-05	NA
rs2298969	4	3186244	A	G	0.490	0.012	0.002	2.79E-08	0.001	0.025	0.974	30.851	6.68E-05	NA
rs2299383	7	103418846	C	T	0.410	-0.014	0.002	2.20E-10	0.022	0.024	0.363	40.282	8.72E-05	NA
rs2307111	5	75003678	T	C	0.396	0.025	0.002	4.41E-29	-0.021	0.025	0.391	125.285	2.71E-04	NA
rs2396625	7	113028634	T	A	0.401	0.014	0.002	1.99E-10	-0.041	0.025	0.096	40.473	8.76E-05	NA
rs2436726	6	40366427	C	T	0.405	-0.016	0.002	2.24E-12	0.006	0.024	0.795	49.262	1.07E-04	NA
rs2453763	5	92376460	T	A	0.347	-0.012	0.002	3.72E-08	0.003	0.025	0.903	30.290	6.56E-05	NA
rs2470945	7	104585760	C	A	0.408	-0.012	0.002	2.10E-08	0.020	0.025	0.416	31.396	6.80E-05	NA
rs2526754	3	50125996	G	A	0.494	0.022	0.002	5.98E-22	0.022	0.024	0.366	92.735	2.01E-04	Educational attainment; Intelligence
rs2531686	10	118651011	C	G	0.276	0.015	0.002	2.22E-10	-0.019	0.028	0.487	40.261	8.72E-05	NA
rs2531995	16	4013467	C	T	0.387	-0.019	0.002	2.80E-17	0.002	0.028	0.956	71.477	1.55E-04	NA
rs2596933	3	9365746	G	A	0.267	0.014	0.002	1.26E-08	0.006	0.030	0.851	32.396	7.01E-05	NA
rs261336	15	58742418	G	A	0.161	-0.020	0.003	2.07E-14	0.001	0.034	0.989	58.461	1.27E-04	NA
rs2781668	6	131897278	C	T	0.170	-0.015	0.003	3.64E-08	0.021	0.034	0.544	30.331	6.57E-05	NA
rs2787693	1	49915848	C	A	0.302	0.017	0.002	1.94E-11	-0.002	0.026	0.936	45.027	9.75E-05	NA
rs2814944	6	34552797	G	A	0.137	-0.034	0.003	1.03E-26	-0.011	0.032	0.726	114.464	2.48E-04	NA
rs2820232	6	35003603	G	T	0.459	-0.012	0.002	2.95E-08	0.008	0.025	0.749	30.741	6.65E-05	NA
rs2836753	21	40291187	T	C	0.385	-0.013	0.002	7.29E-10	0.015	0.025	0.568	37.941	8.21E-05	NA
rs2861688	2	67838474	T	C	0.273	0.016	0.002	8.97E-11	-0.013	0.028	0.642	42.033	9.10E-05	NA
rs2925979	16	81534790	T	C	0.288	0.014	0.002	1.10E-10	0.000	0.026	0.987	41.628	9.01E-05	NA
rs2968856	7	150614934	T	C	0.261	0.016	0.002	1.80E-11	-0.011	0.029	0.694	45.179	9.78E-05	NA
rs2990997	1	190119703	T	A	0.353	-0.012	0.002	3.24E-08	0.011	0.026	0.681	30.558	6.62E-05	NA
rs320	8	19819077	T	G	0.287	0.031	0.002	2.30E-43	0.015	0.028	0.583	190.642	4.13E-04	NA
rs323781	5	86752616	A	C	0.034	-0.032	0.006	3.82E-08	-0.082	0.077	0.288	30.237	6.55E-05	NA
rs349088	11	84814393	C	A	0.480	0.013	0.002	3.23E-09	0.006	0.025	0.812	35.040	7.59E-05	NA
rs350832	19	4069426	G	A	0.227	-0.014	0.003	1.88E-08	0.015	0.031	0.633	31.615	6.84E-05	NA
rs3808436	8	116560967	A	G	0.432	0.016	0.002	1.58E-13	0.011	0.025	0.655	54.468	1.18E-04	NA
rs3810291	19	47569003	G	A	0.337	-0.019	0.002	2.74E-17	0.015	0.026	0.572	71.521	1.55E-04	NA
rs3814424	5	87968953	C	T	0.172	-0.025	0.003	2.55E-16	-0.061	0.034	0.072	67.124	1.45E-04	Intelligence
rs3814883	16	29994922	C	T	0.460	-0.026	0.002	7.26E-31	0.021	0.024	0.397	133.436	2.89E-04	Nervous feelings
rs3852012	3	44045981	G	A	0.287	0.013	0.002	1.57E-08	-0.022	0.026	0.388	31.963	6.92E-05	NA
rs3864004	3	41240177	G	A	0.432	-0.014	0.002	2.30E-09	0.015	0.024	0.533	35.700	7.73E-05	NA
rs3902840	9	129419025	G	A	0.092	-0.021	0.004	1.72E-08	-0.004	0.042	0.933	31.786	6.88E-05	NA
rs397092	21	46582564	A	G	0.456	-0.013	0.002	1.07E-09	-0.012	0.025	0.635	37.196	8.05E-05	NA
rs40067	5	107439012	G	A	0.180	0.018	0.003	4.05E-11	0.015	0.033	0.657	43.590	9.44E-05	NA
rs4074448	15	63888495	G	A	0.434	0.012	0.002	2.37E-08	-0.019	0.026	0.461	31.161	6.75E-05	NA
rs4283409	2	228977142	A	G	0.387	-0.012	0.002	7.64E-09	0.008	0.025	0.766	33.365	7.22E-05	NA
rs429343	2	147903382	A	G	0.437	0.012	0.002	1.26E-08	0.000	0.025	0.995	32.394	7.01E-05	NA
rs4377779	6	12117344	T	C	0.334	0.013	0.002	5.27E-09	-0.017	0.025	0.507	34.086	7.38E-05	NA
rs4410790	7	17284577	T	C	0.385	-0.012	0.002	5.72E-09	-0.025	0.026	0.334	33.927	7.34E-05	NA
rs442177	4	88030261	G	T	0.421	-0.011	0.002	3.89E-08	-0.007	0.025	0.769	30.204	6.54E-05	NA
rs4477562	13	54104968	C	T	0.110	-0.020	0.003	9.80E-09	0.004	0.036	0.906	32.880	7.12E-05	NA
rs4482463	2	205375909	C	A	0.081	0.029	0.004	4.75E-12	-0.002	0.045	0.967	47.787	1.03E-04	NA
rs4660208	1	39722214	A	G	0.195	-0.023	0.003	2.84E-18	0.010	0.029	0.743	75.995	1.64E-04	NA
rs4665991	2	27766284	G	A	0.264	-0.013	0.002	2.16E-09	0.027	0.029	0.355	35.821	7.75E-05	NA
rs4671328	2	58935282	T	G	0.430	0.018	0.002	9.13E-17	-0.004	0.025	0.865	69.149	1.50E-04	NA
rs4693325	4	94408529	A	C	0.199	-0.016	0.003	1.74E-09	0.049	0.032	0.123	36.241	7.85E-05	NA
rs4740619	9	15634326	T	C	0.481	0.014	0.002	6.33E-11	-0.019	0.025	0.447	42.715	9.25E-05	NA
rs4755720	11	43628749	C	T	0.400	0.016	0.002	4.50E-14	0.032	0.025	0.206	56.935	1.23E-04	NA
rs4777542	15	73082366	C	T	0.300	0.014	0.002	3.14E-09	0.051	0.027	0.058	35.098	7.60E-05	NA

rs4784745	16	57014875	A	G	0.329	-0.019	0.002	4.00E-21	0.021	0.026	0.410	88.973	1.93E-04	NA
rs4790292	17	1824305	C	A	0.144	0.020	0.003	2.66E-10	0.011	0.033	0.742	39.913	8.64E-05	NA
rs479315	11	66064991	G	C	0.170	-0.016	0.003	2.08E-08	0.052	0.032	0.099	31.420	6.80E-05	NA
rs4805758	19	32866424	G	A	0.162	0.020	0.003	5.55E-13	-0.039	0.035	0.263	51.999	1.13E-04	Fed-up feelings
rs4889782	17	78640510	C	T	0.415	0.013	0.002	1.61E-09	-0.008	0.026	0.759	36.391	7.88E-05	NA
rs4890439	18	40732586	G	A	0.375	0.012	0.002	4.60E-08	-0.007	0.025	0.772	29.879	6.47E-05	NA
rs4929923	11	8639200	T	C	0.377	-0.015	0.002	4.28E-11	-0.002	0.025	0.951	43.484	9.41E-05	NA
rs4947406	7	50661735	T	G	0.350	0.016	0.002	9.35E-12	0.009	0.026	0.737	46.461	1.01E-04	NA
rs512005	1	33377679	T	C	0.236	0.014	0.003	4.27E-08	0.009	0.029	0.766	30.024	6.50E-05	NA
rs545608	1	177899121	G	C	0.186	-0.038	0.003	1.86E-37	-0.006	0.030	0.830	163.594	3.54E-04	NA
rs563296	10	99772404	G	A	0.441	-0.013	0.002	1.23E-08	-0.005	0.025	0.857	32.438	7.02E-05	NA
rs6115015	20	25027692	G	A	0.333	-0.013	0.002	6.30E-09	0.003	0.026	0.919	33.741	7.30E-05	NA
rs619054	11	116660813	G	A	0.232	0.015	0.002	2.70E-11	0.020	0.030	0.504	44.384	9.61E-05	NA
rs645040	3	135926622	G	T	0.229	-0.023	0.002	1.31E-20	-0.010	0.030	0.734	86.621	1.87E-04	NA
rs6463489	7	5542513	T	C	0.069	0.024	0.004	1.03E-08	-0.010	0.042	0.818	32.787	7.10E-05	NA
rs649458	1	23712528	T	A	0.137	0.018	0.003	5.66E-09	-0.006	0.038	0.872	33.949	7.35E-05	NA
rs6499240	16	69686912	A	G	0.422	-0.020	0.002	1.52E-18	-0.037	0.025	0.127	77.230	1.67E-04	NA
rs6499861	16	56991495	C	G	0.162	-0.018	0.003	8.61E-12	-0.010	0.033	0.772	46.623	1.01E-04	NA
rs6548834	3	82704753	G	A	0.378	-0.013	0.002	1.08E-08	0.004	0.025	0.874	32.694	7.08E-05	NA
rs6550597	3	18738940	G	A	0.298	-0.013	0.002	4.61E-09	0.016	0.028	0.552	34.346	7.43E-05	NA
rs6575340	14	94023972	G	A	0.377	-0.014	0.002	8.82E-10	-0.012	0.026	0.634	37.571	8.13E-05	NA
rs6577584	1	6715390	T	G	0.372	-0.014	0.002	1.65E-10	-0.045	0.026	0.075	40.843	8.84E-05	NA
rs6602151	10	16764483	T	C	0.294	-0.015	0.002	5.89E-10	0.017	0.027	0.531	38.356	8.30E-05	NA
rs6603004	15	84568504	A	G	0.466	-0.013	0.002	1.81E-09	-0.029	0.024	0.234	36.163	7.83E-05	NA
rs6673977	1	7736097	A	G	0.354	-0.013	0.002	1.63E-08	-0.002	0.025	0.926	31.892	6.90E-05	NA
rs6754311	2	136707982	T	C	0.489	0.013	0.002	3.95E-10	0.004	0.031	0.886	39.137	8.47E-05	NA
rs676387	17	40706273	C	A	0.266	-0.013	0.002	3.42E-08	-0.022	0.027	0.421	30.452	6.59E-05	NA
rs6868198	5	122750714	G	A	0.432	0.013	0.002	2.10E-08	-0.031	0.025	0.211	31.399	6.80E-05	NA
rs6870983	5	87697533	C	T	0.236	0.020	0.003	1.66E-15	-0.051	0.030	0.087	63.434	1.37E-04	NA
rs687672	11	65649984	T	C	0.189	0.021	0.003	5.92E-14	0.026	0.031	0.400	56.399	1.22E-04	NA
rs6907923	6	51802669	G	A	0.341	-0.015	0.002	1.71E-10	0.003	0.025	0.914	40.774	8.83E-05	NA
rs7016529	8	19806631	T	C	0.011	-0.102	0.009	1.94E-27	-0.163	0.101	0.106	117.778	2.55E-04	NA
rs703965	10	80955067	C	T	0.456	-0.012	0.002	8.25E-09	0.002	0.025	0.929	33.214	7.19E-05	NA
rs705986	8	64737682	A	G	0.491	0.012	0.002	2.53E-08	-0.029	0.025	0.245	31.039	6.72E-05	NA
rs7075195	10	65050659	A	G	0.435	0.017	0.002	5.31E-16	-0.009	0.025	0.717	65.676	1.42E-04	Educational attainment
rs7084454	10	21821274	G	A	0.338	-0.016	0.002	9.04E-13	0.013	0.026	0.614	51.042	1.10E-04	NA
rs7132908	12	50263148	G	A	0.357	-0.019	0.002	9.07E-15	-0.013	0.025	0.590	60.088	1.30E-04	NA
rs7144011	14	79940383	G	T	0.207	-0.023	0.003	1.71E-17	-0.033	0.030	0.271	72.451	1.57E-04	NA
rs7161874	15	53163740	C	G	0.214	-0.015	0.003	4.11E-09	0.024	0.028	0.395	34.573	7.48E-05	NA
rs7186893	16	24806420	G	T	0.267	0.020	0.002	3.04E-16	0.015	0.028	0.597	66.779	1.45E-04	Mood swings
rs7198357	16	67884619	C	T	0.178	0.017	0.003	4.09E-11	0.031	0.032	0.334	43.573	9.43E-05	NA
rs7206608	16	82872628	C	G	0.313	-0.013	0.002	1.26E-08	0.006	0.026	0.817	32.386	7.01E-05	NA
rs7212293	17	65847325	A	G	0.216	-0.022	0.003	4.05E-17	-0.062	0.030	0.035	70.753	1.53E-04	NA
rs7241918	18	47160953	G	T	0.163	0.018	0.003	7.52E-13	0.017	0.032	0.599	51.404	1.11E-04	NA
rs7323827	13	94116675	A	G	0.304	0.013	0.002	4.59E-09	0.018	0.026	0.491	34.358	7.44E-05	NA
rs741959	1	47676233	A	G	0.402	0.014	0.002	6.19E-11	0.020	0.025	0.428	42.760	9.26E-05	NA
rs7488867	12	103699685	C	T	0.281	0.015	0.003	1.57E-09	0.000	0.028	0.999	36.447	7.89E-05	NA
rs7515635	1	42408070	T	C	0.471	0.012	0.002	4.68E-09	0.034	0.024	0.161	34.317	7.43E-05	NA
rs7537581	1	2725475	C	A	0.476	-0.012	0.002	4.55E-08	0.007	0.026	0.790	29.899	6.47E-05	NA
rs7549987	1	80789011	C	T	0.299	-0.014	0.002	4.02E-09	-0.024	0.027	0.365	34.614	7.49E-05	NA
rs7581217	2	25524944	T	C	0.370	0.013	0.002	5.93E-09	0.012	0.028	0.677	33.857	7.33E-05	NA

rs7601028	2	642499	C	G	0.174	-0.046	0.003	1.02E-52	0.010	0.033	0.773	233.512	5.05E-04	NA
rs7638388	3	47914974	C	T	0.088	-0.024	0.004	1.01E-10	0.002	0.046	0.961	41.794	9.05E-05	NA
rs7642876	3	15883094	A	G	0.404	0.012	0.002	1.35E-08	-0.003	0.025	0.911	32.265	6.98E-05	NA
rs7668263	4	143781835	T	C	0.382	-0.012	0.002	4.21E-08	0.028	0.026	0.280	30.049	6.50E-05	NA
rs779655	5	170495158	G	C	0.272	-0.014	0.002	1.21E-08	-0.003	0.027	0.923	32.464	7.03E-05	NA
rs7824519	8	14222796	T	G	0.324	-0.013	0.002	1.51E-08	-0.001	0.026	0.980	32.035	6.93E-05	NA
rs7928842	11	47566352	T	C	0.422	-0.020	0.002	2.30E-20	0.015	0.025	0.544	85.511	1.85E-04	NA
rs7947951	11	13356030	A	G	0.297	-0.014	0.002	1.59E-10	-0.007	0.026	0.794	40.911	8.86E-05	NA
rs7962636	12	116450006	T	C	0.355	0.013	0.002	1.32E-08	0.004	0.028	0.876	32.308	6.99E-05	NA
rs7982447	13	54453811	T	C	0.200	-0.019	0.003	6.75E-13	0.016	0.030	0.600	51.616	1.12E-04	NA
rs8015400	14	25930988	C	A	0.348	-0.018	0.002	8.13E-15	-0.029	0.027	0.274	60.303	1.31E-04	NA
rs8055138	16	28891465	C	T	0.329	-0.030	0.002	1.86E-35	0.004	0.025	0.872	154.432	3.34E-04	Educational attainment; Intelligence
rs8078079	17	34959346	T	C	0.395	0.015	0.002	2.89E-11	0.012	0.025	0.639	44.247	9.58E-05	NA
rs818524	1	85201228	T	C	0.289	-0.013	0.002	2.84E-08	-0.009	0.029	0.748	30.812	6.67E-05	NA
rs8192675	3	170724883	T	C	0.289	-0.013	0.002	4.30E-08	0.033	0.026	0.209	30.007	6.50E-05	NA
rs891387	18	21103909	T	C	0.473	0.021	0.002	3.25E-21	0.044	0.024	0.070	89.383	1.93E-04	Educational attainment
rs906673	3	12898941	C	T	0.389	0.013	0.002	3.67E-09	-0.003	0.025	0.904	34.789	7.53E-05	NA
rs9277972	6	33276770	A	T	0.168	-0.016	0.003	1.55E-08	-0.032	0.033	0.337	31.995	6.93E-05	NA
rs938875	15	67952922	A	G	0.408	-0.019	0.002	3.72E-18	0.001	0.025	0.956	75.463	1.63E-04	NA
rs9563569	13	58571207	G	A	0.150	0.018	0.003	2.09E-08	0.000	0.031	0.989	31.408	6.80E-05	NA
rs9630985	2	181607676	A	C	0.349	-0.014	0.002	9.30E-11	0.003	0.026	0.915	41.963	9.08E-05	NA
rs9686661	5	55861786	C	T	0.186	-0.023	0.003	4.65E-18	0.026	0.033	0.430	75.025	1.62E-04	NA
rs9816226	3	185834499	A	T	0.188	-0.016	0.003	8.56E-09	-0.044	0.033	0.186	33.144	7.17E-05	NA
rs9872156	3	176337348	G	A	0.260	-0.013	0.002	4.04E-08	0.025	0.027	0.362	30.129	6.52E-05	NA
rs987237	6	50803050	A	G	0.181	-0.030	0.003	7.81E-26	0.017	0.032	0.607	110.449	2.39E-04	NA
rs9947450	18	58052665	C	T	0.030	0.062	0.007	1.22E-21	0.006	0.071	0.932	91.321	1.98E-04	NA
rs998584	6	43757896	C	A	0.496	-0.013	0.002	9.99E-11	0.018	0.024	0.451	41.823	9.05E-05	NA
rs9987289	8	9183358	A	G	0.075	0.022	0.004	1.84E-09	0.020	0.042	0.641	36.136	7.82E-05	NA

Genomic positions reported in the GWAS of Mets refer to human reference assembly (GRCh37/hg19).

Abbreviations: MetS, Metabolic syndrome; ANX, Anxiety; SNPs, Single nucleotide polymorphisms; CHR, Chromosome; EA, Effect allele; OA, Other allele; EAF, Effect allele frequency; SE, Standard error; PVE, Proportion of phenotypic variance explained.

SNPs that are unavailable in the outcome GWAS: rs1046080, rs705986, rs1412239, rs2516740, among them, rs705986 and rs1412239 were substitute by proxy SNPs: rs2685563 and rs1412235, respectively.

3 SNPs were detected by PRESSO outlier test, and 17 SNPs were detected by MR-Radial outlier test.

Supplementary Table 6. Genetic instruments for the causal relationship of Mets and ASD.

SNP	CHR	Position	EA	OA	EAF	Beta for exposure	SE for exposure	P value for exposure	Beta for outcome	SE for outcome	P value for outcome	F statistics	PVE	Association with potential confounding factors
rs10116353	9	111950088	G	G	0.307	0.013	0.002	9.39E-09	-0.022	0.015	0.141	32.964	7.14E-05	NA
rs1013402	11	27712381	A	A	0.290	-0.025	0.002	1.34E-23	-0.001	0.015	0.962	100.255	2.17E-04	NA
rs10160660	11	76474537	T	T	0.248	0.013	0.002	4.83E-08	0.015	0.017	0.362	29.786	6.45E-05	NA
rs102275	11	61557803	T	T	0.363	-0.013	0.002	6.28E-11	0.008	0.015	0.577	42.731	9.25E-05	NA
rs1032524	1	201790826	T	T	0.484	-0.015	0.002	6.01E-12	0.006	0.014	0.645	47.325	1.02E-04	NA
rs1042034	2	21225281	C	C	0.218	-0.020	0.002	7.07E-21	0.007	0.017	0.682	87.848	1.90E-04	NA
rs10423928	19	46182304	T	T	0.214	0.020	0.003	8.49E-13	0.018	0.017	0.284	51.166	1.11E-04	NA
rs1046080	6	31595882	C	C	0.233	-0.015	0.003	1.75E-09	-0.016	0.016	0.317	36.231	7.84E-05	NA
rs10734924	12	123018894	T	T	0.259	-0.015	0.003	2.19E-09	0.009	0.016	0.584	35.801	7.75E-05	NA
rs10768994	11	43936945	T	T	0.430	0.012	0.002	4.52E-08	0.020	0.014	0.149	29.912	6.48E-05	NA
rs10779835	1	230299949	T	T	0.391	0.015	0.002	2.45E-14	-0.008	0.014	0.575	58.131	1.26E-04	NA
rs10783779	12	56491880	T	T	0.403	0.013	0.002	3.84E-09	-0.006	0.014	0.694	34.704	7.51E-05	NA
rs10808546	8	126495818	C	C	0.431	0.016	0.002	4.56E-19	-0.006	0.014	0.658	79.612	1.72E-04	NA
rs10838620	11	46818098	G	G	0.214	-0.015	0.002	1.25E-09	-0.011	0.016	0.487	36.883	7.98E-05	NA
rs10887579	10	88097210	A	A	0.354	-0.013	0.002	4.09E-09	-0.015	0.015	0.288	34.579	7.49E-05	NA
rs10930502	2	172890588	A	A	0.304	0.013	0.002	1.18E-08	-0.017	0.015	0.246	32.519	7.04E-05	NA
rs10938397	4	45182527	A	A	0.420	-0.023	0.002	8.14E-26	0.001	0.014	0.960	110.367	2.39E-04	NA
rs11079849	17	47090785	C	C	0.290	0.015	0.002	6.90E-11	-0.012	0.015	0.443	42.547	9.21E-05	NA
rs11150604	16	31037020	A	A	0.389	0.024	0.002	3.91E-28	0.014	0.014	0.328	120.954	2.62E-04	NA
rs11165643	1	96924097	C	C	0.417	-0.013	0.002	6.90E-10	0.001	0.014	0.954	38.049	8.24E-05	NA
rs11187845	10	96060198	C	C	0.096	0.020	0.004	8.08E-09	0.032	0.025	0.204	33.256	7.20E-05	NA
rs1138429	16	56942921	A	A	0.101	-0.028	0.003	1.42E-18	-0.018	0.024	0.447	77.365	1.67E-04	NA
rs1159974	6	126090277	T	T	0.499	-0.013	0.002	3.30E-10	-0.012	0.014	0.372	39.489	8.55E-05	NA
rs11611246	12	939480	G	G	0.183	-0.016	0.003	1.14E-08	-0.004	0.017	0.800	32.583	7.05E-05	NA
rs11631408	15	51746236	A	A	0.463	0.014	0.002	2.44E-11	0.027	0.014	0.049	44.582	9.65E-05	NA
rs11636565	15	36391846	C	C	0.488	-0.014	0.002	6.84E-12	-0.008	0.014	0.572	47.072	1.02E-04	NA
rs11660335	18	22154235	T	T	0.161	0.018	0.003	1.07E-09	-0.019	0.017	0.279	37.189	8.05E-05	NA
rs11747810	5	153100273	T	T	0.495	0.013	0.002	1.83E-09	-0.008	0.014	0.590	36.148	7.83E-05	NA
rs11766944	7	1888051	G	G	0.214	0.015	0.003	4.17E-08	0.014	0.018	0.417	30.068	6.51E-05	NA
rs11826999	11	116553778	G	G	0.223	-0.023	0.002	6.07E-26	-0.003	0.016	0.849	110.950	2.40E-04	NA
rs11866815	16	387867	C	C	0.242	0.016	0.002	2.32E-10	-0.022	0.016	0.190	40.181	8.70E-05	NA
rs11880870	19	18830704	A	A	0.499	0.013	0.002	2.98E-09	-0.005	0.014	0.710	35.198	7.62E-05	NA
rs11981190	7	71659538	G	G	0.341	0.013	0.002	5.84E-09	-0.021	0.015	0.144	33.887	7.34E-05	NA
rs1200349	15	41821752	T	T	0.482	-0.013	0.002	1.36E-09	0.013	0.014	0.346	36.724	7.95E-05	NA
rs12033257	1	112318484	A	A	0.382	0.014	0.002	8.97E-10	0.023	0.015	0.124	37.538	8.13E-05	NA
rs12201987	6	143182396	C	C	0.205	0.016	0.003	1.87E-09	0.019	0.018	0.276	36.103	7.82E-05	NA
rs12446554	16	19935073	G	G	0.130	0.020	0.003	3.79E-10	-0.035	0.020	0.085	39.220	8.49E-05	NA
rs12513212	4	96149346	A	A	0.387	-0.014	0.002	2.85E-10	-0.015	0.014	0.304	39.778	8.61E-05	NA
rs12520822	5	105699867	C	C	0.408	0.012	0.002	1.84E-08	-0.004	0.014	0.779	31.658	6.85E-05	NA
rs12596491	16	88928417	C	C	0.340	0.012	0.002	4.58E-08	0.003	0.016	0.878	29.888	6.47E-05	NA
rs12608504	19	18389135	A	A	0.359	0.016	0.002	2.27E-13	-0.006	0.014	0.692	53.757	1.16E-04	NA
rs12624640	20	32952125	G	G	0.430	-0.012	0.002	7.17E-09	-0.016	0.014	0.250	33.489	7.25E-05	NA
rs12824567	12	124495203	G	G	0.350	0.013	0.002	1.94E-09	0.009	0.015	0.543	36.032	7.80E-05	NA
rs12967135	18	57849023	G	G	0.240	-0.046	0.003	3.09E-66	-0.016	0.016	0.308	295.539	6.39E-04	Worrier or anxious feelings
rs12981256	19	1865901	G	G	0.475	-0.016	0.002	7.75E-14	-0.005	0.015	0.732	55.867	1.21E-04	NA
rs13024601	2	100902032	C	C	0.346	0.014	0.002	1.39E-09	-0.023	0.014	0.107	36.680	7.94E-05	NA
rs13029936	2	208465544	C	C	0.176	0.017	0.003	1.31E-09	0.020	0.018	0.253	36.795	7.96E-05	NA
rs13107325	4	103188709	C	C	0.080	-0.023	0.004	1.94E-09	-0.049	0.031	0.109	36.033	7.80E-05	Intelligence
rs13244268	7	72911843	T	T	0.114	0.024	0.003	9.17E-18	0.039	0.021	0.068	73.684	1.59E-04	NA

rs13250456	8	72408720	C	C	0.055	-0.025	0.005	4.67E-08	-0.040	0.034	0.235	29.850	6.46E-05	NA
rs13275593	8	68257300	G	G	0.285	-0.015	0.002	2.14E-10	0.029	0.016	0.058	40.332	8.73E-05	NA
rs1330304	9	16714629	C	C	0.267	0.013	0.002	2.32E-08	-0.016	0.016	0.326	31.202	6.75E-05	NA
rs13322435	3	156795468	A	A	0.407	0.020	0.002	6.60E-20	0.009	0.014	0.534	83.432	1.81E-04	NA
rs13416004	2	69664976	C	C	0.415	0.012	0.002	3.16E-08	-0.001	0.014	0.948	30.605	6.63E-05	NA
rs13416667	2	56608267	C	C	0.274	-0.015	0.002	2.32E-10	0.003	0.016	0.827	40.179	8.70E-05	NA
rs13435860	4	136975545	A	A	0.368	-0.013	0.002	1.94E-09	-0.009	0.015	0.526	36.037	7.80E-05	NA
rs1353580	15	47738732	T	T	0.374	-0.012	0.002	1.70E-08	0.012	0.014	0.376	31.810	6.89E-05	NA
rs1363695	5	130378027	C	C	0.228	0.015	0.003	6.49E-09	-0.012	0.016	0.450	33.681	7.29E-05	NA
rs1412239	9	28425515	C	C	0.304	-0.022	0.002	2.39E-21	0.004	0.015	0.794	89.993	1.95E-04	NA
rs1421085	16	53800954	T	T	0.432	-0.062	0.002	5.07E-152	0.004	0.014	0.759	689.747	1.49E-03	NA
rs1431659	8	73439070	A	A	0.278	0.019	0.002	4.48E-15	0.014	0.016	0.379	61.476	1.33E-04	NA
rs1448613	3	85875265	G	G	0.386	0.014	0.002	2.40E-10	-0.001	0.014	0.950	40.110	8.68E-05	NA
rs1456116	3	123084379	G	G	0.417	-0.013	0.002	1.99E-09	-0.006	0.014	0.676	35.987	7.79E-05	NA
rs1458156	12	41887940	C	C	0.449	-0.013	0.002	4.45E-09	-0.016	0.014	0.261	34.416	7.45E-05	NA
rs1460940	1	72814617	G	G	0.182	-0.020	0.003	2.89E-13	-0.026	0.018	0.152	53.284	1.15E-04	NA
rs1507153	6	79484386	C	C	0.364	-0.013	0.002	3.86E-09	0.001	0.014	0.949	34.694	7.51E-05	NA
rs1513475	3	35724256	T	T	0.343	0.015	0.002	8.46E-11	0.004	0.015	0.787	42.149	9.12E-05	NA
rs1534696	7	26397239	C	C	0.439	0.014	0.002	1.24E-11	-0.009	0.014	0.552	45.905	9.94E-05	NA
rs1580099	3	94003603	C	C	0.406	0.014	0.002	4.35E-09	0.010	0.014	0.489	34.459	7.46E-05	NA
rs17024393	1	110154688	T	T	0.033	-0.048	0.006	1.96E-15	-0.038	0.039	0.326	63.106	1.37E-04	NA
rs17115145	14	30122409	C	C	0.406	-0.012	0.002	1.29E-08	-0.010	0.014	0.487	32.350	7.00E-05	NA
rs17162333	1	27236757	C	C	0.132	-0.020	0.003	6.24E-11	-0.011	0.019	0.574	42.744	9.25E-05	NA
rs17207196	7	75101065	C	C	0.444	0.018	0.002	2.68E-16	0.026	0.014	0.069	67.024	1.45E-04	NA
rs1730858	1	107619244	T	T	0.328	0.015	0.002	1.04E-10	-0.008	0.015	0.573	41.736	9.03E-05	NA
rs17391694	1	78623626	C	C	0.096	-0.026	0.004	2.39E-12	-0.008	0.020	0.690	49.132	1.06E-04	NA
rs17618704	17	46221010	T	T	0.068	0.025	0.004	1.52E-09	-0.016	0.026	0.545	36.510	7.90E-05	NA
rs17639546	3	61251635	G	G	0.148	0.018	0.003	3.65E-09	-0.035	0.019	0.070	34.803	7.53E-05	NA
rs17694506	2	171632225	C	C	0.388	0.014	0.002	3.33E-11	-0.004	0.014	0.767	43.971	9.52E-05	NA
rs17724992	19	18454825	A	A	0.245	0.014	0.002	8.13E-09	-0.019	0.016	0.227	33.243	7.20E-05	NA
rs17783751	16	89513984	C	C	0.176	-0.015	0.003	4.79E-08	-0.012	0.017	0.473	29.800	6.45E-05	NA
rs17806379	20	51107290	C	C	0.185	0.020	0.003	3.64E-13	0.019	0.018	0.297	52.829	1.14E-04	NA
rs17821316	15	58703069	A	A	0.488	-0.012	0.002	9.97E-10	0.023	0.015	0.125	37.331	8.08E-05	NA
rs1784460	11	118938371	T	T	0.360	-0.015	0.002	8.15E-12	-0.003	0.014	0.848	46.729	1.01E-04	NA
rs1876359	16	4930100	C	C	0.377	-0.013	0.002	5.40E-09	0.010	0.015	0.507	34.038	7.37E-05	NA
rs1886558	13	111982615	A	A	0.389	0.012	0.002	4.14E-08	0.005	0.015	0.756	30.082	6.51E-05	NA
rs1899689	7	121964349	C	C	0.380	-0.012	0.002	4.27E-08	-0.016	0.014	0.257	30.021	6.50E-05	NA
rs1914888	17	21267541	A	A	0.462	0.013	0.002	1.33E-09	-0.009	0.014	0.520	36.762	7.96E-05	NA
rs192812	3	161491831	C	C	0.454	-0.012	0.002	4.87E-09	0.001	0.014	0.966	34.241	7.41E-05	NA
rs1945941	11	132620118	G	G	0.376	0.012	0.002	3.63E-08	0.020	0.014	0.145	30.337	6.57E-05	NA
rs2002023	10	76848524	C	C	0.441	-0.012	0.002	8.92E-09	0.024	0.014	0.090	33.064	7.16E-05	NA
rs2015407	14	103274547	A	A	0.345	0.014	0.002	4.68E-10	0.014	0.015	0.324	38.808	8.40E-05	NA
rs2035831	3	173683791	G	G	0.321	0.013	0.002	1.48E-08	-0.002	0.015	0.918	32.085	6.95E-05	NA
rs2060604	8	76650334	T	T	0.414	0.015	0.002	2.80E-12	0.010	0.014	0.466	48.822	1.06E-04	NA
rs2065418	11	30422068	T	T	0.354	0.016	0.002	1.39E-12	0.015	0.014	0.288	50.196	1.09E-04	NA
rs2066295	6	26168903	A	A	0.219	0.023	0.003	5.73E-18	-0.025	0.016	0.121	74.611	1.61E-04	NA
rs2074881	19	1970021	C	C	0.179	0.015	0.003	2.25E-08	0.018	0.019	0.348	31.270	6.77E-05	NA
rs2075226	2	36805676	A	A	0.396	-0.013	0.002	2.48E-08	-0.008	0.015	0.572	31.073	6.73E-05	NA
rs208015	17	46252346	T	T	0.088	0.022	0.004	1.07E-08	0.011	0.028	0.684	32.711	7.08E-05	NA
rs2143253	20	41987392	G	G	0.137	0.019	0.003	1.29E-09	-0.037	0.022	0.095	36.823	7.97E-05	NA
rs2180454	14	29690513	T	T	0.241	-0.015	0.003	1.90E-09	0.010	0.016	0.518	36.071	7.81E-05	NA

rs2237717	7	116405387	T	T	0.443	-0.011	0.002	2.63E-08	-0.004	0.014	0.790	30.960	6.70E-05	NA
rs2242449	17	7095507	C	C	0.417	-0.013	0.002	2.85E-09	0.005	0.014	0.732	35.287	7.64E-05	NA
rs227199	1	210265384	G	G	0.316	0.013	0.002	5.06E-09	0.000	0.014	0.978	34.168	7.40E-05	NA
rs2298969	4	3186244	A	A	0.490	0.012	0.002	2.79E-08	0.006	0.014	0.692	30.851	6.68E-05	NA
rs2299383	7	103418846	C	C	0.410	-0.014	0.002	2.20E-10	0.010	0.014	0.462	40.282	8.72E-05	NA
rs2307111	5	75003678	T	T	0.396	0.025	0.002	4.41E-29	-0.006	0.014	0.691	125.285	2.71E-04	NA
rs2396625	7	113028634	T	T	0.401	0.014	0.002	1.99E-10	-0.015	0.014	0.284	40.473	8.76E-05	NA
rs2436726	6	40366427	C	C	0.405	-0.016	0.002	2.24E-12	0.002	0.014	0.903	49.262	1.07E-04	NA
rs2470945	7	104585760	C	C	0.408	-0.012	0.002	2.10E-08	-0.013	0.014	0.353	31.396	6.80E-05	NA
rs2516740	16	2097110	A	A	0.226	0.020	0.003	7.50E-14	0.007	0.019	0.717	55.934	1.21E-04	NA
rs2531686	10	118651011	C	C	0.276	0.015	0.002	2.22E-10	0.016	0.016	0.317	40.261	8.72E-05	NA
rs2531995	16	4013467	C	C	0.387	-0.019	0.002	2.80E-17	-0.001	0.015	0.947	71.477	1.55E-04	NA
rs2596933	3	9365746	G	G	0.267	0.014	0.002	1.26E-08	0.012	0.017	0.491	32.396	7.01E-05	NA
rs261336	15	58742418	G	G	0.161	-0.020	0.003	2.07E-14	-0.007	0.019	0.701	58.461	1.27E-04	NA
rs2781668	6	131897278	C	C	0.170	-0.015	0.003	3.64E-08	-0.031	0.019	0.103	30.331	6.57E-05	NA
rs2787693	1	49915848	C	C	0.302	0.017	0.002	1.94E-11	-0.012	0.015	0.421	45.027	9.75E-05	NA
rs2814944	6	34552797	G	G	0.137	-0.034	0.003	1.03E-26	0.023	0.019	0.222	114.464	2.48E-04	NA
rs2820232	6	35003603	G	G	0.459	-0.012	0.002	2.95E-08	0.001	0.014	0.943	30.741	6.65E-05	NA
rs2836753	21	40291187	T	T	0.385	-0.013	0.002	7.29E-10	-0.002	0.014	0.867	37.941	8.21E-05	NA
rs2861688	2	67838474	T	T	0.273	0.016	0.002	8.97E-11	-0.012	0.016	0.430	42.033	9.10E-05	NA
rs2925979	16	81534790	T	T	0.288	0.014	0.002	1.10E-10	-0.004	0.015	0.817	41.628	9.01E-05	NA
rs2968856	7	150614934	T	T	0.261	0.016	0.002	1.80E-11	-0.021	0.016	0.204	45.179	9.78E-05	NA
rs2990997	1	190119703	T	T	0.353	-0.012	0.002	3.24E-08	0.007	0.015	0.610	30.558	6.62E-05	NA
rs320	8	19819077	T	T	0.287	0.031	0.002	2.30E-43	-0.005	0.016	0.762	190.642	4.13E-04	NA
rs323781	5	86752616	A	A	0.034	-0.032	0.006	3.82E-08	0.067	0.041	0.102	30.237	6.55E-05	NA
rs349088	11	84814393	C	C	0.480	0.013	0.002	3.23E-09	0.003	0.014	0.822	35.040	7.59E-05	NA
rs350832	19	4069426	G	G	0.227	-0.014	0.003	1.88E-08	-0.011	0.018	0.522	31.615	6.84E-05	NA
rs3808436	8	116560967	A	A	0.432	0.016	0.002	1.58E-13	-0.001	0.014	0.943	54.468	1.18E-04	NA
rs3810291	19	47569003	G	G	0.337	-0.019	0.002	2.74E-17	-0.008	0.015	0.600	71.521	1.55E-04	NA
rs3814424	5	87968953	C	C	0.172	-0.025	0.003	2.55E-16	-0.022	0.019	0.236	67.124	1.45E-04	Intelligence
rs3852012	3	44045981	G	G	0.287	0.013	0.002	1.57E-08	0.017	0.015	0.262	31.963	6.92E-05	NA
rs3864004	3	41240177	G	G	0.432	-0.014	0.002	2.30E-09	0.026	0.014	0.061	35.700	7.73E-05	NA
rs3902840	9	129419025	G	G	0.092	-0.021	0.004	1.72E-08	0.002	0.025	0.951	31.786	6.88E-05	NA
rs397092	21	46582564	A	A	0.456	-0.013	0.002	1.07E-09	0.004	0.014	0.797	37.196	8.05E-05	NA
rs40067	5	107439012	G	G	0.180	0.018	0.003	4.05E-11	0.028	0.019	0.132	43.590	9.44E-05	NA
rs4074448	15	63888495	G	G	0.434	0.012	0.002	2.37E-08	0.012	0.014	0.410	31.161	6.75E-05	NA
rs4283409	2	228977142	A	A	0.387	-0.012	0.002	7.64E-09	-0.014	0.014	0.342	33.365	7.22E-05	NA
rs4377779	6	12117344	T	T	0.334	0.013	0.002	5.27E-09	-0.019	0.014	0.196	34.086	7.38E-05	NA
rs4410790	7	17284577	T	T	0.385	-0.012	0.002	5.72E-09	-0.013	0.015	0.370	33.927	7.34E-05	NA
rs442177	4	88030261	G	G	0.421	-0.011	0.002	3.89E-08	-0.008	0.014	0.582	30.204	6.54E-05	NA
rs4477562	13	54104968	C	C	0.110	-0.020	0.003	9.80E-09	0.012	0.021	0.547	32.880	7.12E-05	NA
rs4482463	2	205375909	C	C	0.081	0.029	0.004	4.75E-12	-0.030	0.026	0.259	47.787	1.03E-04	NA
rs4660208	1	39722214	A	A	0.195	-0.023	0.003	2.84E-18	0.013	0.017	0.431	75.995	1.64E-04	NA
rs4665991	2	27766284	G	G	0.264	-0.013	0.002	2.16E-09	0.007	0.016	0.655	35.821	7.75E-05	NA
rs4671328	2	58935282	T	T	0.430	0.018	0.002	9.13E-17	0.026	0.014	0.061	69.149	1.50E-04	NA
rs4693325	4	94408529	A	A	0.199	-0.016	0.003	1.74E-09	0.013	0.018	0.458	36.241	7.85E-05	NA
rs4740619	9	15634326	T	T	0.481	0.014	0.002	6.33E-11	-0.003	0.014	0.857	42.715	9.25E-05	NA
rs4755720	11	43628749	C	C	0.400	0.016	0.002	4.50E-14	-0.002	0.014	0.867	56.935	1.23E-04	NA
rs4777542	15	73082366	C	C	0.300	0.014	0.002	3.14E-09	0.011	0.015	0.484	35.098	7.60E-05	NA
rs4784745	16	57014875	A	A	0.329	-0.019	0.002	4.00E-21	0.027	0.015	0.066	88.973	1.93E-04	NA
rs4790292	17	1824305	C	C	0.144	0.020	0.003	2.66E-10	-0.001	0.019	0.942	39.913	8.64E-05	NA

rs479315	11	66064991	G	G	0.170	-0.016	0.003	2.08E-08	0.029	0.018	0.108	31.420	6.80E-05	NA
rs4805758	19	32866424	G	G	0.162	0.020	0.003	5.55E-13	-0.001	0.019	0.955	51.999	1.13E-04	Fed-up feelings
rs4810479	20	44545048	C	C	0.288	0.013	0.002	6.17E-10	-0.010	0.016	0.535	38.267	8.28E-05	NA
rs4889782	17	78640510	C	C	0.415	0.013	0.002	1.61E-09	0.000	0.014	0.978	36.391	7.88E-05	NA
rs4890439	18	40732586	G	G	0.375	0.012	0.002	4.60E-08	-0.003	0.014	0.820	29.879	6.47E-05	NA
rs4929923	11	8639200	T	T	0.377	-0.015	0.002	4.28E-11	-0.003	0.014	0.824	43.484	9.41E-05	NA
rs4947406	7	50661735	T	T	0.350	0.016	0.002	9.35E-12	-0.002	0.015	0.876	46.461	1.01E-04	NA
rs4971632	2	50240091	T	T	0.166	0.017	0.003	1.26E-09	-0.025	0.019	0.184	36.870	7.98E-05	NA
rs512005	1	33377679	T	T	0.236	0.014	0.003	4.27E-08	-0.003	0.017	0.856	30.024	6.50E-05	NA
rs545608	1	177899121	G	G	0.186	-0.038	0.003	1.86E-37	0.015	0.017	0.374	163.594	3.54E-04	NA
rs563296	10	99772404	G	G	0.441	-0.013	0.002	1.23E-08	0.016	0.014	0.257	32.438	7.02E-05	NA
rs6115015	20	25027692	G	G	0.333	-0.013	0.002	6.30E-09	0.004	0.015	0.780	33.741	7.30E-05	NA
rs619054	11	116660813	G	G	0.232	0.015	0.002	2.70E-11	0.008	0.017	0.638	44.384	9.61E-05	NA
rs645040	3	135926622	G	G	0.229	-0.023	0.002	1.31E-20	-0.020	0.017	0.236	86.621	1.87E-04	NA
rs6463489	7	5542513	T	T	0.069	0.024	0.004	1.03E-08	0.034	0.023	0.139	32.787	7.10E-05	NA
rs649458	1	23712528	T	T	0.137	0.018	0.003	5.66E-09	-0.028	0.021	0.178	33.949	7.35E-05	NA
rs6499240	16	69686912	A	A	0.422	-0.020	0.002	1.52E-18	0.018	0.014	0.201	77.230	1.67E-04	NA
rs6499861	16	56991495	C	C	0.162	-0.018	0.003	8.61E-12	-0.003	0.019	0.862	46.623	1.01E-04	NA
rs6545714	2	59307725	G	G	0.382	0.019	0.002	3.15E-18	-0.021	0.014	0.141	75.796	1.64E-04	NA
rs6548834	3	82704753	G	G	0.378	-0.013	0.002	1.08E-08	0.014	0.014	0.324	32.694	7.08E-05	NA
rs6550597	3	18738940	G	G	0.298	-0.013	0.002	4.61E-09	0.004	0.016	0.813	34.346	7.43E-05	NA
rs6575340	14	94023972	G	G	0.377	-0.014	0.002	8.82E-10	-0.010	0.015	0.473	37.571	8.13E-05	NA
rs6577584	1	6715390	T	T	0.372	-0.014	0.002	1.65E-10	-0.016	0.014	0.255	40.843	8.84E-05	NA
rs6602151	10	16764483	T	T	0.294	-0.015	0.002	5.89E-10	-0.010	0.015	0.522	38.356	8.30E-05	NA
rs6603004	15	84568504	A	A	0.466	-0.013	0.002	1.81E-09	0.008	0.014	0.572	36.163	7.83E-05	NA
rs6673977	1	7736097	A	A	0.354	-0.013	0.002	1.63E-08	-0.019	0.014	0.193	31.892	6.90E-05	NA
rs6754311	2	136707982	T	T	0.489	0.013	0.002	3.95E-10	-0.024	0.016	0.130	39.137	8.47E-05	NA
rs676387	17	40706273	C	C	0.266	-0.013	0.002	3.42E-08	0.022	0.015	0.159	30.452	6.59E-05	NA
rs6868198	5	122750714	G	G	0.432	0.013	0.002	2.10E-08	-0.004	0.014	0.759	31.399	6.80E-05	NA
rs6870983	5	87697533	C	C	0.236	0.020	0.003	1.66E-15	0.023	0.017	0.183	63.434	1.37E-04	NA
rs6907923	6	51802669	G	G	0.341	-0.015	0.002	1.71E-10	-0.012	0.015	0.412	40.774	8.83E-05	NA
rs7016529	8	19806631	T	T	0.011	-0.102	0.009	1.94E-27	0.050	0.058	0.392	117.778	2.55E-04	NA
rs703965	10	80955067	C	C	0.456	-0.012	0.002	8.25E-09	0.017	0.014	0.236	33.214	7.19E-05	NA
rs705986	8	64737682	A	A	0.491	0.012	0.002	2.53E-08	-0.017	0.014	0.213	31.039	6.72E-05	NA
rs7075195	10	65050659	A	A	0.435	0.017	0.002	5.31E-16	-0.011	0.014	0.419	65.676	1.42E-04	Educational attainment
rs7132908	12	50263148	G	G	0.357	-0.019	0.002	9.07E-15	-0.015	0.014	0.284	60.088	1.30E-04	NA
rs7161874	15	53163740	C	C	0.214	-0.015	0.003	4.11E-09	0.021	0.016	0.193	34.573	7.48E-05	NA
rs7198357	16	67884619	C	C	0.178	0.017	0.003	4.09E-11	0.003	0.018	0.872	43.573	9.43E-05	NA
rs7206608	16	82872628	C	C	0.313	-0.013	0.002	1.26E-08	-0.001	0.015	0.940	32.386	7.01E-05	NA
rs7241918	18	47160953	G	G	0.163	0.018	0.003	7.52E-13	0.019	0.018	0.312	51.404	1.11E-04	NA
rs7323827	13	94116675	A	A	0.304	0.013	0.002	4.59E-09	0.004	0.015	0.791	34.358	7.44E-05	NA
rs741959	1	47676233	A	A	0.402	0.014	0.002	6.19E-11	-0.019	0.014	0.174	42.760	9.26E-05	NA
rs7488867	12	103699685	C	C	0.281	0.015	0.003	1.57E-09	0.003	0.016	0.858	36.447	7.89E-05	NA
rs7515635	1	42408070	T	T	0.471	0.012	0.002	4.68E-09	-0.006	0.014	0.664	34.317	7.43E-05	NA
rs7549987	1	80789011	C	C	0.299	-0.014	0.002	4.02E-09	-0.012	0.015	0.444	34.614	7.49E-05	NA
rs7581217	2	25524944	T	T	0.370	0.013	0.002	5.93E-09	0.021	0.015	0.160	33.857	7.33E-05	NA
rs7601028	2	642499	C	C	0.174	-0.046	0.003	1.02E-52	-0.001	0.019	0.940	233.512	5.05E-04	NA
rs7638388	3	47914974	C	C	0.088	-0.024	0.004	1.01E-10	-0.018	0.023	0.444	41.794	9.05E-05	NA
rs7642876	3	15883094	A	A	0.404	0.012	0.002	1.35E-08	0.003	0.014	0.816	32.265	6.98E-05	NA
rs7668263	4	143781835	T	T	0.382	-0.012	0.002	4.21E-08	-0.008	0.015	0.581	30.049	6.50E-05	NA
rs779655	5	170495158	G	G	0.272	-0.014	0.002	1.21E-08	0.016	0.015	0.290	32.464	7.03E-05	NA

rs7824519	8	14222796	T	T	0.324	-0.013	0.002	1.51E-08	-0.007	0.015	0.638	32.035	6.93E-05	NA
rs7928842	11	47566352	T	T	0.422	-0.020	0.002	2.30E-20	-0.013	0.014	0.368	85.511	1.85E-04	NA
rs7947951	11	13356030	A	A	0.297	-0.014	0.002	1.59E-10	0.008	0.015	0.596	40.911	8.86E-05	NA
rs7962636	12	116450006	T	T	0.355	0.013	0.002	1.32E-08	-0.009	0.015	0.533	32.308	6.99E-05	NA
rs7982447	13	54453811	T	T	0.200	-0.019	0.003	6.75E-13	-0.005	0.017	0.764	51.616	1.12E-04	NA
rs8015400	14	25930988	C	C	0.348	-0.018	0.002	8.13E-15	0.017	0.015	0.240	60.303	1.31E-04	NA
rs8055138	16	28891465	C	C	0.329	-0.030	0.002	1.86E-35	-0.003	0.014	0.848	154.432	3.34E-04	Educational attainment; Intelligence
rs8078079	17	34959346	T	T	0.395	0.015	0.002	2.89E-11	0.010	0.014	0.493	44.247	9.58E-05	NA
rs818524	1	85201228	T	T	0.289	-0.013	0.002	2.84E-08	-0.012	0.016	0.461	30.812	6.67E-05	NA
rs8192675	3	170724883	T	T	0.289	-0.013	0.002	4.30E-08	-0.007	0.015	0.662	30.007	6.50E-05	NA
rs891387	18	21103909	T	T	0.473	0.021	0.002	3.25E-21	-0.024	0.014	0.081	89.383	1.93E-04	Educational attainment
rs906673	3	12898941	C	C	0.389	0.013	0.002	3.67E-09	0.012	0.014	0.395	34.789	7.53E-05	NA
rs9277972	6	33276770	A	A	0.168	-0.016	0.003	1.55E-08	-0.006	0.018	0.765	31.995	6.93E-05	NA
rs9478499	6	154345289	T	T	0.147	-0.019	0.003	1.35E-10	0.017	0.019	0.370	41.238	8.93E-05	NA
rs9556979	13	99241507	T	T	0.324	-0.013	0.002	4.53E-09	-0.009	0.015	0.567	34.382	7.44E-05	NA
rs9563569	13	58571207	G	G	0.150	0.018	0.003	2.09E-08	0.015	0.018	0.384	31.408	6.80E-05	NA
rs9630985	2	181607676	A	A	0.349	-0.014	0.002	9.30E-11	0.010	0.015	0.499	41.963	9.08E-05	NA
rs9686661	5	55861786	C	C	0.186	-0.023	0.003	4.65E-18	0.008	0.018	0.675	75.025	1.62E-04	NA
rs9816226	3	185834499	A	A	0.188	-0.016	0.003	8.56E-09	0.009	0.019	0.646	33.144	7.17E-05	NA
rs9872156	3	176337348	G	G	0.260	-0.013	0.002	4.04E-08	-0.009	0.015	0.552	30.129	6.52E-05	NA
rs987237	6	50803050	A	A	0.181	-0.030	0.003	7.81E-26	-0.021	0.018	0.250	110.449	2.39E-04	NA
rs9947450	18	58052665	C	C	0.030	0.062	0.007	1.22E-21	-0.007	0.037	0.845	91.321	1.98E-04	NA
rs998584	6	43757896	C	C	0.496	-0.013	0.002	9.99E-11	0.002	0.014	0.914	41.823	9.05E-05	NA
rs9987289	8	9183358	A	A	0.075	0.022	0.004	1.84E-09	-0.008	0.024	0.726	36.136	7.82E-05	NA

Genomic positions reported in the GWAS of Mets refer to human reference assembly (GRCh37/hg19).

Abbreviations: MetS, Metabolic syndrome; ASD, Autism spectrum disorder; SNPs, Single nucleotide polymorphisms; CHR, Chromosome; EA, Effect allele; OA, Other allele; EAF, Effect allele frequency; SE, Standard error; PVE, Proportion of phenotypic variance explained.

Palindromic and not-inferrable SNPs: rs1937433, rs2253310, rs6916318.

3 SNPs were detected by PRESSO outlier test, and 20 SNPs were detected by MR-Radial outlier test.

Supplementary Table 7. Genetic instruments for the causal relationship of Mets and BIP.

SNP	CHR	Position	EA	OA	EAF	Beta for exposure	SE for exposure	P value for exposure	Beta for outcome	SE for outcome	P value for outcome	F statistics	PVE	Association with potential confounding factors
rs10116353	9	111950088	G	T	0.307	0.013	0.002	9.39E-09	-0.014	0.015	0.334	32.964	7.14E-05	NA
rs1013402	11	27712381	A	G	0.290	-0.025	0.002	1.34E-23	-0.014	0.015	0.327	100.255	2.17E-04	NA
rs10160660	11	76474537	T	C	0.248	0.013	0.002	4.83E-08	-0.028	0.016	0.081	29.786	6.45E-05	NA
rs10423928	19	46182304	T	A	0.214	0.020	0.003	8.49E-13	0.032	0.018	0.070	51.166	1.11E-04	NA
rs10768994	11	43936945	T	C	0.430	0.012	0.002	4.52E-08	0.017	0.014	0.211	29.912	6.48E-05	NA
rs10779835	1	230299949	T	C	0.391	0.015	0.002	2.45E-14	-0.009	0.014	0.511	58.131	1.26E-04	NA
rs10783779	12	56491880	T	G	0.403	0.013	0.002	3.84E-09	-0.006	0.014	0.693	34.704	7.51E-05	NA
rs10808546	8	126495818	C	T	0.431	0.016	0.002	4.56E-19	-0.006	0.013	0.638	79.612	1.72E-04	NA
rs10838620	11	46818098	G	A	0.214	-0.015	0.002	1.25E-09	0.001	0.016	0.960	36.883	7.98E-05	NA
rs10887579	10	88097210	A	C	0.354	-0.013	0.002	4.09E-09	-0.010	0.014	0.503	34.579	7.49E-05	NA
rs10930502	2	172890588	A	G	0.304	0.013	0.002	1.18E-08	-0.016	0.015	0.280	32.519	7.04E-05	NA
rs10938397	4	45182527	A	G	0.420	-0.023	0.002	8.14E-26	0.007	0.014	0.622	110.367	2.39E-04	NA
rs11072938	15	81021661	C	T	0.241	0.016	0.002	4.30E-11	-0.021	0.015	0.154	43.470	9.41E-05	NA
rs11079849	17	47090785	C	T	0.290	0.015	0.002	6.90E-11	-0.018	0.015	0.220	42.547	9.21E-05	NA
rs11150604	16	31037020	A	T	0.389	0.024	0.002	3.91E-28	0.014	0.014	0.307	120.954	2.62E-04	NA
rs11165643	1	96924097	C	T	0.417	-0.013	0.002	6.90E-10	-0.008	0.014	0.578	38.049	8.24E-05	NA
rs1159974	6	126090277	T	C	0.499	-0.013	0.002	3.30E-10	-0.012	0.013	0.391	39.489	8.55E-05	NA
rs11611246	12	939480	G	T	0.183	-0.016	0.003	1.14E-08	-0.001	0.016	0.947	32.583	7.05E-05	NA
rs11631408	15	51746236	A	G	0.463	0.014	0.002	2.44E-11	-0.020	0.014	0.149	44.582	9.65E-05	NA
rs11660335	18	22154235	T	C	0.161	0.018	0.003	1.07E-09	0.002	0.017	0.889	37.189	8.05E-05	NA
rs11676272	2	25141538	A	G	0.464	-0.020	0.002	1.05E-17	-0.007	0.013	0.622	73.421	1.59E-04	NA
rs11747810	5	153100273	T	C	0.495	0.013	0.002	1.83E-09	-0.005	0.013	0.692	36.148	7.83E-05	NA
rs11826999	11	116553778	G	A	0.223	-0.023	0.002	6.07E-26	-0.004	0.016	0.814	110.950	2.40E-04	NA
rs11866815	16	387867	C	T	0.242	0.016	0.002	2.32E-10	0.016	0.016	0.296	40.181	8.70E-05	NA
rs11981190	7	71659538	G	A	0.341	0.013	0.002	5.84E-09	0.009	0.014	0.539	33.887	7.34E-05	NA
rs1200349	15	41821752	T	C	0.482	-0.013	0.002	1.36E-09	0.009	0.013	0.489	36.724	7.95E-05	NA
rs12033257	1	112318484	A	G	0.382	0.014	0.002	8.97E-10	0.000	0.014	0.983	37.538	8.13E-05	NA
rs12201987	6	143182396	C	T	0.205	0.016	0.003	1.87E-09	0.009	0.017	0.609	36.103	7.82E-05	NA
rs12446554	16	19935073	G	T	0.130	0.020	0.003	3.79E-10	0.009	0.020	0.637	39.220	8.49E-05	NA
rs12520822	5	105699867	C	T	0.408	0.012	0.002	1.84E-08	0.006	0.014	0.662	31.658	6.85E-05	NA
rs12596491	16	88928417	C	T	0.340	0.012	0.002	4.58E-08	-0.005	0.015	0.722	29.888	6.47E-05	NA
rs12608504	19	18389135	A	G	0.359	0.016	0.002	2.27E-13	0.018	0.014	0.191	53.757	1.16E-04	NA
rs12824567	12	124495203	G	C	0.350	0.013	0.002	1.94E-09	0.021	0.014	0.144	36.032	7.80E-05	NA
rs12967135	18	57849023	G	A	0.240	-0.046	0.003	3.09E-66	0.011	0.016	0.488	295.539	6.39E-04	Worrier or anxious feelings
rs12981256	19	1865901	G	A	0.475	-0.016	0.002	7.75E-14	0.009	0.014	0.522	55.867	1.21E-04	NA
rs13024601	2	100902032	C	T	0.346	0.014	0.002	1.39E-09	0.010	0.014	0.487	36.680	7.94E-05	NA
rs13107325	4	103188709	C	T	0.080	-0.023	0.004	1.94E-09	-0.043	0.027	0.104	36.033	7.80E-05	Intelligence
rs13244268	7	72911843	T	C	0.114	0.024	0.003	9.17E-18	-0.011	0.021	0.583	73.684	1.59E-04	NA
rs13250456	8	72408720	C	T	0.055	-0.025	0.005	4.67E-08	-0.028	0.032	0.372	29.850	6.46E-05	NA
rs13275593	8	68257300	G	A	0.285	-0.015	0.002	2.14E-10	-0.019	0.015	0.221	40.332	8.73E-05	NA
rs1330304	9	16714629	C	G	0.267	0.013	0.002	2.32E-08	0.028	0.015	0.067	31.202	6.75E-05	NA
rs13322435	3	156795468	A	G	0.407	0.020	0.002	6.60E-20	0.006	0.014	0.670	83.432	1.81E-04	NA
rs13416004	2	69664976	C	T	0.415	0.012	0.002	3.16E-08	-0.001	0.014	0.948	30.605	6.63E-05	NA
rs13416667	2	56608267	C	A	0.274	-0.015	0.002	2.32E-10	-0.016	0.015	0.284	40.179	8.70E-05	NA
rs13435860	4	136975545	A	T	0.368	-0.013	0.002	1.94E-09	0.003	0.014	0.858	36.037	7.80E-05	NA
rs1353580	15	47738732	T	A	0.374	-0.012	0.002	1.70E-08	0.011	0.014	0.439	31.810	6.89E-05	NA
rs1362910	8	30856464	A	G	0.386	0.014	0.002	9.70E-11	-0.011	0.014	0.419	41.882	9.07E-05	NA
rs1363695	5	130378027	C	T	0.228	0.015	0.003	6.49E-09	0.004	0.016	0.812	33.681	7.29E-05	NA
rs1421085	16	53800954	T	C	0.432	-0.062	0.002	5.07E-152	-0.009	0.014	0.494	689.747	1.49E-03	NA
rs1431659	8	73439070	A	G	0.278	0.019	0.002	4.48E-15	-0.019	0.015	0.213	61.476	1.33E-04	NA
rs1448613	3	85875265	G	A	0.386	0.014	0.002	2.40E-10	0.016	0.014	0.244	40.110	8.68E-05	NA
rs1452075	3	62481063	C	T	0.284	-0.014	0.002	9.96E-09	-0.021	0.015	0.162	32.850	7.11E-05	NA

rs1456116	3	123084379	G	A	0.417	-0.013	0.002	1.99E-09	-0.011	0.013	0.420	35.987	7.79E-05	NA
rs1458156	12	41887940	C	T	0.449	-0.013	0.002	4.45E-09	-0.007	0.013	0.581	34.416	7.45E-05	NA
rs1460940	1	72814617	G	A	0.182	-0.020	0.003	2.89E-13	-0.007	0.017	0.688	53.284	1.15E-04	NA
rs1507153	6	79484386	C	A	0.364	-0.013	0.002	3.86E-09	0.018	0.014	0.192	34.694	7.51E-05	NA
rs1513475	3	35724256	T	C	0.343	0.015	0.002	8.46E-11	0.009	0.014	0.540	42.149	9.12E-05	NA
rs1534696	7	26397239	C	A	0.439	0.014	0.002	1.24E-11	0.007	0.014	0.602	45.905	9.94E-05	NA
rs17024393	1	110154688	T	C	0.033	-0.048	0.006	1.96E-15	-0.044	0.039	0.257	63.106	1.37E-04	NA
rs17162333	1	27236757	C	G	0.132	-0.020	0.003	6.24E-11	-0.024	0.019	0.193	42.744	9.25E-05	NA
rs17207196	7	75101065	C	T	0.444	0.018	0.002	2.68E-16	0.018	0.015	0.231	67.024	1.45E-04	NA
rs1730858	1	107619244	T	C	0.328	0.015	0.002	1.04E-10	-0.005	0.014	0.730	41.736	9.03E-05	NA
rs17513613	19	30286822	T	C	0.289	-0.017	0.002	1.94E-13	0.017	0.015	0.241	54.063	1.17E-04	NA
rs17618704	17	46221010	T	G	0.068	0.025	0.004	1.52E-09	-0.012	0.026	0.649	36.510	7.90E-05	NA
rs17639546	3	61251635	G	A	0.148	0.018	0.003	3.65E-09	0.032	0.019	0.096	34.803	7.53E-05	NA
rs17694506	2	171632225	C	T	0.388	0.014	0.002	3.33E-11	0.023	0.014	0.090	43.971	9.52E-05	NA
rs17724992	19	18454825	A	G	0.245	0.014	0.002	8.13E-09	-0.011	0.015	0.491	33.243	7.20E-05	NA
rs17806379	20	51107290	C	T	0.185	0.020	0.003	3.64E-13	0.002	0.018	0.932	52.829	1.14E-04	NA
rs17821316	15	58703069	A	C	0.488	-0.012	0.002	9.97E-10	0.027	0.015	0.061	37.331	8.08E-05	NA
rs1784460	11	118938371	T	A	0.360	-0.015	0.002	8.15E-12	0.010	0.014	0.467	46.729	1.01E-04	NA
rs1876359	16	4930100	C	T	0.377	-0.013	0.002	5.40E-09	0.018	0.014	0.189	34.038	7.37E-05	NA
rs1886558	13	111982615	A	G	0.389	0.012	0.002	4.14E-08	0.001	0.014	0.919	30.082	6.51E-05	NA
rs1899689	7	121964349	C	T	0.380	-0.012	0.002	4.27E-08	-0.002	0.014	0.896	30.021	6.50E-05	NA
rs1914888	17	21267541	A	G	0.462	0.013	0.002	1.33E-09	-0.025	0.014	0.075	36.762	7.96E-05	NA
rs1945941	11	132620118	G	A	0.376	0.012	0.002	3.63E-08	0.005	0.014	0.741	30.337	6.57E-05	NA
rs2002023	10	76848524	C	T	0.441	-0.012	0.002	8.92E-09	-0.018	0.014	0.191	33.064	7.16E-05	NA
rs2015407	14	103274547	A	G	0.345	0.014	0.002	4.68E-10	0.013	0.014	0.335	38.808	8.40E-05	NA
rs2035831	3	173683791	G	C	0.321	0.013	0.002	1.48E-08	-0.001	0.014	0.944	32.085	6.95E-05	NA
rs2060604	8	76650334	T	C	0.414	0.015	0.002	2.80E-12	-0.003	0.014	0.824	48.822	1.06E-04	NA
rs2065418	11	30422068	T	G	0.354	0.016	0.002	1.39E-12	0.008	0.014	0.589	50.196	1.09E-04	NA
rs2066295	6	26168903	A	G	0.219	0.023	0.003	5.73E-18	-0.020	0.016	0.191	74.611	1.61E-04	NA
rs2074881	19	1970021	C	T	0.179	0.015	0.003	2.25E-08	-0.016	0.019	0.416	31.270	6.77E-05	NA
rs2075226	2	36805676	A	G	0.396	-0.013	0.002	2.48E-08	0.001	0.014	0.966	31.073	6.73E-05	NA
rs208015	17	46252346	T	C	0.088	0.022	0.004	1.07E-08	-0.009	0.025	0.726	32.711	7.08E-05	NA
rs2180454	14	29690513	T	C	0.241	-0.015	0.003	1.90E-09	0.005	0.016	0.771	36.071	7.81E-05	NA
rs2237717	7	116405387	T	C	0.443	-0.011	0.002	2.63E-08	-0.017	0.013	0.199	30.960	6.70E-05	NA
rs2242449	17	7095507	C	T	0.417	-0.013	0.002	2.85E-09	-0.020	0.015	0.174	35.287	7.64E-05	NA
rs227199	1	210265384	G	C	0.316	0.013	0.002	5.06E-09	-0.006	0.014	0.696	34.168	7.40E-05	NA
rs2275543	9	107651174	T	C	0.099	-0.019	0.003	6.58E-09	0.016	0.023	0.466	33.655	7.29E-05	NA
rs2298969	4	3186244	A	G	0.490	0.012	0.002	2.79E-08	0.020	0.013	0.141	30.851	6.68E-05	NA
rs2299383	7	103418846	C	T	0.410	-0.014	0.002	2.20E-10	0.005	0.014	0.684	40.282	8.72E-05	NA
rs2307111	5	75003678	T	C	0.396	0.025	0.002	4.41E-29	0.000	0.014	0.994	125.285	2.71E-04	NA
rs2436726	6	40366427	C	T	0.405	-0.016	0.002	2.24E-12	0.011	0.013	0.391	49.262	1.07E-04	NA
rs2453763	5	92376460	T	A	0.347	-0.012	0.002	3.72E-08	-0.001	0.014	0.948	30.290	6.56E-05	NA
rs2526754	3	50125996	G	A	0.494	0.022	0.002	5.98E-22	0.010	0.013	0.443	92.735	2.01E-04	Educational attainment; Intelligence
rs2531686	10	118651011	C	G	0.276	0.015	0.002	2.22E-10	0.024	0.015	0.110	40.261	8.72E-05	NA
rs2531995	16	4013467	C	T	0.387	-0.019	0.002	2.80E-17	0.007	0.014	0.619	71.477	1.55E-04	NA
rs2596933	3	9365746	G	A	0.267	0.014	0.002	1.26E-08	0.011	0.015	0.476	32.396	7.01E-05	NA
rs261336	15	58742418	G	A	0.161	-0.020	0.003	2.07E-14	0.029	0.018	0.110	58.461	1.27E-04	NA
rs2781668	6	131897278	C	T	0.170	-0.015	0.003	3.64E-08	0.001	0.018	0.965	30.331	6.57E-05	NA
rs2787693	1	49915848	C	A	0.302	0.017	0.002	1.94E-11	0.016	0.014	0.251	45.027	9.75E-05	NA
rs2814944	6	34552797	G	A	0.137	-0.034	0.003	1.03E-26	-0.036	0.019	0.050	114.464	2.48E-04	NA

rs2820232	6	35003603	G	T	0.459	-0.012	0.002	2.95E-08	-0.015	0.013	0.250	30.741	6.65E-05	NA
rs2861688	2	67838474	T	C	0.273	0.016	0.002	8.97E-11	0.016	0.015	0.289	42.033	9.10E-05	NA
rs2925979	16	81534790	T	C	0.288	0.014	0.002	1.10E-10	0.004	0.015	0.777	41.628	9.01E-05	NA
rs2968856	7	150614934	T	C	0.261	0.016	0.002	1.80E-11	-0.002	0.016	0.919	45.179	9.78E-05	NA
rs2990997	1	190119703	T	A	0.353	-0.012	0.002	3.24E-08	0.020	0.014	0.140	30.558	6.62E-05	NA
rs323781	5	86752616	A	C	0.034	-0.032	0.006	3.82E-08	-0.054	0.039	0.163	30.237	6.55E-05	NA
rs350832	19	4069426	G	A	0.227	-0.014	0.003	1.88E-08	0.002	0.017	0.894	31.615	6.84E-05	NA
rs3749262	3	131624097	G	A	0.263	-0.019	0.002	1.12E-14	0.021	0.015	0.162	59.681	1.29E-04	NA
rs3808436	8	116560967	A	G	0.432	0.016	0.002	1.58E-13	0.002	0.013	0.887	54.468	1.18E-04	NA
rs3810291	19	47569003	G	A	0.337	-0.019	0.002	2.74E-17	0.007	0.015	0.615	71.521	1.55E-04	NA
rs3852012	3	44045981	G	A	0.287	0.013	0.002	1.57E-08	-0.016	0.015	0.264	31.963	6.92E-05	NA
rs3864004	3	41240177	G	A	0.432	-0.014	0.002	2.30E-09	-0.008	0.013	0.531	35.700	7.73E-05	NA
rs3902840	9	129419025	G	A	0.092	-0.021	0.004	1.72E-08	-0.004	0.023	0.867	31.786	6.88E-05	NA
rs397092	21	46582564	A	G	0.456	-0.013	0.002	1.07E-09	-0.007	0.013	0.606	37.196	8.05E-05	NA
rs40067	5	107439012	G	A	0.180	0.018	0.003	4.05E-11	0.024	0.018	0.182	43.590	9.44E-05	NA
rs4074448	15	63888495	G	A	0.434	0.012	0.002	2.37E-08	0.005	0.014	0.707	31.161	6.75E-05	NA
rs4283409	2	228977142	A	G	0.387	-0.012	0.002	7.64E-09	0.025	0.014	0.074	33.365	7.22E-05	NA
rs429343	2	147903382	A	G	0.437	0.012	0.002	1.26E-08	0.001	0.014	0.947	32.394	7.01E-05	NA
rs4377779	6	12117344	T	C	0.334	0.013	0.002	5.27E-09	0.021	0.014	0.138	34.086	7.38E-05	NA
rs4410790	7	17284577	T	C	0.385	-0.012	0.002	5.72E-09	-0.005	0.014	0.719	33.927	7.34E-05	NA
rs442177	4	88030261	G	T	0.421	-0.011	0.002	3.89E-08	0.010	0.014	0.449	30.204	6.54E-05	NA
rs4477562	13	54104968	C	T	0.110	-0.020	0.003	9.80E-09	-0.008	0.020	0.704	32.880	7.12E-05	NA
rs4482463	2	205375909	C	A	0.081	0.029	0.004	4.75E-12	0.022	0.025	0.378	47.787	1.03E-04	NA
rs4693325	4	94408529	A	C	0.199	-0.016	0.003	1.74E-09	0.003	0.017	0.859	36.241	7.85E-05	NA
rs4740619	9	15634326	T	C	0.481	0.014	0.002	6.33E-11	-0.019	0.014	0.168	42.715	9.25E-05	NA
rs4755720	11	43628749	C	T	0.400	0.016	0.002	4.50E-14	-0.004	0.014	0.800	56.935	1.23E-04	NA
rs4777542	15	73082366	C	T	0.300	0.014	0.002	3.14E-09	0.014	0.015	0.331	35.098	7.60E-05	NA
rs4784745	16	57014875	A	G	0.329	-0.019	0.002	4.00E-21	0.013	0.015	0.377	88.973	1.93E-04	NA
rs4805758	19	32866424	G	A	0.162	0.020	0.003	5.55E-13	-0.011	0.018	0.526	51.999	1.13E-04	Fed-up feelings
rs4810479	20	44545048	C	T	0.288	0.013	0.002	6.17E-10	-0.011	0.016	0.494	38.267	8.28E-05	NA
rs4889782	17	78640510	C	T	0.415	0.013	0.002	1.61E-09	0.023	0.014	0.090	36.391	7.88E-05	NA
rs4890439	18	40732586	G	A	0.375	0.012	0.002	4.60E-08	0.017	0.014	0.207	29.879	6.47E-05	NA
rs4929923	11	8639200	T	C	0.377	-0.015	0.002	4.28E-11	-0.012	0.014	0.392	43.484	9.41E-05	NA
rs4947406	7	50661735	T	G	0.350	0.016	0.002	9.35E-12	0.016	0.015	0.270	46.461	1.01E-04	NA
rs4971632	2	50240091	T	C	0.166	0.017	0.003	1.26E-09	0.003	0.018	0.854	36.870	7.98E-05	NA
rs545608	1	177899121	G	C	0.186	-0.038	0.003	1.86E-37	0.008	0.017	0.628	163.594	3.54E-04	NA
rs6115015	20	25027692	G	A	0.333	-0.013	0.002	6.30E-09	0.007	0.015	0.622	33.741	7.30E-05	NA
rs619054	11	116660813	G	A	0.232	0.015	0.002	2.70E-11	0.002	0.016	0.884	44.384	9.61E-05	NA
rs645040	3	135926622	G	T	0.229	-0.023	0.002	1.31E-20	0.005	0.016	0.743	86.621	1.87E-04	NA
rs6463489	7	5542513	T	C	0.069	0.024	0.004	1.03E-08	0.003	0.022	0.888	32.787	7.10E-05	NA
rs649458	1	23712528	T	A	0.137	0.018	0.003	5.66E-09	-0.002	0.019	0.918	33.949	7.35E-05	NA
rs6499240	16	69686912	A	G	0.422	-0.020	0.002	1.52E-18	-0.003	0.014	0.814	77.230	1.67E-04	NA
rs6499861	16	56991495	C	G	0.162	-0.018	0.003	8.61E-12	-0.011	0.019	0.549	46.623	1.01E-04	NA
rs6545714	2	59307725	G	A	0.382	0.019	0.002	3.15E-18	-0.006	0.014	0.672	75.796	1.64E-04	NA
rs6548834	3	82704753	G	A	0.378	-0.013	0.002	1.08E-08	0.010	0.014	0.463	32.694	7.08E-05	NA
rs6550597	3	18738940	G	A	0.298	-0.013	0.002	4.61E-09	-0.005	0.015	0.742	34.346	7.43E-05	NA
rs6575340	14	94023972	G	A	0.377	-0.014	0.002	8.82E-10	0.018	0.014	0.206	37.571	8.13E-05	NA
rs6577584	1	6715390	T	G	0.372	-0.014	0.002	1.65E-10	0.002	0.014	0.903	40.843	8.84E-05	NA
rs6602151	10	16764483	T	C	0.294	-0.015	0.002	5.89E-10	0.005	0.015	0.710	38.356	8.30E-05	NA
rs6603004	15	84568504	A	G	0.466	-0.013	0.002	1.81E-09	0.009	0.013	0.502	36.163	7.83E-05	NA
rs6673977	1	7736097	A	G	0.354	-0.013	0.002	1.63E-08	0.013	0.014	0.346	31.892	6.90E-05	NA
rs6711375	2	161090873	G	A	0.320	-0.014	0.002	1.11E-09	-0.017	0.014	0.243	37.115	8.03E-05	NA

rs6754311	2	136707982	T	C	0.489	0.013	0.002	3.95E-10	-0.012	0.016	0.431	39.137	8.47E-05	NA
rs676387	17	40706273	C	A	0.266	-0.013	0.002	3.42E-08	0.007	0.015	0.615	30.452	6.59E-05	NA
rs6868198	5	122750714	G	A	0.432	0.013	0.002	2.10E-08	0.024	0.014	0.078	31.399	6.80E-05	NA
rs6870983	5	87697533	C	T	0.236	0.020	0.003	1.66E-15	-0.024	0.016	0.138	63.434	1.37E-04	NA
rs687672	11	65649984	T	C	0.189	0.021	0.003	5.92E-14	-0.020	0.017	0.251	56.399	1.22E-04	NA
rs6907923	6	51802669	G	A	0.341	-0.015	0.002	1.71E-10	0.003	0.014	0.826	40.774	8.83E-05	NA
rs7016529	8	19806631	T	C	0.011	-0.102	0.009	1.94E-27	-0.039	0.055	0.475	117.778	2.55E-04	NA
rs705986	8	64737682	A	G	0.491	0.012	0.002	2.53E-08	0.017	0.013	0.194	31.039	6.72E-05	NA
rs7075195	10	65050659	A	G	0.435	0.017	0.002	5.31E-16	-0.002	0.014	0.871	65.676	1.42E-04	Educational attainment
rs7132908	12	50263148	G	A	0.357	-0.019	0.002	9.07E-15	0.010	0.014	0.454	60.088	1.30E-04	NA
rs7144011	14	79940383	G	T	0.207	-0.023	0.003	1.71E-17	0.014	0.016	0.390	72.451	1.57E-04	NA
rs7161874	15	53163740	C	G	0.214	-0.015	0.003	4.11E-09	0.001	0.015	0.958	34.573	7.48E-05	NA
rs7186893	16	24806420	G	T	0.267	0.020	0.002	3.04E-16	0.011	0.015	0.476	66.779	1.45E-04	Mood swings
rs7206608	16	82872628	C	G	0.313	-0.013	0.002	1.26E-08	0.020	0.015	0.168	32.386	7.01E-05	NA
rs7212293	17	65847325	A	G	0.216	-0.022	0.003	4.05E-17	-0.003	0.017	0.861	70.753	1.53E-04	NA
rs7241918	18	47160953	G	T	0.163	0.018	0.003	7.52E-13	0.017	0.018	0.345	51.404	1.11E-04	NA
rs7323827	13	94116675	A	G	0.304	0.013	0.002	4.59E-09	-0.009	0.014	0.508	34.358	7.44E-05	NA
rs741959	1	47676233	A	G	0.402	0.014	0.002	6.19E-11	-0.018	0.014	0.193	42.760	9.26E-05	NA
rs7488867	12	103699685	C	T	0.281	0.015	0.003	1.57E-09	0.009	0.015	0.561	36.447	7.89E-05	NA
rs7515635	1	42408070	T	C	0.471	0.012	0.002	4.68E-09	-0.001	0.013	0.923	34.317	7.43E-05	NA
rs7537581	1	2725475	C	A	0.476	-0.012	0.002	4.55E-08	-0.012	0.014	0.392	29.899	6.47E-05	NA
rs7549987	1	80789011	C	T	0.299	-0.014	0.002	4.02E-09	-0.015	0.015	0.317	34.614	7.49E-05	NA
rs7581217	2	25524944	T	C	0.370	0.013	0.002	5.93E-09	0.019	0.015	0.196	33.857	7.33E-05	NA
rs7601028	2	642499	C	G	0.174	-0.046	0.003	1.02E-52	0.010	0.018	0.574	233.512	5.05E-04	NA
rs7638388	3	47914974	C	T	0.088	-0.024	0.004	1.01E-10	0.039	0.024	0.115	41.794	9.05E-05	NA
rs7642876	3	15883094	A	G	0.404	0.012	0.002	1.35E-08	0.000	0.014	0.982	32.265	6.98E-05	NA
rs779655	5	170495158	G	C	0.272	-0.014	0.002	1.21E-08	0.017	0.015	0.246	32.464	7.03E-05	NA
rs7928842	11	47566352	T	C	0.422	-0.020	0.002	2.30E-20	0.010	0.014	0.448	85.511	1.85E-04	NA
rs7962636	12	116450006	T	C	0.355	0.013	0.002	1.32E-08	-0.002	0.014	0.905	32.308	6.99E-05	NA
rs7982447	13	54453811	T	C	0.200	-0.019	0.003	6.75E-13	-0.018	0.017	0.267	51.616	1.12E-04	NA
rs8015400	14	25930988	C	A	0.348	-0.018	0.002	8.13E-15	-0.001	0.014	0.944	60.303	1.31E-04	NA
rs808820	12	89744826	A	G	0.116	-0.022	0.004	8.37E-10	-0.032	0.021	0.121	37.671	8.15E-05	NA
rs818524	1	85201228	T	C	0.289	-0.013	0.002	2.84E-08	0.012	0.015	0.410	30.812	6.67E-05	NA
rs8192675	3	170724883	T	C	0.289	-0.013	0.002	4.30E-08	-0.010	0.015	0.507	30.007	6.50E-05	NA
rs891387	18	21103909	T	C	0.473	0.021	0.002	3.25E-21	-0.016	0.013	0.244	89.383	1.93E-04	Educational attainment
rs906673	3	12898941	C	T	0.389	0.013	0.002	3.67E-09	-0.010	0.014	0.491	34.789	7.53E-05	NA
rs9277972	6	33276770	A	T	0.168	-0.016	0.003	1.55E-08	0.028	0.017	0.105	31.995	6.93E-05	NA
rs938875	15	67952922	A	G	0.408	-0.019	0.002	3.72E-18	-0.026	0.014	0.052	75.463	1.63E-04	NA
rs9478499	6	154345289	T	A	0.147	-0.019	0.003	1.35E-10	-0.018	0.018	0.328	41.238	8.93E-05	NA
rs9556979	13	99241507	T	G	0.324	-0.013	0.002	4.53E-09	-0.010	0.015	0.501	34.382	7.44E-05	NA
rs9563569	13	58571207	G	A	0.150	0.018	0.003	2.09E-08	-0.029	0.017	0.099	31.408	6.80E-05	NA
rs9630985	2	181607676	A	C	0.349	-0.014	0.002	9.30E-11	0.008	0.014	0.569	41.963	9.08E-05	NA
rs9686661	5	55861786	C	T	0.186	-0.023	0.003	4.65E-18	0.000	0.017	0.991	75.025	1.62E-04	NA
rs9872156	3	176337348	G	A	0.260	-0.013	0.002	4.04E-08	-0.013	0.015	0.389	30.129	6.52E-05	NA
rs987237	6	50803050	A	G	0.181	-0.030	0.003	7.81E-26	-0.021	0.017	0.213	110.449	2.39E-04	NA
rs9947450	18	58052665	C	T	0.030	0.062	0.007	1.22E-21	-0.011	0.035	0.752	91.321	1.98E-04	NA
rs998584	6	43757896	C	A	0.496	-0.013	0.002	9.99E-11	0.007	0.014	0.632	41.823	9.05E-05	NA
rs9987289	8	9183358	A	G	0.075	0.022	0.004	1.84E-09	0.029	0.023	0.220	36.136	7.82E-05	NA

Genomic positions reported in the GWAS of MetS refer to human reference assembly (GRCh37/hg19).

Abbreviations: MetS, Metabolic syndrome; BIP, Bipolar disorder; SNPs, Single nucleotide polymorphisms; CHR, Chromosome; EA, Effect allele; OA, Other allele; EAF, Effect allele frequency; SE, Standard error; PVE, Proportion of phenotypic variance explained.

SNPs that are unavailable in the outcome GWAS: rs1046080, rs705986, rs1412239,among them, rs705986 and rs1412239 were substitute by proxy SNPs: rs811715 and rs1412235, respectively.

Palindromic and not-inferrable SNPs that are abandoned as instruments: rs1937433, rs2253310, rs2396625, rs6916318.

7 SNPs were detected by PRESSO outlier test, and 38 SNPs were detected by MR-Radial outlier test.

Supplementary Table 8. Genetic instruments for the causal relationship of Mets and MDD.

SNP	CHR	Position	EA	OA	EAF	Beta for exposure	SE for exposure	P value for exposure	Beta for outcome	SE for outcome	P value for outcome	F statistics	PVE	Association with potential confounding factors
rs10116353	9	111950088	G	T	0.307	0.013	0.002	9.39E-09	0.009	0.010	0.364	32.964	7.14E-05	NA
rs10160660	11	76474537	T	C	0.248	0.013	0.002	4.83E-08	0.022	0.011	0.044	29.786	6.45E-05	NA
rs1032524	1	201790826	T	C	0.484	-0.015	0.002	6.01E-12	-0.016	0.009	0.074	47.325	1.02E-04	NA
rs1042034	2	21225281	C	T	0.218	-0.020	0.002	7.07E-21	-0.003	0.011	0.755	87.848	1.90E-04	NA
rs10423928	19	46182304	T	A	0.214	0.020	0.003	8.49E-13	0.017	0.011	0.135	51.166	1.11E-04	NA
rs10734924	12	123018894	T	G	0.259	-0.015	0.003	2.19E-09	0.005	0.010	0.645	35.801	7.75E-05	NA
rs10768994	11	43936945	T	C	0.430	0.012	0.002	4.52E-08	0.003	0.009	0.748	29.912	6.48E-05	NA
rs10779835	1	230299949	T	C	0.391	0.015	0.002	2.45E-14	0.007	0.009	0.449	58.131	1.26E-04	NA
rs10783779	12	56491880	T	G	0.403	0.013	0.002	3.84E-09	0.004	0.009	0.660	34.704	7.51E-05	NA
rs10808546	8	126495818	C	T	0.431	0.016	0.002	4.56E-19	-0.003	0.009	0.710	79.612	1.72E-04	NA
rs10838620	11	46818098	G	A	0.214	-0.015	0.002	1.25E-09	-0.009	0.011	0.375	36.883	7.98E-05	NA
rs10872224	6	98435125	G	T	0.409	-0.014	0.002	9.76E-11	0.007	0.009	0.410	41.868	9.06E-05	Educational attainment; Intelligence
rs10887579	10	88097210	A	C	0.354	-0.013	0.002	4.09E-09	-0.005	0.010	0.628	34.579	7.49E-05	NA
rs10938397	4	45182527	A	G	0.420	-0.023	0.002	8.14E-26	-0.012	0.009	0.201	110.367	2.39E-04	NA
rs11072938	15	81021661	C	T	0.241	0.016	0.002	4.30E-11	0.013	0.010	0.187	43.470	9.41E-05	NA
rs11079849	17	47090785	C	T	0.290	0.015	0.002	6.90E-11	-0.012	0.010	0.201	42.547	9.21E-05	NA
rs11150604	16	31037020	A	T	0.389	0.024	0.002	3.91E-28	0.012	0.009	0.173	120.954	2.62E-04	NA
rs11165643	1	96924097	C	T	0.417	-0.013	0.002	6.90E-10	0.010	0.009	0.276	38.049	8.24E-05	NA
rs11187845	10	96060198	C	A	0.096	0.020	0.004	8.08E-09	-0.015	0.016	0.343	33.256	7.20E-05	NA
rs1138429	16	56942921	A	T	0.101	-0.028	0.003	1.42E-18	-0.014	0.016	0.353	77.365	1.67E-04	NA
rs1159974	6	126090277	T	C	0.499	-0.013	0.002	3.30E-10	0.004	0.009	0.637	39.489	8.55E-05	NA
rs11611246	12	939480	G	T	0.183	-0.016	0.003	1.14E-08	-0.001	0.011	0.942	32.583	7.05E-05	NA
rs11631408	15	51746236	A	G	0.463	0.014	0.002	2.44E-11	0.005	0.009	0.564	44.582	9.65E-05	NA
rs11660335	18	22154235	T	C	0.161	0.018	0.003	1.07E-09	0.001	0.011	0.915	37.189	8.05E-05	NA
rs11676272	2	25141538	A	G	0.464	-0.020	0.002	1.05E-17	-0.006	0.009	0.486	73.421	1.59E-04	NA
rs11747810	5	153100273	T	C	0.495	0.013	0.002	1.83E-09	0.019	0.009	0.028	36.148	7.83E-05	NA
rs11826999	11	116553778	G	A	0.223	-0.023	0.002	6.07E-26	-0.008	0.011	0.452	110.950	2.40E-04	NA
rs11866815	16	387867	C	T	0.242	0.016	0.002	2.32E-10	0.011	0.011	0.313	40.181	8.70E-05	NA
rs11880870	19	18830704	A	G	0.499	0.013	0.002	2.98E-09	0.016	0.010	0.094	35.198	7.62E-05	NA
rs11981190	7	71659538	G	A	0.341	0.013	0.002	5.84E-09	-0.016	0.010	0.084	33.887	7.34E-05	NA
rs1200349	15	41821752	T	C	0.482	-0.013	0.002	1.36E-09	-0.005	0.009	0.582	36.724	7.95E-05	NA
rs12033257	1	112318484	A	G	0.382	0.014	0.002	8.97E-10	-0.008	0.010	0.405	37.538	8.13E-05	NA
rs12446554	16	19935073	G	T	0.130	0.020	0.003	3.79E-10	-0.016	0.013	0.207	39.220	8.49E-05	NA
rs12513212	4	96149346	A	C	0.387	-0.014	0.002	2.85E-10	-0.002	0.009	0.843	39.778	8.61E-05	NA
rs12520822	5	105699867	C	T	0.408	0.012	0.002	1.84E-08	0.001	0.009	0.955	31.658	6.85E-05	NA
rs12596491	16	88928417	C	T	0.340	0.012	0.002	4.58E-08	0.021	0.011	0.052	29.888	6.47E-05	NA
rs12608504	19	18389135	A	G	0.359	0.016	0.002	2.27E-13	-0.012	0.010	0.220	53.757	1.16E-04	NA
rs12624640	20	32952125	G	A	0.430	-0.012	0.002	7.17E-09	-0.013	0.009	0.137	33.489	7.25E-05	NA
rs12824567	12	124495203	G	C	0.350	0.013	0.002	1.94E-09	0.012	0.010	0.195	36.032	7.80E-05	NA
rs12967135	18	57849023	G	A	0.240	-0.046	0.003	3.09E-66	0.011	0.010	0.281	295.539	6.39E-04	Worrier or anxious feelings
rs13024601	2	100902032	C	T	0.346	0.014	0.002	1.39E-09	0.007	0.009	0.466	36.680	7.94E-05	NA
rs13107325	4	103188709	C	T	0.080	-0.023	0.004	1.94E-09	0.027	0.019	0.161	36.033	7.80E-05	Intelligence
rs13206549	6	162978032	T	G	0.134	0.019	0.003	1.54E-09	0.014	0.013	0.293	36.486	7.90E-05	NA
rs13244268	7	72911843	T	C	0.114	0.024	0.003	9.17E-18	0.010	0.014	0.462	73.684	1.59E-04	NA
rs13250456	8	72408720	C	T	0.055	-0.025	0.005	4.67E-08	-0.009	0.022	0.665	29.850	6.46E-05	NA
rs13275593	8	68257300	G	A	0.285	-0.015	0.002	2.14E-10	0.002	0.010	0.865	40.332	8.73E-05	NA
rs1330304	9	16714629	C	G	0.267	0.013	0.002	2.32E-08	-0.001	0.010	0.953	31.202	6.75E-05	NA
rs13322435	3	156795468	A	G	0.407	0.020	0.002	6.60E-20	0.003	0.009	0.772	83.432	1.81E-04	NA
rs13416004	2	69664976	C	T	0.415	0.012	0.002	3.16E-08	-0.001	0.009	0.885	30.605	6.63E-05	NA

rs13416667	2	56608267	C	A	0.274	-0.015	0.002	2.32E-10	0.001	0.010	0.913	40.179	8.70E-05	NA
rs13435860	4	136975545	A	T	0.368	-0.013	0.002	1.94E-09	0.000	0.009	0.983	36.037	7.80E-05	NA
rs1353580	15	47738732	T	A	0.374	-0.012	0.002	1.70E-08	-0.010	0.009	0.282	31.810	6.89E-05	NA
rs1362910	8	30856464	A	G	0.386	0.014	0.002	9.70E-11	0.020	0.009	0.026	41.882	9.07E-05	NA
rs1363695	5	130378027	C	T	0.228	0.015	0.003	6.49E-09	0.008	0.011	0.455	33.681	7.29E-05	NA
rs1412239	9	28425515	C	G	0.304	-0.022	0.002	2.39E-21	0.002	0.010	0.853	89.993	1.95E-04	NA
rs1421085	16	53800954	T	C	0.432	-0.062	0.002	5.07E-152	-0.016	0.009	0.072	689.747	1.49E-03	NA
rs1431659	8	73439070	A	G	0.278	0.019	0.002	4.48E-15	-0.014	0.010	0.156	61.476	1.33E-04	NA
rs1448613	3	85875265	G	A	0.386	0.014	0.002	2.40E-10	0.002	0.009	0.872	40.110	8.68E-05	NA
rs1456116	3	123084379	G	A	0.417	-0.013	0.002	1.99E-09	0.004	0.009	0.660	35.987	7.79E-05	NA
rs1458156	12	41887940	C	T	0.449	-0.013	0.002	4.45E-09	0.008	0.009	0.375	34.416	7.45E-05	NA
rs1513475	3	35724256	T	C	0.343	0.015	0.002	8.46E-11	0.019	0.010	0.049	42.149	9.12E-05	NA
rs1580099	3	94003603	C	A	0.406	0.014	0.002	4.35E-09	0.014	0.009	0.125	34.459	7.46E-05	NA
rs17115145	14	30122409	C	T	0.406	-0.012	0.002	1.29E-08	-0.014	0.009	0.119	32.350	7.00E-05	NA
rs17162333	1	27236757	C	G	0.132	-0.020	0.003	6.24E-11	-0.004	0.012	0.723	42.744	9.25E-05	NA
rs17207196	7	75101065	C	T	0.444	0.018	0.002	2.68E-16	0.015	0.010	0.136	67.024	1.45E-04	NA
rs1730858	1	107619244	T	C	0.328	0.015	0.002	1.04E-10	0.017	0.010	0.068	41.736	9.03E-05	NA
rs17391694	1	78623626	C	T	0.096	-0.026	0.004	2.39E-12	-0.009	0.014	0.503	49.132	1.06E-04	NA
rs17513613	19	30286822	T	C	0.289	-0.017	0.002	1.94E-13	-0.004	0.010	0.666	54.063	1.17E-04	NA
rs17618704	17	46221010	T	G	0.068	0.025	0.004	1.52E-09	0.011	0.017	0.522	36.510	7.90E-05	NA
rs17694506	2	171632225	C	T	0.388	0.014	0.002	3.33E-11	0.016	0.009	0.075	43.971	9.52E-05	NA
rs17724992	19	18454825	A	G	0.245	0.014	0.002	8.13E-09	0.015	0.011	0.176	33.243	7.20E-05	NA
rs17783751	16	89513984	C	A	0.176	-0.015	0.003	4.79E-08	-0.001	0.011	0.957	29.800	6.45E-05	NA
rs17806379	20	51107290	C	T	0.185	0.020	0.003	3.64E-13	0.018	0.012	0.118	52.829	1.14E-04	NA
rs17821316	15	58703069	A	C	0.488	-0.012	0.002	9.97E-10	0.014	0.010	0.135	37.331	8.08E-05	NA
rs1784460	11	118938371	T	A	0.360	-0.015	0.002	8.15E-12	0.002	0.009	0.837	46.729	1.01E-04	NA
rs1876359	16	4930100	C	T	0.377	-0.013	0.002	5.40E-09	-0.017	0.010	0.077	34.038	7.37E-05	NA
rs1886558	13	111982615	A	G	0.389	0.012	0.002	4.14E-08	0.018	0.009	0.054	30.082	6.51E-05	NA
rs1899689	7	121964349	C	T	0.380	-0.012	0.002	4.27E-08	-0.016	0.009	0.089	30.021	6.50E-05	NA
rs1914888	17	21267541	A	G	0.462	0.013	0.002	1.33E-09	0.002	0.010	0.803	36.762	7.96E-05	NA
rs192812	3	161491831	C	T	0.454	-0.012	0.002	4.87E-09	-0.014	0.009	0.120	34.241	7.41E-05	NA
rs1945941	11	132620118	G	A	0.376	0.012	0.002	3.63E-08	0.003	0.009	0.772	30.337	6.57E-05	NA
rs2002023	10	76848524	C	T	0.441	-0.012	0.002	8.92E-09	-0.015	0.009	0.099	33.064	7.16E-05	NA
rs2015407	14	103274547	A	G	0.345	0.014	0.002	4.68E-10	0.006	0.009	0.519	38.808	8.40E-05	NA
rs2035831	3	173683791	G	C	0.321	0.013	0.002	1.48E-08	-0.010	0.009	0.292	32.085	6.95E-05	NA
rs2060604	8	76650334	T	C	0.414	0.015	0.002	2.80E-12	0.018	0.009	0.046	48.822	1.06E-04	NA
rs2065418	11	30422068	T	G	0.354	0.016	0.002	1.39E-12	0.004	0.009	0.664	50.196	1.09E-04	NA
rs2074881	19	1970021	C	T	0.179	0.015	0.003	2.25E-08	0.004	0.014	0.796	31.270	6.77E-05	NA
rs2075226	2	36805676	A	G	0.396	-0.013	0.002	2.48E-08	-0.005	0.009	0.606	31.073	6.73E-05	NA
rs2143253	20	41987392	G	A	0.137	0.019	0.003	1.29E-09	0.024	0.014	0.078	36.823	7.97E-05	NA
rs2180454	14	29690513	T	C	0.241	-0.015	0.003	1.90E-09	-0.013	0.010	0.215	36.071	7.81E-05	NA
rs2237717	7	116405387	T	C	0.443	-0.011	0.002	2.63E-08	0.000	0.009	0.964	30.960	6.70E-05	NA
rs2242449	17	7095507	C	T	0.417	-0.013	0.002	2.85E-09	0.004	0.009	0.671	35.287	7.64E-05	NA
rs227199	1	210265384	G	C	0.316	0.013	0.002	5.06E-09	0.014	0.009	0.135	34.168	7.40E-05	NA
rs2272168	7	99102777	G	C	0.163	0.017	0.003	3.68E-09	-0.022	0.013	0.100	34.785	7.53E-05	NA
rs2275543	9	107651174	T	C	0.099	-0.019	0.003	6.58E-09	-0.013	0.015	0.402	33.655	7.29E-05	NA
rs2298969	4	3186244	A	G	0.490	0.012	0.002	2.79E-08	0.009	0.009	0.339	30.851	6.68E-05	NA
rs2299383	7	103418846	C	T	0.410	-0.014	0.002	2.20E-10	0.004	0.009	0.673	40.282	8.72E-05	NA
rs2307111	5	75003678	T	C	0.396	0.025	0.002	4.41E-29	0.006	0.009	0.505	125.285	2.71E-04	NA
rs2436726	6	40366427	C	T	0.405	-0.016	0.002	2.24E-12	0.011	0.009	0.234	49.262	1.07E-04	NA
rs2453763	5	92376460	T	A	0.347	-0.012	0.002	3.72E-08	-0.020	0.009	0.035	30.290	6.56E-05	NA

rs2470945	7	104585760	C	A	0.408	-0.012	0.002	2.10E-08	-0.014	0.009	0.138	31.396	6.80E-05	NA
rs2516740	16	2097110	A	C	0.226	0.020	0.003	7.50E-14	-0.018	0.013	0.177	55.934	1.21E-04	NA
rs2526754	3	50125996	G	A	0.494	0.022	0.002	5.98E-22	0.013	0.009	0.141	92.735	2.01E-04	Educational attainment; Intelligence
rs2531686	10	118651011	C	G	0.276	0.015	0.002	2.22E-10	0.002	0.010	0.874	40.261	8.72E-05	NA
rs2531995	16	4013467	C	T	0.387	-0.019	0.002	2.80E-17	0.010	0.010	0.317	71.477	1.55E-04	NA
rs261336	15	58742418	G	A	0.161	-0.020	0.003	2.07E-14	0.010	0.012	0.425	58.461	1.27E-04	NA
rs2781668	6	131897278	C	T	0.170	-0.015	0.003	3.64E-08	0.016	0.012	0.189	30.331	6.57E-05	NA
rs2787693	1	49915848	C	A	0.302	0.017	0.002	1.94E-11	0.003	0.010	0.713	45.027	9.75E-05	NA
rs2814944	6	34552797	G	A	0.137	-0.034	0.003	1.03E-26	0.008	0.012	0.517	114.464	2.48E-04	NA
rs2820232	6	35003603	G	T	0.459	-0.012	0.002	2.95E-08	0.010	0.009	0.271	30.741	6.65E-05	NA
rs2836753	21	40291187	T	C	0.385	-0.013	0.002	7.29E-10	-0.006	0.009	0.500	37.941	8.21E-05	NA
rs2861688	2	67838474	T	C	0.273	0.016	0.002	8.97E-11	-0.011	0.010	0.267	42.033	9.10E-05	NA
rs2925979	16	81534790	T	C	0.288	0.014	0.002	1.10E-10	-0.002	0.010	0.808	41.628	9.01E-05	NA
rs2968856	7	150614934	T	C	0.261	0.016	0.002	1.80E-11	-0.011	0.011	0.299	45.179	9.78E-05	NA
rs2990997	1	190119703	T	A	0.353	-0.012	0.002	3.24E-08	-0.007	0.009	0.478	30.558	6.62E-05	NA
rs320	8	19819077	T	G	0.287	0.031	0.002	2.30E-43	-0.009	0.010	0.384	190.642	4.13E-04	NA
rs323781	5	86752616	A	C	0.034	-0.032	0.006	3.82E-08	-0.021	0.026	0.435	30.237	6.55E-05	NA
rs349088	11	84814393	C	A	0.480	0.013	0.002	3.23E-09	0.011	0.009	0.234	35.040	7.59E-05	NA
rs350832	19	4069426	G	A	0.227	-0.014	0.003	1.88E-08	-0.002	0.012	0.857	31.615	6.84E-05	NA
rs3749262	3	131624097	G	A	0.263	-0.019	0.002	1.12E-14	-0.010	0.010	0.322	59.681	1.29E-04	NA
rs3810291	19	47569003	G	A	0.337	-0.019	0.002	2.74E-17	0.007	0.010	0.444	71.521	1.55E-04	NA
rs3852012	3	44045981	G	A	0.287	0.013	0.002	1.57E-08	0.007	0.010	0.479	31.963	6.92E-05	NA
rs3864004	3	41240177	G	A	0.432	-0.014	0.002	2.30E-09	0.009	0.009	0.301	35.700	7.73E-05	NA
rs3902840	9	129419025	G	A	0.092	-0.021	0.004	1.72E-08	-0.004	0.016	0.820	31.786	6.88E-05	NA
rs40067	5	107439012	G	A	0.180	0.018	0.003	4.05E-11	0.025	0.012	0.035	43.590	9.44E-05	NA
rs4283409	2	228977142	A	G	0.387	-0.012	0.002	7.64E-09	-0.006	0.009	0.550	33.365	7.22E-05	NA
rs429343	2	147903382	A	G	0.437	0.012	0.002	1.26E-08	-0.007	0.009	0.475	32.394	7.01E-05	NA
rs4410790	7	17284577	T	C	0.385	-0.012	0.002	5.72E-09	0.001	0.009	0.880	33.927	7.34E-05	NA
rs4477562	13	54104968	C	T	0.110	-0.020	0.003	9.80E-09	-0.019	0.013	0.164	32.880	7.12E-05	NA
rs4482463	2	205375909	C	A	0.081	0.029	0.004	4.75E-12	-0.018	0.016	0.264	47.787	1.03E-04	NA
rs4660208	1	39722214	A	G	0.195	-0.023	0.003	2.84E-18	0.002	0.011	0.859	75.995	1.64E-04	NA
rs4665991	2	27766284	G	A	0.264	-0.013	0.002	2.16E-09	-0.015	0.010	0.152	35.821	7.75E-05	NA
rs4671328	2	58935282	T	G	0.430	0.018	0.002	9.13E-17	-0.012	0.009	0.205	69.149	1.50E-04	NA
rs4693325	4	94408529	A	C	0.199	-0.016	0.003	1.74E-09	0.011	0.012	0.321	36.241	7.85E-05	NA
rs4740619	9	15634326	T	C	0.481	0.014	0.002	6.33E-11	0.009	0.009	0.291	42.715	9.25E-05	NA
rs4755720	11	43628749	C	T	0.400	0.016	0.002	4.50E-14	0.001	0.009	0.939	56.935	1.23E-04	NA
rs4777542	15	73082366	C	T	0.300	0.014	0.002	3.14E-09	0.011	0.010	0.274	35.098	7.60E-05	NA
rs4790292	17	1824305	C	A	0.144	0.020	0.003	2.66E-10	-0.016	0.013	0.212	39.913	8.64E-05	NA
rs479315	11	66064991	G	C	0.170	-0.016	0.003	2.08E-08	-0.001	0.012	0.966	31.420	6.80E-05	NA
rs4805758	19	32866424	G	A	0.162	0.020	0.003	5.55E-13	-0.007	0.012	0.575	51.999	1.13E-04	Fed-up feelings
rs4810479	20	44545048	C	T	0.288	0.013	0.002	6.17E-10	0.002	0.010	0.854	38.267	8.28E-05	NA
rs4889782	17	78640510	C	T	0.415	0.013	0.002	1.61E-09	0.003	0.009	0.752	36.391	7.88E-05	NA
rs4890439	18	40732586	G	A	0.375	0.012	0.002	4.60E-08	-0.002	0.009	0.852	29.879	6.47E-05	NA
rs4929923	11	8639200	T	C	0.377	-0.015	0.002	4.28E-11	-0.018	0.009	0.058	43.484	9.41E-05	NA
rs4947406	7	50661735	T	G	0.350	0.016	0.002	9.35E-12	-0.002	0.010	0.827	46.461	1.01E-04	NA
rs4971632	2	50240091	T	C	0.166	0.017	0.003	1.26E-09	-0.004	0.012	0.737	36.870	7.98E-05	NA
rs512005	1	33377679	T	C	0.236	0.014	0.003	4.27E-08	-0.001	0.011	0.963	30.024	6.50E-05	NA
rs545608	1	177899121	G	C	0.186	-0.038	0.003	1.86E-37	0.008	0.011	0.449	163.594	3.54E-04	NA
rs563296	10	99772404	G	A	0.441	-0.013	0.002	1.23E-08	0.005	0.009	0.613	32.438	7.02E-05	NA
rs6115015	20	25027692	G	A	0.333	-0.013	0.002	6.30E-09	-0.004	0.010	0.654	33.741	7.30E-05	NA
rs619054	11	116660813	G	A	0.232	0.015	0.002	2.70E-11	0.016	0.011	0.134	44.384	9.61E-05	NA

rs645040	3	135926622	G	T	0.229	-0.023	0.002	1.31E-20	-0.015	0.011	0.172	86.621	1.87E-04	NA
rs6463489	7	5542513	T	C	0.069	0.024	0.004	1.03E-08	0.020	0.015	0.165	32.787	7.10E-05	NA
rs649458	1	23712528	T	A	0.137	0.018	0.003	5.66E-09	0.010	0.013	0.440	33.949	7.35E-05	NA
rs6499240	16	69686912	A	G	0.422	-0.020	0.002	1.52E-18	-0.016	0.009	0.075	77.230	1.67E-04	NA
rs6499861	16	56991495	C	G	0.162	-0.018	0.003	8.61E-12	-0.018	0.013	0.148	46.623	1.01E-04	NA
rs6545714	2	59307725	G	A	0.382	0.019	0.002	3.15E-18	0.009	0.009	0.334	75.796	1.64E-04	NA
rs6548834	3	82704753	G	A	0.378	-0.013	0.002	1.08E-08	0.015	0.009	0.102	32.694	7.08E-05	NA
rs6550597	3	18738940	G	A	0.298	-0.013	0.002	4.61E-09	-0.016	0.010	0.117	34.346	7.43E-05	NA
rs6575340	14	94023972	G	A	0.377	-0.014	0.002	8.82E-10	-0.016	0.009	0.078	37.571	8.13E-05	NA
rs6602151	10	16764483	T	C	0.294	-0.015	0.002	5.89E-10	0.001	0.010	0.928	38.356	8.30E-05	NA
rs6603004	15	84568504	A	G	0.466	-0.013	0.002	1.81E-09	-0.010	0.009	0.242	36.163	7.83E-05	NA
rs6673977	1	7736097	A	G	0.354	-0.013	0.002	1.63E-08	0.012	0.010	0.195	31.892	6.90E-05	NA
rs6711375	2	161090873	G	A	0.320	-0.014	0.002	1.11E-09	-0.018	0.010	0.067	37.115	8.03E-05	NA
rs6754311	2	136707982	T	C	0.489	0.013	0.002	3.95E-10	0.009	0.011	0.405	39.137	8.47E-05	NA
rs676387	17	40706273	C	A	0.266	-0.013	0.002	3.42E-08	0.010	0.010	0.308	30.452	6.59E-05	NA
rs6868198	5	122750714	G	A	0.432	0.013	0.002	2.10E-08	-0.012	0.009	0.176	31.399	6.80E-05	NA
rs687672	11	65649984	T	C	0.189	0.021	0.003	5.92E-14	0.006	0.011	0.583	56.399	1.22E-04	NA
rs6907923	6	51802669	G	A	0.341	-0.015	0.002	1.71E-10	0.008	0.009	0.389	40.774	8.83E-05	NA
rs7016529	8	19806631	T	C	0.011	-0.102	0.009	1.94E-27	-0.012	0.037	0.751	117.778	2.55E-04	NA
rs703965	10	80955067	C	T	0.456	-0.012	0.002	8.25E-09	-0.007	0.010	0.481	33.214	7.19E-05	NA
rs7075195	10	65050659	A	G	0.435	0.017	0.002	5.31E-16	0.010	0.009	0.272	65.676	1.42E-04	Educational attainment
rs7084454	10	21821274	G	A	0.338	-0.016	0.002	9.04E-13	-0.004	0.010	0.651	51.042	1.10E-04	NA
rs7132908	12	50263148	G	A	0.357	-0.019	0.002	9.07E-15	-0.013	0.009	0.171	60.088	1.30E-04	NA
rs7144011	14	79940383	G	T	0.207	-0.023	0.003	1.71E-17	-0.014	0.011	0.195	72.451	1.57E-04	NA
rs7161874	15	53163740	C	G	0.214	-0.015	0.003	4.11E-09	-0.017	0.010	0.096	34.573	7.48E-05	NA
rs7186893	16	24806420	G	T	0.267	0.020	0.002	3.04E-16	0.004	0.010	0.709	66.779	1.45E-04	Mood swings
rs7198357	16	67884619	C	T	0.178	0.017	0.003	4.09E-11	0.003	0.012	0.825	43.573	9.43E-05	NA
rs7206608	16	82872628	C	G	0.313	-0.013	0.002	1.26E-08	0.006	0.010	0.525	32.386	7.01E-05	NA
rs7241918	18	47160953	G	T	0.163	0.018	0.003	7.52E-13	-0.010	0.012	0.411	51.404	1.11E-04	NA
rs7323827	13	94116675	A	G	0.304	0.013	0.002	4.59E-09	0.002	0.010	0.817	34.358	7.44E-05	NA
rs741959	1	47676233	A	G	0.402	0.014	0.002	6.19E-11	0.015	0.009	0.089	42.760	9.26E-05	NA
rs7488867	12	103699685	C	T	0.281	0.015	0.003	1.57E-09	0.004	0.010	0.702	36.447	7.89E-05	NA
rs7515635	1	42408070	T	C	0.471	0.012	0.002	4.68E-09	-0.001	0.009	0.937	34.317	7.43E-05	NA
rs7537581	1	2725475	C	A	0.476	-0.012	0.002	4.55E-08	0.008	0.010	0.382	29.899	6.47E-05	NA
rs7549987	1	80789011	C	T	0.299	-0.014	0.002	4.02E-09	-0.019	0.010	0.055	34.614	7.49E-05	NA
rs7581217	2	25524944	T	C	0.370	0.013	0.002	5.93E-09	0.001	0.010	0.944	33.857	7.33E-05	NA
rs7601028	2	642499	C	G	0.174	-0.046	0.003	1.02E-52	-0.022	0.012	0.068	233.512	5.05E-04	NA
rs7638388	3	47914974	C	T	0.088	-0.024	0.004	1.01E-10	-0.032	0.016	0.042	41.794	9.05E-05	NA
rs7642876	3	15883094	A	G	0.404	0.012	0.002	1.35E-08	0.010	0.009	0.262	32.265	6.98E-05	NA
rs779655	5	170495158	G	C	0.272	-0.014	0.002	1.21E-08	-0.002	0.010	0.811	32.464	7.03E-05	NA
rs7928842	11	47566352	T	C	0.422	-0.020	0.002	2.30E-20	0.004	0.009	0.692	85.511	1.85E-04	NA
rs7947951	11	13356030	A	G	0.297	-0.014	0.002	1.59E-10	-0.001	0.010	0.899	40.911	8.86E-05	NA
rs7962636	12	116450006	T	C	0.355	0.013	0.002	1.32E-08	0.012	0.010	0.208	32.308	6.99E-05	NA
rs7982447	13	54453811	T	C	0.200	-0.019	0.003	6.75E-13	0.003	0.011	0.813	51.616	1.12E-04	NA
rs8015400	14	25930988	C	A	0.348	-0.018	0.002	8.13E-15	-0.008	0.010	0.411	60.303	1.31E-04	NA
rs8055138	16	28891465	C	T	0.329	-0.030	0.002	1.86E-35	0.003	0.009	0.744	154.432	3.34E-04	Educational attainment; Intelligence
rs8078079	17	34959346	T	C	0.395	0.015	0.002	2.89E-11	-0.010	0.009	0.277	44.247	9.58E-05	NA
rs818524	1	85201228	T	C	0.289	-0.013	0.002	2.84E-08	0.011	0.010	0.276	30.812	6.67E-05	NA
rs8192675	3	170724883	T	C	0.289	-0.013	0.002	4.30E-08	-0.008	0.010	0.392	30.007	6.50E-05	NA
rs906673	3	12898941	C	T	0.389	0.013	0.002	3.67E-09	-0.002	0.009	0.818	34.789	7.53E-05	NA
rs9277972	6	33276770	A	T	0.168	-0.016	0.003	1.55E-08	0.020	0.012	0.093	31.995	6.93E-05	NA

rs9563569	13	58571207	G	A	0.150	0.018	0.003	2.09E-08	0.009	0.012	0.429	31.408	6.80E-05		NA
rs9630985	2	181607676	A	C	0.349	-0.014	0.002	9.30E-11	-0.005	0.010	0.598	41.963	9.08E-05		NA
rs9686661	5	55861786	C	T	0.186	-0.023	0.003	4.65E-18	-0.009	0.012	0.454	75.025	1.62E-04		NA
rs9816226	3	185834499	A	T	0.188	-0.016	0.003	8.56E-09	-0.012	0.012	0.317	33.144	7.17E-05		NA
rs9872156	3	176337348	G	A	0.260	-0.013	0.002	4.04E-08	0.010	0.010	0.317	30.129	6.52E-05		NA
rs987237	6	50803050	A	G	0.181	-0.030	0.003	7.81E-26	-0.019	0.012	0.100	110.449	2.39E-04		NA
rs9947450	18	58052665	C	T	0.030	0.062	0.007	1.22E-21	-0.018	0.024	0.451	91.321	1.98E-04		NA
rs998584	6	43757896	C	A	0.496	-0.013	0.002	9.99E-11	0.004	0.010	0.650	41.823	9.05E-05		NA
rs9987289	8	9183358	A	G	0.075	0.022	0.004	1.84E-09	-0.025	0.016	0.121	36.136	7.82E-05		NA

Genomic positions reported in the GWAS of Mets refer to human reference assembly (GRCh37/hg19).

Abbreviations: MetS, Metabolic syndrome; MDD, Major depressive disorder; SNPs, Single nucleotide polymorphisms; CHR, Chromosome; EA, Effect allele; OA, Other allele; EAF, Effect allele frequency; SE, Standard error; PVE, Proportion of phenotypic variance explained.

SNPs that are unavailable in the outcome GWAS:rs1046080, rs705986, rs1412239, among them, rs705986 and rs1412239 were substitute by proxy SNPs: rs2685563 and rs1412235, respectively.

palindromic and not-inferrable SNPs: rs1937433, rs2253310, rs2396625, rs6916318.

1 SNPs were detected by PRESSO outlier test, and 35 SNPs were detected by MR-Radial outlier test.

Supplementary Table 9. Genetic instruments for the causal relationship of Mets and SCZ.

SNP	CHR	Position	EA	OA	EAF	Beta for exposure	SE for exposure	P value for exposure	Beta for outcome	SE for outcome	P value for outcome	F statistics	PVE	Association with potential confounding factors
rs10116353	9	111950088	G	T	0.307	0.013	0.002	9.39E-09	0.011	0.009	0.236	32.964	7.14E-05	NA
rs10160660	11	76474537	T	C	0.248	0.013	0.002	4.83E-08	-0.016	0.010	0.120	29.786	6.45E-05	NA
rs1032524	1	201790826	T	C	0.484	-0.015	0.002	6.01E-12	0.001	0.009	0.909	47.325	1.02E-04	NA
rs1042034	2	21225281	C	T	0.218	-0.020	0.002	7.07E-21	-0.012	0.010	0.244	87.848	1.90E-04	NA
rs10423928	19	46182304	T	A	0.214	0.020	0.003	8.49E-13	0.013	0.011	0.227	51.166	1.11E-04	NA
rs1046080	6	31595882	C	A	0.233	-0.015	0.003	1.75E-09	0.013	0.010	0.204	36.231	7.84E-05	NA
rs10768994	11	43936945	T	C	0.430	0.012	0.002	4.52E-08	0.011	0.009	0.227	29.912	6.48E-05	NA
rs10779835	1	230299949	T	C	0.391	0.015	0.002	2.45E-14	-0.012	0.009	0.160	58.131	1.26E-04	NA
rs10783779	12	56491880	T	G	0.403	0.013	0.002	3.84E-09	0.012	0.009	0.187	34.704	7.51E-05	NA
rs10887579	10	88097210	A	C	0.354	-0.013	0.002	4.09E-09	0.017	0.009	0.064	34.579	7.49E-05	NA
rs11072938	15	81021661	C	T	0.241	0.016	0.002	4.30E-11	-0.012	0.010	0.233	43.470	9.41E-05	NA
rs11150604	16	31037020	A	T	0.389	0.024	0.002	3.91E-28	0.011	0.009	0.230	120.954	2.62E-04	NA
rs11165643	1	96924097	C	T	0.417	-0.013	0.002	6.90E-10	0.014	0.009	0.110	38.049	8.24E-05	NA
rs11187845	10	96060198	C	A	0.096	0.020	0.004	8.08E-09	-0.002	0.015	0.912	33.256	7.20E-05	NA
rs1138429	16	56942921	A	T	0.101	-0.028	0.003	1.42E-18	0.013	0.015	0.377	77.365	1.67E-04	NA
rs1159974	6	126090277	T	C	0.499	-0.013	0.002	3.30E-10	-0.015	0.009	0.076	39.489	8.55E-05	NA
rs11611246	12	939480	G	T	0.183	-0.016	0.003	1.14E-08	-0.020	0.011	0.057	32.583	7.05E-05	NA
rs11636565	15	36391846	C	A	0.488	-0.014	0.002	6.84E-12	-0.010	0.009	0.270	47.072	1.02E-04	NA
rs11660335	18	22154235	T	C	0.161	0.018	0.003	1.07E-09	-0.020	0.011	0.070	37.189	8.05E-05	NA
rs11676272	2	25141538	A	G	0.464	-0.020	0.002	1.05E-17	0.003	0.009	0.729	73.421	1.59E-04	NA
rs11747810	5	153100273	T	C	0.495	0.013	0.002	1.83E-09	0.000	0.009	0.954	36.148	7.83E-05	NA
rs11826999	11	116553778	G	A	0.223	-0.023	0.002	6.07E-26	0.017	0.010	0.099	110.950	2.40E-04	NA
rs11866815	16	387867	C	T	0.242	0.016	0.002	2.32E-10	0.000	0.010	0.999	40.181	8.70E-05	NA
rs11880870	19	18830704	A	G	0.499	0.013	0.002	2.98E-09	0.016	0.009	0.066	35.198	7.62E-05	NA
rs11981190	7	71659538	G	A	0.341	0.013	0.002	5.84E-09	-0.004	0.009	0.630	33.887	7.34E-05	NA
rs1200349	15	41821752	T	C	0.482	-0.013	0.002	1.36E-09	-0.011	0.009	0.196	36.724	7.95E-05	NA
rs12201987	6	143182396	C	T	0.205	0.016	0.003	1.87E-09	0.015	0.011	0.163	36.103	7.82E-05	NA
rs12446554	16	19935073	G	T	0.130	0.020	0.003	3.79E-10	-0.022	0.013	0.084	39.220	8.49E-05	NA
rs12513212	4	96149346	A	C	0.387	-0.014	0.002	2.85E-10	0.000	0.009	0.973	39.778	8.61E-05	NA
rs12596491	16	88928417	C	T	0.340	0.012	0.002	4.58E-08	0.017	0.009	0.068	29.888	6.47E-05	NA
rs12608504	19	18389135	A	G	0.359	0.016	0.002	2.27E-13	0.010	0.009	0.256	53.757	1.16E-04	NA
rs12967135	18	57849023	G	A	0.240	-0.046	0.003	3.09E-66	-0.013	0.010	0.204	295.539	6.39E-04	Worrier or anxious feelings
rs13024601	2	100902032	C	T	0.346	0.014	0.002	1.39E-09	0.010	0.009	0.263	36.680	7.94E-05	NA
rs13206549	6	162978032	T	G	0.134	0.019	0.003	1.54E-09	-0.003	0.013	0.816	36.486	7.90E-05	NA
rs13250456	8	72408720	C	T	0.055	-0.025	0.005	4.67E-08	-0.010	0.018	0.578	29.850	6.46E-05	NA
rs13275593	8	68257300	G	A	0.285	-0.015	0.002	2.14E-10	-0.003	0.010	0.718	40.332	8.73E-05	NA
rs13416004	2	69664976	C	T	0.415	0.012	0.002	3.16E-08	-0.012	0.009	0.171	30.605	6.63E-05	NA
rs13416667	2	56608267	C	A	0.274	-0.015	0.002	2.32E-10	-0.018	0.010	0.072	40.179	8.70E-05	NA
rs13435860	4	136975545	A	T	0.368	-0.013	0.002	1.94E-09	0.010	0.009	0.273	36.037	7.80E-05	NA
rs1362910	8	30856464	A	G	0.386	0.014	0.002	9.70E-11	-0.006	0.009	0.467	41.882	9.07E-05	NA
rs1363695	5	130378027	C	T	0.228	0.015	0.003	6.49E-09	-0.006	0.010	0.544	33.681	7.29E-05	NA
rs1421085	16	53800954	T	C	0.432	-0.062	0.002	5.07E-152	0.002	0.009	0.777	689.747	1.49E-03	NA
rs1431659	8	73439070	A	G	0.278	0.019	0.002	4.48E-15	-0.019	0.010	0.056	61.476	1.33E-04	NA
rs1448613	3	85875265	G	A	0.386	0.014	0.002	2.40E-10	-0.013	0.009	0.156	40.110	8.68E-05	NA
rs1456116	3	123084379	G	A	0.417	-0.013	0.002	1.99E-09	-0.006	0.009	0.506	35.987	7.79E-05	NA
rs1458156	12	41887940	C	T	0.449	-0.013	0.002	4.45E-09	0.015	0.009	0.074	34.416	7.45E-05	NA
rs1507153	6	79484386	C	A	0.364	-0.013	0.002	3.86E-09	-0.001	0.009	0.905	34.694	7.51E-05	NA
rs1513475	3	35724256	T	C	0.343	0.015	0.002	8.46E-11	-0.007	0.009	0.454	42.149	9.12E-05	NA
rs1534696	7	26397239	C	A	0.439	0.014	0.002	1.24E-11	-0.004	0.009	0.631	45.905	9.94E-05	NA

rs17207196	7	75101065	C	T	0.444	0.018	0.002	2.68E-16	0.008	0.009	0.389	67.024	1.45E-04	NA
rs17513613	19	30286822	T	C	0.289	-0.017	0.002	1.94E-13	0.006	0.009	0.486	54.063	1.17E-04	NA
rs17618704	17	46221010	T	G	0.068	0.025	0.004	1.52E-09	0.013	0.017	0.432	36.510	7.90E-05	NA
rs17639546	3	61251635	G	A	0.148	0.018	0.003	3.65E-09	-0.023	0.012	0.057	34.803	7.53E-05	NA
rs17694506	2	171632225	C	T	0.388	0.014	0.002	3.33E-11	0.013	0.009	0.158	43.971	9.52E-05	NA
rs17806379	20	51107290	C	T	0.185	0.020	0.003	3.64E-13	0.004	0.011	0.757	52.829	1.14E-04	NA
rs17821316	15	58703069	A	C	0.488	-0.012	0.002	9.97E-10	0.008	0.009	0.377	37.331	8.08E-05	NA
rs1784460	11	118938371	T	A	0.360	-0.015	0.002	8.15E-12	0.004	0.009	0.623	46.729	1.01E-04	NA
rs1914888	17	21267541	A	G	0.462	0.013	0.002	1.33E-09	0.003	0.009	0.715	36.762	7.96E-05	NA
rs2002023	10	76848524	C	T	0.441	-0.012	0.002	8.92E-09	-0.006	0.009	0.501	33.064	7.16E-05	NA
rs2035831	3	173683791	G	C	0.321	0.013	0.002	1.48E-08	0.001	0.009	0.899	32.085	6.95E-05	NA
rs2060604	8	76650334	T	C	0.414	0.015	0.002	2.80E-12	0.004	0.009	0.652	48.822	1.06E-04	NA
rs2074881	19	1970021	C	T	0.179	0.015	0.003	2.25E-08	0.013	0.012	0.297	31.270	6.77E-05	NA
rs2075226	2	36805676	A	G	0.396	-0.013	0.002	2.48E-08	0.012	0.009	0.197	31.073	6.73E-05	NA
rs208015	17	46252346	T	C	0.088	0.022	0.004	1.07E-08	0.006	0.016	0.703	32.711	7.08E-05	NA
rs2180454	14	29690513	T	C	0.241	-0.015	0.003	1.90E-09	-0.015	0.010	0.148	36.071	7.81E-05	NA
rs2237717	7	116405387	T	C	0.443	-0.011	0.002	2.63E-08	0.002	0.009	0.839	30.960	6.70E-05	NA
rs227199	1	210265384	G	C	0.316	0.013	0.002	5.06E-09	-0.008	0.009	0.390	34.168	7.40E-05	NA
rs2272168	7	99102777	G	C	0.163	0.017	0.003	3.68E-09	-0.017	0.012	0.151	34.785	7.53E-05	NA
rs2275543	9	107651174	T	C	0.099	-0.019	0.003	6.58E-09	-0.021	0.014	0.137	33.655	7.29E-05	NA
rs2298969	4	3186244	A	G	0.490	0.012	0.002	2.79E-08	0.005	0.009	0.590	30.851	6.68E-05	NA
rs2299383	7	103418846	C	T	0.410	-0.014	0.002	2.20E-10	0.009	0.009	0.327	40.282	8.72E-05	NA
rs2307111	5	75003678	T	C	0.396	0.025	0.002	4.41E-29	-0.012	0.009	0.167	125.285	2.71E-04	NA
rs2436726	6	40366427	C	T	0.405	-0.016	0.002	2.24E-12	0.008	0.009	0.379	49.262	1.07E-04	NA
rs2516740	16	2097110	A	C	0.226	0.020	0.003	7.50E-14	-0.001	0.011	0.945	55.934	1.21E-04	NA
rs2531686	10	118651011	C	G	0.276	0.015	0.002	2.22E-10	0.006	0.010	0.555	40.261	8.72E-05	NA
rs2531995	16	4013467	C	T	0.387	-0.019	0.002	2.80E-17	-0.006	0.009	0.485	71.477	1.55E-04	NA
rs2596933	3	9365746	G	A	0.267	0.014	0.002	1.26E-08	0.013	0.010	0.191	32.396	7.01E-05	NA
rs2781668	6	131897278	C	T	0.170	-0.015	0.003	3.64E-08	-0.015	0.012	0.209	30.331	6.57E-05	NA
rs2820232	6	35003603	G	T	0.459	-0.012	0.002	2.95E-08	-0.003	0.009	0.697	30.741	6.65E-05	NA
rs2861688	2	67838474	T	C	0.273	0.016	0.002	8.97E-11	-0.006	0.010	0.532	42.033	9.10E-05	NA
rs2925979	16	81534790	T	C	0.288	0.014	0.002	1.10E-10	0.018	0.010	0.057	41.628	9.01E-05	NA
rs2968856	7	150614934	T	C	0.261	0.016	0.002	1.80E-11	0.001	0.010	0.904	45.179	9.78E-05	NA
rs2990997	1	190119703	T	A	0.353	-0.012	0.002	3.24E-08	0.010	0.009	0.275	30.558	6.62E-05	NA
rs320	8	19819077	T	G	0.287	0.031	0.002	2.30E-43	0.007	0.010	0.485	190.642	4.13E-04	NA
rs323781	5	86752616	A	C	0.034	-0.032	0.006	3.82E-08	-0.014	0.025	0.570	30.237	6.55E-05	NA
rs349088	11	84814393	C	A	0.480	0.013	0.002	3.23E-09	0.013	0.009	0.149	35.040	7.59E-05	NA
rs3749262	3	131624097	G	A	0.263	-0.019	0.002	1.12E-14	0.011	0.010	0.253	59.681	1.29E-04	NA
rs3814424	5	87968953	C	T	0.172	-0.025	0.003	2.55E-16	-0.020	0.012	0.091	67.124	1.45E-04	Intelligence
rs3852012	3	44045981	G	A	0.287	0.013	0.002	1.57E-08	-0.007	0.009	0.476	31.963	6.92E-05	NA
rs3864004	3	41240177	G	A	0.432	-0.014	0.002	2.30E-09	0.013	0.009	0.138	35.700	7.73E-05	NA
rs3902840	9	129419025	G	A	0.092	-0.021	0.004	1.72E-08	-0.024	0.015	0.113	31.786	6.88E-05	NA
rs397092	21	46582564	A	G	0.456	-0.013	0.002	1.07E-09	0.000	0.009	0.981	37.196	8.05E-05	NA
rs40067	5	107439012	G	A	0.180	0.018	0.003	4.05E-11	0.001	0.011	0.949	43.590	9.44E-05	NA
rs4074448	15	63888495	G	A	0.434	0.012	0.002	2.37E-08	0.002	0.009	0.825	31.161	6.75E-05	NA
rs4377779	6	12117344	T	C	0.334	0.013	0.002	5.27E-09	0.012	0.009	0.197	34.086	7.38E-05	NA
rs4410790	7	17284577	T	C	0.385	-0.012	0.002	5.72E-09	0.002	0.009	0.841	33.927	7.34E-05	NA
rs442177	4	88030261	G	T	0.421	-0.011	0.002	3.89E-08	0.001	0.009	0.941	30.204	6.54E-05	NA
rs4477562	13	54104968	C	T	0.110	-0.020	0.003	9.80E-09	0.002	0.013	0.855	32.880	7.12E-05	NA
rs4482463	2	205375909	C	A	0.081	0.029	0.004	4.75E-12	0.016	0.016	0.324	47.787	1.03E-04	NA
rs4665991	2	27766284	G	A	0.264	-0.013	0.002	2.16E-09	-0.008	0.010	0.389	35.821	7.75E-05	NA

rs4693325	4	94408529	A	C	0.199	-0.016	0.003	1.74E-09	0.021	0.011	0.053	36.241	7.85E-05	NA
rs4740619	9	15634326	T	C	0.481	0.014	0.002	6.33E-11	-0.006	0.009	0.468	42.715	9.25E-05	NA
rs4755720	11	43628749	C	T	0.400	0.016	0.002	4.50E-14	0.002	0.009	0.826	56.935	1.23E-04	NA
rs4777542	15	73082366	C	T	0.300	0.014	0.002	3.14E-09	0.003	0.009	0.751	35.098	7.60E-05	NA
rs4784745	16	57014875	A	G	0.329	-0.019	0.002	4.00E-21	-0.003	0.009	0.790	88.973	1.93E-04	NA
rs479315	11	66064991	G	C	0.170	-0.016	0.003	2.08E-08	0.009	0.011	0.424	31.420	6.80E-05	NA
rs4805758	19	32866424	G	A	0.162	0.020	0.003	5.55E-13	-0.010	0.012	0.396	51.999	1.13E-04	Fed-up feelings
rs4810479	20	44545048	C	T	0.288	0.013	0.002	6.17E-10	0.011	0.010	0.250	38.267	8.28E-05	NA
rs4890439	18	40732586	G	A	0.375	0.012	0.002	4.60E-08	-0.006	0.009	0.517	29.879	6.47E-05	NA
rs4929923	11	8639200	T	C	0.377	-0.015	0.002	4.28E-11	-0.006	0.009	0.504	43.484	9.41E-05	NA
rs4947406	7	50661735	T	G	0.350	0.016	0.002	9.35E-12	-0.017	0.009	0.072	46.461	1.01E-04	NA
rs545608	1	177899121	G	C	0.186	-0.038	0.003	1.86E-37	0.000	0.011	0.989	163.594	3.54E-04	NA
rs563296	10	99772404	G	A	0.441	-0.013	0.002	1.23E-08	0.010	0.009	0.237	32.438	7.02E-05	NA
rs6115015	20	25027692	G	A	0.333	-0.013	0.002	6.30E-09	0.003	0.009	0.729	33.741	7.30E-05	NA
rs619054	11	116660813	G	A	0.232	0.015	0.002	2.70E-11	-0.009	0.010	0.380	44.384	9.61E-05	NA
rs649458	1	23712528	T	A	0.137	0.018	0.003	5.66E-09	-0.015	0.013	0.225	33.949	7.35E-05	NA
rs6499240	16	69686912	A	G	0.422	-0.020	0.002	1.52E-18	-0.003	0.009	0.692	77.230	1.67E-04	NA
rs6499861	16	56991495	C	G	0.162	-0.018	0.003	8.61E-12	0.002	0.012	0.876	46.623	1.01E-04	NA
rs6545714	2	59307725	G	A	0.382	0.019	0.002	3.15E-18	0.005	0.009	0.552	75.796	1.64E-04	NA
rs6548834	3	82704753	G	A	0.378	-0.013	0.002	1.08E-08	0.010	0.009	0.288	32.694	7.08E-05	NA
rs6550597	3	18738940	G	A	0.298	-0.013	0.002	4.61E-09	-0.003	0.010	0.723	34.346	7.43E-05	NA
rs6602151	10	16764483	T	C	0.294	-0.015	0.002	5.89E-10	0.007	0.010	0.439	38.356	8.30E-05	NA
rs6673977	1	7736097	A	G	0.354	-0.012	0.002	1.63E-08	-0.005	0.009	0.573	31.892	6.90E-05	NA
rs6711375	2	161090873	G	A	0.320	-0.014	0.002	1.11E-09	-0.008	0.009	0.413	37.115	8.03E-05	NA
rs6754311	2	136707982	T	C	0.489	0.013	0.002	3.95E-10	-0.011	0.010	0.276	39.137	8.47E-05	NA
rs676387	17	40706273	C	A	0.266	-0.013	0.002	3.42E-08	0.005	0.010	0.616	30.452	6.59E-05	NA
rs6868198	5	122750714	G	A	0.432	0.013	0.002	2.10E-08	-0.001	0.009	0.878	31.399	6.80E-05	NA
rs6907923	6	51802669	G	A	0.341	-0.015	0.002	1.71E-10	0.007	0.009	0.419	40.774	8.83E-05	NA
rs7016529	8	19806631	T	C	0.011	-0.102	0.009	1.94E-27	0.012	0.036	0.733	117.778	2.55E-04	NA
rs703965	10	80955067	C	T	0.456	-0.012	0.002	8.25E-09	0.008	0.009	0.373	33.214	7.19E-05	NA
rs7075195	10	65050659	A	G	0.435	0.017	0.002	5.31E-16	0.000	0.009	0.970	65.676	1.42E-04	Educational attainment
rs7084454	10	21821274	G	A	0.338	-0.016	0.002	9.04E-13	0.009	0.009	0.339	51.042	1.10E-04	NA
rs7132908	12	50263148	G	A	0.357	-0.019	0.002	9.07E-15	0.005	0.009	0.546	60.088	1.30E-04	NA
rs7144011	14	79940383	G	T	0.207	-0.023	0.003	1.71E-17	-0.019	0.011	0.070	72.451	1.57E-04	NA
rs7161874	15	53163740	C	G	0.214	-0.015	0.003	4.11E-09	-0.006	0.010	0.551	34.573	7.48E-05	NA
rs7186893	16	24806420	G	T	0.267	0.020	0.002	3.04E-16	-0.013	0.010	0.174	66.779	1.45E-04	Mood swings
rs7212293	17	65847325	A	G	0.216	-0.022	0.003	4.05E-17	-0.007	0.011	0.510	70.753	1.53E-04	NA
rs7241918	18	47160953	G	T	0.163	0.018	0.003	7.52E-13	-0.007	0.012	0.566	51.403	1.11E-04	NA
rs741959	1	47676233	A	G	0.402	0.014	0.002	6.19E-11	-0.008	0.009	0.337	42.760	9.26E-05	NA
rs7515635	1	42408070	T	C	0.471	0.012	0.002	4.68E-09	0.009	0.009	0.288	34.317	7.43E-05	NA
rs7537581	1	2725475	C	A	0.476	-0.012	0.002	4.55E-08	-0.006	0.009	0.504	29.899	6.47E-05	NA
rs7549987	1	80789011	C	T	0.299	-0.014	0.002	4.02E-09	-0.015	0.010	0.112	34.614	7.49E-05	NA
rs7581217	2	25524944	T	C	0.370	0.013	0.002	5.93E-09	-0.014	0.009	0.144	33.857	7.33E-05	NA
rs7638388	3	47914974	C	T	0.088	-0.024	0.004	1.01E-10	0.009	0.016	0.557	41.794	9.05E-05	NA
rs7642876	3	15883094	A	G	0.404	0.012	0.002	1.35E-08	-0.009	0.009	0.318	32.265	6.98E-05	NA
rs779655	5	170495158	G	C	0.272	-0.014	0.002	1.21E-08	-0.006	0.010	0.534	32.464	7.03E-05	NA
rs7928842	11	47566352	T	C	0.422	-0.020	0.002	2.30E-20	0.000	0.009	0.997	85.511	1.85E-04	NA
rs7982447	13	54453811	T	C	0.200	-0.019	0.003	6.75E-13	-0.010	0.011	0.343	51.616	1.12E-04	NA
rs8015400	14	25930988	C	A	0.348	-0.018	0.002	8.13E-15	0.013	0.009	0.158	60.303	1.31E-04	NA
rs808820	12	89744826	A	G	0.116	-0.022	0.004	8.37E-10	0.015	0.014	0.292	37.671	8.15E-05	NA
rs818524	1	85201228	T	C	0.289	-0.013	0.002	2.84E-08	0.003	0.010	0.722	30.812	6.67E-05	NA

rs891387	18	21103909	T	C	0.473	0.021	0.002	3.25E-21	0.002	0.009	0.791	89.383	1.93E-04	Educational attainment
rs906673	3	12898941	C	T	0.389	0.013	0.002	3.67E-09	0.003	0.009	0.720	34.789	7.53E-05	NA
rs9556979	13	99241507	T	G	0.324	-0.013	0.002	4.53E-09	0.004	0.010	0.672	34.382	7.44E-05	NA
rs9630985	2	181607676	A	C	0.349	-0.014	0.002	9.30E-11	0.001	0.009	0.929	41.963	9.08E-05	NA
rs9686661	5	55861786	C	T	0.186	-0.023	0.003	4.65E-18	-0.006	0.011	0.571	75.025	1.62E-04	NA
rs9816226	3	185834499	A	T	0.188	-0.016	0.003	8.56E-09	-0.021	0.011	0.069	33.144	7.17E-05	NA
rs9872156	3	176337348	G	A	0.260	-0.013	0.002	4.04E-08	0.013	0.010	0.191	30.129	6.52E-05	NA
rs987237	6	50803050	A	G	0.181	-0.030	0.003	7.81E-26	0.015	0.011	0.172	110.449	2.39E-04	NA
rs9947450	18	58052665	C	T	0.030	0.062	0.007	1.22E-21	0.007	0.023	0.763	91.321	1.98E-04	NA
rs998584	6	43757896	C	A	0.496	-0.013	0.002	9.99E-11	0.004	0.009	0.684	41.823	9.05E-05	NA

Genomic positions reported in the GWAS of Mets refer to human reference assembly (GRCh37/hg19).

Abbreviations: MetS, Metabolic syndrome; SCZ, Schizophrenia; SNPs, Single nucleotide polymorphisms; CHR, Chromosome; EA, Effect allele; OA, Other allele; EAF, Effect allele frequency; SE, Standard error; PVE, Proportion of phenotypic variance explained.

palindromic and not-inferrable SNPs: rs1937433, rs2253310, rs2396625, rs6916318.

21 SNPs were detected by PRESSO outlier test, and 66 SNPs were detected by MR-Radial outlier test.

Supplementary Table 10. Genetic instruments for the causal relationship of Mets and PTSD.

SNP	CHR	Position	EA	OA	EAF	Beta for exposure	SE for exposure	P value for exposure	Beta for outcome	SE for outcome	P value for outcome	F statistics	PVE	Association with potential confounding factors
rs10116353	9	111950088	G	T	0.307	0.013	0.002	9.39E-09	0.014	0.041	0.738	32.964	7.14E-05	NA
rs1013402	11	27712381	A	G	0.290	-0.025	0.002	1.34E-23	-0.025	0.041	0.541	100.255	2.17E-04	NA
rs10160660	11	76474537	T	C	0.248	0.013	0.002	4.83E-08	-0.036	0.046	0.426	29.786	6.45E-05	NA
rs102275	11	61557803	T	C	0.363	-0.013	0.002	6.28E-11	0.021	0.040	0.593	42.731	9.25E-05	NA
rs1032524	1	201790826	T	C	0.484	-0.015	0.002	6.01E-12	0.025	0.038	0.505	47.325	1.02E-04	NA
rs1042034	2	21225281	C	T	0.218	-0.020	0.002	7.07E-21	0.012	0.046	0.794	87.848	1.90E-04	NA
rs10423928	19	46182304	T	A	0.214	0.020	0.003	8.49E-13	-0.042	0.047	0.376	51.166	1.11E-04	NA
rs1046080	6	31595882	C	A	0.233	-0.015	0.003	1.75E-09	-0.001	0.127	0.996	36.231	7.84E-05	NA
rs10734924	12	123018894	T	G	0.259	-0.015	0.003	2.19E-09	-0.084	0.043	0.050	35.801	7.75E-05	NA
rs10768994	11	43936945	T	C	0.430	0.012	0.002	4.52E-08	-0.057	0.038	0.136	29.912	6.48E-05	NA
rs10779835	1	230299949	T	C	0.391	0.015	0.002	2.45E-14	-0.019	0.039	0.629	58.131	1.26E-04	NA
rs10783779	12	56491880	T	G	0.403	0.013	0.002	3.84E-09	-0.054	0.039	0.170	34.704	7.51E-05	NA
rs10808546	8	126495818	C	T	0.431	0.016	0.002	4.56E-19	-0.001	0.039	0.988	79.612	1.72E-04	NA
rs10838620	11	46818098	G	A	0.214	-0.015	0.002	1.25E-09	-0.023	0.045	0.614	36.883	7.98E-05	NA
rs10872224	6	98435125	G	T	0.409	-0.014	0.002	9.76E-11	-0.049	0.039	0.205	41.868	9.06E-05	Educational attainment; Intelligence
rs10887579	10	88097210	A	C	0.354	-0.013	0.002	4.09E-09	-0.059	0.043	0.163	34.579	7.49E-05	NA
rs10930502	2	172890588	A	G	0.304	0.013	0.002	1.18E-08	0.065	0.041	0.115	32.519	7.04E-05	NA
rs10938397	4	45182527	A	G	0.420	-0.023	0.002	8.14E-26	0.016	0.038	0.669	110.367	2.39E-04	NA
rs11072938	15	81021661	C	T	0.241	0.016	0.002	4.30E-11	0.035	0.043	0.418	43.470	9.41E-05	NA
rs11079849	17	47090785	C	T	0.290	0.015	0.002	6.90E-11	0.001	0.044	0.980	42.547	9.21E-05	NA
rs11150604	16	31037020	A	T	0.389	0.024	0.002	3.91E-28	0.011	0.039	0.772	120.954	2.62E-04	NA
rs11165643	1	96924097	C	T	0.417	-0.013	0.002	6.90E-10	-0.028	0.039	0.471	38.049	8.24E-05	NA
rs11187845	10	96060198	C	A	0.096	0.020	0.004	8.08E-09	0.024	0.063	0.706	33.256	7.20E-05	NA
rs1138429	16	56942921	A	T	0.101	-0.028	0.003	1.42E-18	0.000	0.069	0.998	77.365	1.67E-04	NA
rs1159974	6	126090277	T	C	0.499	-0.013	0.002	3.30E-10	0.018	0.038	0.632	39.489	8.55E-05	NA
rs11611246	12	939480	G	T	0.183	-0.016	0.003	1.14E-08	-0.047	0.050	0.346	32.583	7.05E-05	NA
rs11631408	15	51746236	A	G	0.463	0.014	0.002	2.44E-11	0.002	0.041	0.965	44.582	9.65E-05	NA
rs11636565	15	36391846	C	A	0.488	-0.014	0.002	6.84E-12	-0.042	0.038	0.266	47.072	1.02E-04	NA
rs11660335	18	22154235	T	C	0.161	0.018	0.003	1.07E-09	-0.044	0.047	0.356	37.189	8.05E-05	NA
rs11676272	2	25141538	A	G	0.464	-0.020	0.002	1.05E-17	-0.018	0.041	0.669	73.421	1.59E-04	NA
rs11747810	5	153100273	T	C	0.495	0.013	0.002	1.83E-09	-0.040	0.041	0.333	36.148	7.83E-05	NA
rs11766944	7	1888051	G	A	0.214	0.015	0.003	4.17E-08	0.091	0.048	0.061	30.068	6.51E-05	NA
rs11826999	11	116553778	G	A	0.223	-0.023	0.002	6.07E-26	0.014	0.045	0.761	110.950	2.40E-04	NA
rs11866815	16	387867	C	T	0.242	0.016	0.002	2.32E-10	-0.021	0.043	0.624	40.181	8.70E-05	NA
rs11880870	19	18830704	A	G	0.499	0.013	0.002	2.98E-09	-0.020	0.041	0.631	35.198	7.62E-05	NA
rs11981190	7	71659538	G	A	0.341	0.013	0.002	5.84E-09	-0.015	0.044	0.737	33.887	7.34E-05	NA
rs1200349	15	41821752	T	C	0.482	-0.013	0.002	1.36E-09	-0.044	0.038	0.247	36.724	7.95E-05	NA
rs12033257	1	112318484	A	G	0.382	0.014	0.002	8.97E-10	-0.001	0.045	0.989	37.538	8.13E-05	NA
rs12201987	6	143182396	C	T	0.205	0.016	0.003	1.87E-09	0.055	0.049	0.263	36.103	7.82E-05	NA
rs12513212	4	96149346	A	C	0.387	-0.014	0.002	2.85E-10	0.030	0.038	0.443	39.778	8.61E-05	NA
rs12520822	5	105699867	C	T	0.408	0.012	0.002	1.84E-08	-0.013	0.041	0.754	31.658	6.85E-05	NA
rs12596491	16	88928417	C	T	0.340	0.012	0.002	4.58E-08	0.028	0.043	0.522	29.888	6.47E-05	NA
rs12608504	19	18389135	A	G	0.359	0.016	0.002	2.27E-13	-0.030	0.039	0.449	53.757	1.16E-04	NA
rs12624640	20	32952125	G	A	0.430	-0.012	0.002	7.17E-09	0.041	0.039	0.284	33.489	7.25E-05	NA
rs12824567	12	124495203	G	C	0.350	0.013	0.002	1.94E-09	0.038	0.044	0.390	36.032	7.80E-05	NA
rs12981256	19	1865901	G	A	0.475	-0.016	0.002	7.75E-14	0.004	0.041	0.930	55.867	1.21E-04	NA
rs13024601	2	100902032	C	T	0.346	0.014	0.002	1.39E-09	-0.038	0.039	0.333	36.680	7.94E-05	NA
rs13029936	2	208465544	C	T	0.176	0.017	0.003	1.31E-09	-0.020	0.048	0.675	36.795	7.96E-05	NA
rs13107325	4	103188709	C	T	0.080	-0.023	0.004	1.94E-09	-0.083	0.077	0.285	36.033	7.80E-05	Intelligence

rs13206549	6	162978032	T	G	0.134	0.019	0.003	1.54E-09	-0.026	0.056	0.649	36.486	7.90E-05	NA
rs13244268	7	72911843	T	C	0.114	0.024	0.003	9.17E-18	-0.034	0.060	0.563	73.684	1.59E-04	NA
rs13250456	8	72408720	C	T	0.055	-0.025	0.005	4.67E-08	0.059	0.095	0.536	29.850	6.46E-05	NA
rs13275593	8	68257300	G	A	0.285	-0.015	0.002	2.14E-10	-0.053	0.046	0.249	40.332	8.73E-05	NA
rs1330304	9	16714629	C	G	0.267	0.013	0.002	2.32E-08	0.031	0.047	0.504	31.202	6.75E-05	NA
rs13322435	3	156795468	A	G	0.407	0.020	0.002	6.60E-20	-0.008	0.042	0.841	83.432	1.81E-04	NA
rs13416004	2	69664976	C	T	0.415	0.012	0.002	3.16E-08	0.013	0.038	0.743	30.605	6.63E-05	NA
rs13416667	2	56608267	C	A	0.274	-0.015	0.002	2.32E-10	-0.044	0.046	0.346	40.179	8.70E-05	NA
rs13435860	4	136975545	A	T	0.368	-0.013	0.002	1.94E-09	-0.032	0.039	0.419	36.037	7.80E-05	NA
rs1353580	15	47738732	T	A	0.374	-0.012	0.002	1.70E-08	0.012	0.039	0.752	31.810	6.89E-05	NA
rs1362910	8	30856464	A	G	0.386	0.014	0.002	9.70E-11	-0.017	0.038	0.659	41.882	9.07E-05	NA
rs1363695	5	130378027	C	T	0.228	0.015	0.003	6.49E-09	0.014	0.047	0.763	33.681	7.29E-05	NA
rs1421085	16	53800954	T	C	0.432	-0.062	0.002	5.07E-152	0.000	0.039	0.992	689.747	1.49E-03	NA
rs1431659	8	73439070	A	G	0.278	0.019	0.002	4.48E-15	-0.009	0.042	0.832	61.476	1.33E-04	NA
rs1448613	3	85875265	G	A	0.386	0.014	0.002	2.40E-10	-0.029	0.040	0.465	40.110	8.68E-05	NA
rs1452075	3	62481063	C	T	0.284	-0.014	0.002	9.96E-09	0.028	0.042	0.509	32.850	7.11E-05	NA
rs1456116	3	123084379	G	A	0.417	-0.013	0.002	1.99E-09	-0.067	0.038	0.076	35.987	7.79E-05	NA
rs1458156	12	41887940	C	T	0.449	-0.013	0.002	4.45E-09	-0.026	0.037	0.484	34.416	7.45E-05	NA
rs1460940	1	72814617	G	A	0.182	-0.020	0.003	2.89E-13	0.019	0.050	0.700	53.284	1.15E-04	NA
rs1507153	6	79484386	C	A	0.364	-0.013	0.002	3.86E-09	-0.011	0.039	0.783	34.694	7.51E-05	NA
rs1513475	3	35724256	T	C	0.343	0.015	0.002	8.46E-11	0.009	0.039	0.817	42.149	9.12E-05	NA
rs1534696	7	26397239	C	A	0.439	0.014	0.002	1.24E-11	-0.043	0.043	0.323	45.905	9.94E-05	NA
rs1580099	3	94003603	C	A	0.406	0.014	0.002	4.35E-09	0.037	0.038	0.327	34.459	7.46E-05	NA
rs17024393	1	110154688	T	C	0.033	-0.048	0.006	1.96E-15	0.078	0.114	0.495	63.106	1.37E-04	NA
rs17115145	14	30122409	C	T	0.406	-0.012	0.002	1.29E-08	-0.025	0.039	0.519	32.350	7.00E-05	NA
rs17162333	1	27236757	C	G	0.132	-0.020	0.003	6.24E-11	0.051	0.052	0.328	42.744	9.25E-05	NA
rs17207196	7	75101065	C	T	0.444	0.018	0.002	2.68E-16	-0.037	0.043	0.386	67.024	1.45E-04	NA
rs1730858	1	107619244	T	C	0.328	0.015	0.002	1.04E-10	-0.029	0.044	0.503	41.736	9.03E-05	NA
rs17391694	1	78623626	C	T	0.096	-0.026	0.004	2.39E-12	-0.063	0.066	0.343	49.132	1.06E-04	NA
rs17513613	19	30286822	T	C	0.289	-0.017	0.002	1.94E-13	0.055	0.043	0.209	54.063	1.17E-04	NA
rs17618704	17	46221010	T	G	0.068	0.025	0.004	1.52E-09	0.143	0.077	0.062	36.510	7.90E-05	NA
rs17639546	3	61251635	G	A	0.148	0.018	0.003	3.65E-09	-0.047	0.054	0.385	34.803	7.53E-05	NA
rs17694506	2	171632225	C	T	0.388	0.014	0.002	3.33E-11	-0.051	0.039	0.193	43.971	9.52E-05	NA
rs17724992	19	18454825	A	G	0.245	0.014	0.002	8.13E-09	-0.024	0.043	0.577	33.243	7.20E-05	NA
rs17783751	16	89513984	C	A	0.176	-0.015	0.003	4.79E-08	0.049	0.047	0.301	29.800	6.45E-05	NA
rs17806379	20	51107290	C	T	0.185	0.020	0.003	3.64E-13	0.018	0.050	0.715	52.829	1.14E-04	NA
rs17821316	15	58703069	A	C	0.488	-0.012	0.002	9.97E-10	0.045	0.042	0.285	37.331	8.08E-05	NA
rs1784460	11	118938371	T	A	0.360	-0.015	0.002	8.15E-12	0.042	0.039	0.271	46.729	1.01E-04	NA
rs1876359	16	4930100	C	T	0.377	-0.013	0.002	5.40E-09	-0.006	0.039	0.883	34.038	7.37E-05	NA
rs1886558	13	111982615	A	G	0.389	0.012	0.002	4.14E-08	0.027	0.042	0.518	30.082	6.51E-05	NA
rs1899689	7	121964349	C	T	0.380	-0.012	0.002	4.27E-08	0.015	0.039	0.699	30.021	6.50E-05	NA
rs1914888	17	21267541	A	G	0.462	0.013	0.002	1.33E-09	-0.008	0.042	0.852	36.762	7.96E-05	NA
rs192812	3	161491831	C	T	0.454	-0.012	0.002	4.87E-09	-0.082	0.041	0.047	34.241	7.41E-05	NA
rs1945941	11	132620118	G	A	0.376	0.012	0.002	3.63E-08	-0.015	0.042	0.713	30.337	6.57E-05	NA
rs2002023	10	76848524	C	T	0.441	-0.012	0.002	8.92E-09	-0.030	0.038	0.425	33.064	7.16E-05	NA
rs2015407	14	103274547	A	G	0.345	0.014	0.002	4.68E-10	0.044	0.040	0.270	38.808	8.40E-05	NA
rs2035831	3	173683791	G	C	0.321	0.013	0.002	1.48E-08	-0.009	0.040	0.823	32.085	6.95E-05	NA
rs2060604	8	76650334	T	C	0.414	0.015	0.002	2.80E-12	0.020	0.038	0.612	48.822	1.06E-04	NA
rs2065418	11	30422068	T	G	0.354	0.016	0.002	1.39E-12	0.017	0.039	0.668	50.196	1.09E-04	NA
rs2066295	6	26168903	A	G	0.219	0.023	0.003	5.73E-18	0.003	0.044	0.948	74.611	1.61E-04	NA
rs2074881	19	1970021	C	T	0.179	0.015	0.003	2.25E-08	-0.021	0.056	0.702	31.270	6.77E-05	NA

rs2075226	2	36805676	A	G	0.396	-0.013	0.002	2.48E-08	0.012	0.039	0.766	31.073	6.73E-05	NA
rs208015	17	46252346	T	C	0.088	0.022	0.004	1.07E-08	-0.033	0.077	0.668	32.711	7.08E-05	NA
rs2143253	20	41987392	G	A	0.137	0.019	0.003	1.29E-09	0.044	0.059	0.455	36.823	7.97E-05	NA
rs2180454	14	29690513	T	C	0.241	-0.015	0.003	1.90E-09	0.039	0.045	0.386	36.071	7.81E-05	NA
rs2237717	7	116405387	T	C	0.443	-0.011	0.002	2.63E-08	-0.074	0.038	0.054	30.960	6.70E-05	NA
rs2242449	17	7095507	C	T	0.417	-0.013	0.002	2.85E-09	0.050	0.042	0.236	35.287	7.64E-05	NA
rs227199	1	210265384	G	C	0.316	0.013	0.002	5.06E-09	-0.005	0.043	0.915	34.168	7.40E-05	NA
rs2272168	7	99102777	G	C	0.163	0.017	0.003	3.68E-09	-0.047	0.057	0.413	34.785	7.53E-05	NA
rs2275543	9	107651174	T	C	0.099	-0.019	0.003	6.58E-09	-0.089	0.062	0.154	33.655	7.29E-05	NA
rs2298969	4	3186244	A	G	0.490	0.012	0.002	2.79E-08	0.010	0.038	0.797	30.851	6.68E-05	NA
rs2299383	7	103418846	C	T	0.410	-0.014	0.002	2.20E-10	-0.032	0.038	0.406	40.282	8.72E-05	NA
rs2307111	5	75003678	T	C	0.396	0.025	0.002	4.41E-29	0.004	0.039	0.923	125.285	2.71E-04	NA
rs2396625	7	113028634	T	A	0.401	0.014	0.002	1.99E-10	-0.038	0.038	0.326	40.473	8.76E-05	NA
rs2436726	6	40366427	C	T	0.405	-0.016	0.002	2.24E-12	0.006	0.038	0.879	49.262	1.07E-04	NA
rs2453763	5	92376460	T	A	0.347	-0.012	0.002	3.72E-08	-0.013	0.043	0.757	30.290	6.56E-05	NA
rs2470945	7	104585760	C	A	0.408	-0.012	0.002	2.10E-08	0.008	0.039	0.828	31.396	6.80E-05	NA
rs2516740	16	2097110	A	C	0.226	0.020	0.003	7.50E-14	0.060	0.050	0.231	55.934	1.21E-04	NA
rs2526754	3	50125996	G	A	0.494	0.022	0.002	5.98E-22	0.019	0.038	0.609	92.735	2.01E-04	Educational attainment; Intelligence
rs2531995	16	4013467	C	T	0.387	-0.019	0.002	2.80E-17	-0.005	0.039	0.909	71.477	1.55E-04	NA
rs2596933	3	9365746	G	A	0.267	0.014	0.002	1.26E-08	0.026	0.047	0.579	32.396	7.01E-05	NA
rs261336	15	58742418	G	A	0.161	-0.020	0.003	2.07E-14	0.078	0.056	0.161	58.461	1.27E-04	NA
rs2787693	1	49915848	C	A	0.302	0.017	0.002	1.94E-11	0.034	0.040	0.395	45.027	9.75E-05	NA
rs2814944	6	34552797	G	A	0.137	-0.034	0.003	1.03E-26	0.027	0.052	0.609	114.463	2.48E-04	NA
rs2836753	21	40291187	T	C	0.385	-0.013	0.002	7.29E-10	-0.004	0.039	0.928	37.941	8.21E-05	NA
rs2861688	2	67838474	T	C	0.273	0.016	0.002	8.97E-11	0.064	0.043	0.137	42.033	9.10E-05	NA
rs2925979	16	81534790	T	C	0.288	0.014	0.002	1.10E-10	0.048	0.041	0.238	41.628	9.01E-05	NA
rs2968856	7	150614934	T	C	0.261	0.016	0.002	1.80E-11	0.027	0.048	0.573	45.179	9.78E-05	NA
rs2990997	1	190119703	T	A	0.353	-0.012	0.002	3.24E-08	0.009	0.039	0.822	30.558	6.62E-05	NA
rs320	8	19819077	T	G	0.287	0.031	0.002	2.30E-43	-0.008	0.043	0.849	190.642	4.13E-04	NA
rs323781	5	86752616	A	C	0.034	-0.032	0.006	3.82E-08	-0.149	0.104	0.153	30.237	6.55E-05	NA
rs349088	11	84814393	C	A	0.480	0.013	0.002	3.23E-09	0.026	0.038	0.501	35.040	7.59E-05	NA
rs3749262	3	131624097	G	A	0.263	-0.019	0.002	1.12E-14	-0.078	0.042	0.065	59.681	1.29E-04	NA
rs3808436	8	116560967	A	G	0.432	0.016	0.002	1.58E-13	0.021	0.038	0.588	54.468	1.18E-04	NA
rs3810291	19	47569003	G	A	0.337	-0.019	0.002	2.74E-17	0.048	0.043	0.264	71.521	1.55E-04	NA
rs3814424	5	87968953	C	T	0.172	-0.025	0.003	2.55E-16	-0.061	0.051	0.225	67.124	1.45E-04	Intelligence
rs3864004	3	41240177	G	A	0.432	-0.014	0.002	2.30E-09	0.059	0.038	0.118	35.700	7.73E-05	NA
rs3902840	9	129419025	G	A	0.092	-0.021	0.004	1.72E-08	-0.030	0.069	0.671	31.786	6.88E-05	NA
rs40067	5	107439012	G	A	0.180	0.018	0.003	4.05E-11	0.050	0.053	0.342	43.590	9.44E-05	NA
rs4074448	15	63888495	G	A	0.434	0.012	0.002	2.37E-08	0.020	0.042	0.623	31.161	6.75E-05	NA
rs4283409	2	228977142	A	G	0.387	-0.012	0.002	7.64E-09	0.023	0.042	0.587	33.365	7.22E-05	NA
rs429343	2	147903382	A	G	0.437	0.012	0.002	1.26E-08	0.050	0.038	0.186	32.394	7.01E-05	NA
rs4377779	6	12117344	T	C	0.334	0.013	0.002	5.27E-09	-0.055	0.040	0.164	34.086	7.38E-05	NA
rs4410790	7	17284577	T	C	0.385	-0.012	0.002	5.72E-09	0.008	0.039	0.844	33.927	7.34E-05	NA
rs442177	4	88030261	G	T	0.421	-0.011	0.002	3.89E-08	-0.069	0.039	0.077	30.204	6.54E-05	NA
rs4477562	13	54104968	C	T	0.110	-0.020	0.003	9.80E-09	-0.021	0.063	0.744	32.880	7.12E-05	NA
rs4482463	2	205375909	C	A	0.081	0.029	0.004	4.75E-12	-0.116	0.076	0.127	47.787	1.03E-04	NA
rs4660208	1	39722214	A	G	0.195	-0.023	0.003	2.84E-18	-0.070	0.045	0.121	75.995	1.64E-04	NA
rs4671328	2	58935282	T	G	0.430	0.018	0.002	9.13E-17	0.013	0.038	0.734	69.149	1.50E-04	NA
rs4693325	4	94408529	A	C	0.199	-0.016	0.003	1.74E-09	-0.029	0.053	0.591	36.241	7.85E-05	NA
rs4740619	9	15634326	T	C	0.481	0.014	0.002	6.33E-11	0.038	0.038	0.323	42.715	9.25E-05	NA
rs4755720	11	43628749	C	T	0.400	0.016	0.002	4.50E-14	0.041	0.042	0.324	56.935	1.23E-04	NA

rs4777542	15	73082366	C	T	0.300	0.014	0.002	3.14E-09	-0.013	0.041	0.743	35.098	7.60E-05	NA
rs4784745	16	57014875	A	G	0.329	-0.019	0.002	4.00E-21	0.026	0.044	0.545	88.973	1.93E-04	NA
rs479315	11	66064991	G	C	0.170	-0.016	0.003	2.08E-08	0.025	0.048	0.609	31.420	6.80E-05	NA
rs4805758	19	32866424	G	A	0.162	0.020	0.003	5.55E-13	-0.029	0.051	0.574	51.999	1.13E-04	Fed-up feelings
rs4810479	20	44545048	C	T	0.288	0.013	0.002	6.17E-10	0.016	0.043	0.712	38.267	8.28E-05	NA
rs4889782	17	78640510	C	T	0.415	0.013	0.002	1.61E-09	-0.005	0.039	0.895	36.391	7.88E-05	NA
rs4890439	18	40732586	G	A	0.375	0.012	0.002	4.60E-08	-0.031	0.039	0.432	29.879	6.47E-05	NA
rs4929923	11	8639200	T	C	0.377	-0.015	0.002	4.28E-11	0.012	0.042	0.770	43.484	9.41E-05	NA
rs4947406	7	50661735	T	G	0.350	0.016	0.002	9.35E-12	0.022	0.044	0.621	46.461	1.01E-04	NA
rs4971632	2	50240091	T	C	0.166	0.017	0.003	1.26E-09	-0.028	0.051	0.589	36.870	7.98E-05	NA
rs512005	1	33377679	T	C	0.236	0.014	0.003	4.27E-08	0.023	0.047	0.624	30.024	6.50E-05	NA
rs545608	1	177899121	G	C	0.186	-0.038	0.003	1.86E-37	-0.005	0.049	0.916	163.594	3.54E-04	NA
rs563296	10	99772404	G	A	0.441	-0.013	0.002	1.23E-08	-0.055	0.038	0.148	32.438	7.02E-05	NA
rs6115015	20	25027692	G	A	0.333	-0.013	0.002	6.30E-09	-0.022	0.043	0.617	33.741	7.30E-05	NA
rs619054	11	116660813	G	A	0.232	0.015	0.002	2.70E-11	0.019	0.048	0.689	44.384	9.61E-05	NA
rs645040	3	135926622	G	T	0.229	-0.023	0.002	1.31E-20	-0.023	0.046	0.622	86.621	1.87E-04	NA
rs6463489	7	5542513	T	C	0.069	0.024	0.004	1.03E-08	0.101	0.062	0.104	32.787	7.10E-05	NA
rs649458	1	23712528	T	A	0.137	0.018	0.003	5.66E-09	0.024	0.055	0.661	33.949	7.35E-05	NA
rs6499240	16	69686912	A	G	0.422	-0.020	0.002	1.52E-18	-0.068	0.042	0.105	77.230	1.67E-04	NA
rs6499861	16	56991495	C	G	0.162	-0.018	0.003	8.61E-12	-0.031	0.057	0.591	46.623	1.01E-04	NA
rs6545714	2	59307725	G	A	0.382	0.019	0.002	3.15E-18	-0.023	0.039	0.556	75.796	1.64E-04	NA
rs6548834	3	82704753	G	A	0.378	-0.013	0.002	1.08E-08	-0.002	0.039	0.953	32.694	7.08E-05	NA
rs6550597	3	18738940	G	A	0.298	-0.013	0.002	4.61E-09	-0.018	0.042	0.671	34.346	7.43E-05	NA
rs6575340	14	94023972	G	A	0.377	-0.014	0.002	8.82E-10	0.010	0.039	0.805	37.571	8.13E-05	NA
rs6577584	1	6715390	T	G	0.372	-0.014	0.002	1.65E-10	0.052	0.040	0.193	40.843	8.84E-05	NA
rs6602151	10	16764483	T	C	0.294	-0.015	0.002	5.89E-10	-0.017	0.045	0.701	38.356	8.30E-05	NA
rs6603004	15	84568504	A	G	0.466	-0.013	0.002	1.81E-09	-0.041	0.038	0.280	36.163	7.83E-05	NA
rs6673977	1	7736097	A	G	0.354	-0.012	0.002	1.63E-08	0.014	0.039	0.720	31.892	6.90E-05	NA
rs6711375	2	161090873	G	A	0.320	-0.014	0.002	1.11E-09	0.001	0.044	0.974	37.115	8.03E-05	NA
rs6754311	2	136707982	T	C	0.489	0.013	0.002	3.95E-10	0.069	0.046	0.131	39.137	8.47E-05	NA
rs676387	17	40706273	C	A	0.266	-0.013	0.002	3.42E-08	0.018	0.043	0.682	30.452	6.59E-05	NA
rs6868198	5	122750714	G	A	0.432	0.013	0.002	2.10E-08	-0.016	0.041	0.695	31.399	6.80E-05	NA
rs6870983	5	87697533	C	T	0.236	0.020	0.003	1.66E-15	0.005	0.046	0.907	63.434	1.37E-04	NA
rs687672	11	65649984	T	C	0.189	0.021	0.003	5.92E-14	0.091	0.048	0.054	56.399	1.22E-04	NA
rs6907923	6	51802669	G	A	0.341	-0.015	0.002	1.71E-10	0.020	0.040	0.624	40.774	8.83E-05	NA
rs7016529	8	19806631	T	C	0.011	-0.102	0.009	1.94E-27	0.163	0.167	0.332	117.778	2.55E-04	NA
rs703965	10	80955067	C	T	0.456	-0.012	0.002	8.25E-09	0.041	0.038	0.280	33.214	7.19E-05	NA
rs7075195	10	65050659	A	G	0.435	0.017	0.002	5.31E-16	0.031	0.041	0.446	65.676	1.42E-04	Educational attainment
rs7084454	10	21821274	G	A	0.338	-0.016	0.002	9.04E-13	0.011	0.040	0.781	51.042	1.10E-04	NA
rs7132908	12	50263148	G	A	0.357	-0.019	0.002	9.07E-15	0.020	0.042	0.634	60.088	1.30E-04	NA
rs7144011	14	79940383	G	T	0.207	-0.023	0.003	1.71E-17	0.070	0.047	0.135	72.451	1.57E-04	NA
rs7161874	15	53163740	C	G	0.214	-0.015	0.003	4.11E-09	0.058	0.043	0.174	34.573	7.48E-05	NA
rs7186893	16	24806420	G	T	0.267	0.020	0.002	3.04E-16	0.015	0.042	0.729	66.779	1.45E-04	Mood swings
rs7198357	16	67884619	C	T	0.178	0.017	0.003	4.09E-11	-0.069	0.050	0.168	43.573	9.43E-05	NA
rs7206608	16	82872628	C	G	0.313	-0.013	0.002	1.26E-08	0.023	0.041	0.580	32.386	7.01E-05	NA
rs7212293	17	65847325	A	G	0.216	-0.022	0.003	4.05E-17	0.027	0.046	0.562	70.753	1.53E-04	NA
rs7241918	18	47160953	G	T	0.163	0.018	0.003	7.52E-13	0.009	0.051	0.860	51.403	1.11E-04	NA
rs7323827	13	94116675	A	G	0.304	0.013	0.002	4.59E-09	0.022	0.041	0.593	34.358	7.44E-05	NA
rs741959	1	47676233	A	G	0.402	0.014	0.002	6.19E-11	0.042	0.039	0.279	42.760	9.26E-05	NA
rs7515635	1	42408070	T	C	0.471	0.012	0.002	4.68E-09	-0.050	0.038	0.186	34.317	7.43E-05	NA
rs7537581	1	2725475	C	A	0.476	-0.012	0.002	4.55E-08	0.008	0.042	0.850	29.899	6.47E-05	NA

rs7549987	1	80789011	C	T	0.299	-0.014	0.002	4.02E-09	0.001	0.041	0.977	34.614	7.49E-05	NA
rs7581217	2	25524944	T	C	0.370	0.013	0.002	5.93E-09	-0.026	0.044	0.551	33.857	7.33E-05	NA
rs7601028	2	642499	C	G	0.174	-0.046	0.003	1.02E-52	-0.034	0.052	0.513	233.512	5.05E-04	NA
rs7638388	3	47914974	C	T	0.088	-0.024	0.004	1.01E-10	-0.038	0.073	0.599	41.794	9.05E-05	NA
rs7642876	3	15883094	A	G	0.404	0.012	0.002	1.35E-08	0.045	0.039	0.245	32.265	6.98E-05	NA
rs7668263	4	143781835	T	C	0.382	-0.012	0.002	4.21E-08	-0.031	0.039	0.427	30.049	6.50E-05	NA
rs779655	5	170495158	G	C	0.272	-0.014	0.002	1.21E-08	-0.037	0.043	0.389	32.464	7.03E-05	NA
rs7824519	8	14222796	T	G	0.324	-0.013	0.002	1.51E-08	-0.051	0.040	0.206	32.035	6.93E-05	NA
rs7928842	11	47566352	T	C	0.422	-0.020	0.002	2.30E-20	0.011	0.039	0.780	85.511	1.85E-04	NA
rs7947951	11	13356030	A	G	0.297	-0.014	0.002	1.59E-10	0.029	0.041	0.480	40.911	8.86E-05	NA
rs7962636	12	116450006	T	C	0.355	0.013	0.002	1.32E-08	0.007	0.041	0.863	32.308	6.99E-05	NA
rs7982447	13	54453811	T	C	0.200	-0.019	0.003	6.75E-13	0.011	0.047	0.812	51.616	1.12E-04	NA
rs8015400	14	25930988	C	A	0.348	-0.018	0.002	8.13E-15	0.052	0.041	0.202	60.303	1.31E-04	NA
rs8055138	16	28891465	C	T	0.329	-0.030	0.002	1.86E-35	0.008	0.038	0.845	154.432	3.34E-04	Educational attainment; Intelligence
rs8078079	17	34959346	T	C	0.395	0.015	0.002	2.89E-11	-0.027	0.039	0.481	44.247	9.58E-05	NA
rs808820	12	89744826	A	G	0.116	-0.022	0.004	8.37E-10	-0.057	0.057	0.316	37.671	8.15E-05	NA
rs818524	1	85201228	T	C	0.289	-0.013	0.002	2.84E-08	0.017	0.045	0.703	30.812	6.67E-05	NA
rs8192675	3	170724883	T	C	0.289	-0.013	0.002	4.30E-08	-0.057	0.041	0.164	30.007	6.50E-05	NA
rs891387	18	21103909	T	C	0.473	0.021	0.002	3.25E-21	0.011	0.038	0.774	89.383	1.93E-04	Educational attainment
rs906673	3	12898941	C	T	0.389	0.013	0.002	3.67E-09	0.010	0.039	0.792	34.789	7.53E-05	NA
rs9277972	6	33276770	A	T	0.168	-0.016	0.003	1.55E-08	0.075	0.052	0.154	31.995	6.93E-05	NA
rs938875	15	67952922	A	G	0.408	-0.019	0.002	3.72E-18	-0.065	0.039	0.096	75.463	1.63E-04	NA
rs9478499	6	154345289	T	A	0.147	-0.019	0.003	1.35E-10	0.059	0.055	0.284	41.238	8.93E-05	NA
rs9556979	13	99241507	T	G	0.324	-0.013	0.002	4.53E-09	-0.076	0.045	0.088	34.382	7.44E-05	NA
rs9563569	13	58571207	G	A	0.150	0.018	0.003	2.09E-08	0.048	0.051	0.345	31.408	6.80E-05	NA
rs9630985	2	181607676	A	C	0.349	-0.014	0.002	9.30E-11	-0.032	0.041	0.439	41.963	9.08E-05	NA
rs9686661	5	55861786	C	T	0.186	-0.023	0.003	4.65E-18	-0.054	0.050	0.284	75.025	1.62E-04	NA
rs9816226	3	185834499	A	T	0.188	-0.016	0.003	8.56E-09	-0.071	0.050	0.156	33.144	7.17E-05	NA
rs9872156	3	176337348	G	A	0.260	-0.013	0.002	4.04E-08	0.004	0.043	0.932	30.129	6.52E-05	NA
rs987237	6	50803050	A	G	0.181	-0.030	0.003	7.81E-26	0.054	0.049	0.270	110.449	2.39E-04	NA
rs9947450	18	58052665	C	T	0.030	0.062	0.007	1.22E-21	0.178	0.112	0.114	91.321	1.98E-04	NA
rs998584	6	43757896	C	A	0.496	-0.013	0.002	9.99E-11	0.044	0.042	0.287	41.823	9.05E-05	NA
rs9987289	8	9183358	A	G	0.075	0.022	0.004	1.84E-09	-0.046	0.067	0.496	36.136	7.82E-05	NA

Genomic positions reported in the GWAS of Mets refer to human reference assembly (GRCh37/hg19).

Abbreviations: MetS, Metabolic syndrome; PTSD, Ppost-traumatic stress disorder; SNPs, Single nucleotide polymorphisms; CHR, Chromosome; EA, Effect allele; OA, Other allele; EAF, Effect allele frequency; SE, Standard error; PVE, Proportion of phenotypic variance explained.

SNPs that are unavailable in the outcome GWAS:rs705986,rs1412239, among them, rs13288841and rs705986 were substitute by proxy SNPs: rs1412239 and rs705986, respectively.

5 SNPs were detected by PRESSO outlier test, and 12 SNPs were detected by MR-Radial outlier test..

Supplementary Table11. Genetic instruments for the causal relationship of Mets and TS.

SNP	CHR	Position	EA	OA	EAF	Beta for exposure	SE for exposure	P value for exposure	Beta for outcome	SE for outcome	P value for outcome	F statistics	PVE	Association with potential confounding factors
rs10116353	9	111950088	G	T	0.307	0.013	0.002	9.39E-09	0.014	0.028	0.617	32.964	7.14E-05	NA
rs1013402	11	27712381	A	G	0.290	-0.025	0.002	1.34E-23	0.004	0.029	0.881	100.255	2.17E-04	NA
rs102275	11	61557803	T	C	0.363	-0.013	0.002	6.28E-11	0.023	0.027	0.410	42.731	9.25E-05	NA
rs1032524	1	201790826	T	C	0.484	-0.015	0.002	6.01E-12	0.004	0.026	0.875	47.325	1.02E-04	NA
rs1042034	2	21225281	C	T	0.218	-0.020	0.002	7.07E-21	-0.051	0.032	0.110	87.848	1.90E-04	NA
rs10734924	12	123018894	T	G	0.259	-0.015	0.003	2.19E-09	0.032	0.030	0.288	35.801	7.75E-05	NA
rs10768994	11	43936945	T	C	0.430	0.012	0.002	4.52E-08	0.008	0.026	0.771	29.912	6.48E-05	NA
rs10779835	1	230299949	T	C	0.391	0.015	0.002	2.45E-14	-0.013	0.027	0.630	58.131	1.26E-04	NA
rs10783779	12	56491880	T	G	0.403	0.013	0.002	3.84E-09	0.038	0.027	0.153	34.704	7.51E-05	NA
rs10808546	8	126495818	C	T	0.431	0.016	0.002	4.56E-19	-0.020	0.027	0.450	79.612	1.72E-04	NA
rs10838620	11	46818098	G	A	0.214	-0.015	0.002	1.25E-09	-0.032	0.032	0.308	36.883	7.98E-05	NA
rs10887579	10	88097210	A	C	0.354	-0.013	0.002	4.09E-09	0.041	0.028	0.134	34.579	7.49E-05	NA
rs10930502	2	172890588	A	G	0.304	0.013	0.002	1.18E-08	-0.035	0.028	0.220	32.519	7.04E-05	NA
rs10938397	4	45182527	A	G	0.420	-0.023	0.002	8.14E-26	-0.024	0.026	0.354	110.367	2.39E-04	NA
rs11072938	15	81021661	C	T	0.241	0.016	0.002	4.30E-11	-0.026	0.029	0.369	43.470	9.41E-05	NA
rs11079849	17	47090785	C	T	0.290	0.015	0.002	6.90E-11	0.048	0.028	0.092	42.547	9.21E-05	NA
rs11150604	16	31037020	A	T	0.389	0.024	0.002	3.91E-28	-0.016	0.026	0.550	120.954	2.62E-04	NA
rs11165643	1	96924097	C	T	0.417	-0.013	0.002	6.90E-10	-0.009	0.026	0.728	38.049	8.24E-05	NA
rs11187845	10	96060198	C	A	0.096	0.020	0.004	8.08E-09	0.037	0.042	0.374	33.256	7.20E-05	NA
rs1138429	16	56942921	A	T	0.101	-0.028	0.003	1.42E-18	0.004	0.044	0.920	77.365	1.67E-04	NA
rs1159974	6	126090277	T	C	0.499	-0.013	0.002	3.30E-10	-0.021	0.026	0.415	39.489	8.55E-05	NA
rs11611246	12	939480	G	T	0.183	-0.016	0.003	1.14E-08	0.020	0.033	0.535	32.583	7.05E-05	NA
rs11631408	15	51746236	A	G	0.463	0.014	0.002	2.44E-11	0.039	0.026	0.128	44.582	9.65E-05	NA
rs11636565	15	36391846	C	A	0.488	-0.014	0.002	6.84E-12	0.020	0.026	0.443	47.072	1.02E-04	NA
rs11660335	18	22154235	T	C	0.161	0.018	0.003	1.07E-09	0.012	0.035	0.734	37.189	8.05E-05	NA
rs11676272	2	25141538	A	G	0.464	-0.020	0.002	1.05E-17	0.015	0.026	0.572	73.421	1.59E-04	NA
rs11747810	5	153100273	T	C	0.495	0.013	0.002	1.83E-09	-0.013	0.026	0.628	36.148	7.83E-05	NA
rs11766944	7	1888051	G	A	0.214	0.015	0.003	4.17E-08	0.012	0.031	0.692	30.068	6.51E-05	NA
rs11826999	11	116553778	G	A	0.223	-0.023	0.002	6.07E-26	0.012	0.031	0.705	110.950	2.40E-04	NA
rs11866815	16	387867	C	T	0.242	0.016	0.002	2.32E-10	-0.015	0.030	0.617	40.181	8.70E-05	NA
rs11880870	19	18830704	A	G	0.499	0.013	0.002	2.98E-09	0.015	0.026	0.550	35.198	7.62E-05	NA
rs11981190	7	71659538	G	A	0.341	0.013	0.002	5.84E-09	0.023	0.027	0.403	33.887	7.34E-05	NA
rs1200349	15	41821752	T	C	0.482	-0.013	0.002	1.36E-09	-0.030	0.026	0.246	36.724	7.95E-05	NA
rs12033257	1	112318484	A	G	0.382	0.014	0.002	8.97E-10	0.002	0.028	0.946	37.538	8.13E-05	NA
rs12201987	6	143182396	C	T	0.205	0.016	0.003	1.87E-09	0.018	0.033	0.590	36.103	7.82E-05	NA
rs12513212	4	96149346	A	C	0.387	-0.014	0.002	2.85E-10	0.006	0.027	0.830	39.778	8.61E-05	NA
rs12520822	5	105699867	C	T	0.408	0.012	0.002	1.84E-08	0.049	0.028	0.082	31.658	6.85E-05	NA
rs12596491	16	88928417	C	T	0.340	0.012	0.002	4.58E-08	-0.013	0.028	0.628	29.888	6.47E-05	NA
rs12608504	19	18389135	A	G	0.359	0.016	0.002	2.27E-13	0.010	0.027	0.705	53.757	1.16E-04	NA
rs12624640	20	32952125	G	A	0.430	-0.012	0.002	7.17E-09	-0.047	0.026	0.071	33.489	7.25E-05	NA
rs12967135	18	57849023	G	A	0.240	-0.046	0.003	3.09E-66	-0.039	0.030	0.197	295.539	6.39E-04	Worrier or anxious feelings
rs12981256	19	1865901	G	A	0.475	-0.016	0.002	7.75E-14	-0.016	0.027	0.554	55.867	1.21E-04	NA
rs13024601	2	100902032	C	T	0.346	0.014	0.002	1.39E-09	0.004	0.027	0.877	36.680	7.94E-05	NA
rs13029936	2	208465544	C	T	0.176	0.017	0.003	1.31E-09	0.005	0.033	0.885	36.795	7.96E-05	NA
rs13107325	4	103188709	C	T	0.080	-0.023	0.004	1.94E-09	0.006	0.049	0.904	36.033	7.80E-05	Intelligence
rs13206549	6	162978032	T	G	0.134	0.019	0.003	1.54E-09	-0.049	0.040	0.214	36.486	7.90E-05	NA
rs13244268	7	72911843	T	C	0.114	0.024	0.003	9.17E-18	0.017	0.041	0.673	73.684	1.59E-04	NA
rs13250456	8	72408720	C	T	0.055	-0.025	0.005	4.67E-08	0.014	0.062	0.821	29.850	6.46E-05	NA
rs13275593	8	68257300	G	A	0.285	-0.015	0.002	2.14E-10	-0.005	0.029	0.863	40.332	8.73E-05	NA

rs1330304	9	16714629	C	G	0.267	0.013	0.002	2.32E-08	0.027	0.029	0.363	31.202	6.75E-05	NA
rs13322435	3	156795468	A	G	0.407	0.020	0.002	6.60E-20	-0.014	0.029	0.635	83.432	1.81E-04	NA
rs13416667	2	56608267	C	A	0.274	-0.015	0.002	2.32E-10	0.028	0.030	0.348	40.179	8.70E-05	NA
rs13435860	4	136975545	A	T	0.368	-0.013	0.002	1.94E-09	-0.036	0.029	0.217	36.037	7.80E-05	NA
rs1353580	15	47738732	T	A	0.374	-0.012	0.002	1.70E-08	0.019	0.027	0.478	31.810	6.89E-05	NA
rs1362910	8	30856464	A	G	0.386	0.014	0.002	9.70E-11	-0.038	0.026	0.151	41.882	9.07E-05	NA
rs1363695	5	130378027	C	T	0.228	0.015	0.003	6.49E-09	0.010	0.032	0.761	33.681	7.29E-05	NA
rs1421085	16	53800954	T	C	0.432	-0.062	0.002	5.07E-152	-0.007	0.026	0.793	689.747	1.49E-03	NA
rs1448613	3	85875265	G	A	0.386	0.014	0.002	2.40E-10	-0.041	0.027	0.132	40.110	8.68E-05	NA
rs1452075	3	62481063	C	T	0.284	-0.014	0.002	9.96E-09	0.027	0.029	0.342	32.850	7.11E-05	NA
rs1456116	3	123084379	G	A	0.417	-0.013	0.002	1.99E-09	-0.043	0.026	0.101	35.987	7.79E-05	NA
rs1458156	12	41887940	C	T	0.449	-0.013	0.002	4.45E-09	-0.009	0.026	0.732	34.416	7.45E-05	NA
rs1460940	1	72814617	G	A	0.182	-0.020	0.003	2.89E-13	-0.028	0.035	0.420	53.284	1.15E-04	NA
rs1507153	6	79484386	C	A	0.364	-0.013	0.002	3.86E-09	0.011	0.026	0.669	34.694	7.51E-05	NA
rs1513475	3	35724256	T	C	0.343	0.015	0.002	8.46E-11	-0.030	0.027	0.261	42.149	9.12E-05	NA
rs1534696	7	26397239	C	A	0.439	0.014	0.002	1.24E-11	0.002	0.027	0.952	45.905	9.94E-05	NA
rs1580099	3	94003603	C	A	0.406	0.014	0.002	4.35E-09	0.029	0.026	0.260	34.459	7.46E-05	NA
rs17024393	1	110154688	T	C	0.033	-0.048	0.006	1.96E-15	0.041	0.081	0.609	63.106	1.37E-04	NA
rs17115145	14	30122409	C	T	0.406	-0.012	0.002	1.29E-08	-0.003	0.027	0.913	32.350	7.00E-05	NA
rs17162333	1	27236757	C	G	0.132	-0.020	0.003	6.24E-11	0.025	0.037	0.493	42.744	9.25E-05	NA
rs17207196	7	75101065	C	T	0.444	0.018	0.002	2.68E-16	0.018	0.027	0.513	67.024	1.45E-04	NA
rs1730858	1	107619244	T	C	0.328	0.015	0.002	1.04E-10	-0.003	0.028	0.919	41.736	9.03E-05	NA
rs17391694	1	78623626	C	T	0.096	-0.026	0.004	2.39E-12	-0.057	0.049	0.244	49.132	1.06E-04	NA
rs17513613	19	30286822	T	C	0.289	-0.017	0.002	1.94E-13	-0.047	0.028	0.094	54.063	1.17E-04	NA
rs17618704	17	46221010	T	G	0.068	0.025	0.004	1.52E-09	0.030	0.051	0.559	36.510	7.90E-05	NA
rs17639546	3	61251635	G	A	0.148	0.018	0.003	3.65E-09	0.009	0.038	0.814	34.803	7.53E-05	NA
rs17694506	2	171632225	C	T	0.388	0.014	0.002	3.33E-11	-0.037	0.027	0.159	43.971	9.52E-05	NA
rs17724992	19	18454825	A	G	0.245	0.014	0.002	8.13E-09	0.032	0.030	0.278	33.243	7.20E-05	NA
rs17783751	16	89513984	C	A	0.176	-0.015	0.003	4.79E-08	-0.035	0.032	0.270	29.800	6.45E-05	NA
rs17806379	20	51107290	C	T	0.185	0.020	0.003	3.64E-13	-0.035	0.034	0.293	52.829	1.14E-04	NA
rs17821316	15	58703069	A	C	0.488	-0.012	0.002	9.97E-10	0.053	0.034	0.113	37.331	8.08E-05	NA
rs1784460	11	118938371	T	A	0.360	-0.015	0.002	8.15E-12	-0.005	0.026	0.854	46.729	1.01E-04	NA
rs1876359	16	4930100	C	T	0.377	-0.013	0.002	5.40E-09	-0.008	0.027	0.771	34.038	7.37E-05	NA
rs1886558	13	111982615	A	G	0.389	0.012	0.002	4.14E-08	-0.045	0.027	0.093	30.082	6.51E-05	NA
rs1899689	7	121964349	C	T	0.380	-0.012	0.002	4.27E-08	-0.005	0.026	0.846	30.021	6.50E-05	NA
rs1914888	17	21267541	A	G	0.462	0.013	0.002	1.33E-09	-0.019	0.027	0.477	36.762	7.96E-05	NA
rs192812	3	161491831	C	T	0.454	-0.012	0.002	4.87E-09	-0.042	0.026	0.109	34.241	7.41E-05	NA
rs1945941	11	132620118	G	A	0.376	0.012	0.002	3.63E-08	0.009	0.026	0.724	30.337	6.57E-05	NA
rs2002023	10	76848524	C	T	0.441	-0.012	0.002	8.92E-09	-0.013	0.026	0.617	33.064	7.16E-05	NA
rs2015407	14	103274547	A	G	0.345	0.014	0.002	4.68E-10	-0.022	0.027	0.411	38.808	8.40E-05	NA
rs2035831	3	173683791	G	C	0.321	0.013	0.002	1.48E-08	0.003	0.028	0.923	32.085	6.95E-05	NA
rs2060604	8	76650334	T	C	0.414	0.015	0.002	2.80E-12	0.001	0.026	0.979	48.822	1.06E-04	NA
rs2065418	11	30422068	T	G	0.354	0.016	0.002	1.39E-12	-0.033	0.027	0.228	50.196	1.09E-04	NA
rs2066295	6	26168903	A	G	0.219	0.023	0.003	5.73E-18	-0.004	0.030	0.887	74.611	1.61E-04	NA
rs2074881	19	1970021	C	T	0.179	0.015	0.003	2.25E-08	-0.007	0.035	0.840	31.270	6.77E-05	NA
rs2075226	2	36805676	A	G	0.396	-0.013	0.002	2.48E-08	-0.039	0.027	0.142	31.073	6.73E-05	NA
rs208015	17	46252346	T	C	0.088	0.022	0.004	1.07E-08	0.070	0.048	0.146	32.711	7.08E-05	NA
rs2143253	20	41987392	G	A	0.137	0.019	0.003	1.29E-09	-0.039	0.037	0.289	36.823	7.97E-05	NA
rs2180454	14	29690513	T	C	0.241	-0.015	0.003	1.90E-09	-0.007	0.030	0.823	36.071	7.81E-05	NA
rs2237717	7	116405387	T	C	0.443	-0.011	0.002	2.63E-08	-0.035	0.026	0.177	30.960	6.70E-05	NA
rs2242449	17	7095507	C	T	0.417	-0.013	0.002	2.85E-09	0.041	0.027	0.125	35.287	7.64E-05	NA

rs227199	1	210265384	G	C	0.316	0.013	0.002	5.06E-09	0.006	0.027	0.832	34.168	7.40E-05	NA
rs2275543	9	107651174	T	C	0.099	-0.019	0.003	6.58E-09	0.027	0.044	0.528	33.655	7.29E-05	NA
rs2298969	4	3186244	A	G	0.490	0.012	0.002	2.79E-08	-0.040	0.026	0.123	30.851	6.68E-05	NA
rs2299383	7	103418846	C	T	0.410	-0.014	0.002	2.20E-10	0.011	0.026	0.683	40.282	8.72E-05	NA
rs2307111	5	75003678	T	C	0.396	0.025	0.002	4.41E-29	0.015	0.026	0.574	125.285	2.71E-04	NA
rs2396625	7	113028634	T	A	0.401	0.014	0.002	1.99E-10	0.012	0.026	0.652	40.473	8.76E-05	NA
rs2436726	6	40366427	C	T	0.405	-0.016	0.002	2.24E-12	-0.009	0.026	0.727	49.262	1.07E-04	NA
rs2453763	5	92376460	T	A	0.347	-0.012	0.002	3.72E-08	-0.049	0.028	0.079	30.290	6.56E-05	NA
rs2470945	7	104585760	C	A	0.408	-0.012	0.002	2.10E-08	0.046	0.027	0.088	31.396	6.80E-05	NA
rs2526754	3	50125996	G	A	0.494	0.022	0.002	5.98E-22	-0.004	0.026	0.882	92.735	2.01E-04	Educational attainment; Intelligence
rs2531686	10	118651011	C	G	0.276	0.015	0.002	2.22E-10	-0.016	0.029	0.580	40.261	8.72E-05	NA
rs2531995	16	4013467	C	T	0.387	-0.019	0.002	2.80E-17	0.019	0.026	0.463	71.477	1.55E-04	NA
rs2596933	3	9365746	G	A	0.267	0.014	0.002	1.26E-08	-0.043	0.030	0.146	32.396	7.01E-05	NA
rs261336	15	58742418	G	A	0.161	-0.020	0.003	2.07E-14	-0.002	0.036	0.947	58.461	1.27E-04	NA
rs2781668	6	131897278	C	T	0.170	-0.015	0.003	3.64E-08	-0.065	0.037	0.073	30.331	6.57E-05	NA
rs2787693	1	49915848	C	A	0.302	0.017	0.002	1.94E-11	-0.004	0.028	0.877	45.027	9.75E-05	NA
rs2814944	6	34552797	G	A	0.137	-0.034	0.003	1.03E-26	0.009	0.036	0.809	114.463	2.48E-04	NA
rs2820232	6	35003603	G	T	0.459	-0.012	0.002	2.95E-08	0.027	0.026	0.308	30.741	6.65E-05	NA
rs2836753	21	40291187	T	C	0.385	-0.013	0.002	7.29E-10	-0.010	0.027	0.709	37.941	8.21E-05	NA
rs2861688	2	67838474	T	C	0.273	0.016	0.002	8.97E-11	-0.018	0.029	0.532	42.033	9.10E-05	NA
rs2925979	16	81534790	T	C	0.288	0.014	0.002	1.10E-10	0.006	0.029	0.838	41.628	9.01E-05	NA
rs2968856	7	150614934	T	C	0.261	0.016	0.002	1.80E-11	0.003	0.030	0.916	45.179	9.78E-05	NA
rs2990997	1	190119703	T	A	0.353	-0.012	0.002	3.24E-08	0.047	0.027	0.081	30.558	6.62E-05	NA
rs320	8	19819077	T	G	0.287	0.031	0.002	2.30E-43	-0.032	0.029	0.271	190.642	4.13E-04	NA
rs323781	5	86752616	A	C	0.034	-0.032	0.006	3.82E-08	0.009	0.075	0.901	30.237	6.55E-05	NA
rs349088	11	84814393	C	A	0.480	0.013	0.002	3.23E-09	0.035	0.026	0.171	35.040	7.59E-05	NA
rs3749262	3	131624097	G	A	0.263	-0.019	0.002	1.12E-14	-0.048	0.029	0.099	59.681	1.29E-04	NA
rs3808436	8	116560967	A	G	0.432	0.016	0.002	1.58E-13	0.021	0.026	0.415	54.468	1.18E-04	NA
rs3814424	5	87968953	C	T	0.172	-0.025	0.003	2.55E-16	0.056	0.036	0.115	67.124	1.45E-04	Intelligence
rs3814883	16	29994922	C	T	0.460	-0.026	0.002	7.26E-31	-0.026	0.027	0.332	133.436	2.89E-04	Nervous feelings
rs3852012	3	44045981	G	A	0.287	0.013	0.002	1.57E-08	-0.017	0.028	0.539	31.963	6.92E-05	NA
rs3864004	3	41240177	G	A	0.432	-0.014	0.002	2.30E-09	0.035	0.026	0.182	35.700	7.73E-05	NA
rs3902840	9	129419025	G	A	0.092	-0.021	0.004	1.72E-08	-0.004	0.048	0.932	31.786	6.88E-05	NA
rs397092	21	46582564	A	G	0.456	-0.013	0.002	1.07E-09	0.007	0.026	0.794	37.196	8.05E-05	NA
rs4074448	15	63888495	G	A	0.434	0.012	0.002	2.37E-08	-0.052	0.027	0.052	31.161	6.75E-05	NA
rs429343	2	147903382	A	G	0.437	0.012	0.002	1.26E-08	-0.040	0.026	0.122	32.394	7.01E-05	NA
rs4377779	6	12117344	T	C	0.334	0.013	0.002	5.27E-09	-0.035	0.027	0.193	34.086	7.38E-05	NA
rs4410790	7	17284577	T	C	0.385	-0.012	0.002	5.72E-09	0.025	0.027	0.356	33.927	7.34E-05	NA
rs442177	4	88030261	G	T	0.421	-0.011	0.002	3.89E-08	0.010	0.027	0.692	30.204	6.54E-05	NA
rs4477562	13	54104968	C	T	0.110	-0.020	0.003	9.80E-09	0.019	0.038	0.624	32.880	7.12E-05	NA
rs4482463	2	205375909	C	A	0.081	0.029	0.004	4.75E-12	0.053	0.046	0.254	47.787	1.03E-04	NA
rs4660208	1	39722214	A	G	0.195	-0.023	0.003	2.84E-18	0.044	0.031	0.161	75.995	1.64E-04	NA
rs4665991	2	27766284	G	A	0.264	-0.013	0.002	2.16E-09	-0.005	0.029	0.870	35.821	7.75E-05	NA
rs4693325	4	94408529	A	C	0.199	-0.016	0.003	1.74E-09	0.005	0.033	0.877	36.241	7.85E-05	NA
rs4740619	9	15634326	T	C	0.481	0.014	0.002	6.33E-11	-0.031	0.026	0.225	42.715	9.25E-05	NA
rs4755720	11	43628749	C	T	0.400	0.016	0.002	4.50E-14	-0.043	0.027	0.107	56.935	1.23E-04	NA
rs4777542	15	73082366	C	T	0.300	0.014	0.002	3.14E-09	-0.053	0.028	0.056	35.098	7.60E-05	NA
rs4784745	16	57014875	A	G	0.329	-0.019	0.002	4.00E-21	-0.001	0.028	0.962	88.973	1.93E-04	NA
rs479315	11	66064991	G	C	0.170	-0.016	0.003	2.08E-08	0.039	0.033	0.233	31.420	6.80E-05	NA
rs4805758	19	32866424	G	A	0.162	0.020	0.003	5.55E-13	0.066	0.036	0.072	51.999	1.13E-04	Fed-up feelings
rs4810479	20	44545048	C	T	0.288	0.013	0.002	6.17E-10	0.044	0.029	0.131	38.267	8.28E-05	NA

rs4889782	17	78640510	C	T	0.415	0.013	0.002	1.61E-09	-0.017	0.027	0.513	36.391	7.88E-05	NA
rs4890439	18	40732586	G	A	0.375	0.012	0.002	4.60E-08	-0.012	0.027	0.645	29.879	6.47E-05	NA
rs4929923	11	8639200	T	C	0.377	-0.015	0.002	4.28E-11	-0.052	0.027	0.054	43.484	9.41E-05	NA
rs4947406	7	50661735	T	G	0.350	0.016	0.002	9.35E-12	0.024	0.029	0.394	46.461	1.01E-04	NA
rs4971632	2	50240091	T	C	0.166	0.017	0.003	1.26E-09	-0.031	0.036	0.395	36.870	7.98E-05	NA
rs512005	1	33377679	T	C	0.236	0.014	0.003	4.27E-08	0.011	0.031	0.717	30.024	6.50E-05	NA
rs545608	1	177899121	G	C	0.186	-0.038	0.003	1.86E-37	-0.062	0.034	0.071	163.594	3.54E-04	NA
rs563296	10	99772404	G	A	0.441	-0.013	0.002	1.23E-08	-0.002	0.026	0.937	32.438	7.02E-05	NA
rs6115015	20	25027692	G	A	0.333	-0.013	0.002	6.30E-09	-0.015	0.027	0.571	33.741	7.30E-05	NA
rs619054	11	116660813	G	A	0.232	0.015	0.002	2.70E-11	0.036	0.030	0.226	44.384	9.61E-05	NA
rs645040	3	135926622	G	T	0.229	-0.023	0.002	1.31E-20	0.052	0.030	0.086	86.621	1.87E-04	NA
rs6463489	7	5542513	T	C	0.069	0.024	0.004	1.03E-08	-0.051	0.044	0.240	32.787	7.10E-05	NA
rs649458	1	23712528	T	A	0.137	0.018	0.003	5.66E-09	-0.024	0.038	0.535	33.949	7.35E-05	NA
rs6499240	16	69686912	A	G	0.422	-0.020	0.002	1.52E-18	-0.019	0.026	0.479	77.230	1.67E-04	NA
rs6499861	16	56991495	C	G	0.162	-0.018	0.003	8.61E-12	0.018	0.037	0.624	46.623	1.01E-04	NA
rs6545714	2	59307725	G	A	0.382	0.019	0.002	3.15E-18	0.022	0.026	0.399	75.796	1.64E-04	NA
rs6548834	3	82704753	G	A	0.378	-0.013	0.002	1.08E-08	-0.010	0.027	0.718	32.694	7.08E-05	NA
rs6550597	3	18738940	G	A	0.298	-0.013	0.002	4.61E-09	0.003	0.028	0.906	34.346	7.43E-05	NA
rs6575340	14	94023972	G	A	0.377	-0.014	0.002	8.82E-10	-0.026	0.027	0.341	37.571	8.13E-05	NA
rs6577584	1	6715390	T	G	0.372	-0.014	0.002	1.65E-10	-0.024	0.027	0.380	40.843	8.84E-05	NA
rs6602151	10	16764483	T	C	0.294	-0.015	0.002	5.89E-10	0.008	0.029	0.779	38.356	8.30E-05	NA
rs6603004	15	84568504	A	G	0.466	-0.013	0.002	1.81E-09	-0.020	0.026	0.429	36.163	7.83E-05	NA
rs6673977	1	7736097	A	G	0.354	-0.012	0.002	1.63E-08	0.038	0.027	0.164	31.892	6.90E-05	NA
rs6711375	2	161090873	G	A	0.320	-0.014	0.002	1.11E-09	0.012	0.027	0.664	37.115	8.03E-05	NA
rs6754311	2	136707982	T	C	0.489	0.013	0.002	3.95E-10	0.041	0.029	0.164	39.137	8.47E-05	NA
rs676387	17	40706273	C	A	0.266	-0.013	0.002	3.42E-08	0.031	0.030	0.294	30.452	6.59E-05	NA
rs6868198	5	122750714	G	A	0.432	0.013	0.002	2.10E-08	-0.007	0.027	0.785	31.399	6.80E-05	NA
rs6870983	5	87697533	C	T	0.236	0.020	0.003	1.66E-15	0.043	0.030	0.158	63.434	1.37E-04	NA
rs687672	11	65649984	T	C	0.189	0.021	0.003	5.92E-14	0.020	0.033	0.549	56.399	1.22E-04	NA
rs6907923	6	51802669	G	A	0.341	-0.015	0.002	1.71E-10	-0.048	0.028	0.082	40.774	8.83E-05	NA
rs7016529	8	19806631	T	C	0.011	-0.102	0.009	1.94E-27	-0.079	0.106	0.456	117.778	2.55E-04	NA
rs703965	10	80955067	C	T	0.456	-0.012	0.002	8.25E-09	0.000	0.026	0.989	33.214	7.19E-05	NA
rs7075195	10	65050659	A	G	0.435	0.017	0.002	5.31E-16	0.003	0.026	0.899	65.676	1.42E-04	Educational attainment
rs7084454	10	21821274	G	A	0.338	-0.016	0.002	9.04E-13	0.050	0.028	0.075	51.042	1.10E-04	NA
rs7144011	14	79940383	G	T	0.207	-0.023	0.003	1.71E-17	-0.030	0.032	0.360	72.451	1.57E-04	NA
rs7161874	15	53163740	C	G	0.214	-0.015	0.003	4.11E-09	-0.012	0.029	0.688	34.573	7.48E-05	NA
rs7186893	16	24806420	G	T	0.267	0.020	0.002	3.04E-16	-0.019	0.029	0.501	66.779	1.45E-04	Mood swings
rs7206608	16	82872628	C	G	0.313	-0.013	0.002	1.26E-08	0.018	0.028	0.523	32.386	7.01E-05	NA
rs7212293	17	65847325	A	G	0.216	-0.022	0.003	4.05E-17	-0.029	0.032	0.364	70.753	1.53E-04	NA
rs7241918	18	47160953	G	T	0.163	0.018	0.003	7.52E-13	-0.072	0.037	0.048	51.403	1.11E-04	NA
rs7323827	13	94116675	A	G	0.304	0.013	0.002	4.59E-09	0.001	0.028	0.980	34.358	7.44E-05	NA
rs741959	1	47676233	A	G	0.402	0.014	0.002	6.19E-11	0.005	0.026	0.838	42.760	9.26E-05	NA
rs7488867	12	103699685	C	T	0.281	0.015	0.003	1.57E-09	0.000	0.029	0.991	36.447	7.89E-05	NA
rs7515635	1	42408070	T	C	0.471	0.012	0.002	4.68E-09	0.010	0.026	0.695	34.317	7.43E-05	NA
rs7549987	1	80789011	C	T	0.299	-0.014	0.002	4.02E-09	0.008	0.029	0.791	34.614	7.49E-05	NA
rs7581217	2	25524944	T	C	0.370	0.013	0.002	5.93E-09	-0.011	0.028	0.691	33.857	7.33E-05	NA
rs7601028	2	642499	C	G	0.174	-0.046	0.003	1.02E-52	-0.045	0.034	0.183	233.512	5.05E-04	NA
rs7638388	3	47914974	C	T	0.088	-0.024	0.004	1.01E-10	0.076	0.047	0.106	41.794	9.05E-05	NA
rs7642876	3	15883094	A	G	0.404	0.012	0.002	1.35E-08	-0.043	0.027	0.108	32.265	6.98E-05	NA
rs7668263	4	143781835	T	C	0.382	-0.012	0.002	4.21E-08	-0.032	0.027	0.231	30.049	6.50E-05	NA
rs779655	5	170495158	G	C	0.272	-0.014	0.002	1.21E-08	0.049	0.029	0.091	32.464	7.03E-05	NA

rs7824519	8	14222796	T	G	0.324	-0.013	0.002	1.51E-08	0.034	0.028	0.214	32.035	6.93E-05	NA
rs7928842	11	47566352	T	C	0.422	-0.020	0.002	2.30E-20	0.005	0.026	0.852	85.511	1.85E-04	NA
rs7947951	11	13356030	A	G	0.297	-0.014	0.002	1.59E-10	0.000	0.028	0.986	40.911	8.86E-05	NA
rs7962636	12	116450006	T	C	0.355	0.013	0.002	1.32E-08	0.019	0.027	0.482	32.308	6.99E-05	NA
rs8015400	14	25930988	C	A	0.348	-0.018	0.002	8.13E-15	0.044	0.028	0.106	60.303	1.31E-04	NA
rs8055138	16	28891465	C	T	0.329	-0.030	0.002	1.86E-35	-0.014	0.027	0.611	154.432	3.34E-04	Educational attainment; Intelligence
rs808820	12	89744826	A	G	0.116	-0.022	0.004	8.37E-10	-0.045	0.040	0.255	37.671	8.15E-05	NA
rs818524	1	85201228	T	C	0.289	-0.013	0.002	2.84E-08	-0.004	0.029	0.897	30.812	6.67E-05	NA
rs8192675	3	170724883	T	C	0.289	-0.013	0.002	4.30E-08	0.000	0.028	0.998	30.007	6.50E-05	NA
rs891387	18	21103909	T	C	0.473	0.021	0.002	3.25E-21	0.039	0.026	0.136	89.383	1.93E-04	Educational attainment
rs906673	3	12898941	C	T	0.389	0.013	0.002	3.67E-09	0.011	0.026	0.686	34.789	7.53E-05	NA
rs9277972	6	33276770	A	T	0.168	-0.016	0.003	1.55E-08	0.042	0.033	0.208	31.995	6.93E-05	NA
rs938875	15	67952922	A	G	0.408	-0.019	0.002	3.72E-18	0.032	0.026	0.229	75.463	1.63E-04	NA
rs9478499	6	154345289	T	A	0.147	-0.019	0.003	1.35E-10	0.015	0.035	0.672	41.238	8.93E-05	NA
rs9556979	13	99241507	T	G	0.324	-0.013	0.002	4.53E-09	0.017	0.028	0.552	34.382	7.44E-05	NA
rs9630985	2	181607676	A	C	0.349	-0.014	0.002	9.30E-11	-0.005	0.028	0.861	41.963	9.08E-05	NA
rs9686661	5	55861786	C	T	0.186	-0.023	0.003	4.65E-18	-0.018	0.033	0.597	75.025	1.62E-04	NA
rs9872156	3	176337348	G	A	0.260	-0.013	0.002	4.04E-08	0.027	0.030	0.364	30.129	6.52E-05	NA
rs987237	6	50803050	A	G	0.181	-0.030	0.003	7.81E-26	0.034	0.033	0.315	110.449	2.39E-04	NA
rs9947450	18	58052665	C	T	0.030	0.062	0.007	1.22E-21	0.011	0.068	0.868	91.321	1.98E-04	NA
rs998584	6	43757896	C	A	0.496	-0.013	0.002	9.99E-11	0.032	0.028	0.256	41.823	9.05E-05	NA
rs9987289	8	9183358	A	G	0.075	0.022	0.004	1.84E-09	0.002	0.047	0.967	36.136	7.82E-05	NA

Genomic positions reported in the GWAS of Mets refer to human reference assembly (GRCh37/hg19).

Abbreviations: MetS, Metabolic syndrome; TS, Tourette's syndrome; SNPs, Single nucleotide polymorphisms; CHR, Chromosome; EA, Effect allele; OA, Other allele; EAF, Effect allele frequency; SE, Standard error; PVE, Proportion of phenotypic variance explained.

SNPs that are unavailable in the outcome GWAS:rs1046080,rs705986,rs1412239, among them, rs115504677,rs10968576 and rs1216548 were substitute by proxy SNPs: rs1046080,rs1412239 and rs705986.

Palindromic and not-inferrable SNPs: rs1046080, rs1412239, rs705986, rs1937433, rs2253310, rs6916318.

2 SNPs were detected by PRESSO outlier test, and 20 SNPs were detected by MR-Radial outlier test.

Supplementary Table12. Genetic instruments for the causal relationship of ADHD and Mets .

SNP	CHR	Position	EA	OA	EAF	Beta for exposure	SE for exposure	P value for exposure	Beta for outcome	SE for outcome	P value for outcome	F statistics	PVE	Association with potential confounding factors
rs10022287	4	34094207	T	C	0.901	-0.075	0.018	3.68E-05	-0.006	0.003	0.056	16.983	7.53E-05	NA
rs10039321	5	166998385	T	C	0.310	0.043	0.010	1.92E-05	0.005	0.002	0.043	18.297	8.11E-05	NA
rs10177879	2	195749647	C	T	0.971	-0.132	0.029	6.39E-06	-0.012	0.006	0.058	20.436	9.06E-05	NA
rs10191574	2	174743152	G	A	0.815	-0.052	0.012	1.60E-05	-0.005	0.003	0.053	18.777	8.32E-05	NA
rs10479161	5	136941394	A	T	0.863	0.065	0.014	1.63E-06	-0.002	0.003	0.451	22.856	1.01E-04	NA
rs10507517	13	43404546	A	G	0.948	-0.102	0.023	1.09E-05	0.004	0.005	0.411	19.383	8.59E-05	NA
rs10516955	4	95694498	G	T	0.925	-0.074	0.018	3.34E-05	-0.008	0.005	0.083	17.181	7.62E-05	NA
rs10752940	1	184630291	T	C	0.397	0.041	0.010	2.71E-05	0.006	0.002	0.010	17.523	7.77E-05	NA
rs10772947	12	16746127	A	G	0.492	0.041	0.010	2.91E-05	0.003	0.002	0.111	17.523	7.77E-05	NA
rs10773767	12	130824083	G	A	0.751	-0.045	0.011	4.44E-05	-0.003	0.003	0.297	16.516	7.32E-05	NA
rs10783934	12	40017522	A	G	0.144	-0.059	0.014	2.18E-05	-0.004	0.003	0.224	18.079	8.02E-05	NA
rs10808300	7	82408144	G	A	0.526	-0.041	0.009	1.09E-05	-0.001	0.002	0.787	19.120	8.48E-05	NA
rs10815977	9	8860355	G	A	0.500	-0.042	0.010	2.07E-05	-0.004	0.002	0.048	18.276	8.10E-05	NA
rs10832706	11	16994792	C	T	0.675	-0.042	0.010	2.76E-05	0.001	0.002	0.709	17.640	7.82E-05	NA
rs10833285	11	20327608	C	T	0.742	-0.049	0.011	1.16E-05	0.001	0.002	0.538	19.172	8.50E-05	NA
rs10897553	11	79822161	G	A	0.598	-0.045	0.011	3.19E-05	-0.001	0.002	0.510	17.273	7.66E-05	NA
rs10935663	3	146960105	A	G	0.828	0.054	0.013	1.92E-05	0.005	0.003	0.067	18.385	8.15E-05	NA
rs10992792	9	96352243	T	A	0.642	0.044	0.010	6.30E-06	-0.002	0.002	0.484	20.386	9.04E-05	NA
rs11000805	10	75663351	G	C	0.816	0.053	0.012	1.48E-05	-0.002	0.003	0.502	18.657	8.27E-05	NA
rs11037421	11	43381417	C	A	0.855	-0.059	0.014	1.49E-05	0.002	0.003	0.556	18.818	8.34E-05	NA
rs1106258	9	122605088	C	T	0.887	0.065	0.016	3.33E-05	0.006	0.003	0.062	17.300	7.67E-05	NA
rs11111200	12	102587924	C	A	0.907	-0.082	0.016	6.48E-07	0.002	0.004	0.555	24.698	1.09E-04	NA
rs11254284	10	16978429	G	A	0.363	0.057	0.011	5.75E-07	0.001	0.002	0.574	25.087	1.11E-04	NA
rs11593126	10	95743087	A	G	0.914	-0.075	0.017	6.06E-06	-0.008	0.004	0.068	20.521	9.10E-05	Systolic blood pressure
rs11773863	7	70511130	G	C	0.696	0.045	0.010	8.34E-06	-0.002	0.002	0.503	19.814	8.78E-05	NA
rs12022460	1	91202257	G	A	0.755	0.045	0.011	2.60E-05	-0.002	0.003	0.432	17.864	7.92E-05	NA
rs12105131	2	116396058	C	G	0.914	0.079	0.017	4.65E-06	0.000	0.004	0.919	20.958	9.29E-05	NA
rs12116904	1	76481688	T	C	0.650	-0.046	0.010	3.66E-06	0.002	0.002	0.289	21.249	9.42E-05	NA
rs12215808	6	93851462	T	C	0.730	0.048	0.011	4.91E-06	0.004	0.002	0.059	20.766	9.21E-05	NA
rs12475774	2	181353621	G	T	0.982	0.143	0.033	1.27E-05	0.002	0.007	0.804	19.071	8.46E-05	NA
rs12514898	5	106727920	T	C	0.927	-0.076	0.018	1.80E-05	-0.004	0.005	0.491	18.388	8.15E-05	NA
rs12522004	5	145934483	G	A	0.681	-0.042	0.010	3.69E-05	-0.002	0.002	0.329	17.115	7.59E-05	NA
rs12662117	6	113297902	G	A	0.834	0.055	0.013	2.26E-05	0.002	0.003	0.516	18.046	8.00E-05	NA
rs12807357	11	57656902	C	T	0.675	-0.042	0.010	3.08E-05	-0.001	0.002	0.681	17.552	7.78E-05	NA
rs12900589	15	57373236	T	C	0.395	-0.049	0.010	2.86E-07	0.000	0.002	0.901	26.154	1.16E-04	NA
rs131024	22	49174763	G	T	0.728	0.044	0.011	4.02E-05	0.004	0.002	0.049	16.989	7.53E-05	NA
rs13163845	5	3264389	T	C	0.846	0.071	0.014	3.11E-07	-0.001	0.003	0.728	26.324	1.17E-04	NA
rs1335762	1	26485291	T	C	0.586	-0.044	0.010	4.46E-06	0.000	0.002	0.821	21.101	9.36E-05	NA
rs1352724	5	107083487	C	A	0.782	0.050	0.012	1.87E-05	0.004	0.003	0.108	18.212	8.07E-05	NA
rs1357304	7	68023220	A	G	0.177	0.059	0.014	4.63E-05	0.002	0.003	0.376	16.619	7.37E-05	NA
rs1366537	16	62873194	A	G	0.669	-0.045	0.010	1.21E-05	-0.005	0.002	0.027	19.119	8.48E-05	NA
rs1387671	14	63058891	T	G	0.490	0.046	0.009	1.18E-06	0.004	0.002	0.031	23.745	1.05E-04	NA
rs1414203	9	11340708	G	A	0.720	-0.050	0.010	1.26E-06	-0.003	0.002	0.260	23.376	1.04E-04	NA
rs1515736	3	161378009	C	A	0.910	0.073	0.016	7.06E-06	0.003	0.004	0.465	20.277	8.99E-05	NA
rs16949354	16	79074600	A	G	0.935	-0.080	0.019	3.26E-05	-0.005	0.004	0.194	17.275	7.66E-05	NA
rs17015512	2	78840783	T	C	0.612	-0.049	0.010	3.22E-07	-0.004	0.002	0.050	26.036	1.15E-04	NA
rs17191698	6	114455998	G	A	0.947	0.087	0.021	1.91E-05	-0.002	0.004	0.683	18.217	8.08E-05	NA
rs17400325	2	178565913	T	C	0.963	0.124	0.028	1.22E-05	0.011	0.004	0.007	19.167	8.50E-05	Basal metabolic rate
rs17576773	4	112217523	C	T	0.888	0.096	0.015	1.63E-10	0.000	0.003	0.969	40.673	1.80E-04	NA

rs17652815	1	199640708	T	C	0.953	-0.098	0.023	2.62E-05	-0.001	0.005	0.837	17.618	7.81E-05	NA
rs17718444	3	71499401	C	T	0.695	0.061	0.010	2.87E-09	0.001	0.002	0.540	35.303	1.57E-04	NA
rs17758391	2	173795967	G	A	0.687	0.054	0.011	2.44E-07	0.006	0.002	0.011	26.939	1.19E-04	NA
rs1956812	14	27300322	A	C	0.976	0.144	0.033	1.40E-05	0.000	0.006	0.973	18.910	8.38E-05	NA
rs1957651	14	32850171	T	G	0.031	0.121	0.028	2.10E-05	-0.001	0.006	0.867	18.151	8.05E-05	NA
rs2063640	3	102203045	C	A	0.905	0.089	0.020	9.36E-06	0.000	0.004	0.935	19.588	8.68E-05	NA
rs2115442	5	154775995	C	G	0.745	-0.046	0.011	1.96E-05	0.001	0.002	0.617	18.324	8.12E-05	NA
rs216237	12	287936	A	G	0.967	0.113	0.027	2.45E-05	-0.008	0.006	0.186	17.880	7.93E-05	NA
rs2216053	16	52296798	T	G	0.730	0.043	0.011	4.86E-05	-0.001	0.002	0.607	16.377	7.26E-05	NA
rs228619	4	103569283	A	G	0.472	-0.044	0.009	2.48E-06	-0.002	0.002	0.386	22.407	9.93E-05	NA
rs2293445	12	49398862	G	A	0.639	0.043	0.010	1.21E-05	-0.002	0.002	0.359	19.287	8.55E-05	NA
rs2391769	1	96978961	A	G	0.350	-0.048	0.010	5.74E-07	-0.003	0.002	0.242	25.103	1.11E-04	NA
rs2651569	4	101508622	T	C	0.463	0.047	0.009	7.11E-07	0.003	0.002	0.230	24.573	1.09E-04	NA
rs2788178	10	115057896	T	G	0.553	0.039	0.010	3.10E-05	0.004	0.002	0.043	17.288	7.66E-05	NA
rs283469	2	233656997	G	T	0.643	-0.054	0.011	1.42E-06	0.002	0.002	0.402	23.157	1.03E-04	Basal metabolic rate
rs3019310	8	107306869	C	G	0.698	-0.044	0.010	1.72E-05	0.003	0.002	0.230	18.330	8.13E-05	NA
rs3105377	7	68837002	C	T	0.364	0.040	0.010	3.33E-05	0.000	0.002	0.864	17.360	7.70E-05	NA
rs3117763	1	177723931	G	A	0.523	0.040	0.009	2.43E-05	0.002	0.002	0.267	17.925	7.95E-05	NA
rs330942	8	9020439	A	C	0.652	-0.041	0.010	3.48E-05	0.001	0.002	0.767	17.058	7.56E-05	NA
rs342927	3	146293474	T	G	0.307	0.049	0.012	2.36E-05	0.001	0.002	0.574	17.989	7.98E-05	NA
rs3739239	8	142221874	A	G	0.886	0.083	0.020	3.33E-05	0.009	0.003	0.005	17.222	7.64E-05	NA
rs3770884	2	36669071	T	C	0.860	0.059	0.014	1.35E-05	0.002	0.003	0.439	18.841	8.35E-05	NA
rs3782470	12	51755790	T	C	0.103	-0.072	0.015	2.26E-06	0.004	0.003	0.250	22.205	9.84E-05	NA
rs3814526	9	130910687	A	G	0.953	-0.097	0.023	2.32E-05	-0.011	0.005	0.027	17.940	7.95E-05	NA
rs3890134	5	106804928	G	A	0.862	0.058	0.014	3.16E-05	0.006	0.003	0.044	17.421	7.72E-05	NA
rs4264787	4	160486554	T	C	0.622	0.045	0.011	3.96E-05	0.004	0.002	0.035	17.037	7.55E-05	NA
rs4356816	3	143104736	C	T	0.232	-0.049	0.011	7.30E-06	-0.002	0.003	0.345	19.959	8.85E-05	NA
rs4684905	3	13118489	A	G	0.593	0.047	0.010	7.06E-07	-0.001	0.002	0.780	24.378	1.08E-04	NA
rs4733830	8	129104321	C	A	0.440	-0.040	0.009	2.17E-05	0.001	0.002	0.630	18.013	7.99E-05	NA
rs4858690	3	25075058	G	A	0.365	-0.043	0.010	1.04E-05	0.000	0.002	0.877	19.377	8.59E-05	NA
rs4866533	5	3225839	C	G	0.653	0.051	0.010	2.23E-07	0.000	0.002	0.827	26.868	1.19E-04	NA
rs4884	19	45810035	A	G	0.282	-0.052	0.011	5.86E-07	-0.003	0.002	0.215	24.714	1.10E-04	NA
rs4916723	5	87854395	A	C	0.553	-0.085	0.011	9.48E-15	-0.003	0.002	0.227	60.128	2.67E-04	NA
rs4925811	8	145802447	T	G	0.515	-0.058	0.010	8.30E-09	0.001	0.002	0.764	32.977	1.46E-04	NA
rs4936204	11	133588418	G	A	0.741	-0.053	0.012	4.82E-06	0.000	0.002	0.938	20.749	9.20E-05	NA
rs4966011	15	99204006	G	A	0.585	-0.052	0.011	1.74E-06	-0.002	0.002	0.281	22.675	1.01E-04	NA
rs4980964	12	818549	G	A	0.727	0.045	0.011	1.91E-05	0.006	0.002	0.012	18.101	8.03E-05	NA
rs6082363	20	21250843	T	C	0.296	0.070	0.010	4.38E-12	0.002	0.002	0.316	48.447	2.15E-04	Basal metabolic rate
rs6495932	15	38191331	G	C	0.349	-0.041	0.010	4.05E-05	0.001	0.002	0.600	16.883	7.49E-05	NA
rs6567054	18	56768996	A	C	0.194	-0.049	0.012	4.06E-05	-0.002	0.002	0.529	16.739	7.42E-05	NA
rs6671394	1	107343141	G	A	0.304	-0.043	0.010	2.23E-05	-0.004	0.002	0.097	17.873	7.92E-05	NA
rs6757553	2	209711492	T	C	0.279	0.052	0.011	7.11E-07	-0.003	0.002	0.222	24.342	1.08E-04	NA
rs6801864	3	37257242	A	G	0.525	0.041	0.009	9.95E-06	0.005	0.002	0.010	19.717	8.74E-05	NA
rs6812106	4	113921105	C	T	0.845	0.057	0.013	9.99E-06	0.001	0.003	0.660	19.527	8.66E-05	NA
rs6922489	6	166262523	G	A	0.416	0.040	0.010	3.29E-05	0.000	0.002	0.888	17.277	7.66E-05	NA
rs6993410	8	76805453	T	C	0.951	-0.097	0.021	6.09E-06	-0.007	0.005	0.170	20.376	9.03E-05	NA
rs7036801	9	9155721	C	T	0.864	-0.058	0.014	4.07E-05	0.004	0.003	0.235	16.799	7.45E-05	NA
rs7106	6	43608386	C	T	0.697	0.042	0.010	3.74E-05	0.002	0.002	0.287	17.131	7.60E-05	NA
rs7119	15	77777632	C	T	0.565	-0.042	0.009	5.90E-06	-0.004	0.002	0.080	20.342	9.02E-05	NA
rs724973	9	121788515	C	T	0.681	0.056	0.012	4.44E-06	0.005	0.002	0.020	21.111	9.36E-05	NA
rs7485057	12	48578809	T	C	0.247	0.050	0.011	3.79E-06	-0.001	0.002	0.707	21.433	9.50E-05	NA

rs7506904	18	50625779	G	A	0.343	-0.056	0.010	1.24E-08	-0.001	0.002	0.700	32.541	1.44E-04	NA
rs7510312	21	22257478	G	A	0.694	-0.044	0.010	3.00E-05	0.000	0.002	0.946	17.497	7.76E-05	NA
rs7534324	1	96221367	G	A	0.848	-0.067	0.013	4.83E-07	-0.007	0.003	0.021	25.373	1.12E-04	NA
rs7607172	2	100492135	T	C	0.571	-0.039	0.009	2.72E-05	-0.001	0.002	0.746	17.565	7.79E-05	NA
rs7686280	4	94162154	G	T	0.746	-0.049	0.011	1.74E-05	-0.002	0.003	0.544	18.326	8.12E-05	NA
rs7696413	4	163733174	C	G	0.296	-0.047	0.011	1.09E-05	-0.001	0.002	0.543	19.326	8.57E-05	NA
rs7720441	5	123849567	A	G	0.572	0.039	0.010	3.72E-05	0.000	0.002	0.822	17.204	7.63E-05	NA
rs7803385	7	11501136	C	T	0.930	-0.092	0.020	3.92E-06	0.004	0.004	0.355	21.356	9.47E-05	NA
rs7804105	7	19282268	C	T	0.942	-0.084	0.020	2.44E-05	-0.004	0.005	0.407	17.827	7.90E-05	NA
rs7855252	9	120537788	T	A	0.291	0.050	0.010	1.35E-06	0.000	0.002	0.871	23.210	1.03E-04	NA
rs791902	6	33702617	G	A	0.477	-0.040	0.009	1.80E-05	-0.005	0.002	0.018	18.382	8.15E-05	NA
rs7929518	11	85980958	A	G	0.223	-0.049	0.011	1.01E-05	-0.006	0.002	0.009	19.372	8.59E-05	NA
rs7938490	11	128829584	T	C	0.373	-0.049	0.010	3.99E-07	0.000	0.002	0.917	25.631	1.14E-04	NA
rs7953911	12	49948500	T	C	0.906	0.075	0.018	3.54E-05	0.008	0.003	0.013	17.165	7.61E-05	NA
rs8085882	18	22743899	A	G	0.516	0.052	0.010	5.37E-08	-0.001	0.002	0.585	29.678	1.32E-04	NA
rs820987	2	57762227	A	T	0.903	0.067	0.015	1.21E-05	0.006	0.004	0.124	19.212	8.52E-05	NA
rs859065	1	95352030	T	C	0.196	-0.049	0.012	2.95E-05	0.001	0.003	0.784	17.540	7.78E-05	NA
rs9452620	6	95026172	C	T	0.958	-0.104	0.024	1.98E-05	-0.001	0.006	0.904	18.210	8.07E-05	NA
rs9571014	13	64303536	C	T	0.981	0.158	0.033	1.88E-06	-0.004	0.009	0.675	22.706	1.01E-04	NA
rs9638734	7	16655632	C	T	0.814	0.056	0.014	3.97E-05	0.002	0.003	0.536	16.836	7.46E-05	NA
rs9767122	6	150104689	T	G	0.635	0.046	0.011	3.57E-05	0.002	0.002	0.397	17.171	7.61E-05	NA
rs9809370	3	30552292	G	C	0.643	0.047	0.010	3.68E-06	-0.001	0.002	0.698	21.565	9.56E-05	NA
rs9840386	3	192074068	C	T	0.524	0.042	0.009	6.79E-06	-0.002	0.002	0.343	20.252	8.98E-05	NA
rs9902312	17	65070304	T	C	0.665	-0.047	0.010	1.71E-06	0.001	0.002	0.609	22.727	1.01E-04	NA
rs9969232	7	114158954	G	A	0.344	-0.068	0.010	9.98E-12	0.001	0.002	0.523	46.649	2.07E-04	NA
rs9984627	21	34471603	G	A	0.625	-0.051	0.010	1.22E-07	-0.003	0.002	0.144	27.974	1.24E-04	NA
rs9999830	4	109708724	A	G	0.858	-0.061	0.013	5.84E-06	-0.002	0.003	0.524	20.516	9.10E-05	NA

Genomic positions reported in the GWAS of ADHD to human reference assembly (GRCh37/hg19).

Abbreviations: ADHD, Attention-deficit/hyperactivity disorder; MetS, Metabolic syndrome; SNPs, Single nucleotide polymorphisms; CHR, Chromosome; EA, Effect allele; OA, Other allele; EAF, Effect allele frequency; SE, Standard error; PVE, Proportion of phenotypic variance explained.

Palindromic and not-inferrable SNPs:rs2282225, rs2298527, rs7318227, rs879275.

6 SNPs detected by PRESSO outlier test,and 25 SNPs were detected by MR-Radial outlier test.

Supplementary Table 13. Genetic instruments for the causal relationship of ANO and Mets .

SNP	CHR	Position	EA	OA	EAF	Beta for exposure	SE for exposure	P value for exposure	Beta for outcome	SE for outcome	P value for outcome	F statistics	PVE	Association with potential confounding factors
rs10882687	10	97675019	A	G	0.763	0.076	0.017	4.24E-06	9.94E-04	0.002	0.684	21.072	2.90E-04	NA
rs10978723	9	109894432	G	A	0.558	-0.061	0.014	1.20E-05	-2.72E-04	0.002	0.898	19.223	2.65E-04	NA
rs11047523	12	24754947	C	G	0.195	-0.079	0.019	1.96E-05	1.67E-03	0.003	0.525	18.129	2.50E-04	NA
rs11105079	12	89237358	C	T	0.027	0.216	0.052	2.82E-05	-5.23E-03	0.006	0.418	17.571	2.42E-04	NA
rs11109807	12	99747309	C	T	0.261	-0.066	0.016	3.26E-05	1.76E-03	0.002	0.456	17.342	2.39E-04	NA
rs11126614	2	77722736	A	G	0.269	-0.063	0.015	4.11E-05	2.99E-03	0.002	0.198	16.738	2.31E-04	NA
rs11673365	19	14450246	T	G	0.177	-0.089	0.021	1.62E-05	-4.22E-03	0.003	0.121	18.540	2.56E-04	NA
rs11680878	2	5466323	C	T	0.307	-0.060	0.015	4.66E-05	1.27E-03	0.002	0.573	16.603	2.29E-04	NA
rs11742575	5	51822955	T	C	0.490	-0.055	0.013	4.67E-05	-4.33E-03	0.002	0.036	16.663	2.30E-04	NA
rs11852633	15	88734346	C	T	0.179	0.072	0.018	3.51E-05	2.11E-03	0.003	0.435	17.021	2.35E-04	NA
rs11876006	18	24752893	C	T	0.777	0.067	0.016	4.05E-05	-2.28E-03	0.003	0.363	16.846	2.32E-04	NA
rs12345267	9	17386062	C	T	0.550	0.063	0.014	4.28E-06	4.23E-03	0.002	0.044	21.256	2.93E-04	NA
rs12477879	2	112969865	T	C	0.153	0.076	0.019	3.70E-05	6.82E-03	0.003	0.018	17.012	2.35E-04	NA
rs12737676	1	84204210	C	G	0.120	-0.090	0.022	4.94E-05	-5.62E-03	0.003	0.082	16.436	2.27E-04	NA
rs12983352	19	39540593	A	G	0.227	-0.070	0.017	2.49E-05	6.95E-05	0.002	0.978	17.723	2.44E-04	NA
rs13079706	3	111995872	G	T	0.336	-0.061	0.015	4.22E-05	-2.89E-03	0.002	0.188	16.701	2.30E-04	NA
rs13422947	2	23234798	A	G	0.175	0.088	0.019	3.50E-06	-3.13E-03	0.003	0.253	21.452	2.96E-04	NA
rs1405050	12	119443713	T	C	0.513	-0.059	0.013	8.86E-06	-2.72E-03	0.002	0.189	19.650	2.71E-04	NA
rs1539725	1	193153682	C	T	0.372	-0.073	0.014	1.26E-07	-3.75E-03	0.002	0.082	27.884	3.84E-04	NA
rs16963319	15	36816327	G	T	0.101	0.105	0.025	2.41E-05	-9.93E-05	0.003	0.977	17.892	2.47E-04	NA
rs16965238	16	9522660	G	A	0.166	-0.083	0.019	9.63E-06	-5.71E-03	0.003	0.042	19.655	2.71E-04	NA
rs170533	1	96819655	T	G	0.658	0.056	0.014	4.52E-05	-8.76E-04	0.002	0.689	16.647	2.30E-04	NA
rs17447029	1	37761401	A	G	0.280	-0.080	0.019	1.34E-05	-2.93E-05	0.002	0.990	18.840	2.60E-04	NA
rs17452122	5	106513762	C	T	0.196	0.086	0.019	1.00E-05	3.97E-03	0.003	0.131	19.561	2.70E-04	NA
rs17580614	9	122394119	G	A	0.258	-0.086	0.019	4.41E-06	-4.87E-03	0.002	0.042	21.132	2.91E-04	NA
rs1833556	5	52251254	A	C	0.041	-0.184	0.041	8.96E-06	-8.02E-03	0.005	0.129	19.752	2.72E-04	NA
rs1952966	14	32438484	A	G	0.470	-0.060	0.014	9.14E-06	-1.65E-03	0.002	0.428	19.624	2.71E-04	NA
rs2070093	2	215632256	G	A	0.818	-0.081	0.018	6.53E-06	-3.61E-04	0.003	0.901	20.326	2.80E-04	NA
rs2131959	10	75864372	C	G	0.750	0.077	0.015	3.04E-07	3.85E-04	0.002	0.872	26.420	3.64E-04	NA
rs2173141	13	82641339	C	T	0.592	0.062	0.014	9.68E-06	-1.67E-03	0.002	0.430	19.463	2.68E-04	NA
rs2178688	7	132974012	C	G	0.596	0.063	0.014	9.12E-06	-1.89E-03	0.002	0.375	19.835	2.73E-04	NA
rs2188244	7	103657436	A	C	0.530	-0.068	0.014	6.44E-07	-2.14E-03	0.002	0.304	24.784	3.42E-04	NA
rs2685776	2	217261334	G	A	0.500	-0.057	0.014	2.74E-05	-4.89E-03	0.002	0.018	17.503	2.41E-04	NA
rs2884594	12	4505826	G	A	0.228	-0.087	0.018	9.37E-07	-2.07E-03	0.002	0.406	23.940	3.30E-04	NA
rs3848726	20	44666595	T	G	0.349	0.066	0.014	4.44E-06	2.05E-03	0.002	0.348	21.171	2.92E-04	NA
rs4129585	8	143312933	C	A	0.555	0.059	0.013	9.42E-06	7.62E-04	0.002	0.715	19.582	2.70E-04	NA
rs4260895	8	10126348	A	C	0.741	-0.064	0.016	3.81E-05	-2.00E-03	0.002	0.402	16.944	2.34E-04	NA
rs4706643	6	77830117	A	C	0.027	0.181	0.043	3.01E-05	1.14E-03	0.006	0.859	17.431	2.40E-04	NA
rs4905230	14	95142083	C	T	0.926	0.114	0.026	1.02E-05	-2.95E-03	0.004	0.457	19.476	2.69E-04	NA
rs570333	11	78975286	T	C	0.509	-0.065	0.014	2.82E-06	1.49E-03	0.002	0.472	21.937	3.02E-04	NA
rs597879	5	3421732	A	G	0.610	0.057	0.014	4.31E-05	8.11E-04	0.002	0.702	16.640	2.29E-04	NA
rs622917	6	33702869	T	C	0.431	0.058	0.014	1.95E-05	4.11E-03	0.002	0.052	18.231	2.51E-04	NA
rs626930	11	74170831	A	G	0.825	0.076	0.019	5.00E-05	1.57E-03	0.003	0.564	16.344	2.25E-04	NA
rs641325	11	57681828	G	T	0.679	0.060	0.015	4.17E-05	-7.64E-04	0.002	0.732	16.663	2.30E-04	NA
rs6789500	3	20764696	C	T	0.249	0.091	0.018	2.55E-07	-7.26E-05	0.002	0.976	26.616	3.67E-04	NA
rs680109	11	105750709	A	C	0.380	-0.063	0.014	4.38E-06	1.20E-03	0.002	0.574	21.041	2.90E-04	NA
rs6977110	7	3441964	A	G	0.155	0.077	0.018	1.36E-05	-1.77E-03	0.003	0.565	18.906	2.61E-04	NA
rs7147039	14	54203649	A	G	0.035	-0.165	0.039	2.64E-05	-1.01E-02	0.006	0.082	17.695	2.44E-04	NA
rs725861	10	9063776	G	A	0.192	0.088	0.018	4.86E-07	-1.13E-03	0.003	0.667	25.345	3.49E-04	NA

rs7325923	13	24078596	T	A	0.104	-0.092	0.022	2.28E-05	6.91E-04	0.003	0.840	18.004	2.48E-04	NA
rs7329613	13	47831887	G	A	0.495	0.061	0.014	7.92E-06	2.51E-04	0.002	0.904	19.860	2.74E-04	NA
rs750350	11	112885342	T	G	0.131	-0.111	0.021	9.42E-08	-1.08E-03	0.003	0.726	28.426	3.92E-04	NA
rs7797895	7	93888893	C	A	0.211	0.078	0.017	2.33E-06	1.81E-04	0.003	0.944	22.235	3.07E-04	NA
rs7867933	9	83850716	G	A	0.042	-0.153	0.038	4.91E-05	-1.63E-03	0.005	0.753	16.448	2.27E-04	NA
rs879571	5	90079972	T	C	0.071	-0.114	0.028	3.59E-05	-4.44E-03	0.004	0.272	17.060	2.35E-04	NA
rs906313	15	98345558	C	A	0.334	-0.064	0.014	9.37E-06	-3.18E-03	0.002	0.152	19.632	2.71E-04	NA
rs916256	22	43620619	A	G	0.578	-0.059	0.014	1.72E-05	-1.94E-03	0.002	0.359	18.544	2.56E-04	NA
rs923768	8	19530963	C	T	0.524	-0.056	0.014	4.00E-05	-2.24E-03	0.002	0.282	16.833	2.32E-04	NA
rs9302300	15	82098978	C	T	0.787	0.068	0.017	3.65E-05	-1.96E-03	0.003	0.442	16.987	2.34E-04	NA
rs9309871	3	81496341	A	C	0.240	-0.078	0.016	8.58E-07	-2.36E-04	0.002	0.922	24.307	3.35E-04	NA
rs9439752	1	30114164	G	T	0.233	0.070	0.017	2.91E-05	2.91E-03	0.002	0.235	17.468	2.41E-04	NA
rs9530551	13	77035281	G	T	0.014	-0.228	0.055	3.14E-05	1.13E-02	0.009	0.230	17.344	2.39E-04	NA
rs9677559	2	23712008	A	G	0.057	-0.132	0.032	4.12E-05	6.65E-04	0.005	0.883	16.776	2.31E-04	NA
rs9784437	4	147216089	G	A	0.195	-0.083	0.017	8.01E-07	-4.79E-03	0.003	0.068	24.355	3.36E-04	NA
rs9821797	3	48718253	A	T	0.127	-0.157	0.020	6.99E-15	2.35E-03	0.003	0.449	60.697	8.36E-04	NA
rs9863605	3	24026423	C	T	0.265	0.062	0.015	4.33E-05	9.11E-07	0.002	1.000	16.695	2.30E-04	NA
rs9874207	3	71019750	C	T	0.488	-0.081	0.015	2.05E-08	1.04E-04	0.002	0.960	31.435	4.33E-04	NA
rs9963279	18	68811663	A	G	0.018	-0.257	0.057	6.33E-06	3.71E-03	0.008	0.662	20.361	2.81E-04	NA

Genomic positions reported in the GWAS of ANO refer to human reference assembly (GRCh37/hg19).

Abbreviations: ANO, Anorexia nervosa; MetS, Metabolic syndrome; SNPs, Single nucleotide polymorphisms; CHR, Chromosome; EA, Effect allele; OA, Other allele; EAF, Effect allele frequency; SE, Standard error; PVE, Proportion of phenotypic variance explained.

Palindromic and not-inferrable SNPs: rs1660242, rs2068447, rs28380.

4 SNPs detected by PRESSO outlier test, and 10 SNPs were detected by MR-Radial outlier test.

Supplementary Table 14. Genetic instruments for the causal relationship of MDD and Mets.

SNP	CHR	Position	EA	OA	EAF	Beta for exposure	SE for exposure	P value for exposure	Beta for outcome	SE for outcome	P value for outcome	F statistics	PVE	Association with potential confounding factors
rs10174534	2	2449771	A	G	0.551	-0.044	0.009	1.49E-06	0.003	0.002	0.216	23.062	1.62E-04	NA
rs10819460	9	101685520	T	C	0.860	-0.053	0.013	4.91E-05	0.003	0.003	0.376	16.371	1.15E-04	NA
rs1086490	12	40382120	A	G	0.888	0.059	0.014	3.06E-05	0.000	0.003	0.915	17.520	1.23E-04	NA
rs10978733	9	109923051	T	G	0.856	0.053	0.013	3.22E-05	0.004	0.003	0.167	17.274	1.21E-04	NA
rs11077049	16	6604090	A	G	0.025	0.142	0.030	2.56E-06	-0.003	0.007	0.625	22.194	1.56E-04	NA
rs11235880	11	73435296	A	C	0.198	0.050	0.011	9.36E-06	-0.003	0.003	0.267	19.578	1.37E-04	NA
rs11612312	12	52349088	T	C	0.787	-0.045	0.011	4.99E-05	0.000	0.003	0.935	16.510	1.16E-04	NA
rs11957496	5	117074375	A	C	0.665	0.040	0.010	2.78E-05	0.004	0.002	0.075	17.536	1.23E-04	NA
rs12036905	1	79944324	C	G	0.013	0.197	0.045	1.07E-05	-0.008	0.008	0.336	19.336	1.36E-04	NA
rs12132899	1	223840617	T	C	0.069	-0.073	0.017	2.56E-05	0.000	0.004	0.964	17.701	1.24E-04	NA
rs1219935	9	25902763	T	C	0.092	0.074	0.016	2.00E-06	-0.007	0.004	0.076	22.732	1.59E-04	NA
rs12421970	11	127816603	A	G	0.264	0.042	0.010	3.27E-05	-0.002	0.002	0.345	17.200	1.21E-04	NA
rs1256112	14	64814311	T	C	0.566	0.044	0.009	9.44E-07	-0.004	0.002	0.068	23.908	1.68E-04	NA
rs1470769	8	96139617	T	C	0.307	0.042	0.010	2.50E-05	0.004	0.002	0.091	17.725	1.24E-04	NA
rs1479540	3	2629477	A	G	0.871	-0.054	0.013	4.85E-05	0.004	0.003	0.235	16.419	1.15E-04	NA
rs1508522	11	42435951	T	C	0.248	0.045	0.011	2.71E-05	0.004	0.002	0.122	17.764	1.25E-04	NA
rs1585897	12	66383320	A	C	0.549	0.039	0.009	1.69E-05	-0.001	0.002	0.508	18.491	1.30E-04	Basal metabolic rate
rs17011286	4	86931379	A	G	0.955	-0.094	0.023	3.40E-05	0.000	0.005	0.941	17.185	1.20E-04	NA
rs17043773	2	53133809	T	C	0.279	0.046	0.010	4.51E-06	0.004	0.002	0.121	20.981	1.47E-04	NA
rs17182683	6	131461576	T	C	0.259	0.043	0.011	3.65E-05	-0.001	0.002	0.718	16.930	1.19E-04	NA
rs17499892	7	136692467	A	C	0.615	-0.050	0.009	6.64E-08	-0.001	0.002	0.616	29.301	2.05E-04	NA
rs17807255	3	159450722	T	C	0.974	0.129	0.031	2.60E-05	0.000	0.007	0.998	17.712	1.24E-04	NA
rs1859199	16	24317856	C	G	0.757	-0.045	0.011	2.62E-05	0.000	0.002	0.977	17.611	1.23E-04	NA
rs1862743	16	60743834	A	C	0.486	-0.037	0.009	4.88E-05	0.004	0.002	0.070	16.539	1.16E-04	NA
rs1888286	9	119726051	T	C	0.723	0.048	0.010	1.07E-06	0.003	0.002	0.225	23.807	1.67E-04	NA
rs1898711	2	216485141	A	T	0.810	0.050	0.011	1.36E-05	-0.004	0.003	0.158	19.010	1.33E-04	NA
rs1950829	14	42097937	A	G	0.496	0.054	0.009	8.51E-10	0.001	0.002	0.546	37.365	2.62E-04	NA
rs1955596	14	88820021	T	C	0.931	-0.080	0.019	1.82E-05	-0.002	0.005	0.713	18.288	1.28E-04	NA
rs1965183	2	134979966	A	G	0.596	-0.039	0.009	2.22E-05	-0.003	0.002	0.115	17.945	1.26E-04	NA
rs2589213	8	97539802	T	C	0.155	0.053	0.013	2.53E-05	-0.001	0.003	0.685	17.907	1.26E-04	NA
rs2784590	9	10510289	T	C	0.222	0.051	0.011	2.96E-06	0.003	0.003	0.252	21.867	1.53E-04	NA
rs281265	15	47675602	A	C	0.637	-0.046	0.009	8.73E-07	0.000	0.002	0.965	24.146	1.69E-04	NA
rs2855812	6	31472720	T	G	0.243	-0.052	0.011	9.00E-07	0.002	0.003	0.373	24.149	1.69E-04	NA
rs3794671	16	87745998	T	C	0.913	-0.069	0.017	3.57E-05	0.001	0.004	0.841	16.967	1.19E-04	NA
rs4141983	1	18122009	T	C	0.678	0.043	0.010	7.42E-06	-0.004	0.002	0.072	19.967	1.40E-04	NA
rs4810134	20	57078518	A	G	0.055	0.097	0.024	4.34E-05	0.001	0.004	0.897	16.720	1.17E-04	NA
rs6030655	20	41736851	T	C	0.910	-0.071	0.016	8.61E-06	0.002	0.004	0.556	19.719	1.38E-04	NA
rs615747	1	194225405	A	T	0.075	0.079	0.017	3.91E-06	-0.002	0.004	0.559	21.451	1.50E-04	NA
rs6832890	4	42106538	C	G	0.194	-0.060	0.011	1.09E-07	-0.003	0.003	0.198	28.410	1.99E-04	NA
rs6861686	5	125451538	T	C	0.852	0.051	0.012	3.60E-05	0.002	0.003	0.457	16.984	1.19E-04	NA
rs6939697	6	123400798	A	G	0.784	-0.046	0.011	2.49E-05	-0.002	0.002	0.348	17.812	1.25E-04	NA
rs7350298	9	73402790	A	C	0.246	0.044	0.011	3.02E-05	0.000	0.002	0.867	17.482	1.23E-04	NA
rs751	1	87857969	A	G	0.162	-0.051	0.012	3.57E-05	0.003	0.003	0.351	17.127	1.20E-04	NA
rs7528466	1	211898872	C	G	0.117	-0.060	0.014	3.43E-05	0.003	0.003	0.377	17.131	1.20E-04	NA
rs841779	1	177547209	A	G	0.870	0.054	0.013	3.83E-05	0.004	0.003	0.186	17.045	1.19E-04	NA
rs9292849	5	20024013	A	G	0.486	0.037	0.009	4.09E-05	-0.001	0.002	0.748	16.723	1.17E-04	NA

Genomic positions reported in the GWAS of MDD refer to human reference assembly (GRCh37/hg19)

Abbreviations: MDD, Major depressive disorder; MetS, Metabolic syndrome; SNPs, Single nucleotide polymorphisms; CHR, Chromosome; EA, Effect allele; OA, Other allele; EAF, Effect allele frequency; SE, Standard error; PVE, Proportion of phenotypic variance explained.

5 SNPs were detected by PRESSO outlier test, and 9 SNPs were detected by MR-Radial outlier test..

Supplementary Table 15. GWAS summary statistics of the replication study.

Psychiatric disorder	Abbreviation	phenocode	Type	No. of Participants	No. of Variants	Data Source
Attention deficit/hyperactivity disorder	ADHD	F5_ADHD	Cases	2 340	20 170 146	https://storage.googleapis.com/finngen-public-data-r9/summary_stats/finngen_R9_F5_ADHD.gz
			Controls	371 117		
Alcohol dependence	ALC	F5_ALCOHOL_DEPENDENCE	Cases	9 876	20 169 894	https://storage.googleapis.com/finngen-public-data-r9/summary_stats/finngen_R9_F5_ALCOHOL_DEPENDENCE.gz
			Controls	353 355		
Anorexia Nervosa	ANO	F5_ANOREX	Cases	1 897	20 169 986	https://storage.googleapis.com/finngen-public-data-r9/summary_stats/finngen_R9_F5_ANOREX.gz
			Controls	366 876		
Anxiety	ANX	F5_ALLANXIOUS	Cases	24 662	20 169 921	https://storage.googleapis.com/finngen-public-data-r9/summary_stats/finngen_R9_F5_ALLANXIOUS.gz
			Controls	337 577		
Autism spectrum disorder	ASD	KRA_PSY_AUTISM_EXMORE	Cases	564	20 166 885	https://storage.googleapis.com/finngen-public-data-r9/summary_stats/finngen_R9_KRA_PSY_AUTISM_EXMORE.gz
			Controls	277 526		
Bipolar Disorder	BIP	F5_BIPO	Cases	7 006	20 169 199	https://storage.googleapis.com/finngen-public-data-r9/summary_stats/finngen_R9_F5_BIPO.gz
			Controls	329 192		
Major depressive disorder	MDD	F5_DEPRESSIO	Cases	43 280	20 170 115	https://storage.googleapis.com/finngen-public-data-r9/summary_stats/finngen_R9_F5_DEPRESSIO.gz
			Controls	329 192		
schizophrenia	SCZ	F5_SCHZPHR	Cases	6 515	20 169 286	https://storage.googleapis.com/finngen-public-data-r9/summary_stats/finngen_R9_F5_SCHZPHR.gz
			Controls	364 160		
Post-traumatic stress disorder	PTSD	F5_PTSD	Cases	2 282	20 170 086	https://storage.googleapis.com/finngen-public-data-r9/summary_stats/finngen_R9_F5_PTSD.gz
			Controls	337 577		

Supplementary Table 16. Genome-wide genetic correlations across the 10 PDs using LDSC

Trait 1	Trait 2	rg	se	z	P value
ADHD	ALC	0.660	0.117	5.652	1.59E-08
ADHD	ANO	-0.075	0.033	-2.237	2.53E-02
ADHD	ANX	0.371	0.046	8.005	1.19E-15
ADHD	ASD	0.441	0.049	8.939	3.93E-19
ADHD	BIP	0.136	0.032	4.251	2.13E-05
ADHD	MDD	0.654	0.032	20.643	1.12E-94
ADHD	SCZ	0.203	0.024	8.555	1.17E-17
ADHD	PTSD	0.518	0.139	3.721	2.00E-04
ADHD	TS	0.229	0.045	5.123	3.01E-07
ALC	ANO	-0.031	0.097	-0.323	7.46E-01
ALC	ANX	0.697	0.464	1.503	1.33E-01
ALC	ASD	-0.098	0.109	-0.901	3.68E-01
ALC	BIP	0.235	0.062	3.787	2.00E-04
ALC	MDD	0.595	0.096	6.195	5.84E-10
ALC	SCZ	0.391	0.063	6.250	4.10E-10
ALC	PTSD	0.242	0.230	1.051	2.93E-01
ALC	TS	-0.007	0.117	-0.062	9.51E-01
ANO	ANX	-0.541	0.201	-2.696	7.00E-03
ANO	ASD	-0.110	0.053	-2.078	3.77E-02
ANO	BIP	-0.175	0.036	-4.813	1.49E-06
ANO	MDD	-0.335	0.043	-7.722	1.14E-14
ANO	SCZ	-0.223	0.026	-8.432	3.39E-17
ANO	PTSD	0.030	0.121	0.247	8.05E-01
ANO	TS	-0.103	0.063	-1.646	9.98E-02
ANX	ASD	-0.110	0.053	-2.078	3.77E-02
ANX	BIP	0.255	0.205	1.244	2.14E-01
ANX	MDD	0.437	0.049	8.857	8.19E-19
ANX	SCZ	0.761	0.337	2.255	2.41E-02
ANX	PTSD	-0.086	0.397	-0.216	8.29E-01
ANX	TS	-0.180	0.286	-0.632	5.28E-01
ASD	BIP	0.146	0.048	3.019	2.50E-03
ASD	MDD	0.411	0.045	9.184	4.14E-20
ASD	SCZ	0.261	0.037	6.998	2.59E-12
ASD	PTSD	0.239	0.141	1.691	9.08E-02
ASD	TS	0.129	0.061	2.119	3.41E-02
BIP	MDD	0.376	0.033	11.546	7.73E-31
BIP	SCZ	0.721	0.019	37.084	5.07E-301
BIP	PTSD	0.053	0.092	0.581	5.62E-01
BIP	TS	0.079	0.049	1.626	1.04E-01
MDD	SCZ	0.399	0.027	14.893	3.69E-50
MDD	PTSD	0.631	0.173	3.652	3.00E-04
MDD	TS	0.185	0.061	3.050	2.30E-03
SCZ	PTSD	0.130	0.082	1.574	1.16E-01
SCZ	TS	0.106	0.037	2.867	4.10E-03
PTSD	TS	-0.287	0.167	-1.717	8.60E-02

Note: Genetic correlations were estimated by LD Score regression. PDs:psychiatric disorders; rg = genetic correlation; se = standard error of rg; z = Z-score of the rg; P avlue= P-value of the rg.

Supplementary Table 17. Evaluation of instrumental variables.

Exposure	Outcome	No. IVs	F statistic	Global Test P value	Q Test P value	Egger intercept Test P value	PVE	Power
**Causality of Mets with psychiatirc disorders**								
MetS	ADHD	191	49.736	0.177	0.169	0.129	0.021	99.7%
MetS	ALC	235	52.024	0.980	0.975	0.118	0.026	62.3%
MetS	ANO	195	49.510	0.727	0.730	0.629	0.021	85.4%
MetS	ANX	230	51.897	0.997	0.998	0.767	0.026	43.2%
MetS	ASD	226	51.375	0.960	0.962	0.941	0.025	85.9%
MetS	BIP	202	51.867	0.894	0.888	0.818	0.023	86.7%
MetS	MDD	211	51.962	0.247	0.252	0.117	0.024	99.9%
MetS	SCZ	161	52.337	0.264	0.256	0.640	0.018	99.1%
MetS	PTSD	235	50.834	0.995	0.996	0.589	0.026	23.9%
MetS	TS	224	52.358	0.690	0.692	0.081	0.025	37.1%
**Causality of psychiatirc disorders with Mets**								
**Exposure**	**Outcome**	**No. IVs**	**F statistic**	**Global Test P value**	**Q Test P value**	**Egger intercept Test P value**	**PVE**	**Power**
ADHD	MetS	127	21.110	0.061	0.058	0.626	0.012	100.0%
ANO	MetS	68	20.246	0.632	0.622	0.138	0.019	100.0%
MDD	MetS	46	19.687	0.315	0.299	0.461	0.006	100.0%
**Replication causality study of Mets with psychiatirc disorders**								
**Exposure**	**Outcome**	**No. IVs**	**F statistic**	**Global Test P value**	**Q Test P value**	**Egger intercept Test P value**	**PVE**	**Power**
MetS	ADHD	236	52.082	0.351	0.992	0.608	0.027	29.9%
MetS	ALC	216	48.238	0.889	0.888	0.112	0.023	77.3%
MetS	ANO	234	50.907	0.381	0.994	0.158	0.026	24.7%
MetS	ANX	209	51.381	0.574	0.577	0.217	0.023	98.7%
MetS	ASD	231	52.379	0.273	0.995	0.693	0.026	10.3%
MetS	BIP	223	52.068	0.958	0.961	0.998	0.025	66.6%
MetS	MDD	196	53.244	0.654	0.659	0.529	0.023	100.0%
MetS	SCZ	232	51.552	0.088	0.946	0.554	0.026	65.2%
MetS	PTSD	226	51.621	0.998	0.998	0.692	0.025	27.8%
**Replication causality study of psychiatirc disorders with Mets**								
**Exposure**	**Outcome**	**No. IVs**	**F statistic**	**Global Test P value**	**Q Test P value**	**Egger intercept Test P value**	**PVE**	**Power**
ANO	MetS	14	18.9116	0.4040	0.3865	0.4411	0.0006	84.3%
MDD	MetS	79	20.9485	0.2878	0.2889	0.4895	0.0036	100.0%

Abbreviations: MetS, Metabolic syndrome; ADHD, Attention deficit/hyperactivity disorder; ALC, Alcohol dependence; ANO, Anorexia nervosa; ANX, Anxiety; ASD, Autism spectrum disorder; BIP, Bipolar disorder; MDD, Major depressive disorder; SCZ, Schizophrenia; PTSD, Post-traumatic stress disorder; TS, Tourette's syndrome; No. IVs, Number of instrumental variables; PVE, Proportion of phenotypic variance explained.

Supplementary Table 18. MR results.

UVMR sensitivity analyses

Exposure	Outcome	*P* threshold	No. IVs	MLE			WME			RAPS			IVW[a]		
				Estimate	SE	P value	Estimate	SE	P value	Estimate	SE	P value	Estimate	SE	P value
MetS	ADHD	5E-08	191	0.471	0.046	**4.36E-25**	0.328	0.080	**4.26E-05**	0.474	0.046	**<0.001**	0.477	0.048	**3.72E-23**
MetS	ALC		235	0.008	0.097	0.932	-0.029	0.173	0.866	0.008	0.098	0.933	0.039	0.091	0.668
MetS	ANO		195	0.349	0.066	**1.18E-07**	0.314	0.106	**2.89E-03**	0.348	0.067	**1.71E-07**	0.335	0.066	**4.11E-07**
MetS	ANX		230	0.171	0.105	0.103	0.217	0.185	0.243	0.171	0.107	0.108	0.190	0.094	0.043
MetS	ASD		226	0.004	0.060	0.949	-0.064	0.108	0.551	0.004	0.061	0.950	-0.005	0.057	0.923
MetS	BIP		202	0.066	0.061	0.285	0.073	0.104	0.484	0.065	0.062	0.293	0.073	0.059	0.215
MetS	MDD		211	0.205	0.040	**3.39E-07**	0.255	0.073	**4.82E-04**	0.207	0.041	**3.49E-07**	0.226	0.043	**1.05E-07**
MetS	SCZ		161	-0.018	0.044	0.692	-0.035	0.084	0.674	-0.018	0.045	0.690	-0.032	0.047	0.506
MetS	PTSD		235	0.241	0.166	0.147	-0.002	0.294	0.995	0.244	0.169	0.148	0.199	0.150	0.185
MetS	TS		224	0.013	0.113	0.906	0.116	0.207	0.576	0.013	0.114	0.906	-0.037	0.114	0.748
ADHD	MetS	5E-05	127	0.030	0.004	**2.19E-12**	0.022	0.006	**6.76E-04**	0.031	0.004	**9.50E-13**	0.028	0.005	**2.80E-09**
ANO	MetS		68	0.014	0.004	**9.14E-04**	0.004	0.006	0.441	0.014	0.004	**8.64E-04**	0.013	0.004	**7.53E-04**
MDD	MetS		46	-0.004	0.008	0.651	-0.002	0.011	0.847	-0.004	0.008	0.654	-0.001	0.008	0.865

UVMR replication analyses

Exposure	Outcome	*P* threshold	No. IVs	IVW			MLE			WME			RAPS		
				Estimate	SE	P value	Estimate	SE	P value	Estimate	SE	P value	Estimate	SE	P value
MetS	ADHD	5E-8	236	0.032	0.113	0.776	0.032	0.127	0.802	0.276	0.203	0.173	0.033	0.129	0.801
MetS	ALC		216	0.040	0.065	0.537	0.040	0.069	0.563	0.046	0.107	0.666	0.041	0.070	0.563
MetS	ANO		234	0.446	0.125	**3.49E-04**	0.450	0.142	**0.002**	0.490	0.226	**0.030**	0.453	0.144	**0.002**
MetS	ANX		209	0.087	0.043	0.045	0.088	0.044	0.048	0.107	0.085	0.205	0.089	0.045	0.047
MetS	ASD		231	0.516	0.247	0.036	0.515	0.282	0.068	0.602	0.446	0.177	0.524	0.286	0.067
MetS	BIP		223	-0.073	0.074	0.323	-0.074	0.081	0.362	-0.105	0.139	0.448	-0.074	0.082	0.368
MetS	MDD		196	-0.116	0.034	**0.001**	-0.117	0.035	**0.001**	-0.106	0.054	**0.051**	0.035	0.035	**0.001**
MetS	SCZ		232	0.232	0.105	0.027	0.236	0.114	0.039	0.332	0.205	0.106	0.235	0.116	0.042
MetS	PTSD		226	-0.198	0.114	0.081	-0.199	0.132	0.131	-0.056	0.211	0.791	-0.201	0.134	0.132
ANO	MetS	5E-5	14	-1.34E-04	0.004	0.971	-1.40E-04	0.004	0.970	0.001	0.005	0.851	-1.41E-04	0.004	0.971
MDD	MetS		79	0.003	0.007	0.656	0.003	0.007	0.642	-0.003	0.011	0.749	0.003	0.007	0.641

MVMR analyses

Exposure	Outcome	*P* threshold	No. IVs	IVW			Egger			Median			LASSO		
				Estimate	SE	P value	Estimate	SE	P value	Estimate	SE	P value	Estimate	SE	P value
BMI	ADHD	5E-8	1099	-0.031	0.037	0.408	-0.018	0.133	0.892	-0.015	0.043	0.733	-0.002	0.029	0.959
MetS				0.738	0.049	**2.31E-51**	0.738	0.049	**5.41E-51**	0.752	0.058	**4.54E-38**	0.707	0.039	**3.47E-75**
BMI	ANO		1107	-0.025	0.049	0.613	0.317	0.178	0.075	-0.087	0.062	0.158	-0.008	0.042	0.855
MetS				-0.672	0.065	**3.42E-25**	-0.683	0.065	**7.67E-62**	-0.665	0.081	**1.60E-16**	-0.648	0.055	**1.51E-31**
BMI	MDD		1123	0.165	0.031	**8.35E-08**	0.037	0.111	0.740	0.200	0.039	**3.02E-07**	0.201	0.027	**8.71E-14**
MetS				-0.010	0.041	0.799	-0.006	0.041	0.875	-0.033	0.051	0.515	-0.030	0.036	0.398

Abbreviations: MR: Mendelian randomization; UVMR: Univariate Mendelian randomization;MVMR: Multivariate Mendelian randomization;MetS, Metabolic syndrome; ADHD, Attention deficit/hyperactivity disorder; ALC, Alcohol dependence; ANO, Anorexia nervosa; ANX ,Anxiety; ASD, Autism spectrum disorder;BIP, Bipolar disorder; MDD, Major depressive disorder; SCZ, Schizophrenia; PTSD, Post-traumatic stress disorder; TS, Tourette's syndrome; BMI: Body mass index; SE, Standard error; No. IVs, Number of instrumental variables; P threshold, Threshold of the genome-wide association P value to be select as instrument variable; IVW, Inverse variance weighting; MLE, Maximum likelihood estimator; WME, Weighted median estimator; RAPS, Robust adjusted profile score.IVWa, Inverse variance weighting when ruling out potential pleiotropic SNPs that associated with potential known confounding factors.
